# Identification and Characterization of Cannabichromene’s Major Metabolite Following Incubation with Human Liver Microsomes

**DOI:** 10.3390/metabo14060329

**Published:** 2024-06-13

**Authors:** Alexandra M. Ward, Touraj Shokati, Jost Klawitter, Jelena Klawitter, Vu Nguyen, Laura Kozell, Atheir I. Abbas, David Jones, Uwe Christians

**Affiliations:** 1 Department of Pharmaceutical Sciences, Skaggs School of Pharmacy and Pharmaceutical Sciences, University of Colorado Anschutz Medical Campus, Aurora, CO 80045, USA; alexandra.ward@cuanschutz.edu (A.M.W.); vu.t.nguyen@cuanschutz.edu (V.N.); 2 iC42 Clinical Research and Development, Department of Anesthesiology, School of Medicine, University of Colorado Anschutz Medical Campus, Aurora, CO 80045, USAjost.klawitter@cuanschutz.edu (J.K.); jelena.klawitter@cuanschutz.edu (J.K.); 3 Department of Behavioral Neuroscience, Oregon Health & Science University, Portland, OR 97239, USA; kozellla@ohsu.edu (L.K.); abbasat@ohsu.edu (A.I.A.); 4 Department of Psychiatry, Oregon Health & Science University, Portland, OR 97239, USA; 5 Veterans Affairs Portland Health Care System, Portland, OR 97239, USA; 6 Department of Pharmacology, School of Medicine, University of Colorado Anschutz Medical Campus, Aurora, CO 80045, USA; david.jones@cuanschutz.edu

**Keywords:** cannabichromene, 2′-hydroxycannabicitran, cannabicitran, human drug metabolism, phase I metabolism, human liver microsomes, gas chromatography–mass spectrometry, nuclear magnetic resonance spectroscopy, molecular docking, CB_1_ receptor, CB_2_ receptor, binding

## Abstract

Cannabichromene (CBC) is a minor cannabinoid within the array of over 120 cannabinoids identified in the *Cannabis sativa* plant. While CBC does not comprise a significant portion of whole plant material, it is available to the public in a purified and highly concentrated form. As minor cannabinoids become more popular due to their potential therapeutic properties, it becomes crucial to elucidate their metabolism in humans. Therefore, the goal of this was study to identify the major CBC phase I-oxidized metabolite generated in vitro following incubation with human liver microsomes. The novel metabolite structure was identified as 2′-hydroxycannabicitran using gas chromatography–mass spectrometry and nuclear magnetic resonance spectroscopy. Following the identification, in silico molecular modeling experiments were conducted and predicted 2′-hydroxycannabicitran to fit in the orthosteric site of both the CB_1_ and CB_2_ receptors. When tested in vitro utilizing a competitive binding assay, the metabolite did not show significant binding to either the CB_1_ or CB_2_ receptors. Further work necessitates the determination of potential activity of CBC and the here-identified phase I metabolite in other non-cannabinoid receptors.

## 1. Introduction

The best-studied cannabinoids are Δ^9^-tetrahydrocannabinol (Δ^9^-THC) and cannabidiol (CBD). Δ^9^-THC is well known to be a partial agonist of the cannabinoid 2 receptor (CB_2_R) and the cannabinoid 1 receptor (CB_1_R), the latter of which is responsible for its psychotropic effects [1,2]. The activity of CBD is more complex, and evidence suggests minimal direct effects on CB_1_R and CB_2_R [3,4]. The metabolic pathways of Δ^9^-THC and CBD have been identified [5]. Δ^9^-THC is initially metabolized to 11-hydroxy-Δ^9^-THC, which retains activity at CB_1_R; it is not until 11-hydroxy-Δ^9^-THC is further oxidized into the carboxylic acid metabolite that it loses CB_1_ activity [6]. Similarly, CBD is principally hydroxylated to 7-hydroxy-CBD, which, in preclinical models of seizures, remains active, though the precise mechanism of action is unknown [7]. Further metabolism to the carboxylic acid 7-carboxy-CBD arrests its activity [7,8]. However, our group has also proven that CBD can be directly glucuronidated (CBD-gluc) [9], and the activity of CBD-gluc is unknown.

**Figure 1:**
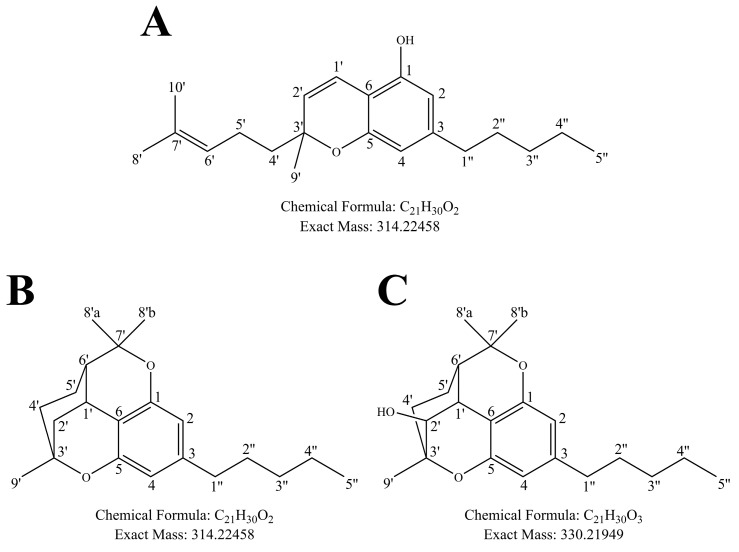
Structures and numbering of (**A**) CBC, (**B**) cannabicitran (CBT-C), and the main oxidized CBC metabolite identified in the present study, (**C**) 2′-hydroxycannabicitran. In this figure and throughout the present manuscript, CBC, CBT-C, and 2′-hydroxycannabicitran are numbered according to a terpenoid system of numbering [36,37]. The theoretical exact masses and chemical formulas are shown below each respective structure.

Besides Δ^9^-THC and CBD, interest in minor phytocannabinoids has intensified, since many of these are believed to lack the psychoactive properties that are primarily mediated through CB_1_R activation; one such minor cannabinoid is cannabichromene (CBC, Figure 1A). CBC represents a small percentage of whole plant material (0.05–0.3%); however, these are relatively old metrics obtained from samples collected from 1993–2014 [10,11,12,13]. More recently, as a result of the passage of the Agricultural Improvement Act (aka Farm Bill) in 2018, the general public in the United States has unrestricted access to highly purified and concentrated minor cannabinoids, including CBC [14]. In 2023, the United States minor cannabinoid market size was estimated at 11.5 billion dollars, but is expected to increase to 33.3 billion dollars by 2030 in part due to far-reaching medicinal claims including treatment for neurological disorders, cancer, inflammation, and pain management [15]. These claims have been drastically understudied; preliminary reports on CBC activity have shown conflicting results. CBC has previously been shown to be a partial agonist of CB_2_R but not CB_1_R [16], while other studies have confirmed partial agonism in CB_2_R and also shown partial agonism in CB_1_R [17]. CBC is generally thought to be non-psychoactive in animal models [18,19]. In exploring non-CB receptor systems, CBC was shown to interact with a variety of transient receptor potential (TRP) channels including TRPA1 [20,21,22,23], TRPV1–4, and TRPV8, thereby having implications for pain and inflammation [2,24], and acute respiratory distress syndrome [25]. Another study showed the anti-inflammatory effects of CBC through a non-CB receptor mechanism in a model of edema [26]. A potential anti-inflammatory mechanism of action was described through downregulating the mitogen-activated protein kinase (MAPK) and nuclear factor kappa B (NF-κB) pathways [27]. CBC has been tested in several model systems of cancer, including urothelial cell carcinoma [28], human breast carcinoma [29], neuroblastoma [30], and bladder cancer [31] with varying efficacy. Due to the wide variety of potential therapeutic applications and potential toxicities, it is important to characterize the pharmacokinetics of CBC and the potential activity of the resulting metabolites.

Previous studies of CBC metabolism applied various animal models resulting in differing metabolic profiles [32,33]. Using gas chromatography–mass spectrometry, Harvey and Brown described hydroxylation in all positions of both aliphatic chains of CBC in rabbit-derived liver microsomes [33]. In hamster-, gerbil-, and cat-derived liver microsomes, epoxidation was detected across the alkene on the branched aliphatic chain representing 10, 12, and 42% of the total amount of metabolites generated in each respective animal model [34]. A recently published study identified four CBC metabolites generated by human liver microsomes: 1″-hydroxy-CBC; 8′-hydroxy-CBC; 6′, 7′-epoxy-CBC; and 6′, 7′-dihydroxy-CBC [35]. However, the authors looked specifically for previously synthesized metabolites and no attempt was made to detect and identify other potentially novel CBC metabolites. This contributes to uncertainty as to whether the major metabolites of CBC were captured.

**Figure 2:**
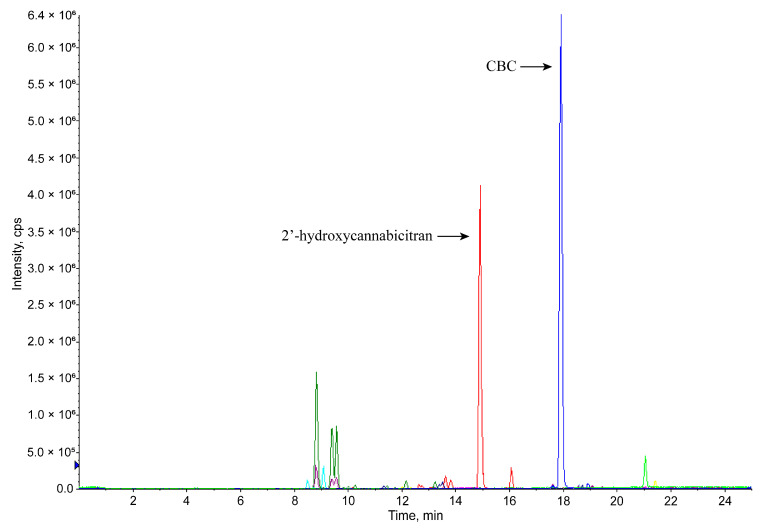
Upscaled metabolite generation following CBC incubation with HLMs analyzed via LC-MS/MS. The figure shows overlaid extracted ion chromatograms recorded on a Sciex API4000 MS/MS system (Supplementary Materials Section S1.2). The blue tracer represents CBC ([M+H]^+^, *m*/*z* = 315.5, primary peak retention time at 17.9 min), the red tracer represents +16 Da from CBC ([M+H]^+^, *m*/*z* = 331.5, indicating addition of one oxygen, primary peak retention time at 14.9 min), and the dark green tracer represents +32 Da from CBC ([M+H]^+^, *m*/*z* = 347.5 indicating addition of two oxygens, primary peak retention time at 8.8 min). The neon green peak at 21.0 min was present in the background controls and is not considered a metabolite. Additional extracted ion chromatogram tracers and analytical details are available in the Supplementary Materials (Section S1.2). As shown, HLMs generated one major metabolite, which we later identified to be 2′-hydroxycannabicitran. Based on this result, we decided to focus here on said major metabolite, its structural identification, and its potential interaction with the CB_1_ and CB_2_ receptors. No attempt was made during the present study to structurally identify any of the other minor metabolites.

In continuation of the aforementioned published CBC drug metabolism studies, we found that incubation of CBC with pooled human liver microsomes (HLMs) resulted in one major, yet unknown oxidized metabolite (Figure 2). Our aim was to identify the structure of said major CBC metabolite. We applied an array of analytical techniques including gas chromatography–tandem mass spectrometry (GC-MS/MS), high-resolution time-of-flight (TOF) tandem mass spectrometry, and nuclear magnetic resonance (NMR) spectroscopy to elucidate this metabolite’s structure. Once the structure was identified, we employed in silico molecular modeling and in vitro competitive binding assays to determine the binding of said major CBC metabolite to CB_1_R and CB_2_R. A diagram of the experimental workflow is available in the Supplementary Materials (Section S1.1).

## 2. Materials and Methods

### 2.1. Enzymes, Reagents, and Chemicals

HPLC-grade water, methanol, acetonitrile, and 88% certified ACS-grade formic acid were purchased from Thermo Fisher Scientific (Waltham, MA, USA). Chloroform-d_1_ (CDCl_3_, 99.8 atom%D, Thermo Fisher Scientific, Waltham, MA, USA), D_2_O (99.9 atom%D, Thermo Fisher Scientific, Waltham, MA, USA), and acetonitrile-d_3_ (ACN-d_3_, 99.96 atom%D, Sigma-Millipore, St. Louis, MO, USA) were used as NMR solvents. Hydrogen peroxide solution (30% *w*/*w* in water containing stabilizer) was used for synthetic generation of the major metabolite of CBC (Sigma-Millipore, St. Louis, MO, USA). CBC used in incubations with HLMs and NMR was ≥98% pure purchased from Cayman Chemical (Ann Arbor, MI, USA), and CBC used in synthesis of 2′-hydroxycannabicitran was >98% pure purchased from Open Book Extracts (Roxboro, NC, USA). The internal standard CBC-d_9_ for LC-MS/MS analyses was a certified reference material purchased from Cayman Chemical (Ann Arbor, MI, USA). HLMs had been characterized for individual cytochrome P450 activity and concentration by the manufacturer (Xenotech, Kansas City, KS, USA); a pool of 50 individuals was used for the kinetic studies (H0620/Lot #1810003), while a pool of 200 individuals was used for upscaled incubations and isolation of the major oxidized CBC metabolite (H2640/Lot #1910096). Dichloromethane (ACS-grade, VWR International, Radnor, PA, USA) was used for metabolite extraction from HLMs. The following reagents were purchased from Sigma-Millipore (St. Louis, MO, USA): Na^+^/K^+^-phosphate buffer, magnesium chloride (MgCl_2_), ethylenediaminetetraacetic acid (EDTA), β-nicotinamide adenine dinucleotide phosphate hydrate (NADP), isocitric acid, and isocitric dehydrogenase were used for the HLM NADPH-generating system. Reagents utilized for derivatization prior to GC-MS/MS analysis included ethyl acetate (anhydrous 99.8%) and N,O-bis(trimethylsilyl)trifluoroacetamide with 1% chlorotrimethylsilane (BSTFA with 1% TMCS analytical standard). Reagents used for competitive CB_1_R and CB_2_R binding assays included WIN 55,212, CP 55,940, Δ^9^-THC, CBC, and cannabicitran (CBT-C), all purchased from Cayman Chemical (Ann Arbor, MI, USA). The radioactive ligand [^3^H]CP 55,940 was purchased from PerkinElmer (Waltham, MA, USA). Buffer components and polyethylenimine were purchased from Sigma-Millipore (St. Louis, MO, USA).

### 2.2. Generation of CBC Metabolites with Pooled Human Liver Microsomes

To maximize the yield of CBC metabolites, parameters including incubation time, HLM protein concentration, and CBC concentration were optimized, and representative extracted ion chromatograms are available in the Supplementary Materials (Section S1.2). The parameters resulting in the highest yield of metabolites were determined to be 0.5 mg/mL HLM, 50 µg/mL CBC, and a 40 min incubation time. Prior to adding the drug, the NADPH-generating system was incubated on a shaker set to 150 rpm at a temperature of 37 ± 2 °C for 10 min (C24 Incubator Shaker, New Brunswick Scientific, Edison, NJ, USA). Concentrations of buffer constituents were Na^+^/K^+^-phosphate buffer (0.1 M, pH 7.4), containing MgCl_2_ (3.0 mM), EDTA (1.0 mM), NADP (1.0 mM), isocitric acid (5.0 mM), and isocitric dehydrogenase (1 Unit/mL). The final volume of the reaction was 100 mL. After 40 min, the reaction was quenched with a 1:1 volume of ice-cold acetonitrile and the slurry was transferred to 50 mL conical tubes and centrifuged at 685× *g*, at 4 °C for 10 min. Following protein precipitation and centrifugation, the supernatants were combined into a 1 L separatory glass funnel and extracted using dichloromethane. The higher-density organic phase was retained and dried under a flow of nitrogen at room temperature. Once dry, the sample was reconstituted in 1.5 mL of pure acetonitrile, transferred into an amber glass HPLC vial and immediately isolated using semi-preparative high-performance liquid chromatography–diode array detection (HPLC-DAD). The resulting fractions were stored at −80 °C until further analysis.

The HPLC components for the semi-preparative isolation of the major CBC metabolite were as follows: G1322A degasser, G1312A binary pump, G1329B 1260 ALS, G1315B DAD, G1364B fraction collector (all Agilent Technologies, Santa Clara, CA, USA), and an external column compartment ThermaSphere (Phenomenex, Torrance, CA, USA). The HPLC-DAD system was controlled, and data were processed using ChemStation software revision 04.03.087 (Agilent Technologies, Santa Clara, CA, USA). The semi-preparative columns were four Eclipse XDB-C8, 5 µm, 9.4 × 250 mm, connected in series (Agilent Technologies, Santa Clara, CA, USA). The flow rate was 3.5 mL/min, and the mobile phases were HPLC-grade water (mobile phase A) and HPLC-grade acetonitrile (mobile phase B). The elution gradient for isolating CBC metabolites was 0.0 min, 40% B; 22.0 min, 52% B; 48.0 min, 75% B; 50.0 min, 96% B; 54.0 min, 99% B; 69.0 min, 99% B; 70.0 min, 40% B; and 84.0 min, 40% B. The injection volume was 100 µL. UV absorbance was monitored at 210 nm and 231 nm. Fractions were collected based on a UV signal. Additional details are available in the Supplementary Materials (Section S1.3). Fractions were dried under nitrogen flow at room temperature and reconstituted in 1.5 mL of pure acetonitrile, which was aliquoted into 3 vials. Isolated metabolites and controls were analyzed utilizing both LC-MS/MS and GC-MS/MS as described below. Samples were stored in a −80 °C freezer until analysis.

### 2.3. Derivatization of CBC Metabolite and GC-MS/MS Analysis

Samples were derivatized and analyzed using GC-MS/MS based on the previous literature [32,33]. Briefly, 100 µL of sample was dried under nitrogen flow, and derivatized with 50 µL of ethyl acetate and 50 µL of BSTFA 1% TMCS. The vial was heated at 70 °C (Isotemp Hot Plate Stirrer, Thermo Fisher Scientific, Waltham, MA, USA, 150 rpm) for 60 min [38]. Samples were cooled, transferred to autosampler vials and immediately analyzed via GC-MS/MS.

The system consisted of an AOC-6000 autosampler, a GC-2010 Plus, and a GCMS-TQ8050 (all Shimadzu Corporation, Kyoto, Japan). The column was a DB-5MS, 30 m, 0.25 mm × 0.25 µm (Agilent Technologies, Santa Clara, CA, USA). The column temperature gradient was 50.0 °C ramped to 230.0 °C at 25 °C/min, 230.0 °C ramped to 258.0 °C at 5 °C/min, and 285.0 °C ramped to 300.0 °C at 10.0 °C/min. The total program runtime was 25.7 min. The injection temperature was 265.0 °C. The carrier gas was helium with a constant flow rate of 1.3 mL/min. A 1 µL sample was injected in split mode with a split ratio of 1:5. Further GC parameters are available in the Supplementary Materials (Section S1.5).

The mass spectrometer was operated with an electron impact ionization source at 70 eV. Spectra were acquired in scan mode from *m*/*z* = 50.00 to 650.00 with a scan speed of 2500 ms, a start time of 3.0 min, and an end time of 25.7 min. The ion source temperature was 250 °C and the interface temperature was 280 °C with a solvent cut time of 2 min. The GC-MS/MS system was controlled, and data were processed using GCMSsolution software (version 4.5, Shimadzu Corporation, Kyoto, Japan).

### 2.4. Chemical Synthesis of the Major Metabolite of CBC

Following HLM incubation, metabolite isolation, and preliminary structural identification hypothesizing an epoxide, the major CBC metabolite was produced by chemical synthesis. For this, 0.048 mmol CBC was incubated with 12.89 mmol hydrogen peroxide at 60 °C, then placed at −20 °C until cool. Isolation of generated products was immediately performed via semi-preparative HPLC-DAD.

The synthetically generated major oxidized CBC metabolite was isolated based on retention time using the same semi-preparative HPLC-DAD system described above including equipment, mobile phases, columns, flow rate, injection volume, and UV detection. The elution gradient was 0.0 min, 80% B; 10.0 min, 80% B; 30.0 min, 99% B; 34.0 min 99% B; 34.1 min, 80% B; and 46.0 min, 80% B. Representative chromatograms of the collected fractions are available in the Supplementary Materials (Section S1.4).

### 2.5. Identification of the Major Metabolite Structure via NMR Analysis

The purity of the isolated major metabolite was confirmed via HPLC-UV-MS/TOF (Supplementary Materials Section S3.1). The sample was dried under nitrogen flow and reconstituted in 750 µL of acetonitrile-d_3_ or chloroform-d_1_ as appropriate and transferred to a 5 mm NMR tube rated for a frequency of 600 MHz (Norell, Morganton, NC, USA).

NMR spectroscopy was performed using a Bruker Avance Neo spectrometer operating at 600 MHz ^1^H equipped with a Bruker TCI ^1^H&^19^F/^13^C/^15^N 5 mm helium cooled cryoprobe (Bruker, Billerica, MA, USA). All NMR experiments were performed at 25 °C using the Bruker pulse sequences provided in TopSpin version 4.3.2. Chemical shifts were referenced to the residual solvent peak: acetonitrile at 1.94 ppm in ^1^H and at 118.26 ppm in the ^13^C dimension and chloroform-d_1_ at 7.26 ppm in the ^1^H dimension. Experiments included ^1^H, ^13^C, ^13^C Distortionless Enhancement by Polarization Transfer (DEPT-135), ^1^H-^13^C Heteronuclear Single Quantum Coherence (HSQC), ^1^H-^13^C Heteronuclear Multiple Bond Correlation (HMBC) experiments, ^1^H-^1^H gradient selected Double Quantum Filtered-Homonuclear Correlation Spectroscopy (DQF-COSY), and Nuclear Overhauser Effect Spectroscopy (NOESY). NMR data were transferred and processed utilizing TopSpin. Further experimental parameters are available in the Supplementary Materials (Sections S3.2 and S3.3).

### 2.6. Molecular Docking

The Schrödinger Suite (release 2023-3, Schrödinger, New York, NY, USA) was used for all ligand and protein preparation, and the Glide Module for all docking calculations [39,40,41]. Using LigPrep, the following compounds were prepared at physiological pH: (−)-Δ^9^-THC, (+)-CBC, (−)-CBC, (R)-2′-hydroxy-(+)-cannabicitran, (S)-2′-hydroxy-(−)-cannabicitran, (+)-CBT-C, and (−)-CBT-C (structures are available in the Supplementary Materials Section S4.1). CB_1_R and CB_2_R were imported from Protein Data Bank (PDB code: 5XR8) and (PDB code: 5ZTY), respectively [42,43]. These protein structures were prepared by removing all water molecules and co-crystallized ligands, and then minimized with OPLS4 force fields and the VSGB solvation model [44]. CB_1_R and CB_2_R were scanned and mapped to confirm their binding site. Computational grids were then formed around the mapped binding sites to have the test compounds docked into. This docking was completed with Standard-Precision (SP) and Extra-Precision (XP) glide models for comparison. Top-ranked ligand–protein conformations were produced with corresponding scoring function values.

### 2.7. Competitive Binding Assays

Radioligand binding to cannabinoid receptors (CB_1_R and CB_2_R) was conducted as previously described [45]. Human embryonic kidney 293 (HEK) cells were transfected using lipofectamine 2000 (Invitrogen, Waltham, MA, USA) using CB_1_R or CB_2_R cDNA in pcDNA3.1+ plasmid (cDNA Resource Center, Bloomsburg, PA, USA), and stable cell lines were established. HEK-CB_1_ and HEK-CB_2_ cells were grown in DMEM supplemented with 10% fetal bovine serum (HyClone Laboratories, Logan, UT, USA), 1% penicillin/streptomycin (final concentration 100 units/mL or 100 µg/mL, respectively), and 300 µg/mL G418. Cells were grown to confluency on 15 cm dishes. Cells from 2 plates were scraped into approximately 7 mL calcium- and magnesium-free phosphate-buffered saline (CMF-PBS) containing 1:3000 protease inhibitor cocktail III (PI) (EMD Millipore, Burlington, MA, USA) and centrifuged at 11,000× *g* at 4 °C for 10 min. The supernatant was removed, and the cell pellet was resuspended in 2 mL of hypotonic buffer (5 mM Tris, 2 mM EDTA + PI) using a polytron. The homogenate was centrifuged at 35,000× *g* for 20 min, resuspended in TME buffer (20 mM Tris, 5 mM MgCl_2_, 1 mM EDTA, pH 7.4 at 4 °C) with PI using a polytron, and an additional 5 mL of hypotonic buffer was added. The homogenate was centrifuged a second time at 35,000× *g* for 20 min, the supernatant was removed, and the final membrane pellet was covered with 2 mL TME buffer and stored at −80 °C until needed. 

The binding assay was performed in duplicate in a 96-well plate using TME supplemented with 5 mg bovine serum albumin (BSA)/mL, pH 7.4 at 30 °C. Total binding and nonspecific binding was determined in duplicate for each concentration of radioligand. The reaction mixture included test compounds Δ^9^-THC, WIN 55,212 or CP 55,940, membrane preparation (40–50 mg protein), [^3^H]CP 55,940, and TME+BSA buffer in a final volume of 0.5 mL. The concentration of [^3^H]CP 55,940 was 1.4 nM. After incubation at 30 °C for 60 min, the reaction was terminated by filtration with TME + 1 mg BSA/mL over Filtermat A filters (Revvity, Boston, MA, USA) presoaked in 0.2% polyethylenimine. An additional wash was added to the normal harvesting program to reduce nonspecific binding. The filters were dried at room temperature, spotted with scintillation cocktail, and remaining radioactivity was determined utilizing a microBetaplate 1405 scintillation counter (PerkinElmer, Waltham, MA, USA).

Full characterization of compounds included the generation of sigmoidal curves for the determination of IC_50_ values and Hill coefficients for the displacement of [^3^H]CP 55,940 using a nonlinear curve-fitting algorithm (GraphPad PRISM, version 10.1.1, GraphPad Software, Boston, MA, USA). K_i_ values were calculated using the Cheng–Prusoff transformation (Equation (1)):

(1) $$K_{i}=\frac{{IC}_{50}}{1+L/K_{d}}$$

where *L* is the radioligand concentration and *K_d_* is the binding affinity of the radioligand, as determined by saturation binding analysis. For [^3^H]CP 55,940, the *K_d_* value is 1.54 ± 0.28 nM and 1.18 ± 0.16 nM, and the density of receptors is 990 ± 120 and 8100 ± 1100 fmol/mg protein for CB_1_ and CB_2_ receptors, respectively. If applicable, outliers were removed based on the results of the Grubbs’ test.

### 2.8. Data Analysis

Chemical structures were drawn using ChemDraw (version 19.0, PerkinElmer, Waltham, MA, USA). Please refer to the subsections above for specific software used for controlling instruments, to interpret spectra and data at various points in this study.

## 3. Results

### 3.1. Metabolism of CBC by Human Liver Microsomes

Once incubation parameters yielding the most metabolites were determined ( Supplemental Materials S1.2), the reaction was scaled up to generate sufficient metabolites to obtain a comprehensive overview of the pattern of CBC metabolites generated by oxidative metabolism (+16 Da and +32 Da). A representative extracted ion chromatogram after incubation of CBC with HLM using the optimized conditions is shown in Figure 2. The majority of metabolites generated showed a +16 Da mass shift compared to CBC, which corresponds to hydroxylation or epoxidation. Among those, HLMs generated one major CBC metabolite. Said metabolite was isolated by semi-preparative HPLC-DAD for further structural elucidation. Harvey and Brown previously successfully employed GC-MS to identify the structures of several CBC metabolites [32,33]. Therefore, samples were derivatized and analyzed using GC-MS/MS with the goal to detect fragments that provided additional information aiding in the structural identification of the major oxidized CBC metabolite.

### 3.2. Identification of the Structure of the Major CBC Metabolite Generated by Human Liver Microsomes Using GC-MS/MS

**Figure 3:**
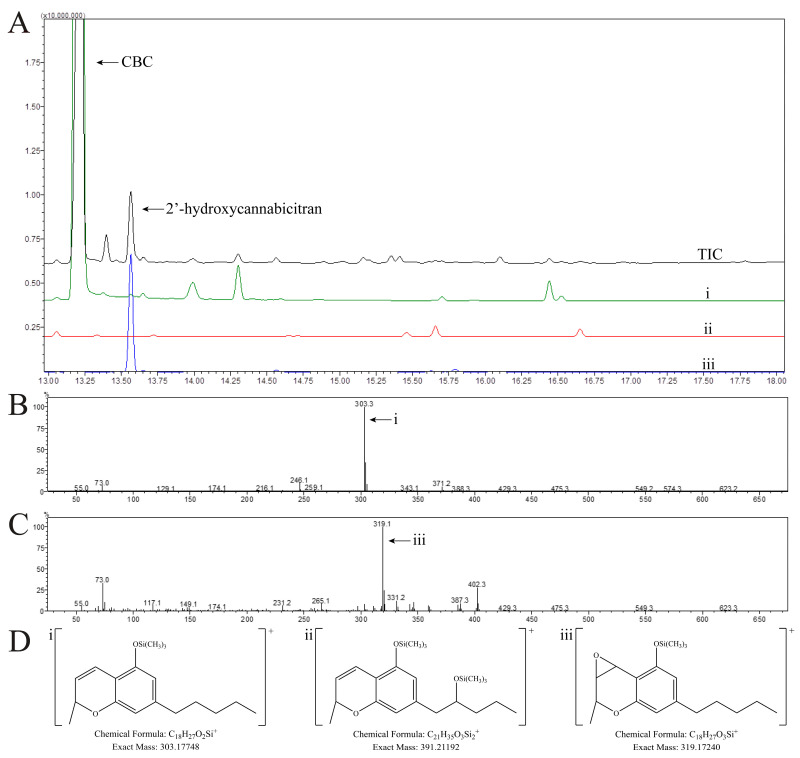
(**A**) Staggered overlay of GC-MS/MS total ion chromatogram (TIC, black tracer) of CBC incubated with HLMs, extracted ion chromatogram of the fragment *m*/*z* = 303.2 ((**i**), green tracer), extracted ion chromatogram of the fragment *m*/*z* = 391.2 ((**ii**), red tracer), and extracted ion chromatogram of the fragment *m*/*z* = 319.1 ((**iii**), blue tracer). Extracted fragments were selected based upon expected major fragments of metabolites following the formation of the chromenyl ion. Intensities of the extracted fragments were multiplied by a factor of 10 for ease of visibility. Major fragments are shown due to the considerable fragmentation of the molecular ion following ionization, as expected from previous reports [32,33]. Mass spectra of CBC and the major oxidized metabolite 2′-hydroxycannabicitran are shown in (**B**,**C**), respectively. Proposed structures for possible chromenyl ions are shown in (**D**(**i**–**iii**)). Importantly, these structures are representative possibilities and an identical modification elsewhere on the molecule resulting in the same chemical formula would be correct.

In GC-MS/MS, a fundamental fragment of CBC is the formation of the chromenyl ion, previously described by Harvey and Brown (please see Supplemental Materials S1.5) [32,33]. This ion was limited in its diagnostic capabilities as it could only inform which half of the CBC molecule the modification may be on (Figure 3D). CBC eluted with a retention time of 13.20 min (Figure 3A). The fragmentation pattern of CBC closely matched that of previous reports (Figure 3B) [32,33]. The major fragment was the chromenyl ion without any additional substitutions with a *m*/*z* = 303.3 (Figure 3D(i)). A fragment with *m*/*z* = 371.2 is the product of demethylation of the M^+**·**^ ion. Other fragments corresponding with the previous literature include *m*/*z* = 304.3, 305.3, and 246.1.

The major metabolite of CBC (Figure 1C) eluted at 13.57 min (Figure 3A). This peak coeluted with a peak in a control sample; however, the fragmentation was considerably different and is available in the Supplementary Materials (Section S1.5). The major fragment was *m*/*z* = 319.1 (Figure 3C). The mass difference of +16 Da to the core chromenyl ion was indicative of an addition of oxygen that was protected from derivatization (Figure 3D(iii)). Other fragments seen were supportive of this hypothesis such as the M^+**·**^ ion with *m*/*z =* 402.3 and the demethylation product of *m*/*z* = 387.3. Based on these ions, we hypothesized an epoxide at the benzylic carbon on the 2H-pyran ring of CBC. From the upscaled incubation with HLMs, the isolated fraction corresponding to this peak was both derivatized and run on GC-MS/MS and LC-MS/MS systems to confirm the retention time and peak assignment (Figure 2). Similar to LC-MS/TOF, the GC-MS/MS method could not offer any absolute confirmatory structural information due to the lack of sufficiently unique MS/MS fragment ions. Therefore, we employed NMR spectroscopy to completely identify the structure of the major metabolite with necessary certainty.

To provide sufficient material for NMR analysis (>1 mg), based on our hypothesis of an epoxidation on the 2H-pyran ring of CBC, we sought to generate the major metabolite using synthesis via incubation with hydrogen peroxide. As expected, several products were created during this synthesis, which could be chromatographically separated and isolated via semi-preparative HPLC-DAD (Supplementary Materials Section S1.4). Moreover, a comprehensive comparison of the synthetically and biologically generated metabolite is shown in the Supplemental Materials Sections S1.5 and S2.1. Briefly, the synthetically generated metabolite matched the chromatographic retention time, MS/MS fragmentation patterns including high-resolution mass of the fragments, and relative fragment intensities when analyzed on both GC-MS/MS and HPLC-MS/TOF. Multiple batches of the synthetically generated major metabolite were required to provide sufficient quantity and purity for NMR analysis. Purity was established by HPLC-UV-MS/TOF, whereby the MS was run in the scan mode (Supplementary Materials Section S3.1).

### 3.3. Structural Identification of 2′-Hydroxycannabicitran via NMR

First, we analyzed CBC to establish a baseline for determining the metabolite structure. Several NMR experiments were conducted with CBC in ACN-d_3_ including ^1^H-NMR, ^13^C-NMR, HSQC, HMBC, and DQF-COSY (for more details, please see the Supplementary Materials Section S3.2). The NMR peak assignments of CBC in ACN-d_3_ needed to be established as the isolated major CBC metabolite proved not to be stable in CDCl_3_, but inACN-d_3_ (vide infra). A summary of assigned proton and carbon chemical shifts is shown in Table 1. As a reference, CBC was analyzed in CDCl_3_ as well as ACN-d_3_ to allow for a direct comparison to the previous literature [46]. The corresponding spectra are available in the Supplementary Materials (Section S3.2). Briefly, our ^1^H-NMR spectrum collected in CDCl_3_ matched that of a previously published report with respect to multiplicity and chemical shift [46]. Our assignments of CBC in ACN-d_3_ were similar to measurements made in CDCl_3_ with some differences corresponding to the chemical shift of the protons. The only significant discrepancy was the chemical shift of the hydroxyl proton, which was 4.561 ppm in CDCl_3_ and 6.814 ppm in ACN-d_3_.

We synthesized 2.23 mg/mL of 100% pure compound that was identical to the major metabolite of CBC generated by human HLM. Quantity was determined by HPLC-UV-MS/TOF and an NMR-based calibration curve (Supplementary Materials Section S3.1).

**Figure 4:**
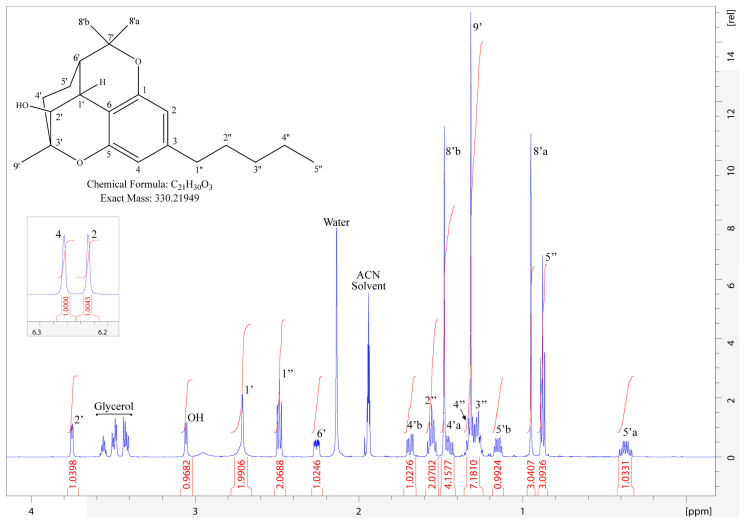
^1^H NMR of 2′-hydroxycannabicitran. Solvent peaks and impurities are labeled accordingly. There was poor resolution between H-8′b and H-4′a as well as H-4″, H-9′, and H-3″; therefore, all subsets were integrated as a group.

We observed metabolite reactivity with CDCl_3_ (Supplementary Materials Section S3.1); therefore, ACN-d_3_ was selected as the NMR solvent. Based on the interpretation of the NMR peak assignments of the purified major oxidized metabolite (Table 2), the structure was identified as 2′-hydroxycannabicitran. Several experiments were conducted to confirm the given assignments such as HSQC, HMBC, DEPT-135, DQF-COSY, and NOESY experiments (Supplemental Materials S3.3). The major metabolite displayed complex coupling patterns in the ^1^H spectrum (Figure 4) and differed greatly compared to CBC (please compare to Table 1), indicating limited structural similarity.

Evidence for the complex ring structure of 2′-hydroxycannabicitran came from the observation of multiple new short- and long-range correlations. In particular, the proton at position 2, while previously only seeing correlations within the ring at position 1, now showed correlations to multiple spin systems including three-bond *J*-couplings in the DQF-COSY to H-2′ and H-6′. When analyzing C-6′, HMBC correlations in CBC showed the terminal methyl groups as well as adjacent CH_2_ groups. H-6′ of the metabolite retained a long-range correlation to one of the terminal methyl groups, but no longer had correlations to the previous chain. Instead, it showed a correlation reaching to the aromatic ring (C-6). It also displayed a correlation to C-1′ and C-2′, a correlation that would be too great of a bond distance to establish an HMBC correlation assuming identical bond arrangement to CBC. Another correlation was the cross-peak between C-7′ and H-1′. Similarly, the methyl protons labeled 8′b showed a long-range correlation in the HMBC to the preexisting ring C-1. All these interactions were evidence of the formation of a new heterocyclic ring. NMR spectra from 2′-hydroxycannabicitran are available in the Supplementary Materials (Section S3.3).

Another key differentiator between CBC and 2′-hydroxycannabicitran was the chemical shift in the proton corresponding to the hydroxylation. CBC has a hydroxylation on C-1 and the proton had a ^1^H chemical shift of 6.814 ppm as a result of its proximity to the aromatic ring. 2′-Hydroxycannabicitran no longer displayed said hydroxyl hydrogen adjacent to the aromatic ring. However, it did contain a hydroxyl proton, as evidenced by long-range correlations (C-3′, C-2′, and C-1′) observed in the HMBC experiment originating from a ^1^H that was not directly bonded to ^13^C as evidenced in the HSQC. Additionally, multiple *J*-coupling correlations to other protons in the ring structure of the metabolite were observed in DQF-COSY experiments. In the major metabolite, the ^1^H chemical shift corresponding to the hydroxyl proton, was a doublet at 3.060 ppm. In the NOESY experiment, this proton exhibited the effects of chemical exchange with residual water, and NOE cross-peaks were the opposite sign to pure NOE cross-peaks between other protons in the metabolite.

The tetracyclic ring system was further verified by protons on C-4′ and C-5′ being split in the ^1^H spectra, with protons on C-5′ appearing as multiplets at 1.152 ppm (H-5′b) and 0.373 ppm (H-5′a). Long range coupling on HMBC displayed differing correlations with the exception of a correlation to both protons at the C-6′ position representing a two-bond correlation. This feature has been described previously in a study analyzing extracted cannabicitran (CBT-C, Figure 1B) [47]. Due to the high structural similarity between the major metabolite of CBC and CBT-C, we analyzed the ^1^H- and HSQC NMR spectra of CBT-C in ACN-d_3_ and compared the results to 2′-hydroxycannabicitran, where the primary difference is the presence of a hydroxylation at the 2′ position (Supplementary Materials Section S3.4). Taken together, these results showed that the major metabolite of CBC generated by HLMs using the incubation conditions described above was 2′-hydroxycannabicitran.

### 3.4. Molecular Docking Prediction of CB_1_ and CB_2_ Receptor Binding

After structural identification, the next step was to determine if the metabolite binds to CB_1_ and CB_2_ receptors and to compare its binding to that of CBC. Simulations were performed comparing the binding of Δ^9^-THC, CBC, 2′-hydroxycannabicitran, and CBT-C to both CB_1_R and CB_2_R. Importantly, since natural and synthetic CBC exists as a racemic mixture of enantiomers [23,37,48,49,50], we considered the stereospecificity of the ligands to the orthosteric site and modeled (−)-Δ^9^-THC, (+)-CBC, (−)-CBC, (R)-2′-hydroxy-(+)-cannabicitran, (S)-2′-hydroxy-(−)-cannabicitran, (+)-CBT-C, and (−)-CBT-C (structures are available in the Supplementary Materials Section S4.1). We utilized both Standard-Precision (SP) and Extra-Precision (XP) glide models to rank all ligands according to their docking scores. The SP model attempts to minimize false negatives by being a more forgiving function that identifies ligands with a reasonable disposition to bind. Contrastingly, the XP model attempts to minimize false positives by being a strict function exacting penalties when violations of established physical chemistry principles are predicted [39]. The SP model rankings and highlighted residue interactions for CB_1_R and CB_2_R are shown in Table 3 and Table 4, respectively. XP model rankings and figures of all ligands in the active site of CB_1_R and CB_2_R are available in the Supplementary Materials (Sections S4.2 and S4.3, respectively).

Interestingly, (+)-CBC was ranked the highest with a docking score of −10.600 at CB_1_R (Table 3). The aromatic ring and pyran-type ring each formed π–π stacking with Phe170 and Phe268. In this simulation, a hydrogen bond was formed between the hydrogen of the phenolic hydroxyl moiety and the oxygen of Ser505, providing further stability of CBC in the orthosteric site of CB_1_R. (−)-Δ^9^-THC displayed similar binding to the CB_1_R, specifically π–π interactions between the aromatic ring and Phe170 and Phe268, which corresponds to the previous literature [42]. The hydroxyl group on (−)-Δ^9^-THC had a hydrogen bond to Ser505 with a slightly longer predicted distance of 1.84 Å compared to the analogous interaction with (+)-CBC, which measured at 1.69 Å. This slight increase in hydrogen bond distance and additional π–π stacking justifies (+)-CBC having a higher docking ranking than (−)-Δ^9^-THC. (−)-CBC was the third-ranking ligand and showed similar interactions to (+)-CBC with the exception of one additional π–π interaction between the aromatic ring and Phe174. This residue has been previously described as interacting with (−)-Δ^9^-THC [51]. The major metabolite of CBC, (R)-2′-hydroxy-(+)-cannabicitran, and (S)-2′-hydroxy-(−)-cannabicitran did not show particularly strong docking to CB_1_R. (R)-2′-hydroxy-(+)-cannabicitran predicted a hydrogen bond from the added hydroxylation to the δ-carboxylic acid of Ile267. (S)-2′-hydroxy-(−)-cannabicitran was the lowest ranked of the ligands examined, only showing a single π–π interaction from the aromatic ring to Phe170. Both stereoisomers of CBT-C displayed low binding affinity with π–π interactions.

Overall, there were fewer interactions between the ligands and the active site of the CB_2_R (Table 4). Again, (+)-CBC was ranked the highest, showing π–π interactions through the pyran-type ring to Phe87 and Phe183. Interestingly, (+)-CBT-C had the second highest docking score, though no registered interactions. (+)-CBT-C docked deeper in the orthosteric pocket of CB_2_R compared to (−)-Δ^9^-THC, which may contribute to its docking score through potential van der Waals forces between the tetracyclic ring system and pi groups of the abundant aromatic residues in the orthosteric site. (−)-Δ^9^-THC also interacted with the same phenylalanine residues as (+)-CBC, but instead through the aromatic ring, and sat with the tricyclic ring system towards the N-terminus and alkyl chain residing towards the middle of the receptor. (S)-2′-hydroxy-(−)-cannabicitran was ranked fourth in the SP precision model with no noted interactions. (R)-2′-hydroxy-(+)-cannabicitran docked nearly in a mirrored image when compared to the enantiomer, which may indicate selectivity for one isomer over the other. In the XP model the (S)-2′-hydroxy-(−)-cannabicitran was ranked first without highlighted interactions, where the (R)-2′-hydroxy-(+)-cannabicitran was not ranked. (−)-Δ^9^-THC was ranked second of two ligands ranked in the XP model displaying a singular π–π interaction between the aromatic ring and Phe183 (Supplementary Materials Section S4.3).

### 3.5. In Vitro Interactions at the CB_1_ and CB_2_ Receptors 

**Figure 5:**
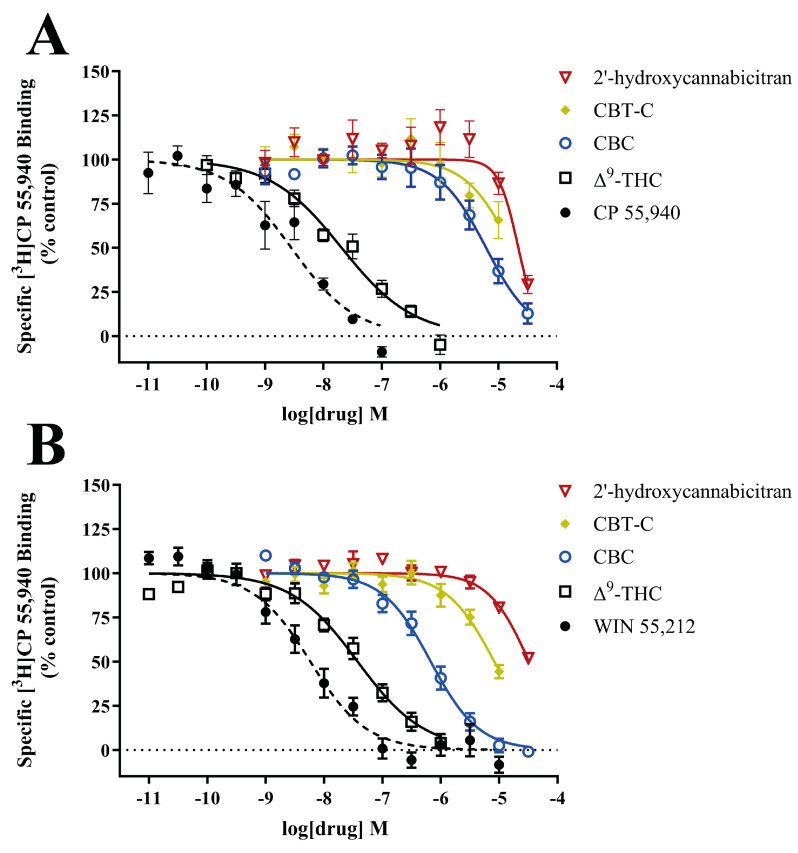
Concentration–response curves of tested ligands in radioligand binding assays at (**A**) CB_1_R and (**B**) CB_2_R. Data shown represent the means ± S.E.M. of four to ten experiments conducted in duplicate.

As the docking simulations indicated that the major metabolite of CBC has the potential to interact with CB_1_ and CB_2_ receptors, we used a highly purified and structurally confirmed metabolite to study its interactions with CB_1_R and CB_2_R in vitro. The displacement of [^3^H]CP 55,940 by CBC, 2′-hydroxycannabicitran, CBT-C, Δ^9^-THC, CP 55,940, and WIN 55,212 from human recombinant CB_1_R (Figure 5A) and CB_2_R (Figure 5B) were examined. The resulting K_i_ and Hill slope values are displayed in Table 5. In alignment with the previous literature, CBC was shown to bind at CB_2_R with moderate affinity (K_i_ 301 ± 72 nM) [16,17]. CBC displayed weak binding affinity to CB_1_R with a K_i_ of 3500 ± 1200 nM. 2′-Hydroxycannabicitran did not retain CBC’s binding affinity at either cannabinoid receptor. CBT-C was able to displace [^3^H]CP 55,940 from CB_2_R at high concentrations corresponding to a K_i_ of 4200 ± 410 nM. Δ^9^-THC was used as a positive control for both receptor assays. CP 55,940 and WIN 55,212 were included as more selective positive controls for CB_1_ and CB_2_ assays, respectively. Positive controls were included in the assays for comparison purposes and displayed high affinity to CB_1_R and CB_2_R where applicable, resembling previously reported values [1,30,45,52].

## 4. Discussion

The metabolic pathways of Δ^9^-THC and CBD have been well established, and both exhibit phase I oxidative metabolism of hydroxylation at the allylic methyl group on the cyclohexene moiety. These metabolites remain biologically active [6,7]. Both then undergo further oxidation and potential phase II conjugation to be eliminated [6,9]. Interestingly, we did not observe any carboxylic acid metabolites for CBC (Supplemental Materials Section S1.2), which seems to be in contrast to Δ^9^-THC metabolism and, to a lesser extent, CBD metabolism.

Previous research conducted in the early 1990s utilized liver microsomes isolated from mouse, rat, guinea pig, rabbit, hamster, gerbil, and cat to determine the metabolic profiles of CBC [32,34]. Harvey and Brown identified various modifications to CBC including eight hydroxylations, one epoxidation, and one dihydroxylation between all species [34]. Of note, the metabolic profile across different species was highly variable in terms of relative abundance of the described metabolites. In their later studies, Harvey and Brown identified structures of eight additional dihydroxylated metabolites in rabbits, adding to the complexity of CBC metabolism in the investigated model organisms [33]. This group did not make an attempt to identify human metabolites of CBC. Based on these initial reports, we sought to utilize comparative and complementary methods to Harvey and Brown and objectively analyze CBC metabolites generated by HLMs.

The first study to characterize the human hepatic biotransformation of CBC was conducted by Havlasek et al. Of note, monoglucuronides represented nearly 50% of all CBC metabolites produced, followed by hydroxylation with 31% [53]. There are several critical caveats to their work. First, the authors reported the use of CBC that contained an impurity described as a structural isomer; therefore, caution must be taken when interpreting the data, particularly when many cannabinoids have identical molecular formulas, such as CBC, CBT-C, Δ^9^-THC, and CBD. Second, the authors’ analytical method confounds chemical species with identical *m*/*z*, such as hydroxylations and epoxidations. While the study was the first to describe human metabolites of CBC, it did not localize the exact oxidation or conjugation positions.

Recently, Roy et al. identified four CBC metabolites stemming from incubation with human liver microsomes [35]. They indicated two principal metabolites, 8′-hydroxy-CBC and 6′, 7′-epoxy-CBC, and two lesser metabolites, 1″-hydroxy-CBC and 6′, 7′-dihydroxy-CBC. Incubation parameters, extraction solvent, and analytical methods differed between their study and the methods reported here, which may have contributed to different identified metabolites. Therefore, we recapitulated the incubation and extraction methodologies of Roy et al. and analyzed the data using our analytical methods and observed identical primary oxidized metabolite profiles between what Roy et al. described and using the methods in the present study (Supplementary Materials Section S2.1). We utilized our verified synthetic standard of 2′-hydroxycannabicitran for comparison to the mixture of CBC incubated with HLMs and confirmed via retention time and MS fragmentation patterns that 2′-hydroxycannabicitran represented the major metabolite using both Roy et al.’s and our incubation and extraction protocols. We were able to confirm the presence of 6′, 7′-epoxy-CBC via a unique α-cleavage fragment (exact mass 71.04914 Da) and supporting fragments (Supplemental Materials Section S2.1). Based on these recapitulation experiments comparing both procedures and workflows, we determined that, most likely, Roy et al. seem to have selected for minor CBC metabolites based on UV signal wherein the double-bond of the 2H-pyran ring remains intact and connected to the aromatic ring. Thus, the major metabolite 2′-hydroxycannabicitran would have been overlooked, despite having been formed with Roy et al.’s incubation and extraction methods (Supplemental Materials Section S2.1). Hence, the results of the present study complement those of Roy et al. [35].

We focused on the major primary oxidized metabolite of CBC due to its predominance in the overlaid extracted ion chromatograms (Figure 2) and total ion chromatogram (Figure 3A). Using the incubation conditions described above, HLMs metabolized CBC to several metabolites with +16 Da and +32 Da, indicating the addition of one or two oxygens, respectively. Among them was said major metabolite with +16 Da that clearly stood out. We have made no attempt yet to structurally identify any of the other metabolites with +16 Da and +32 Da other than those already described by Roy et al. [35] (vide supra). Structural identification of the major CBC metabolite offered several challenges. Previous studies utilizing HLMs to assess oxidative metabolism generally identified metabolites by comparing the MS fragmentation patterns of the parent drug with those of the metabolites, thereby often relying on a combination of ion trap (MS^n^) and high-resolution mass spectrometry [54,55]. However, this strategy was unsuccessful with the major CBC metabolite identified in the present study. Fragments of said major CBC metabolite and CBC were similar or showed a loss of water (−18 Da), which made it impossible to determine the exact location of the metabolic modification. A unique α-cleavage fragment enabled by the addition of an oxygen molecule was not present in the fragmentation pattern of the major oxidative metabolite, which may be the reason that the exact position of modification could not be detected using LC-MS/TOF and MS^n^ (linear Sciex 6500 QTRAP). Therefore, for the identification of the major metabolite of CBC, we employed a combination of GC-MS/MS and ultimately NMR spectroscopy. For the NMR experiments, initially, CBC and its metabolite were dissolved in CDCl_3_ in alignment with the previous cannabinoid literature [46,56]. However, it was observed that CDCl_3_ reacted with the metabolite yielding halogenation of the metabolite, as confirmed by high-resolution TOF analysis. This analysis showed a characteristic chlorine isotopic pattern of two peaks representing ^35^Cl and ^37^Cl with a relative intensity of 3:1 with high mass accuracy (Supplementary Materials Section S3.1). This reactivity provided further evidence for an epoxide intermediate between CBC and 2′-hydroxycannabicitran, leading to our hypothesis that CBC was epoxidized by cytochrome P450 enzymes present in the HLMs. Based on our results, we hypothesize that the initially produced epoxide is labile due to the presence of a nucleophilic double-bond, which in a follow up reaction can attack which results in the opening of the epoxide and, subsequently, forms two new, more stable six-membered ring structures present in 2′-hydroxycannabicitran.

For further confirmation of the metabolite structure, we referred to the detailed analysis of CBT-C in CDCl_3_ conducted by Wood et al. [47]. This group performed both theoretical calculations and determined experimental values for ^1^H and ^13^C assignments of CBT-C extracted from hemp extract. The authors note and explain the unique separation of the protons on C-5′ by quantum mechanical modeling leading to the conclusion the low chemical shift (reported 0.61 ppm, in CDCl_3_) was attributed to shielding stemming from vertical proximity to the phenyl moiety [47]. In the present study, the CBC material used with the incubation of HLMs did not contain any detectable levels of CBT-C, and therefore we are confident that the resulting metabolites were generated from CBC and not residual CBT-C. The verified structure of our newly identified major metabolite of CBC shares significant homology with CBT-C with the exception of a hydroxylation at the 2′ position as shown by our NMR analyses, thereby providing further confidence in our structure assignment. 

Overall, in HLMs, we postulate CBC was initially epoxidized and rearranged into 2′-hydroxycannabicitran. The kinetics and potential biological activity of this hypothesized reaction is entirely unknown. However, a similar reaction has been observed in cannabigerol (CBG) [34,57]. Importantly, epoxidized CBG metabolites after cyclic rearrangement have been shown to be bioactive, showing anti-inflammatory activity in microglia cells [58]. Roy et al. investigated the potential for anti-inflammatory activity of their aforementioned CBC metabolites and determined 6′, 7′-dihydroxy-CBC to favor an anti-inflammatory phenotype by a reduction in nitric oxide production in microglia following lipopolysaccharide challenge to induce an inflammatory response. Contrastingly, 8′-hydroxy-CBC was pro-inflammatory by the same metric [35].

To date, no studies have been published that explore the potential biological activity of CBC metabolites to CB_1_R and CB_2_R; therefore, in a first step, we employed in silico molecular docking. The binding pocket of CB_1_R is known to be rather plastic [59], thus explaining large ligand tolerance of the explored cannabinoids in the orthosteric site. Between the two receptors, (+)-CBC was ranked the highest in docking scores. No other stereospecific patterns were discernable between the remaining ligands tested. There were fewer overall predicted interactions of the ligands at the CB_2_R, but (S)-2′-hydroxy-(−)-cannabicitran was ranked surprisingly high despite not boasting any noted hydrogen bonds or π–π stacking. However, in in vitro competitive inhibition binding assays, 2′-hydroxycannabicitran showed no binding affinity to CB_1_R or CB_2_R. The metabolite is comprised of a rather bulky tetracyclic three-dimensional structure, which may contribute to steric hindrance in displacing [^3^H]CP 55,940 from the orthosteric site of CB_1_R or CB_2_R. It is known that ligands of CB_1_R, such as anandamide and (−)-Δ^9^-THC, access the binding pocket of the receptor by entering via the plasma membrane and pass through residues Phe174 and Phe177 by forming π–π interactions [60]. While we did observe some of these interactions in other ligands tested, the molecular docking experiments do not consider these more dynamic processes and may explain the discrepancies between the results of molecular docking and in vitro competitive binding assays observed in the present study. Novelly, from our in vitro assays, we identified that CBT-C weakly binds to CB_2_R, but does not significantly bind to CB_1_R. A key finding was that 2′-hydroxycannabicitran does not bind to CB_1_R or CB_2_R, so any potential biologic activity would occur at other yet-to-be-determined receptors. Importantly, the binding assays used in the present study do not access allosteric modulation and do not capture potential pharmacodynamics relationships between cannabinoids, metabolites, and receptors.

## 5. Conclusions

Here, we describe for the first time the structure of the major metabolite of CBC resulting from oxidative metabolism by human liver microsomes and its interaction with CB_1_ and CB_2_ receptors. We determined that said primary oxidized metabolite is 2′-hydroxycannabicitran and confirmed its structure via NMR spectroscopy. Further research is needed to establish additional fundamental aspects of CBC metabolism including identification of other phase I-oxidized (+16 Da and +32 Da) metabolites, phase II metabolites, and determination of the clinical pharmacokinetics of CBC and its metabolites. Furthermore, the activity of CBC and CBC metabolites on targets other than CB_1_R and CB_2_R that may have therapeutic merit, such as the TRP family of receptors, still needs to be assessed.

## Figures and Tables

**Table 1:** ^1^H and ^13^C peak assignments for CBC in ACN-d_3_ with detailed HMBC correlations listed. Chemical shifts were aligned to the residual solvent peak of acetonitrile.

1H Chemical Shift (δ ppm)	^1^H Multiplicity *J* (Hz)	Assignment	Integration	^13^C Chemical Shift (δ ppm)	HMBC Correlations
6.814	s	OH	1H	-	5, 6
6.580	dd (0.6, 10.1)	1′	1H	116.919	1, 5, 6, 2′, 3′, 4′, 9′
6.176	s	4	1H	107.543	1, 2, 5, 6, 1′, 1″
6.130	m	2	1H	107.962	1, 4, 5, 6, 1′, 1″
5.520	d (10.0)	2′	1H	126.980	1, 6, 3′, 4′, 9′
5.104	tq (1.4, 7.3)	6′	1H	124.150	4′, 5′, 8′, 10′
2.424	dd (7.0, 7.8)	1″	2H	35.443	2, 3, 4, 2″
2.068	q (8.0)	5′	2H	22.381	3′, 4′, 6′, 7′
1.641	s	8′	5H	24.787	6′, 7′, 10′
1.625	m	4′		40.543	2′, 3′, 4′, 9′
1.555	s	10′	5H	16.658	6′, 7′, 8′
1.544	m	2″		30.620	3, 1″, 3″, 4″
1.329	m	4″	7H	22.232	3″, 5″
1.319	s	9′		25.452	1′, 2′, 3′, 4′
1.278	m	3″		31.205	1″, 2″, 4″, 5″
0.887	t (7.0)	5″	3H	13.340	3″, 4″
-		1	-	153.947	
-		3	-	144.776	
-		5	-	152.295	
-		6	-	106.905	
-		3′	-	77.815	
-		7′	-	131.386	

**Table 2:** ^1^H and ^13^C peak assignments for the purified major oxidized CBC metabolite (2′-hydroxycannabicitran) in ACN-d_3_ with detailed HMBC correlations listed. 1H* refers to a peak that integrated on the ^1^H spectra for two protons, but upon the results of the DEPT-135 experiment was rectified to represent one proton. Chemical shifts were aligned to the residual solvent peak of acetonitrile.

^1^H Chemical Shift (δ ppm)	^1^H Multiplicity *J* (Hz)	Assignment	Integration	^13^C Chemical Shift (δ ppm)	^13^C Multiplicity	HMBC Correlations
6.266	s	4	1H	109.261	CH	2, 5, 6, 1″
6.228	s	2	1H	110.699	CH	1, 4, 6, 1″
3.749	dd (1.9, 5.8)	2′	1H	71.161	CH	6, 1′, 3′, 6′
3.060	d (6.1)	OH	1H	-	-	1′, 2′, 3′
2.719	t (2.3)	1′	1H*	37.036	CH	1, 5, 6, 2′, 3′, 5′, 6′, 7′
2.485	dd (7.4)	1″	2H	36.583	CH_2_	2, 3, 4, 2″
2.258	ddd (2.8, 5.3, 11.5)	6′	1H	48.335	CH	6, 1′, 2′, 8′b
1.690	ddd (0.9, 6.1, 15.4)	4′b	1H	37.398	CH_2_	2′, 3′, 5′, 6′, 7′
1.554	m	2″	2H	31.918	CH_2_	3, 1″, 3″, 4″
1.478	s	8′b	4H	29.767	CH_3_	1, 6′, 7′, 8′a
1.455	td (7.1, 15.3)	4′a		37.398	CH_2_	5′, 9′
1.321	m	4″	7H	23.166	CH_2_	3″, 5″
1.315	s	9′		24.796	CH_3_	5, 2′, 3′, 4′, 5′
1.270	m	3″		32.144	CH_2_	1″, 2″, 4″, 5″
1.152	dt (5.9, 12.8)	5′b	1H	22.418	CH_2_	1′, 3′, 6′
0.948	s	8′a	3H	23.936	CH_3_	2′, 5′, 6′, 7′, 8′b
0.880	t (7.1)	5″	3H	14.300	CH_3_	3″, 4″
0.373	tdd (6.2, 11.9, 13.4)	5′a	1H	22.418	CH_2_	4′, 6′, 7′
-		3′	-	78.238	C	
-		7′	-	84.205	C	
-		6	-	113.632	C	
-		3	-	143.421	C	
-		5	-	156.996	C	
-		1	-	158.480	C	

**Table 3:** CB_1_R (PDB code: 5XR8) SP precision model ligands ranked. Distances were measured based on the closest atom-to-atom distance between a given residue and the ligand.

Computer Ranking	Ligand	Docking Score	Highlighted Residue-Ligand Interactions	Distance of Interaction (Å)	Type of Interaction
1	(+)-CBC	−10.600	Ser505-OH	1.69	H-bond
			Phe170-AR	3.70	π–π stacking
			Phe170-P	3.70	π–π stacking
			Phe268-AR	3.78	π–π stacking
			Phe268-P	3.78	π-π stacking
2	(−)-Δ^9^-THC	−10.569	Ser505-OH	1.84	H-bond
			Phe268-AR	3.75	π–π stacking
			Phe170-AR	3.84	π–π stacking
3	(−)-CBC	−9.668	Ser505-OH	1.81	H-bond
			Phe174-AR	3.33	π–π stacking
			Phe268-P	3.46	π–π stacking
			Phe268-AR	3.62	π–π stacking
			Phe170-AR	3.70	π–π stacking
			Phe170-P	3.70	π–π stacking
4	(R)-2′-OH-(+)-cannabicitran	−9.254	Ile267-OH	2.49	H-bond
5	(+)-CBT-C	−8.345	Phe170-AR	3.38	π–π stacking
6	(−)-CBT-C	−7.967	Phe170-AR	3.55	π–π stacking
			Phe268-AR	3.55	π–π stacking
7	(S)-2′-OH-(−)-cannabicitran	−6.796	Phe170-AR	3.23	π–π stacking

Abbreviation AR indicates aromatic ring and P indicates a pyran-type ring interaction.

**Table 4:** CB_2_R (PDB code: 5ZTY) SP precision model ligands ranked. Distances were measured based on the closest atom-to-atom distance between a given residue and the ligand.

Computer Ranking	Ligand	Docking Score	Highlighted Residue-Ligand Interactions	Distance of Interaction (Å)	Type of Interaction
1	(+)-CBC	−8.545	Phe87-P	3.81	π–π stacking
			Phe183-P	3.99	π–π stacking
2	(+)-CBT-C	−8.472	-	-	-
3	(−)-Δ^9^-THC	−8.312	Phe87-AR	3.68	π–π stacking
			Phe183-AR	3.99	π–π stacking
4	(S)-2′-OH-(−)-cannabicitran	−8.042	-	-	-
5	(−)-CBT-C	−7.949	Phe183-AR	3.28	π–π stacking
6	(−)-CBC	−7.892	Phe87-P	3.57	π–π stacking
			Phe87-AR	3.57	π–π stacking
			Phe183-P	4.17	π–π stacking
7	(R)-2′-OH-(+)-cannabicitran	−7.810	Ser90-OH	2.67	H-bond
			Phe183-AR	3.63	π–π stacking
			Phe87-AR	3.91	π–π stacking

Abbreviation AR indicates aromatic ring and P indicates a pyran-type ring interaction.

**Table 5:** Calculated K_i_ values (nM) and Hill slopes determined from competitive inhibition binding assays at CB_1_R and CB_2_R. Data shown are the means ± S.E.M. of four to ten experiments.

	CB_1_R		CB_2_R	
Ligand	K_i_ (nM)	Hill Slope	K_i_ (nM)	Hill Slope
CBC	3500 ± 1200	−0.27 ± 0.51	301 ± 72	−1.32 ± 0.36
2′-hydroxycannabicitran	>10,000	-	>10,000	-
CBT-C	>10,000	-	4200 ± 410	−1.15 ± 0.14
Δ^9^-THC	13.2 ± 1.9	−0.73 ± 0.09	22.6 ± 5.0	−0.96 ± 0.16
CP 55,940	1.77 ± 0.32	−0.87 ± 0.18	-	-
WIN 55,212	-	-	3.9 ± 1.6	−1.07 ± 0.13

## Data Availability

The original contributions presented in this study are included in the article/Supplementary Material; further inquiries can be directed to the corresponding author. However, the raw data supporting the conclusions of this article will be made available by the authors on request.

## Supplementary Materials

The following supporting information can be downloaded at: https://www.mdpi.com/article/10.3390/metabo14060329/s1, Supplemental Materials S1.1 Experimental Design Flowchart, S1.2 Optimization of CBC HLM Incubation Parameters, S1.3 Isolation of Upscaled CBC Metabolites Generated by HLMs, S1.4 Isolation of Fractions after Incubation of CBC with Hydrogen Peroxide, S1.5 CBC Major Metabolite Identified via GC-MS/MS, S2.1 Recapitulation and Comparison to Roy et al., S3.1 Purity of 2′-hydroxycannabicitran, S3.2 Cannabichromene NMR Experiments, S3.3 2′-hydroxycannabicitran NMR Experiments, S3.4 Cannabicitran and Overlay NMR Experiments, S4.1 Structures Imported for Molecular Docking, S4.2 Cannabinoid 1 Receptor Molecular Docking, and S4.3 Cannabinoid 2 Receptor Molecular Docking.
