# Supplementary material for: Identification and Characterization of Cannabichromene’s Major Metabolite Following Incubation with Human Liver Microsomes
Source: Metabolites. 2024 Jun 13;14(6):329. doi: 10.3390/metabo14060329 (PMC11206029; doi:10.3390/metabo14060329)
Supplement: Supplementary file 1 [file metabolites-14-00329-s001.zip › CBC_Metabolite_Supplementary_Materials_S1.pdf]

Supplemental Materials S1

Identification and Characterization of Cannabichromene's Major Metabolite Following Incubation with Human Liver Microsomes

Alexandra M. Ward[1], Touraj Shokati[2], Jost Klawitter[2], Jelena Klawitter[2], Vu Nguyen[1], Laura Kozell[3, 4, 5], Atheir I. Abbas[3, 4, 5], David Jones[6], and Uwe Christians[2,*]

[1]*Department of Pharmaceutical Sciences, Skaggs School of Pharmacy and Pharmaceutical Sciences, University of Colorado Anschutz Medical Campus, Aurora, CO, USA; alexandra.ward@cuanschutz.edu, vu.t.nguyen@cuanschutz.edu.*

[2]*iC42 Clinical Research and Development, Department of Anesthesiology, School of Medicine, University of Colorado Anschutz Medical Campus, Aurora, CO, USA; touraj.shokati@cuanschutz.edu, jost.klawitter@cuanschutz.edu, jelena.klawitter@cuanschutz.edu, uwe.christians@cuanschutz.edu.*

[3]*Department of Behavioral Neuroscience, Oregon Health & Science University, Portland, OR, USA; abbasat@ohsu.edu, kozellla@ohsu.edu.*

[4]*Department of Psychiatry, Oregon Health & Science University, Portland, OR, USA.*

[5]*Veterans Affairs Portland Health Care System, Portland, OR, USA.*

[6]*Department of Pharmacology, School of Medicine, University of Colorado Anschutz Medical Campus, Aurora, CO, USA; david.jones@cuanschutz.edu.*

*Correspondence: uwe.christians@cuanschutz.edu

Table of Contents Supplemental Materials S1

S1.1
Experimental Design Flowchart Page 3

S1.2
Optimization of CBC HLM Incubation Parameters Page 4

S1.3
Isolation of Upscaled CBC Metabolites Generated by HLMs Page 9

S1.4
Isolation of Fractions after Incubation of CBC with Hydrogen Peroxide Page 15

S1.5
CBC Major Metabolite Identified via GC-MS/MS Page 19

References Page 28

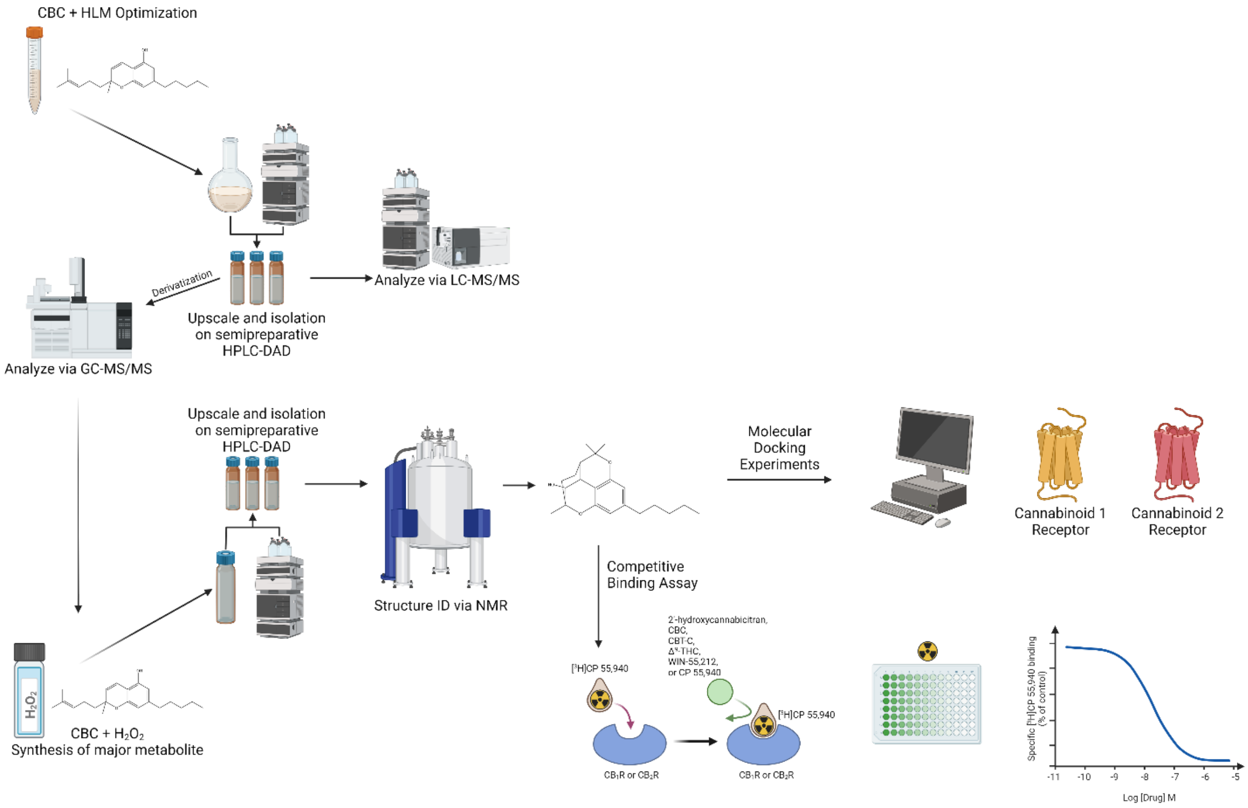

Figure S1.1.1. Experimental Design Flowchart.

S1.2

Optimization of CBC HLM Incubation Parameters

We optimized the incubation parameters of CBC with HLMs, namely incubation time, HLM protein concentration, and CBC concentration.

Please see supplementary figures for representative extracted ion chromatograms of samples (Figure S1.2.1 and Figure S1.2.5), metabolite generation plots (Figure S1.2.2- S1.2.4) and controls (Figure S1.2.6) used to determine the optimal parameters of 0.5 mg/mL HLM, 50 µg/mL CBC, and a 40 min incubation time.

Methods

We used a quantitative online trapping tandem mass spectrometry (LC/LC-MS/MS) approach for the determination of metabolite yields. The instrumentation for this experiment was a 1290 Infinity LC injector HTS, G1379A degasser (x2), G1312A binary pump (x2), G1316A column compartment (all Agilent Technologies, Santa Clara, CA, USA) equipped with an API 4000 LC/MS-MS system as a detector (AB Sciex, Concord, ON, Canada). The analytical columns were 2 x Eclipse XDB-C8 5 µm, 4.6 x 250 mm in series and the extraction column was an XDB-C8 (Agilent Technologies, Santa Clara, CA, USA). The HPLC/MS system was controlled and data was processed using Analyst 1.6.2 software (AB Sciex, Concord, ON, Canada).

The injection volume was 25 µL with the column temperature set at 60 °C. Mobile phase A was water + 0.1% formic acid and mobile phase B was methanol + 0.1% formic acid. The pump gradient associated with the extraction column was as follows: 0.0 min, 15% B, 3000 µL/min; 1.0 min, 15% B, 3000 µL/min; 1.1 min, 98% B, 200 µL/min; 23.0 min, 98% B, 200 µL/min; 23.1 min, 98% B, 3000 µL/min; 23.2 min, 15% B, 3000 µL/min; and 25.0 min, 15% B, 3000 µL/min. The flow was switched from the extraction column to the analytical column after 1.1 min. The pump gradient associated with the analytical columns was: 0.0 min, 75% B; 1.0 min, 75% B; 14.0 min, 95% B; 17.0 min, 98% B; 21.0 min, 98% B; 21.1 min, 75% B; and 25.0 min, 75% B; the flow rate was 1000 µL/min throughout the gradient.

The mass spectra were collected in positive electrospray ionization (ESI) MRM mode and the following masses were collected as both Q1 and Q3 masses with a 25.0 msec collection time for each (all $[M+H]^+$): 315.5/315.5 (CBC), 331.5/331.5 (CBC-OH), 347.5/347.5 (CBC-2OH), 363.5/363.5 (CBC-3OH), 379.5/379.5 (CBC-4OH), 395.5/395.5 (CBC-5OH), 324.5/324.5 (CBC-d_9), 329.5/329.5(CBC-1OH-H2), 345.5/345.5 (CBC-2OH-H2), 361.5/361.5 (CBC-3OH-H2), 377.5/377.5 (CBC-4OH-H2), 393.5/393.5 (CBC-5OH-H2), 343.5/343.5 (CBC-2OH-2H2), 359.5/359.5 (CBC-COOH), 403.5/403.5 (CBC-2COOH), and 447.5/447.5 (CBC-3COOH). The mass spectrometer parameters were as follows: collision gas 10 psi, curtain gas 30 psi, ion source gas 50 psi, ion spray voltage 5500 V, source temperature 550 °C, declustering potential 95 V, and collision energy 8 eV.

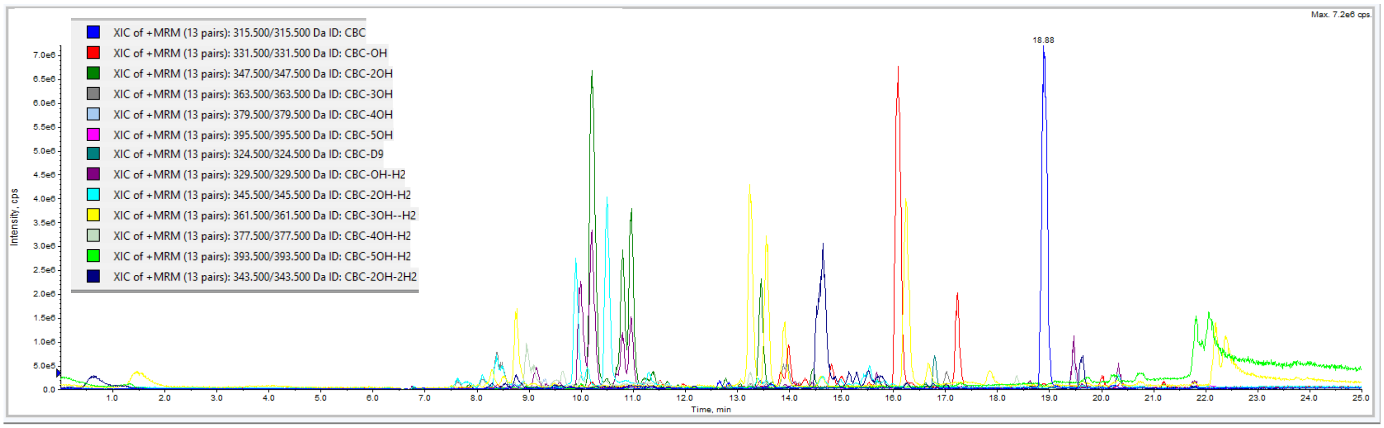

Figure S1.2.1. Representative extracted ion chromatogram recorded during time dependence experiments with an incubation time = 40 min. HLMs used were H0620/Lot #1810003 from Xenotech, Lenexa, KS, USA.

A plot of the peak areas at the different incubation times (5, 10, 30, 40, 60, 80, 90, and 120 min), microsomal protein concentrations (0.05, 0.1, 0.25, 0.5, 1, 2.5, 5.0, and 10.0 mg/mL) and CBC concentrations (1.0, 2.5, 5.0, 10.0, 25.0, 50.0, 100.0, and 200.0 μg/mL) studied are shown in the next figures.

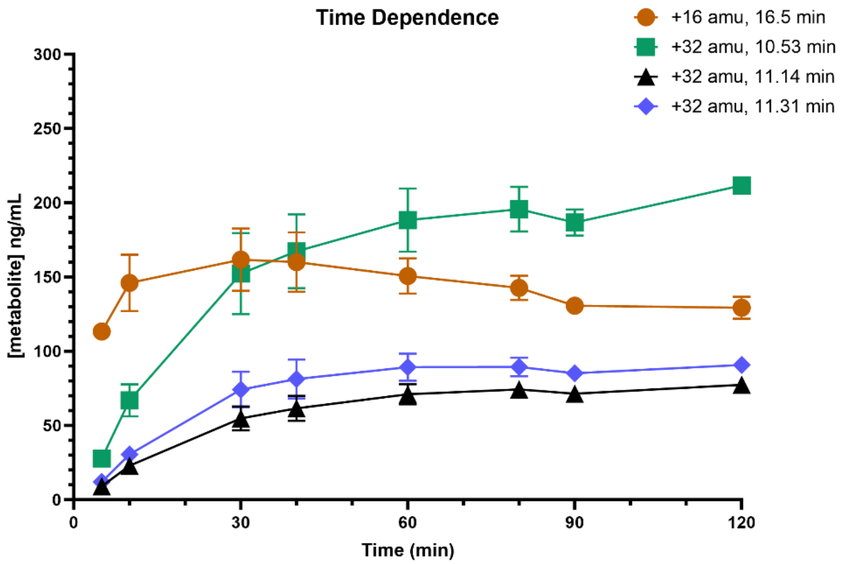

Figure S1.2.2. Time-dependent generation of select metabolites with +16 and +32 amu mass shift during incubation of CBC (10 μg/mL) with HLM (1 mg/mL). The data is presented as means ± standard deviation (n=3). CBC was incubated with HLMs for 5, 10, 30, 40, 60, 80, 90, and 120 min. 40 min was determined to be the optimal incubation time across many observed metabolites. HLMs used were H0620/Lot #1810003 from Xenotech, Lenexa, KS, USA).

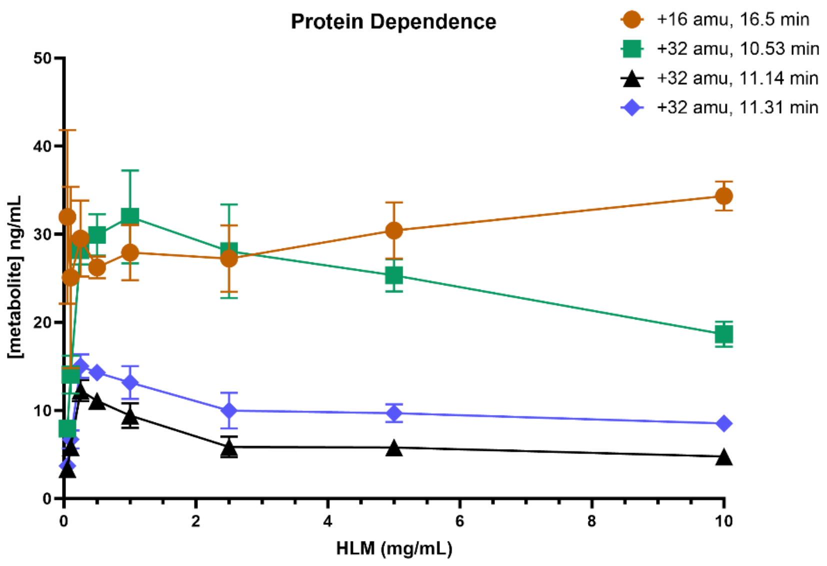

Figure S1.2.3. Microsomal protein-dependent generation of select metabolites with +16 and +32 amu mass shift during incubation of CBC (10 µg /mL) for 40 min with varying concentrations of HLM. The data is presented as means ± standard deviation (n=3). CBC was incubated with 0.05, 0.10, 0.25, 0.50, 1.00, 2.50, 5.00, and 10.00 mg/mL HLM. 0.50 mg/mL HLM was determined to be the optimal concentration with the best yield for most CBC metabolites. HLMs used were H0620/Lot #1810003 from Xenotech, Lenexa, KS, USA.

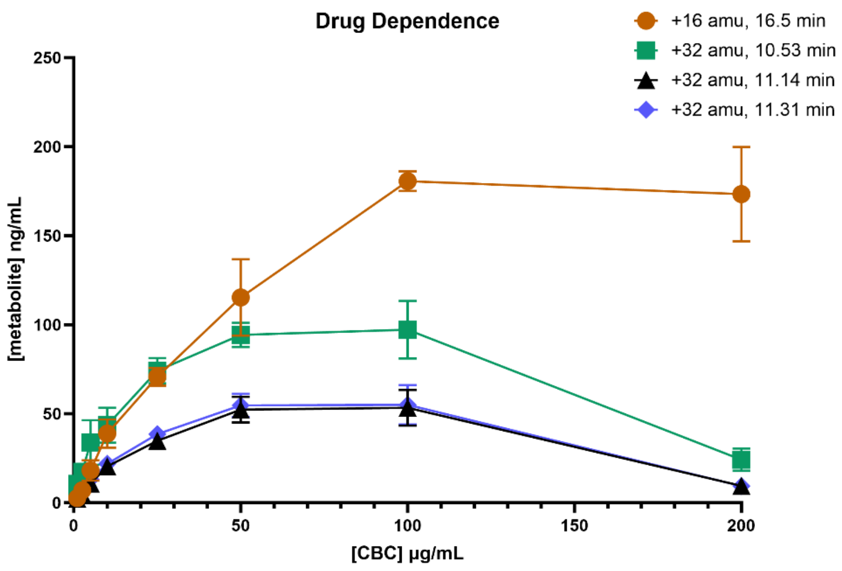

Figure S1.2.4. CBC-dependent generation of select metabolites with +16 and +32 amu mass shift during incubation of HLM (0.5 mg/mL) for 40 min. The data is presented as means ± standard deviation (n=3). Varying concentrations of CBC (1.0, 2.5, 5.0, 10.0, 25.0, 50.0, 100.0, and 200.0 µg/mL) were tested. 50 µg/mL CBC was determined to result in the best yield of most metabolites. HLMs used were H0620/Lot #1810003 from Xenotech, Lenexa, KS.

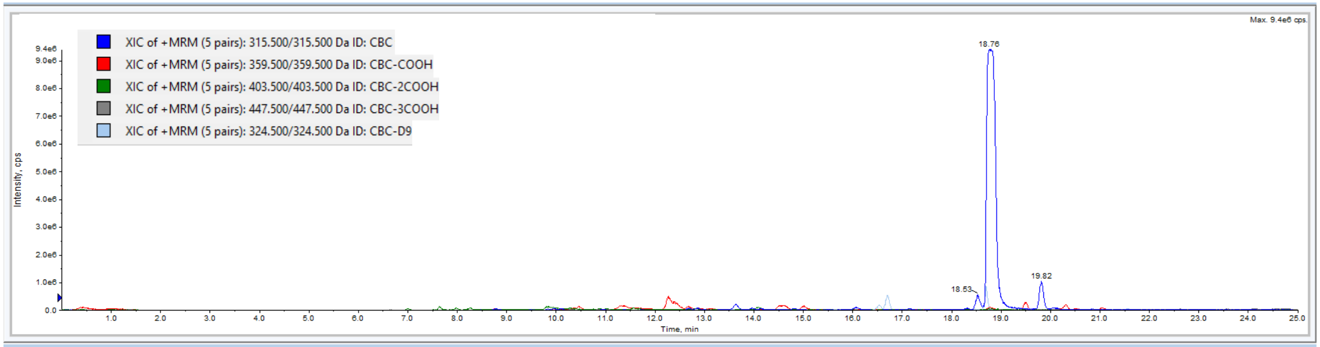

Figure S1.2.5. Representative extracted ion chromatogram during CBC-dependence studies (50 µg/mL), HLM (0.5 mg/mL), incubation time (40 min) displaying potential carboxylated metabolites. HLMs used were H0620/Lot #1810003 from Xenotech, Lenexa, KS.

Using the optimized incubation conditions, it was specifically assessed if carboxylated metabolites were formed. This assessment was carried out since carboxylated metabolites are major oxidative metabolites of several cannabinoids such as THC and CBD. These experiments indicated that CBC incubation with HLM under the present conditions does not significantly produce any carboxylated metabolites.

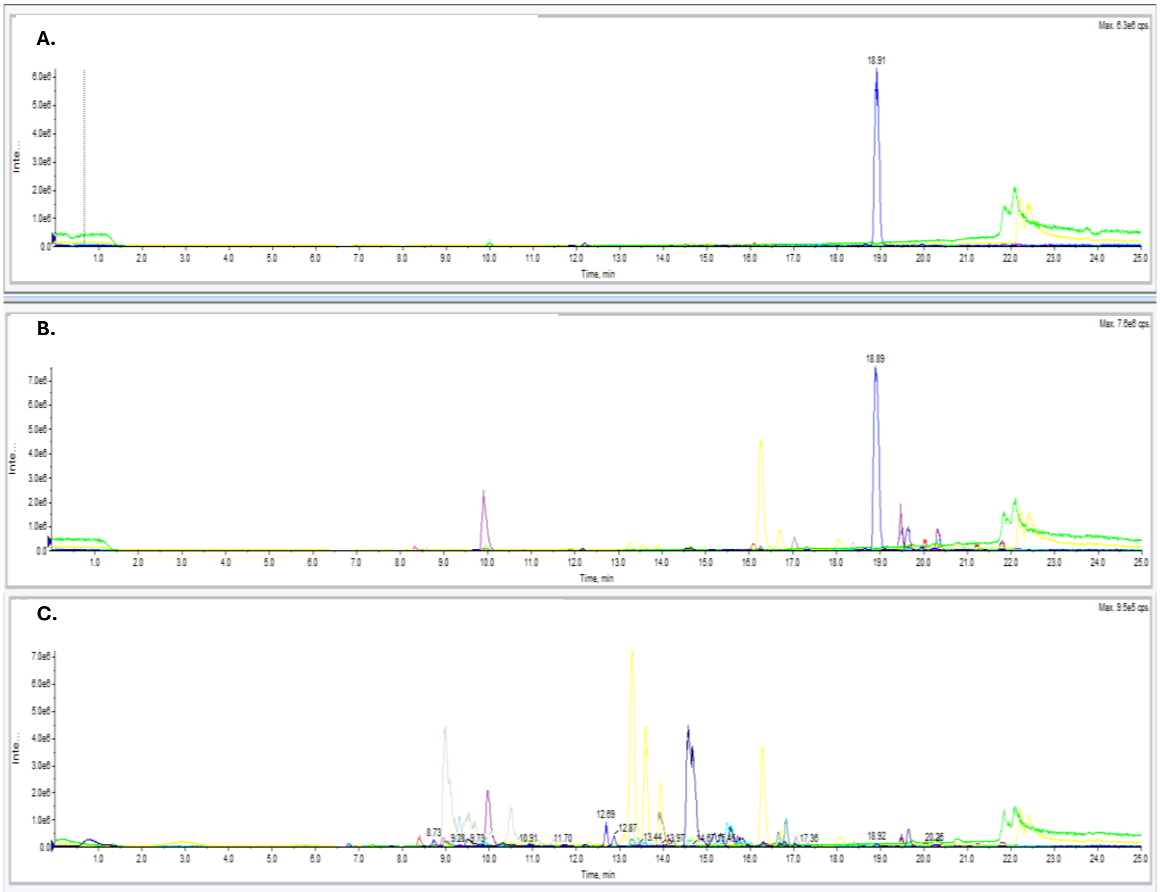

Figure S1.2.6. Representative extracted ion chromatograms from optimization control samples. The legend of chromatogram tracer colors is identical to that of Figure S1.2. (A) displays the without HLM control (+ CBC 10 µg/mL + NADPH-generating system), (B) displays the without NADPH-generating system control (+ CBC 10 µg/mL +HLM 1 mg/mL), and (C) displays the without CBC control (+HLM 1 mg/mL + NADPH-generating system). The incubation time was 60 min. HLMs used were H0620/Lot #1810003 from Xenotech, Lenexa, KS.

Based on these control extracted ion chromatograms, it was concluded that there is very little background noise in the +16 and +32 amu channels, lending confidence to our identification of metabolite peaks.

S1.3

Isolation of Upscaled CBC Metabolites Generated by HLMs

Utilizing the optimized incubation parameters (0.5 mg/mL HLM, 50 µg/mL CBC, and a 40 min incubation time), we upscaled the generation of CBC metabolites by making a 200x batch in HLMs. We then isolated all metabolite-containing fractions via semipreparative HPLC-DAD. Isolated, pure metabolite fractions were then analyzed via GC-MS/MS and HPLC-MS/TOF.

Please see supplementary Figures S1.3.1 and S1.3.2 for representative controls, Figures S1.3.3-S1.3.8 for fractions collected, and Table S.1.3.1 for tabulated details of fractions collected.

Methods

Prior to adding drug, the NADPH-generating system was incubated at a constant temperature of 37 ± 2 °C, 150 rpm, for 10 minutes on a shaker (C24 Incubator Shaker, New Brunswick Scientific, Edison, NJ, USA). Concentrations of buffer constituents were: Na^+/K^+- phosphate buffer (0.1 M, pH 7.4), containing $MgCl_2$ (3.0 mM), EDTA (1.0 mM), NADP (1.0 mM), isocitric acid (5.0 mM), and isocitric dehydrogenase (1 Unit/mL). The final volume of the reaction was 100 mL. After 40 min, the reaction was quenched with a 1:1 volume of ice-cold acetonitrile and the slurry was transferred to 50 mL conical tubes and centrifuged at 4 °C, 685 x g, for 10 minutes. The supernatants were combined following protein precipitation and centrifugation into a 1-L separatory glass funnel and extracted using dichloromethane. The higher density organic phase was retained and dried under a flow of nitrogen at room temperature; once dry, the sample was reconstituted in 1.5 mL of pure acetonitrile and transferred to an amber glass HPLC vial and immediately isolated via semi-preparative HPLC-DAD. The resulting fractions were stored at -80 °C until further analysis.

The HPLC components for the semi-preparative isolation of CBC metabolites were as follows: G1322A degasser, G1312A binary pump, G1329B 1260 ALS, G1315B DAD, G1364B fraction collector (all Agilent Technologies, Santa Clara, CA, USA) and an external column compartment ThermaSphere (Phenomenex, Torrance, CA, USA). The HPLC-DAD system was controlled and data was processed using ChemStation software revision 04.03.087 (Agilent Technologies, Santa Clara, CA, USA). The semi-preparative columns were 4x Eclipse XDB-C8, 5 µm, 9.4 x 250 mm connected in series (Agilent Technologies, Santa Clara, CA, USA). The flow rate was 3.5 mL/min and the mobile phases were HPLC grade water (mobile phase A) and HPLC grade acetonitrile (mobile phase B). The elution gradient for isolating CBC metabolites was 0.0 min, 40% B; 22.0 min, 52% B; 48.0 min, 75% B; 50.0 min, 96% B; 54.0 min, 99% B; 69.0 min, 99% B; 70.0 min, 40% B, and 84.0 min, 40% B. The injection volume was 100 µL; UV absorbance was monitored at 210 nm and 231 nm. Fractions were collected based on UV signal and are available in Table S.1.3.1 and Figure S.1.3.5.- Figure S1.3.8. Fractions were dried under nitrogen flow at room temperature and reconstituted in 1.5 mL of pure acetonitrile which was aliquoted into 3 vials. Isolated metabolites and controls were analyzed utilizing both LC-MS/MS and GC-MS/MS to assure retention time and fragmentation pattern in each respective assay. Samples were stored in a -80 °C freezer until analysis.

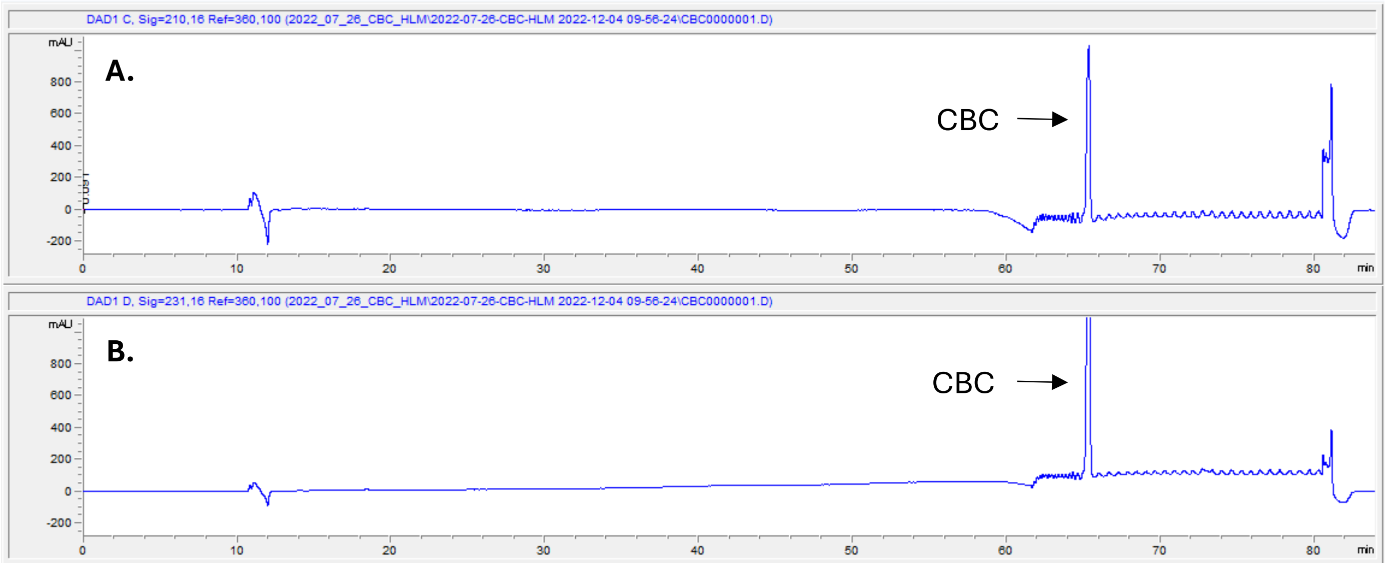

Figure S1.3.1. UV chromatograms of a representative control without HLM (+50 µg/mL CBC + NADPH-generating system, incubation for 40 min) sample. (A) displays the UV chromatogram measured at 210 nm and (B) displays the UV chromatogram measured at 231 nm.

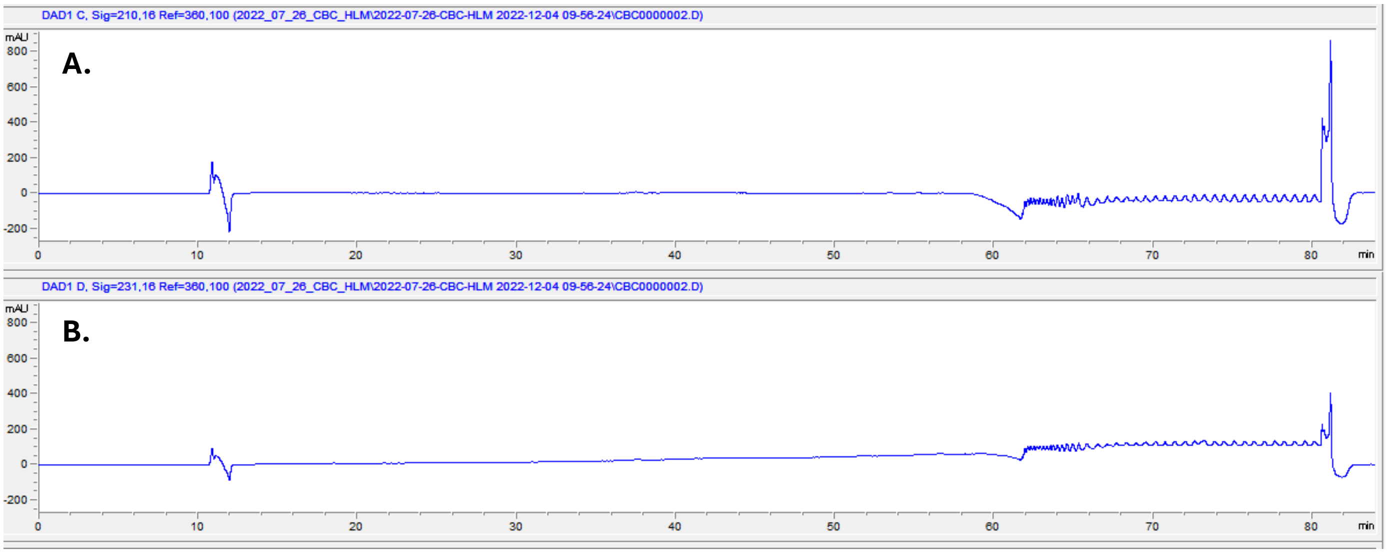

Figure S1.3.2. UV chromatograms of a representative control without CBC (+0.5 mg/mL HLM and NADPH-generating system, incubation for 40 min) sample. (A) displays the UV chromatogram measured at 210 nm and (B) displays the UV chromatogram measured at 231 nm. HLMs used were H2640/Lot #1910096 from Xenotech, Lenexa, KS.

From these control samples, we determined the expected retention time of CBC and that no significant UV signal were generated from the HLM/NADPH-generating system alone when measured at 210 and 231 nm.

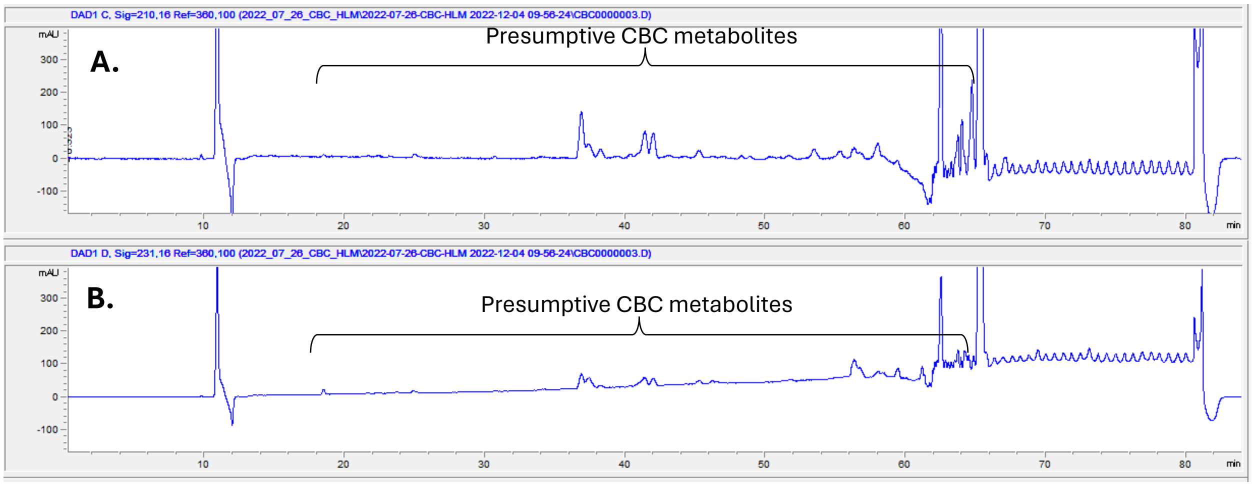

Figure S1.3.3. UV Chromatograms of representative sample of upscaled incubation of 50 µg/mL CBC with 0.5 mg/mL HLMs for 40 min. (A) displays the UV chromatogram measured at 210 nm and (B) displays the UV chromatogram measured at 231 nm. HLMs used were H2640/Lot #1910096 from Xenotech, Lenexa, KS. Presumptive CBC metabolite peaks are marked by brackets.

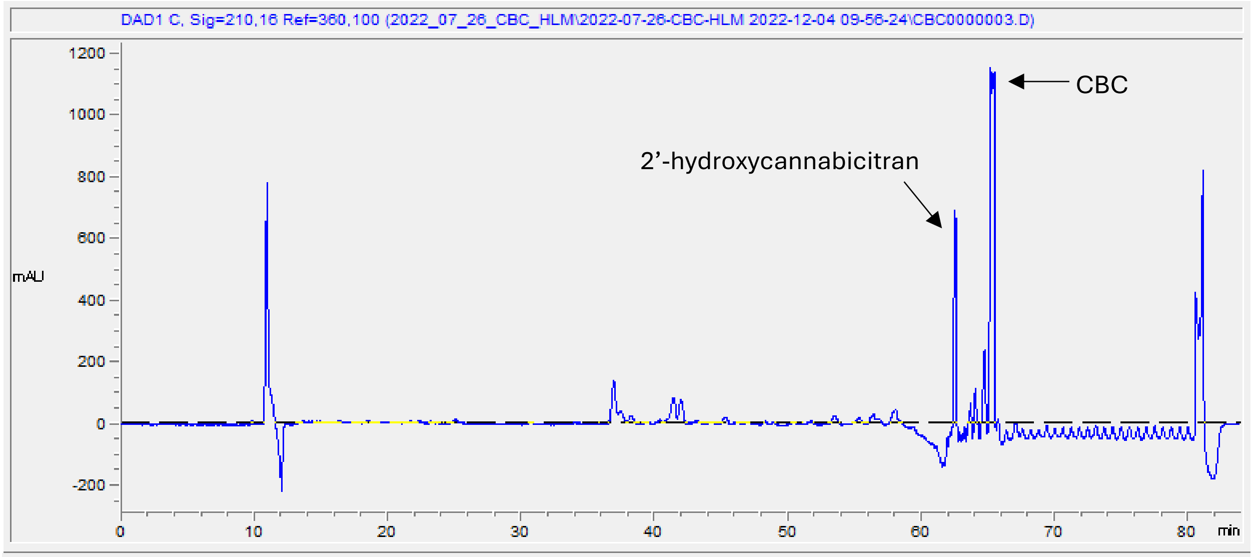

Figure S1.3.4. UV chromatogram of upscaled incubation of CBC with HLMs (50 µg/mL CBC with 0.5 mg/mL HLMs for 40 min) measured at 210 nm. CBC and 2’-hydroxycannabicitran are labeled on the chromatogram. HLMs used were H2640/Lot #1910096 from Xenotech, Lenexa, KS.

From this upscaled CBC HLM incubation, isolated fractions were collected (Table S1.3.1 and Figures S1.3.5-S1.3.8).

Table S1.3.1. Fractions collected from the upscaled CBC HLM incubation. Fraction 30 is highlighted and represents 2'-hydroxycannabicitran.

Fraction #	Time		Timeslices (min)	Flow rate (mL/min)	# of injections	volume collected (mL)
	On	Off				
1	18.2	18.8	0.6	3.5	14	27.0
2	21.3	21.9	0.6	3.5	14	29.4
3	22.0	22.8	0.8	3.5	14	39.2
4	23.4	23.8	0.4	3.5	14	19.6
5	24.6	25.4	0.8	3.5	14	39.2
6	30.4	31.0	0.6	3.5	14	29.4
7	33.7	34.5	0.8	3.5	14	39.2
8	36.4	37.2	0.8	3.5	14	40.2
9	37.2	37.9	0.6	3.5	14	30.9
10	37.9	38.7	0.8	3.5	14	36.8
11	39.1	39.8	0.7	3.5	14	34.3
12	40.0	40.7	0.7	3.5	14	31.9
13	41.0	41.7	0.7	3.5	14	33.8
14	41.7	42.4	0.7	3.5	14	32.8
15	42.7	43.6	0.9	3.5	14	44.1
16	43.9	44.7	0.8	3.5	14	39.2
17	44.9	45.7	0.9	3.5	14	41.7
18	46.0	47.2	1.2	3.5	14	58.8
19	48.0	48.5	0.5	3.5	14	24.5
20	48.6	49.3	0.7	3.5	14	34.3
21	50.0	50.8	0.8	3.5	14	39.2
22	51.3	52.0	0.8	3.5	14	36.8
23	53.1	53.9	0.8	3.5	14	39.2
24	54.8	55.8	1.0	3.5	14	49.0
25	55.9	56.6	0.7	3.5	14	34.3
26	56.7	57.2	0.6	3.5	14	27.0
27	57.5	58.4	1.0	3.5	14	46.6
28	59.1	59.7	0.6	3.5	14	29.4
29	60.9	61.6	0.7	3.5	14	31.9
30	62.2	62.8	0.6	3.5	14	29.4
31	63.4	64.4	1.0	3.5	14	49.0
32	64.4	65.0	0.6	3.5	14	27.0
33	65.0	65.9	0.9	3.5	14	44.1

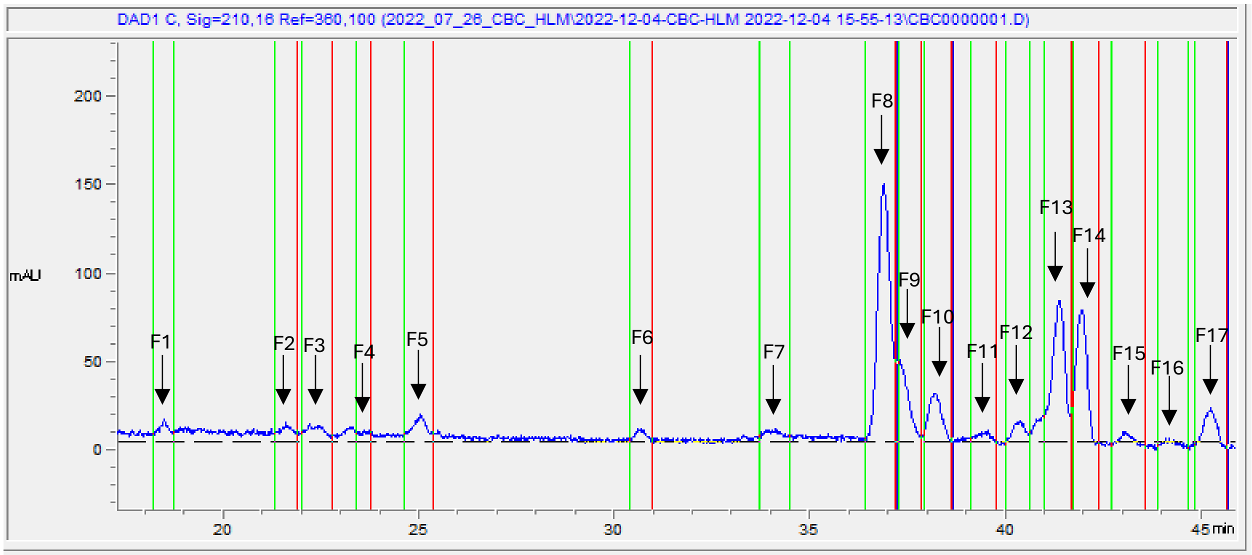

Figure S1.3.5. Zoomed-in UV chromatogram measured at 210 nm displaying fractions 1-17 collected from upscaled CBC HLM incubation (50 µg/mL CBC with 0.5 mg/mL HLMs for 40 min). HLMs used were H2640/Lot #1910096 from Xenotech, Lenexa, KS.

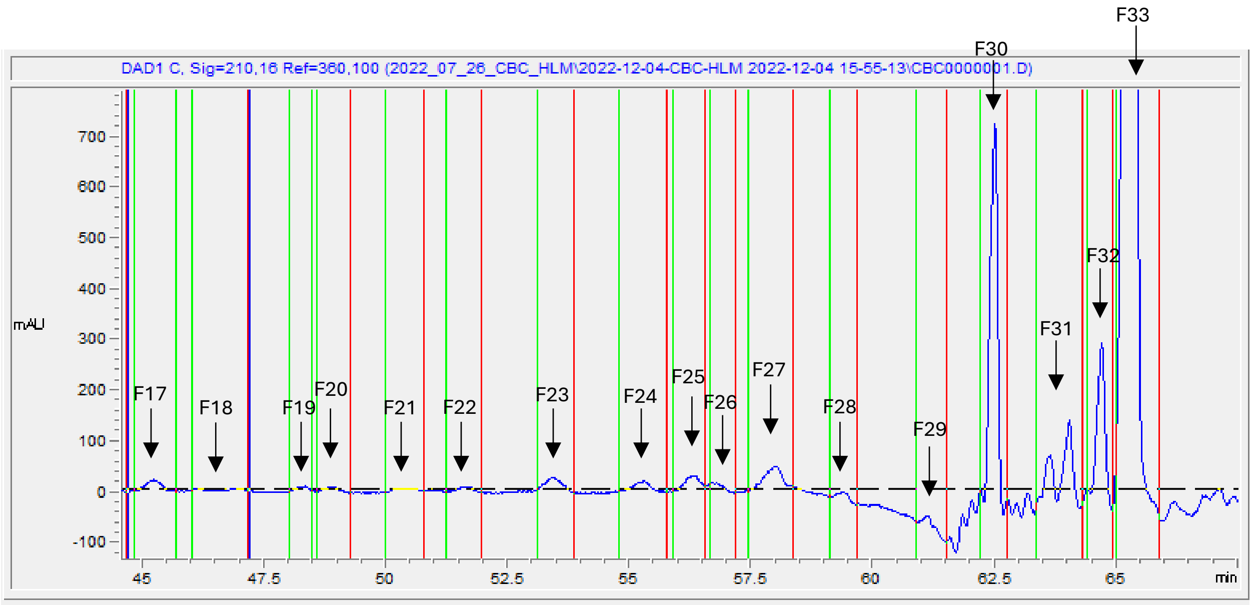

Figure S1.3.6. Zoomed-in UV chromatogram measured at 210 nm displaying fractions 17-33 collected from upscaled CBC HLM incubation (50 µg/mL CBC with 0.5 mg/mL HLMs for 40 min). F30 is 2'-hydroxycannabicitran and F33 is CBC. HLMs used were H2640/Lot #1910096 from Xenotech, Lenexa, KS.

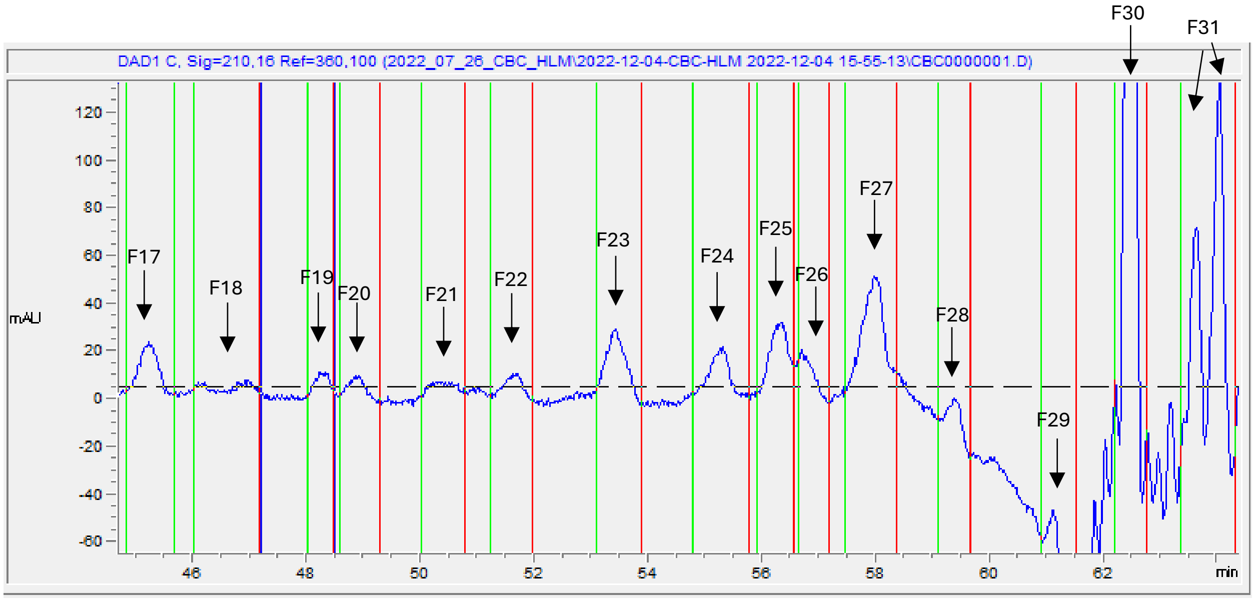

Figure S1.3.7. Zoomed-in UV chromatogram measured at 210 nm displaying fractions 17-31 collected from upscaled CBC HLM incubation (50 µg/mL CBC with 0.5 mg/mL HLMs for 40 min). F30 is 2'-hydroxycannabicitran. HLMs used were H2640/Lot #1910096 from Xenotech, Lenexa, KS.

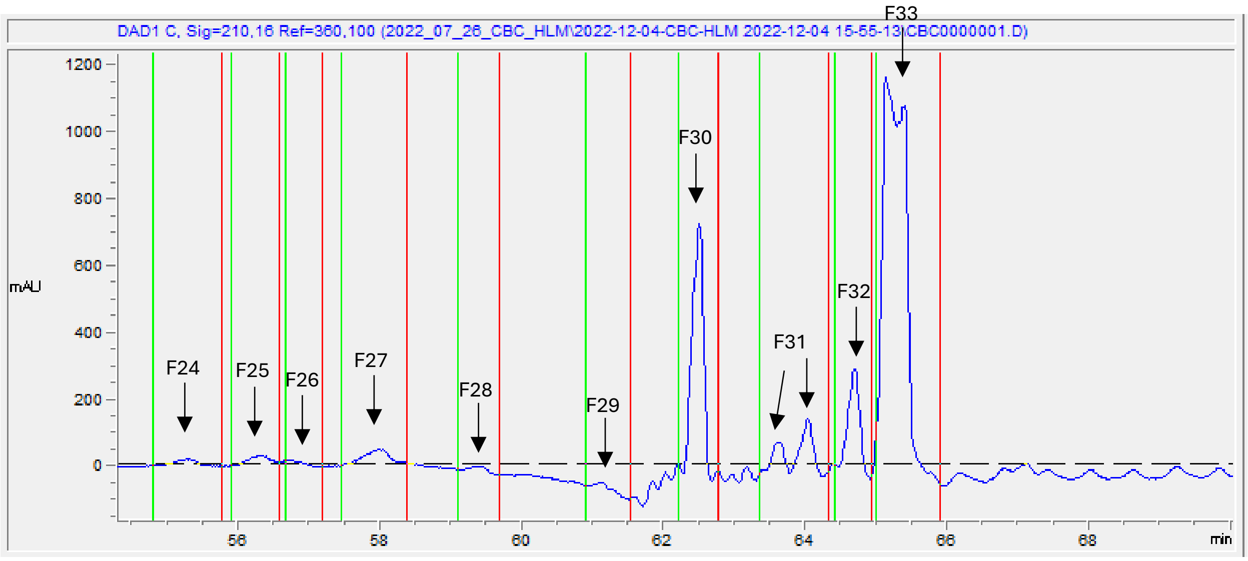

Figure S1.3.8. Zoomed-in UV chromatogram measured at 210 nm displaying fractions 24-33 collected from upscaled CBC HLM incubation (50 µg/mL CBC with 0.5 mg/mL HLMs for 40 min). F30 is 2'-hydroxycannabicitran and F33 is CBC. HLMs used were H2640/Lot #1910096 from Xenotech, Lenexa, KS.

F33 shows oversaturation of the UV detector. This was not a concern as CBC was baseline separated from the material underlying the peak in fraction F32.

S1.4

Isolation of Fractions after Incubation of CBC with Hydrogen Peroxide

Based on a detailed analysis of the fragmentation patterns of the metabolite in F30 using the GC-MS/MS system, we initially hypothesized that the major CBC metabolite generated by incubation with HLM was an epoxide on the 2H-pyran ring of CBC. We sought to determine the structure of said metabolite via NMR and identified a simple synthesis to generate this metabolite. Identity with the enzymatically generated metabolite was confirmed by the identical HPLC retention time, identical UV absorption spectrum, identical GC-MS/MS fragmentation pattern including ion ratios and the same high-resolution MS/TOF spectrum.

We incubated CBC with hydrogen peroxide (H_2O_2) and nonspecifically generated the major metabolite of CBC. We then isolated all detectable generated compounds via semipreparative HPLC-DAD. Isolated fractions could then be analyzed via GC-MS/MS and HPLC-MS/TOF.

Please see supplementary Figure S1.4.1 for a representative control UV chromatogram, Figures S1.4.2-S1.4.4 for fractions collected from the synthetic generation of 2'-hydroxycannabicitran, and Table S1.4.1 for tabulated details of the fractions collected.

Methods

Following HLM incubation, metabolite isolation, and preliminary identification, the major CBC metabolite was produced by chemical synthesis. For this, 0.048 mmol CBC was incubated with 12.89 mmol hydrogen peroxide for 15 minutes at 60 °C, then placed at -20 °C until cool. Isolation of generated products was immediately performed via semi-preparative HPLC-DAD.

The synthetically generated major CBC metabolite was isolated on the same semi-preparative HPLC-DAD system detailed above including equipment, mobile phases, columns, flow rate, injection volume, and UV detection. The elution gradient for isolating the synthetically generated major CBC metabolite was: 0.0 min, 80% B; 10.0 min, 80% B; 30.0 min, 99% B; 34.0 min 99% B; 34.1 min, 80% B; and 46.0 min, 80% B.

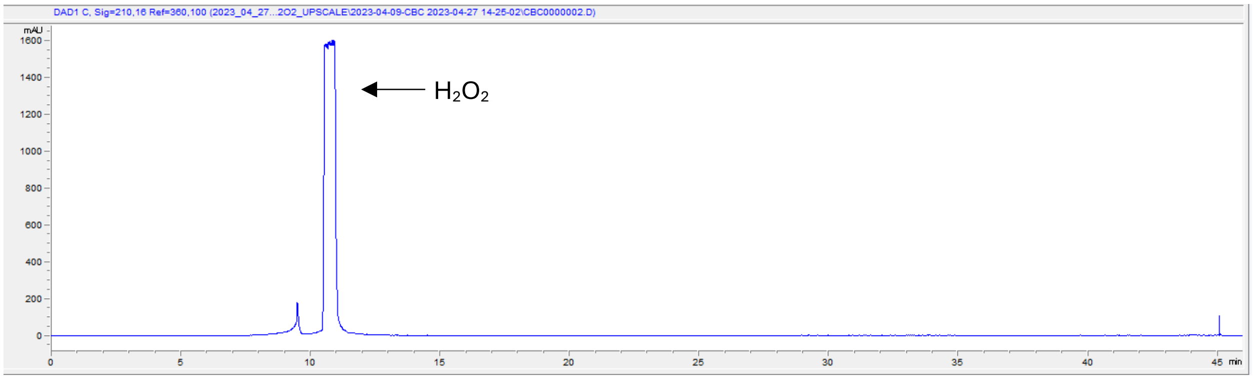

Figure S1.4.1. UV chromatogram measured at 210 nm displaying a representative control of acetonitrile incubated with H_2O_2.

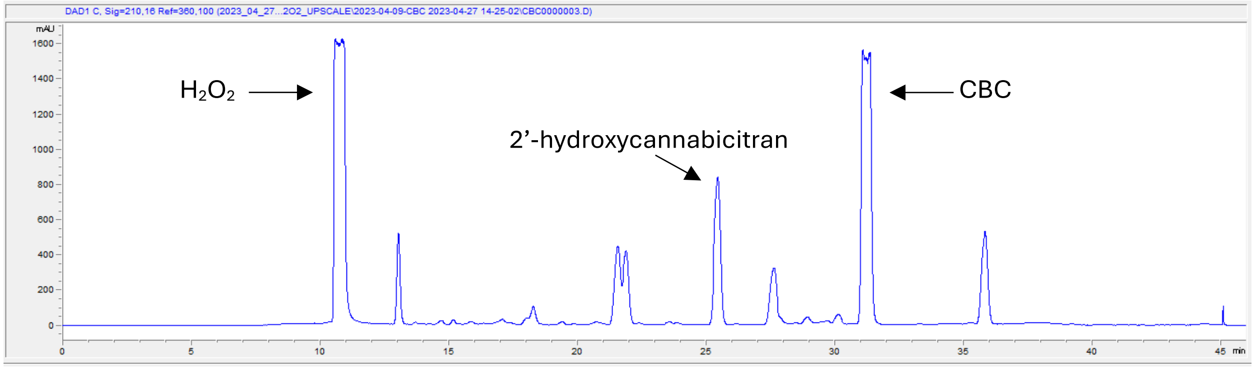

Figure S1.4.2. UV chromatogram measured at 210 nm displaying a representative sample of CBC incubated with H_2O_2. Peaks representing the synthetically generated major CBC metabolite (later identified to be 2’-hydroxycannabicitran), CBC, and H_2O_2 are labeled in the chromatogram.

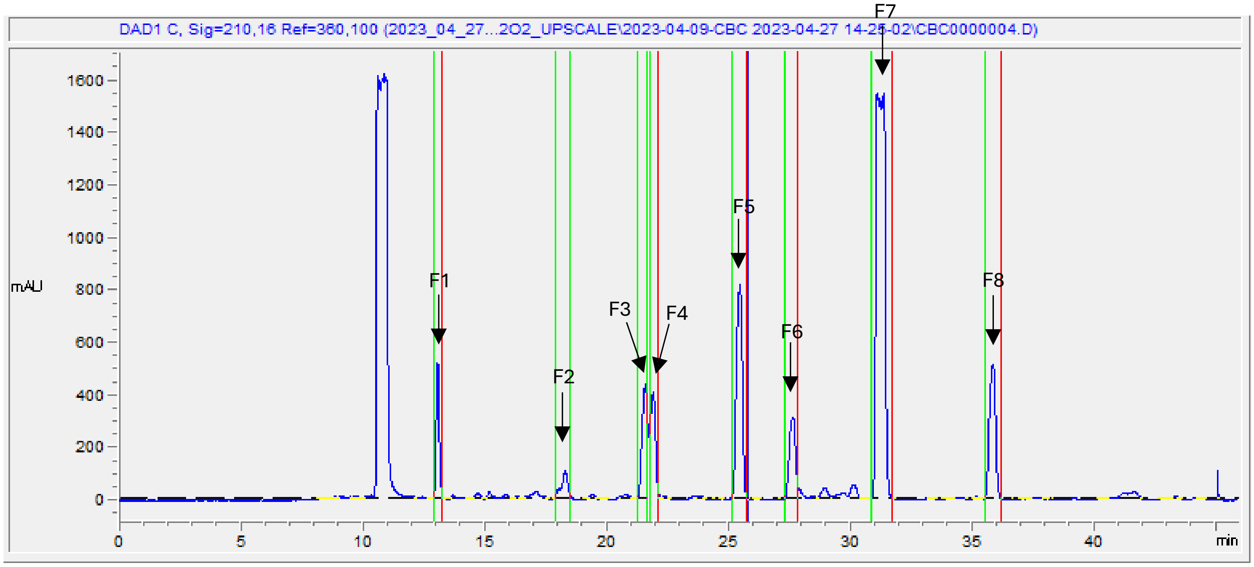

Figure S1.4.3. UV chromatogram measured at 210 nm displaying 8 fractions collected following CBC incubated with H_2O_2. F5 represents 2'-hydroxycannabicitran and F7 represents CBC.

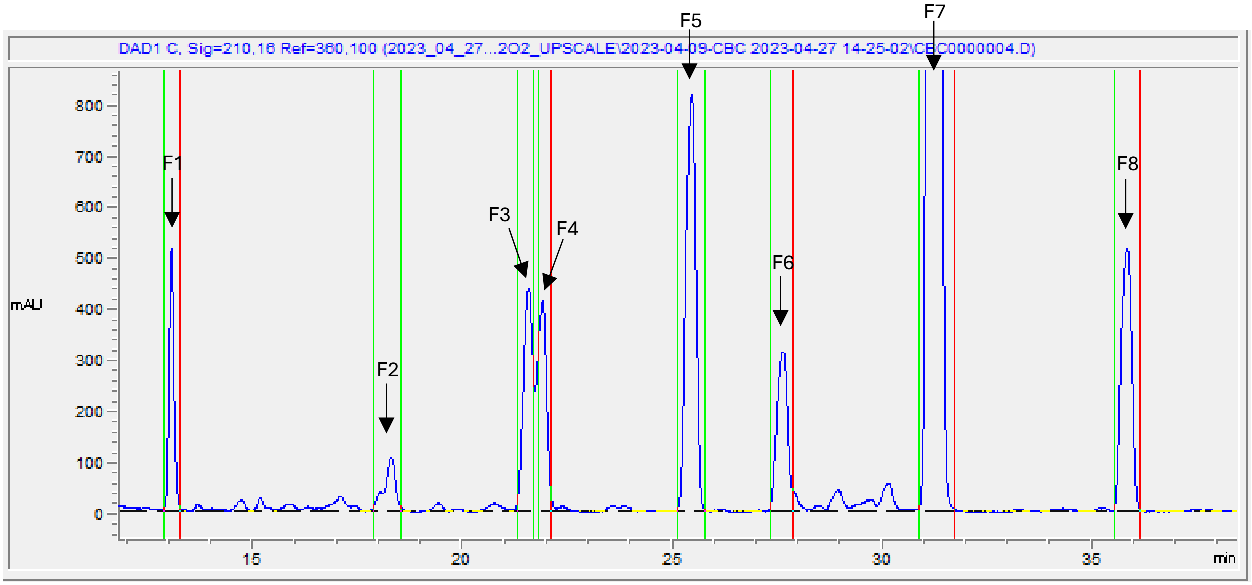

Figure S1.4.4. Zoomed-in UV chromatogram measured at 210 nm displaying 8 fractions collected following CBC incubated with H_2O_2. F5 represents 2'-hydroxycannabicitran and F7 represents CBC.

Table S1.4.1. Fractions collected from CBC H_2O_2 incubation. Fraction 5 is highlighted and represents the synthesized major CBC metabolite later identified as 2'-hydroxycannabicitran.

Fraction #	Time		Timeslices (min)	Flow rate (mL/min)	# of injections	volume collected (mL)
	On	Off				
1	12.9	13.3	0.4	3.5	33	46.2
2	17.9	18.5	0.7	3.5	33	75.1
3	21.3	21.7	0.4	3.5	33	46.2
4	21.8	22.1	0.4	3.5	33	40.4
5	25.1	25.8	0.7	3.5	33	75.1
6	27.3	27.9	0.6	3.5	33	63.5
7	30.9	31.7	0.9	3.5	33	98.2
8	35.5	36.2	0.7	3.5	33	75.1

S1.5

CBC Major Metabolite Identified via GC-MS/MS

We analyzed the complex mixture of CBC incubated with HLM as well as appropriate control samples. Included was fraction 30 of the isolated CBC HLM after the incubation was upscaled, corresponding to later identified 2'-hydroxycannabicitran.

We also analyzed isolated synthetic fractions and compared the retention times and mass spectra/fragmentation patterns to CBC metabolites generated via HLMs. Only fraction 5 from the synthetic generation corresponded to metabolites generated with HLMs (analyzed via GC-MS/MS, data from other fractions is not shown).

Please see supplementary Figure S1.5.1 for a modified description of the chromenyl ion, Figure S1.5.2 for an overlay of CBC incubated with HLM and appropriate controls, Figure S1.5.3 for a total ion chromatogram and mass spectra highlighting 2'-hydroxycannabicitran isolated from the upscaled incubation of CBC with HLM, S1.5.4 comparing the mass spectra of 2'-hydroxycannabicitran to a coeluting peak, Figure S1.5.5 for proposed 2'-hydroxycannabicitran fragments, Table S1.5.1 for major fragments of 2'-hydroxycannabicitran ranked by relative intensity, Figure S1.5.6 displays a proposed reaction scheme for the major fragment of 2'-hydroxycannabicitran, Figures S1.5.7-S1.5.9 display various chromatographic overlays and mass spectra comparing the upscaled CBC HLM mixture, the fraction F30 isolated from the upscaled incubation of CBC with HLM, and the isolated F5 fraction after the synthetic generation of 2'-hydroxycannabicitran. Table S1.5.2 displays a side-by-side comparison of the major fragments from the HLM generated (F30) and synthetically generated (F5) 2'-hydroxycannabicitran. Overall, these comparisons indicated that the major CBC metabolite generated by HLM and the synthesized compound in fraction F5 were identical.

Methods

Samples were derivatized and analyzed via GC-MS/MS based upon previous literature [32, 33] Briefly, 100 µL of sample was dried under nitrogen flow, and derivatized with 50 µL of ethyl acetate and 50 µL of BSTFA 1% TMCS, the vial was heated at 70 °C (Isotemp Hot Plate Stirrer, Thermo Fisher Scientific, Waltham, MA, USA) for 60 minutes at 150 rpm [38]. Samples were cooled, transferred to autosampler vials and immediately analyzed via GC-MS/MS.

To identify the CBC metabolite fragmentation pattern, we used a GC-MS approach following derivatization with BSTFA with 1% TMCS. The system consisted of an AOC-6000 autosampler, a GC-2010 Plus and a TQ8050 GC-MS/MS (all Shimadzu Corporation, Kyoto, Japan). The GC parameters were as follows: injection mode split 1 to 5, carrier gas helium, flow control mode linear velocity, pressure 75.2 pKa, total flow 9.8 mL/min, column flow 1.3 mL/min, linear velocity 41.4 cm/sec, purge flow 2.0 mL/min, injection temperature 265.0 °C. Column temperature gradient was: 50.0 °C ramped to 230.0 °C at 25 °C/min, 230.0 °C ramped to 258.0 °C at 5 °C /min, and 285.0 °C ramped to 300.0 °C at 10.0 °C /min. The column was DB-5MS, 30 m, 0.25 um x 0.25 µm. The total program runtime was 25.7 minutes.

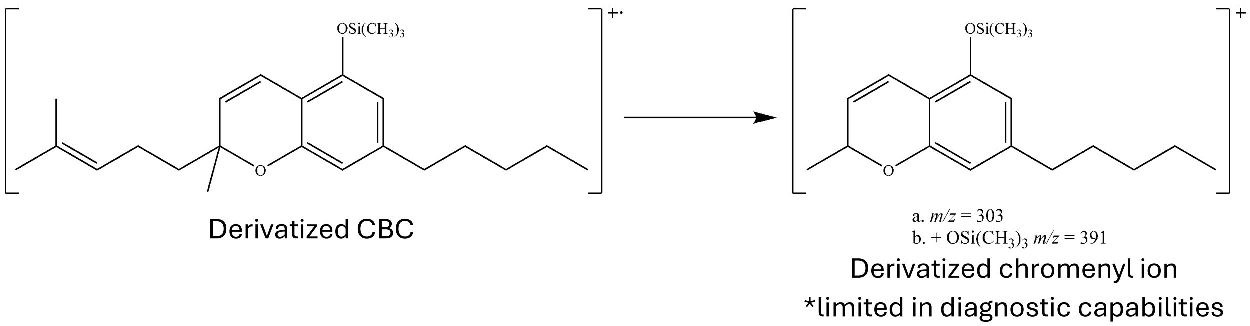

Figure S1.5.1. CBC formation of the chromenyl ion. Previously described by Harvey and Brown [32,33].

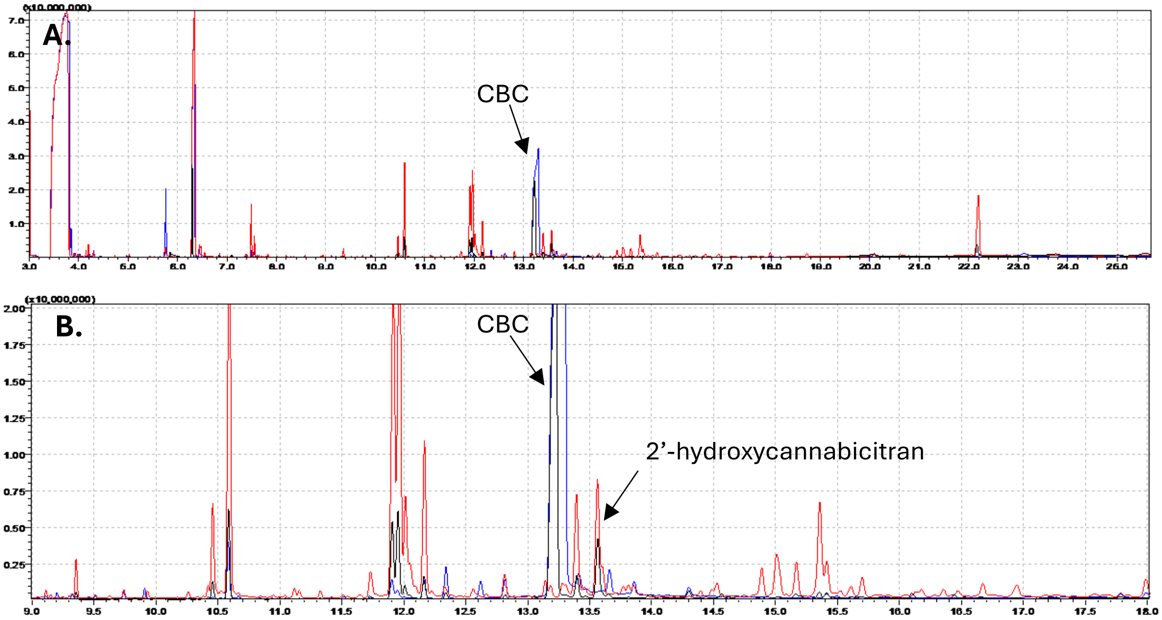

Figure S1.5.2. Overlay of CBC incubated with HLMs (50 µg/mL CBC with 0.5 mg/mL HLMs for 40 min) and appropriate controls analyzed via GC-MS/MS. (A) displays the full total ion chromatogram while (B) displays a zoomed-in area. The black tracer is CBC incubated with HLMs with a 1:100 dilution, the red tracer is a control without CBC (+ 0.5 mg/mL HLM and NADPH-generating system), incubated for 40 min, and the blue tracer is a control without HLM (+ 50 µg/mL CBC and NADPH-generating system, incubation for 40 min).

The control samples are 5x more concentrated than the 1:100 dilution of the CBC HLM sample. Overlay of 2'-hydroxycannabicitran and the background control sample can be seen in Figure S1.5.4.

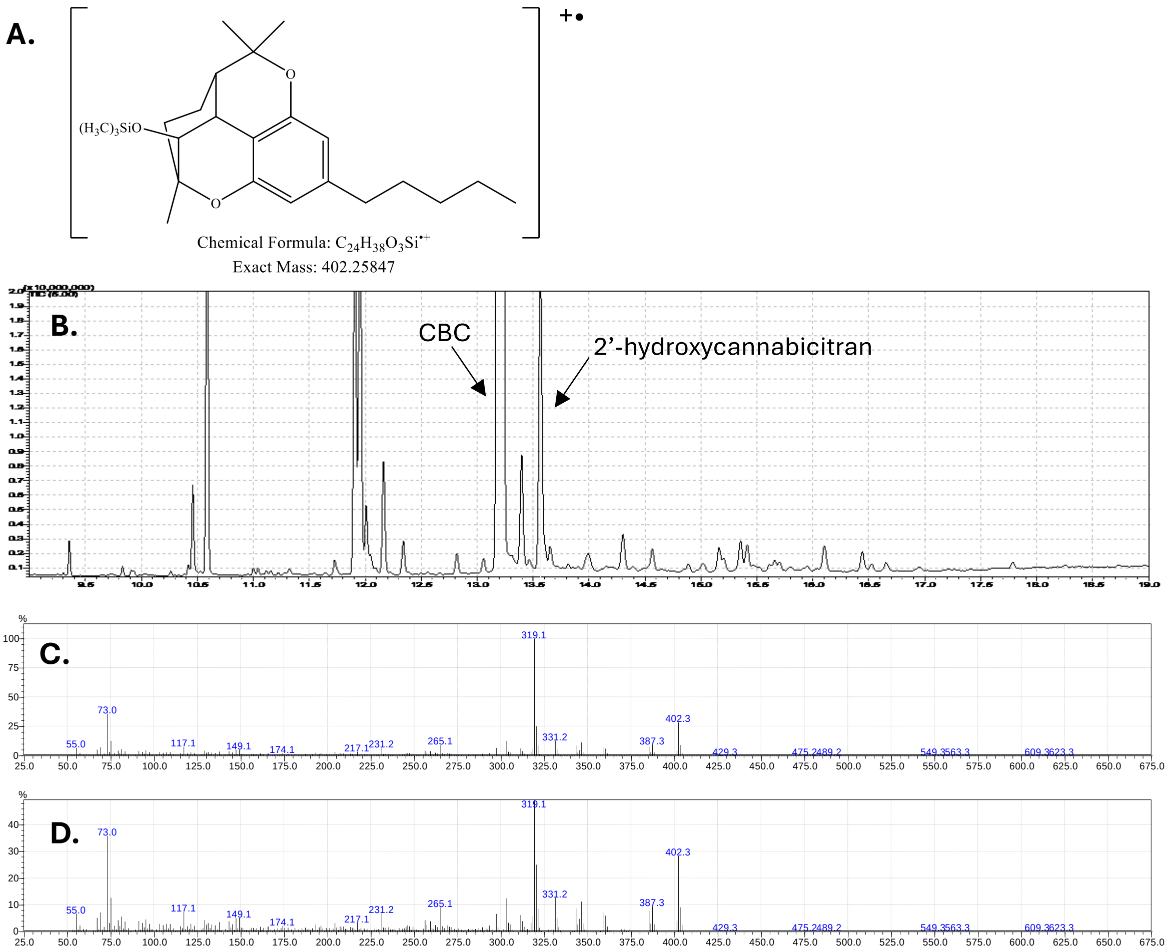

Figure S1.5.3. Derivatized 2’-hydroxycannabicitran in an upscaled incubation of CBC with HLM (50 μg/mL CBC with 0.5 mg/mL HLMs for 40 min). (A) structure of derivatized 2’-hydroxycannabicitran, (B) zoomed-in total ion chromatogram, cursor shows where mass spectrum originates from, (C) mass spectrum of 2’-hydroxycannabicitran, (D) zoomed-in mass spectrum of 2’-hydroxycannabicitran.

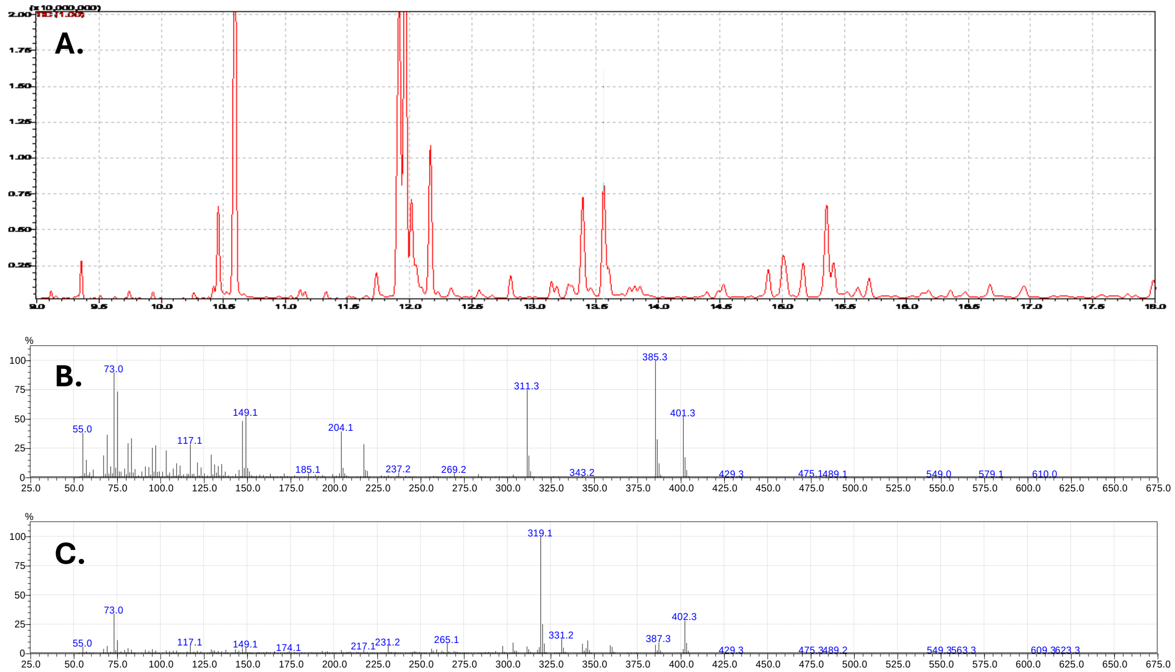

Figure S1.5.4. Comparison of coeluting peak in a control without CBC (+ 0.5 mg/mL HLM and NADPH-generating system) and 2'-hydroxycannabicitran. (A) displays the total ion chromatogram of the control sample without CBC (+ 0.5 mg/mL HLM and NADPH-generating system) the cursor indicates where the mass spectrum originates, (B) mass spectrum of the control sample without CBC (+ 0.5 mg/mL HLM and NADPH-generating system), and (C) mass spectrum of CBC incubated with HLM (50 µg/mL CBC with 0.5 mg/mL HLMs for 40 min) 1:100 dilution at the same retention time.

Despite a peak representing HLM/NADPH-generating system background overlaps with 2'-hydroxycannabicitran, they contain differing fragmentation patterns. In the CBC HLM sample (50 µg/mL CBC with 0.5 mg/mL HLMs for 40 min) we can expect this background peak to minimally contribute to the relative abundance of ions based on dilution factors applied following analysis. However, we were careful not to over interpret the spectra due to this overlapping peak with the major metabolite of CBC.

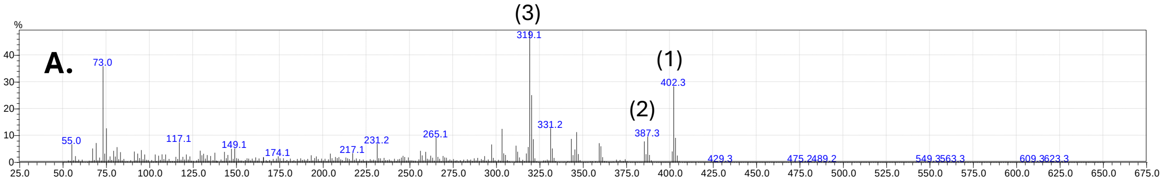

B.

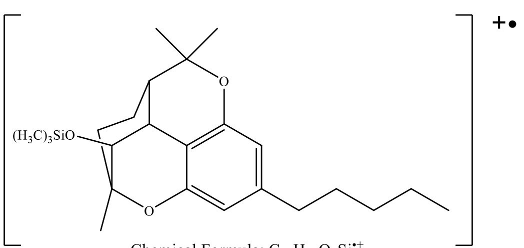

(1) Chemical Formula: $C_{24}H_{38}O_3Si^{\bullet+}$
Exact Mass: 402.25847

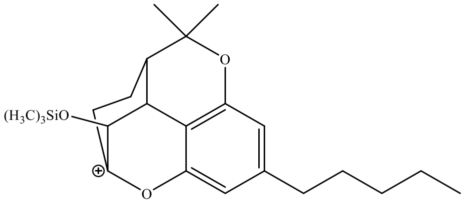

(2) Chemical Formula: $C_{23}H_{35}O_3Si^+$
Exact Mass: 387.23500

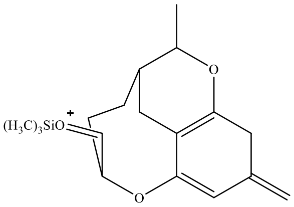

(3a) Chemical Formula: $C_{18}H_{27}O_3Si^+$
Exact Mass: 319.17240

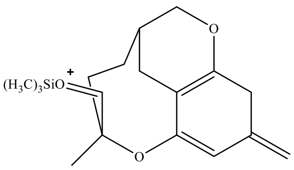

(3b) Chemical Formula: $C_{18}H_{27}O_3Si^+$
Exact Mass: 319.17240

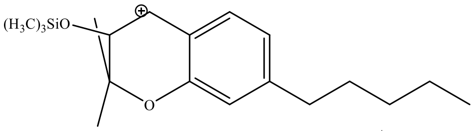

(3c) Chemical Formula: $C_{19}H_{31}O_2Si^+$
Exact Mass: 319.20878

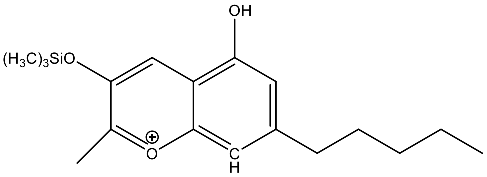

(3d) Chemical Formula: $C_{18}H_{27}O_3Si^+$
Exact Mass: 319.17240

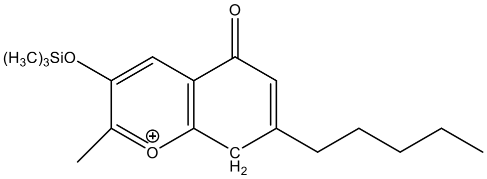

(3e) Chemical Formula: $C_{18}H_{27}O_3Si^+$
Exact Mass: 319.17240

Figure S1.5.5. Zoomed-in mass spectrum of derivatized 2'-hydroxycannabicitran from CBC incubated with HLMs (50 µg/mL CBC with 0.5 mg/mL HLMs for 40 min) (A) and proposed fragments (B) analyzed via GC-MS/MS.

Table S1.5.1. Major fragments of 2'-hydroxycannabicitran ranked by relative intensity.

m/z	Absolute Intensity	Relative Intensity
319.15	199623	100.00
402.25	56642	28.37
331.20	25525	12.79
346.20	22524	11.28
387.25	19199	9.62
265.15	18547	9.29
201.10	2694	1.35
191.10	2628	1.32
275.15	2473	1.24

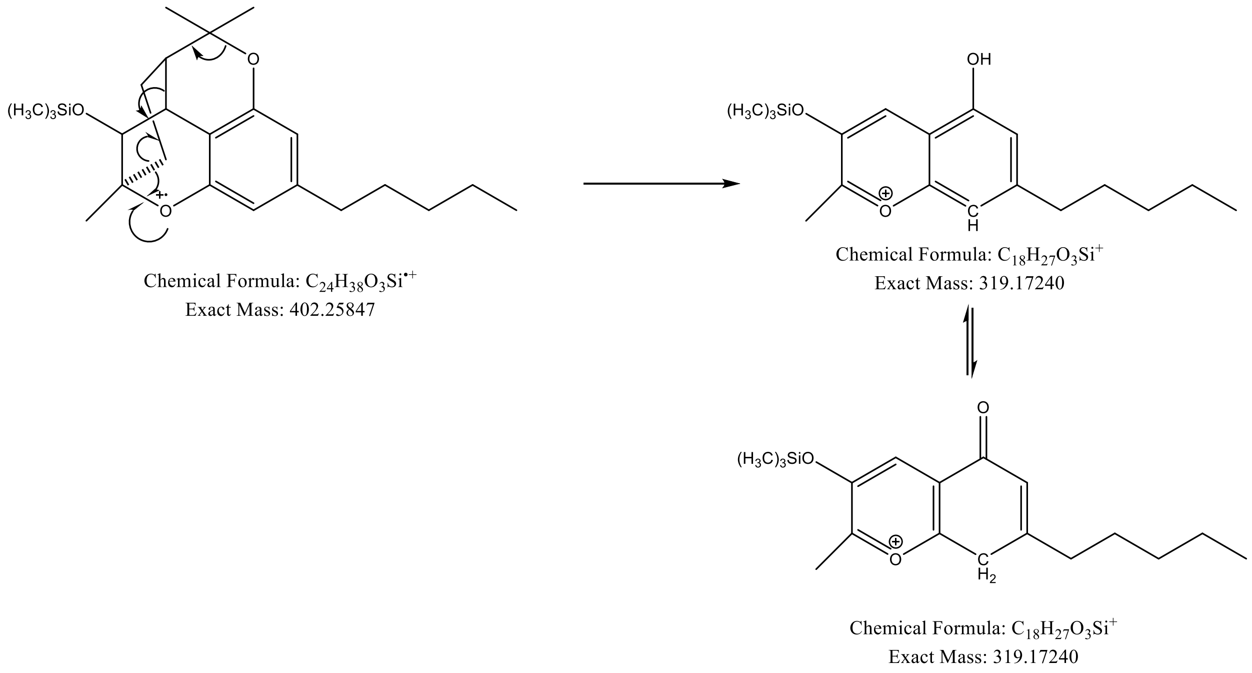

Figure S1.5.6. Proposed reaction scheme of derivatized 2'-hydroxycannabicitran generating the *m/z* = 319 fragment.

It is shown in Figures S1.5.5 and S1.5.6 that uncertainty with the GC-MS/MS fragments of the major oxidized CBC metabolite remains. Accordingly, we could not determine the structure of the major oxidized CBC metabolite based on GC-MS/MS fragments with sufficient confidence. Therefore, we synthetically generated 2'-hydroxycannabicitran and determined its structure via NMR. It is shown in Figure S1.5.8, Figure S1.5.9, Table S1.5.2, and Figures S2.1.14-S2.1.18 that the synthetically generated metabolite matched that of the major CBC metabolite generated with HLMs.

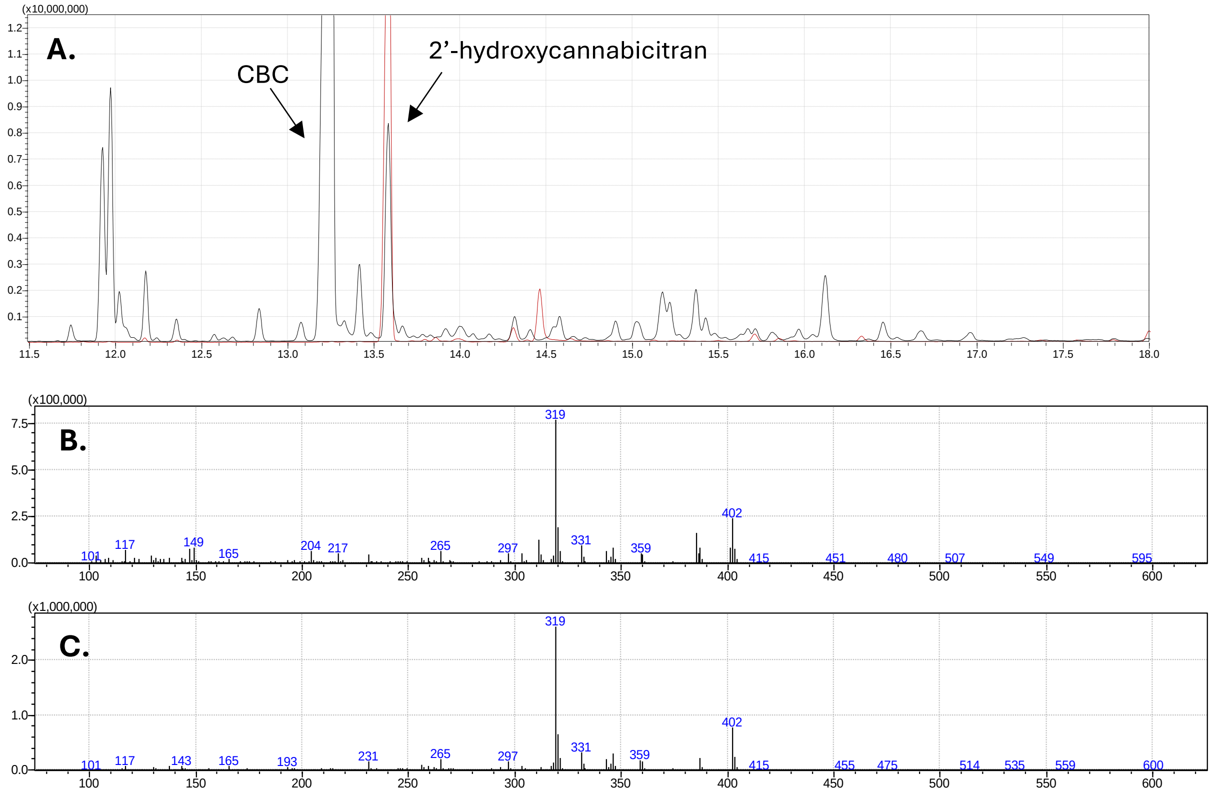

Figure S1.5.7. Overlay of total ion chromatograms of CBC incubated with HLMs (50 μg/mL CBC with 0.5 mg/mL HLMs for 40 min) and isolated fraction (F30) from an upscaled incubation of CBC with HLM (A). The black tracer displays the upscaled incubation of CBC with the HLM mixture and the red tracer is isolated F30 from the upscale. (B) mass spectrum of 2’-hydroxycannabicitran from the CBC HLM mixture and (C) mass spectrum of 2’-hydroxycannabicitran from the isolated F30 fraction.

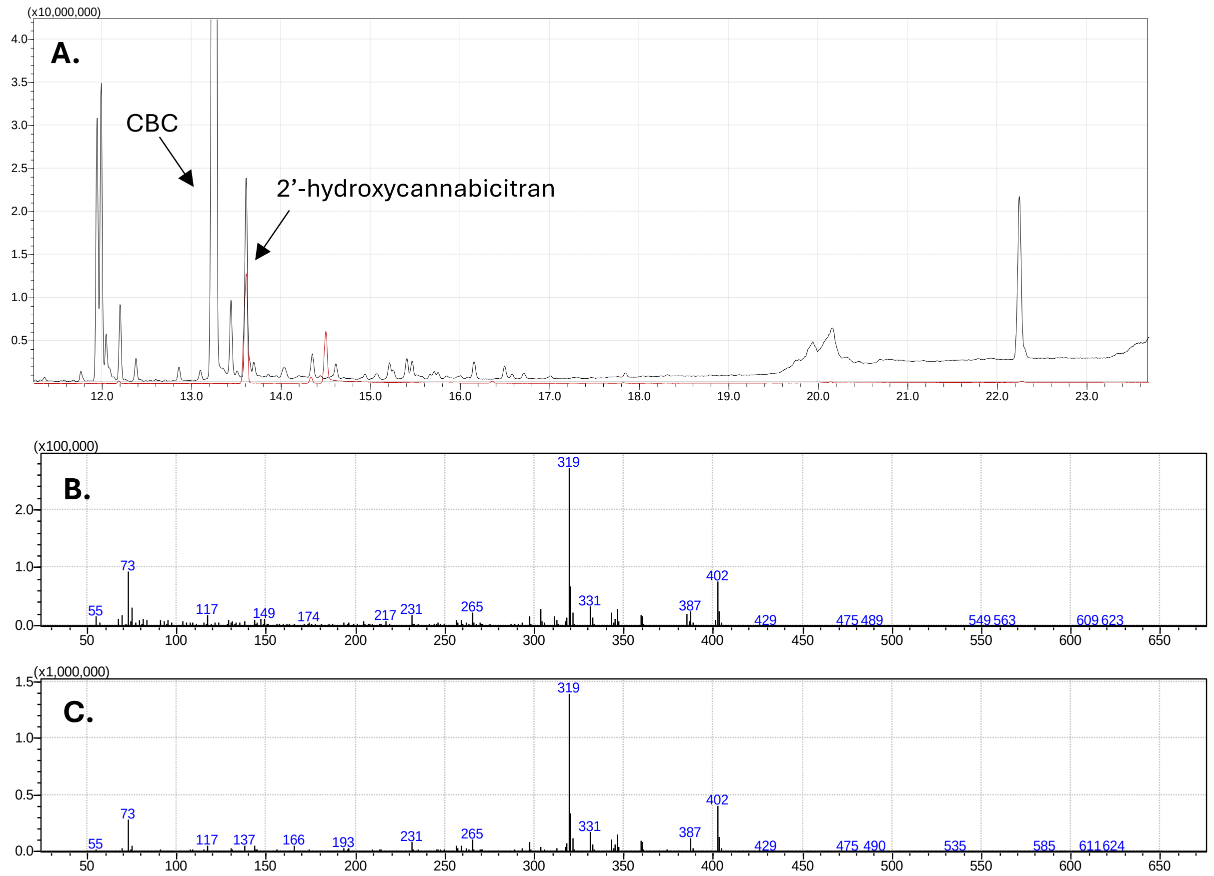

Figure S1.5.8. Overlay of total ion chromatograms of CBC incubated with HLM (50 µg/mL CBC with 0.5 mg/mL HLMs for 40 min) and isolated fraction (F5) from CBC incubated with H_2O_2 (A). The black tracer displays the upscaled CBC incubation with HLM and the red tracer is isolated F5 from the synthetic generation of 2'-hydroxycannabicitran. (B) mass spectrum of 2'-hydroxycannabicitran from the CBC HLM mixture and (C) mass spectrum of synthetically generated 2'-hydroxycannabicitran from the isolated F5 fraction.

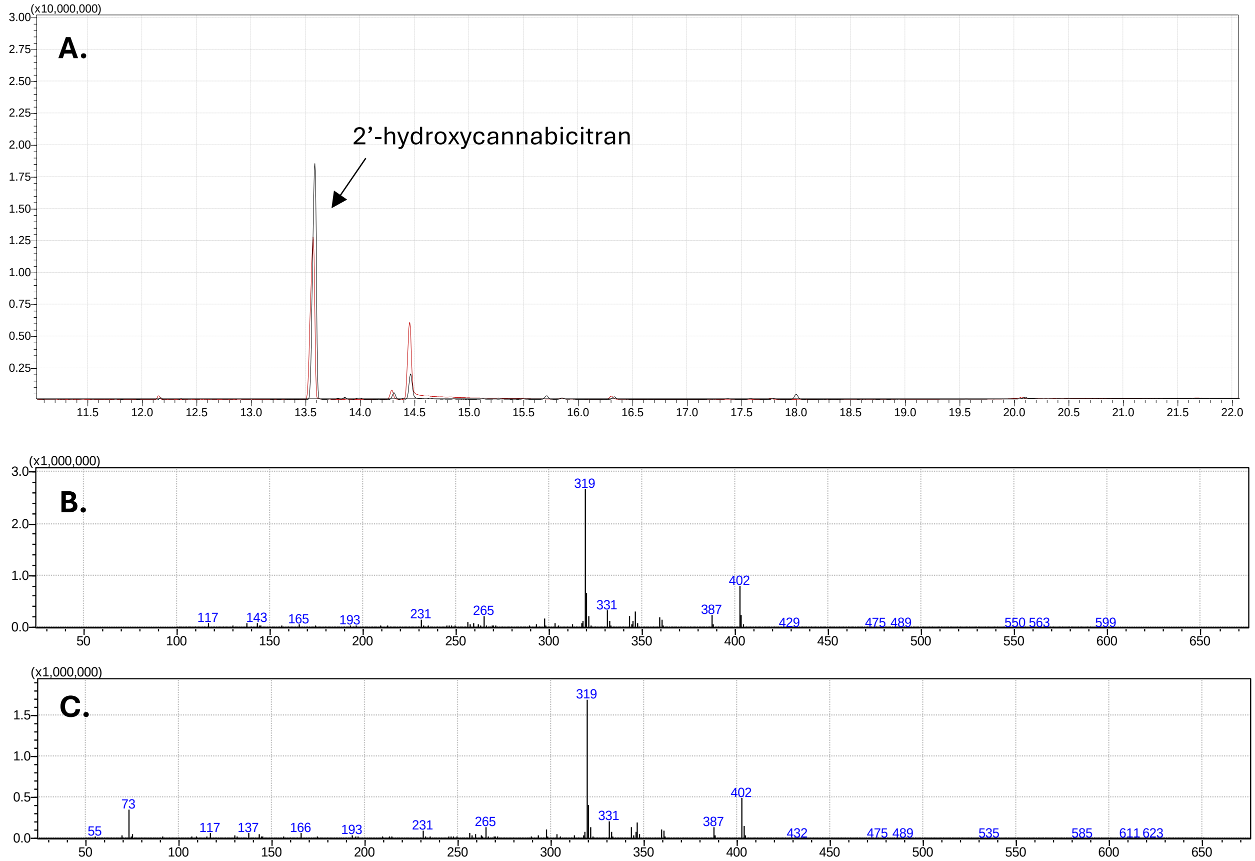

Figure S1.5.9. Overlay of total ion chromatograms of isolated fraction (F30) of CBC incubated with HLM and isolated fraction (F5) from CBC incubated with H_2O_2 (A). The black tracer displays the isolated fraction (F30) of CBC incubated with HLM (50 µg/mL CBC with 0.5 mg/mL HLMs for 40 min) upscale and the red tracer is isolated F5 from the synthetic generation of 2'-hydroxycannabicitran. (B) mass spectrum of 2'-hydroxycannabicitran from the isolated F30 fraction of CBC incubated with HLM upscale and (C) mass spectrum of synthetically generated 2'-hydroxycannabicitran from the isolated F5 fraction.

Top fragments ranked by relative intensity from isolated F30 and F5 fractions are shown in Table S1.5.2.

Table S1.5.2. Top fragments ranked by relative intensity from isolated F30 and F5 fractions.

F30 from CBC HLM

m/z	Absolute Intensity	Relative Intensity
319.15	2701469	100.00
402.20	810722	30.01
320.15	678850	25.13
331.15	333962	12.36
346.15	313065	11.59
403.20	255605	9.46
387.20	245415	9.08
321.15	233287	8.64
343.10	224005	8.29
265.10	220612	8.17
359.15	194727	7.21
297.15	175148	6.48
360.15	165789	6.14
231.10	161738	5.99
318.15	146521	5.42
332.15	136697	5.06
345.15	131731	4.88

F5 from Synthetic Generation

m/z	Absolute Intensity	Relative Intensity
319.15	1701945	100.00
402.25	497948	29.26
320.15	413953	24.32
331.20	208617	12.26
346.20	194191	11.41
403.25	156324	9.19
387.25	148557	8.73
321.15	143966	8.46
343.20	139624	8.20
265.15	138982	8.17
359.20	117613	6.91
297.20	109007	6.40
231.10	105116	6.18
360.20	100459	5.90
318.25	91845	5.40
332.20	83108	4.88
345.20	79562	4.67
73.05	349340	20.53

The overlay of synthetically generated and HLM generated 2'-hydroxycannabicitran shows retention time alignment. Moreover, the top 17 fragments ranked by relative intensity of both samples are nearly identical. An exception is the flipped ranked order of *m/z* = 360.15 and *m/z* = 231.10 in the samples, as well as *m/z* = 73.05 in F5, representing the loss of -$Si(CH_3)_3$.

In summary, the data and comparisons presented above indicated that the synthetically generated compound in F5 and the major metabolite generated after CBC incubation with HLMs were identical.

References

32. Harvey DJ, Brown NK. A Method for the Structural Determination of Cannabichromene Metabolites by Mass Spectrometry. Rapid Communications in Mass Spectrometry. 1990;4(4):135-6.

33. Harvey DJ, Brown NK. Identification of Cannabichromene Metabolites by Mass Spectrometry: Identification of Eight New Dihydroxy Metabolites in the Rabbit. Biological Mass Spectrometry. 1991;20:275-85.

38. Sanderson J, Westland J. Quantitation of Cannabinoids in Hemp Flower by Derivatization GC/MS: Agilent Technologies; 2021 [cited 2022 November 11]. Available from: https://www.agilent.com/cs/library/applications/application-hemp-cannabis-thc-gcms-5994-2757en-agilent.pdf.
