# Supplementary material for: Identification and Characterization of Cannabichromene’s Major Metabolite Following Incubation with Human Liver Microsomes
Source: Metabolites. 2024 Jun 13;14(6):329. doi: 10.3390/metabo14060329 (PMC11206029; doi:10.3390/metabo14060329)
Supplement: Supplementary file 1 [file metabolites-14-00329-s001.zip › CBC_Metabolite_Supplementary_Materials_S2.pdf]

Supplemental Materials S2

Identification and Characterization of Cannabichromene's Major Metabolite Following Incubation with Human Liver Microsomes

Alexandra M. Ward[1], Touraj Shokati[2], Jost Klawitter[2], Jelena Klawitter[2], Vu Nguyen[1], Laura Kozell[3, 4, 5], Atheir I. Abbas[3, 4, 5], David Jones[6], and Uwe Christians[2,*]

[1]*Department of Pharmaceutical Sciences, Skaggs School of Pharmacy and Pharmaceutical Sciences, University of Colorado Anschutz Medical Campus, Aurora, CO, USA;* alexandra.ward@cuanschutz.edu, vu.t.nguyen@cuanschutz.edu.

[2]*iC42 Clinical Research and Development, Department of Anesthesiology, School of Medicine, University of Colorado Anschutz Medical Campus, Aurora, CO, USA;* touraj.shokati@cuanschutz.edu, jost.klawitter@cuanschutz.edu, jelena.klawitter@cuanschutz.edu, uwe.christians@cuanschutz.edu.

[3]*Department of Behavioral Neuroscience, Oregon Health & Science University, Portland, OR, USA;* abbasat@ohsu.edu, kozellla@ohsu.edu.

[4]*Department of Psychiatry, Oregon Health & Science University, Portland, OR, USA.*

[5]*Veterans Affairs Portland Health Care System, Portland, OR, USA.*

[6]*Department of Pharmacology, School of Medicine, University of Colorado Anschutz Medical Campus, Aurora, CO, USA;* david.jones@cuanschutz.edu.

*Correspondence: uwe.christians@cuanschutz.edu

Table of Contents Supplemental Materials S2

S2.1
Recapitulation and Comparison to Roy et al. Page 3

References Page 34

S2.1

Recapitulation and Comparison to Roy et al.

During the preparation of this manuscript, another article assessing the drug metabolism of CBC was published by Roy et al. They described 4 CBC metabolites: 8'-hydroxy-CBC, 6', 7'-epoxy-CBC,1''-hydroxy-CBC, and 6', 7'-dihydroxy-CBC [35].

We sought to recapitulate this study to determine if we generated the same or different metabolites. We segmented our experimental methodology by incubation and extraction parameters (for details, please see below) to determine if these differences explained the different CBC metabolites identified. We did not recapitulate the analytical methodology of Roy et al. as our method offered better chromatographic resolution.

We determined that the incubation and extraction parameters tested did not have an impact on the phenotypic metabolic profile and the parameters described by Roy et al. and also yielded 2'-hydroxycannabicitran as the major metabolite of CBC. We were able to confirm the presence of 6', 7'-epoxy-CBC based on a specific MS/MS fragment resulting from α-cleavage fragment adjacent to the oxidation position. Other metabolites described by Roy et al. could not be verified due to lack of specific fragments and/or verified synthetic standards.

Please see supplementary Figures S.2.1.1 and S2.1.2 for representative extracted ion chromatograms and mass spectra of CBC and 2'-hydroxycannabicitran in neat solution, respectively. Figures S2.1.3-S2.1.7 detail the results and controls from the incubation and extraction of Roy et al. and Figures S2.1.8-S2.1.12 detail the results and controls from the incubation and extraction of the study herein. Figure S2.1.13 shows the overlay of CBC from the study herein and Roy et al. Figures S2.1.14-S2.1.18 show an overlay focused on 2'-hydroxycannabicitran between the study herein, Roy et al., and synthetically generated metabolite.

Figures S2.1.19-S2.1.21 and S2.1.24 show the remaining unidentified metabolites (1-3, 5) compared between Roy et al. and the study herein. Figures S2.1.22 and S2.1.23 show unidentified metabolite 4 as well as 6',7'-epoxy-CBC compared between Roy et al. and the study herein.

A CBC fragmentation pattern is described in Figures S2.1.25 and S2.1.26, corresponding to Δppm listed in Table 2.1.1. A 6',7'-epoxy-CBC fragmentation pattern is described in Figures S2.1.27 and S2.1.28, corresponding to Δppm of theoretical and measured exact mass of the fragments listed in Table 2.1.2.

Materials and Methods

CBC was purchased from Cayman Chemical (Ann Arbor, MI, USA), NADPH was purchased from Sigma-Millipore (St. Louis, MO, USA), and HLM was purchased from Xenotech (Kansas City, KS, USA), pool of 50 individuals H0620/Lot #1810003.

The experimental methodologies are summarized below:

Roy et al. Incubation

- 10 min incubation of CBC (12.5 µg/mL) + HLM (0.36 mg/mL)
- Add NADPH (final conc. 1 mM)
- 30 min incubation

Roy et al. Extraction “Hex:EtOAc”

- Quench with 80:20 Hex:EtOAc (500 µL)
- Centrifuge 900 x g, 5 min
- Transfer organic layer to LC vial
- 2x 80:20 Hex:EtOAc (500 µL), centrifuge, transfer
- Dry under N_2
- Reconstitute in 100 µL 95% ethanol
- Analyze

Study Herein Incubation

- 10 min NADPH-generating system incubation (0.9 mM final conc, $NADP^+$)
- Add CBC (50 µg/mL) and HLM (1 mg/mL)
- 40 min incubation

Study Herein Extraction “ACN crash”

- Quench with cold ACN (500 µL)
- Centrifuge 685 x g, 10 min
- Transfer 550 µL supernatant to LC vial
- Analyze

We generated several sets of samples (n=3 for all):

Incubation as described herein with acetonitrile crash

- CBC + HLM + NADPH generating system
- Control without CBC (+HLM + NADPH generating system)
- Control without HLM (+CBC + NADPH generating system)
- Control without NADPH generating system (+CBC + HLM)

Incubation as described herein with Hex:EtOAc extraction

- CBC + HLM + NADPH generating system
- Control without CBC (+HLM + NADPH generating system)
- Control without HLM (+CBC + NADPH generating system)
- Control without NADPH generating system (+CBC + HLM)

Incubation conditions as described by Roy et al. with Hex:EtOAc extraction

- CBC + HLM + NADPH solution
- Control without CBC (+HLM + NADPH solution)
- Control without HLM (+CBC + NADPH solution)
- Control without NADPH solution (+CBC + HLM)

Incubation conditions as described by Roy et al. with acetonitrile crash

- CBC + HLM + NADPH solution
- Control without CBC (+HLM + NADPH solution)
- Control without HLM (+CBC + NADPH solution)
- Control without NADPH solution (+CBC + HLM)

Incubation as described herein with Hex:EtOAc extraction and incubation conditions as described by Roy et al. with acetonitrile crash yielded identical metabolites generated as the other conditions tested. Since the focus was comparing the experimental methodologies of Roy et al. and what is described by the study herein, we omitted the crossed incubation/extraction data for clarity.

We recapitulated the CBC HLM incubation experiment described by Roy et al. to compare the subsequent metabolites generated with our analytical methodology. The instrumentation for this experiment was a 1290 Infinity LC injector HTS, G1379A degasser, G1312A binary pump, G1316C column compartment (all Agilent Technologies, Santa Clara, CA, USA) equipped with a 5600+ high-resolution TOF (AB Sciex, Concord, ON, Canada). The analytical columns were 2 x Eclipse XDB-C8 5 µm, 4.6 x 250 mm in series (Agilent Technologies, Santa Clara, CA, USA). The HPLC/MS system was controlled and data was processed using Analyst TF1.8.1 software (AB Sciex, Concord, ON, Canada).

The injection volume was 25 µL with the column temperature set at 60 °C. Mobile phase A was water + 0.1% formic acid and mobile phase B was acetonitrile + 0.1% formic acid. The pump gradient was: 0.0 min, 75% B; 1.0 min, 75% B; 14.0 min, 95% B; 17.0 min, 98% B; 22.0 min, 98% B; 22.1 min, 75% B; and 30.0 min, 75% B; the flow rate was 1000 µL/min throughout the gradient. We also analyzed a representative CBC HLM sample at 30 °C and observed no change in metabolite fragmentation or elution pattern, indicating column compartment temperature did not cause additional reactivity of the measured metabolites. Data is not shown.

The mass spectra were collected in positive electrospray ionization (ESI). Three MS experiments were run: 1. TOF MS Scan from 50-600 *m/z* with an accumulation time of 0.5 sec, 2. Product Ion at 315.2 with an accumulation time of 0.1 sec, and 3. Product Ion at 331.2 with an accumulation time of 0.1 sec. The mass spectrometer parameters were as follows: ion source gas 1 50 psi, ion source gas 2 50 psi, curtain gas 50 psi, source temperature 600 °C, ion spray voltage 5000 V, declustering potential 80 V, and collision energy 25 eV.

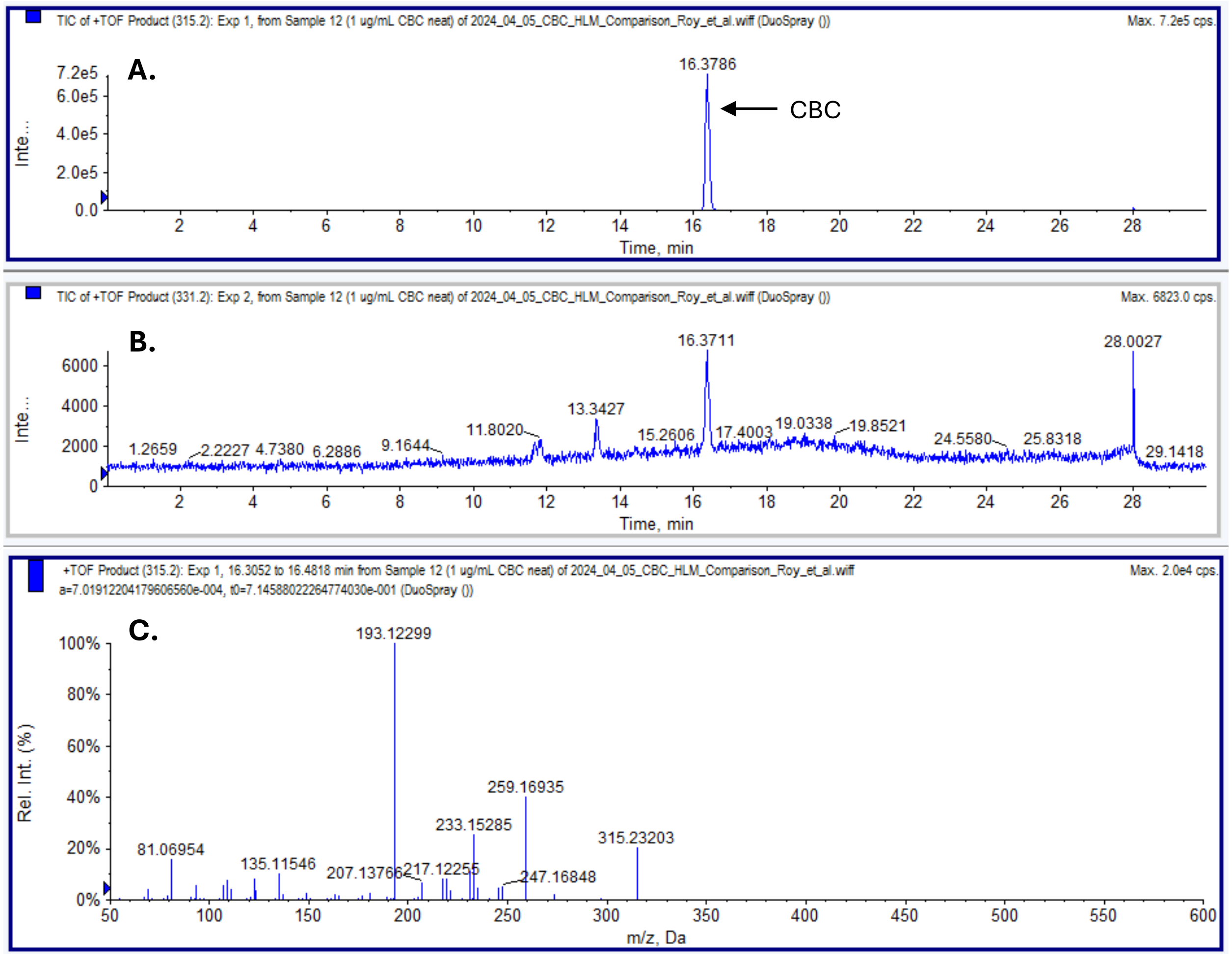

Figure S2.1.1. Representative extracted ion chromatograms and mass spectrum of CBC (1 µg/mL) in neat acetonitrile. (A) Extracted ion chromatogram of *m/z* = 315.2, (B) extracted ion chromatogram of *m/z* = 331.2, and (C) mass spectrum of CBC stemming from the primary peak in (A).

While there is a signal present in the extracted ion chromatogram of *m/z* = 331.2 with a retention time consistent with the major metabolite of CBC (Figure S2.1.2), we did not consider this as significant because of the low intensity of the signal relative to CBC at *m/z* = 315.2.

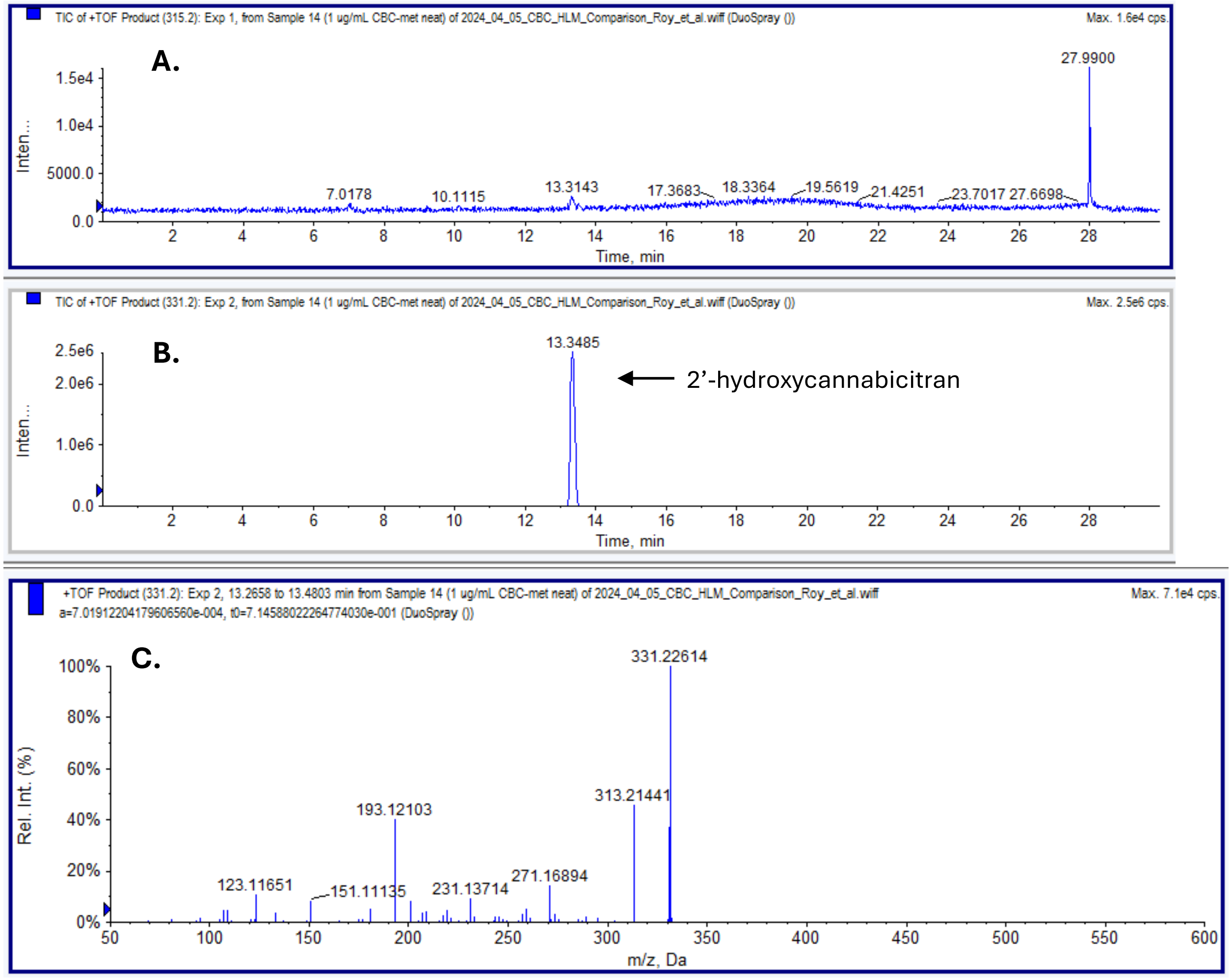

Figure S2.1.2. Representative chromatograms and mass spectrum of 2'-hydroxycannabicitran (1 µg/mL) in neat acetonitrile. 2'-hydroxycannabicitran was synthetically generated and verified via NMR spectroscopy. (A) Extracted ion chromatogram of *m/z* = 315.2, (B) extracted ion chromatogram of *m/z* = 331.2, and (C) mass spectrum of 2'-hydroxycannabicitran stemming from the primary peak in (B).

These chromatograms and mass spectra provide a reference for the expected fragmentation pattern and retention time of 2'-hydroxycannabicitran generated from incubation with HLMs.

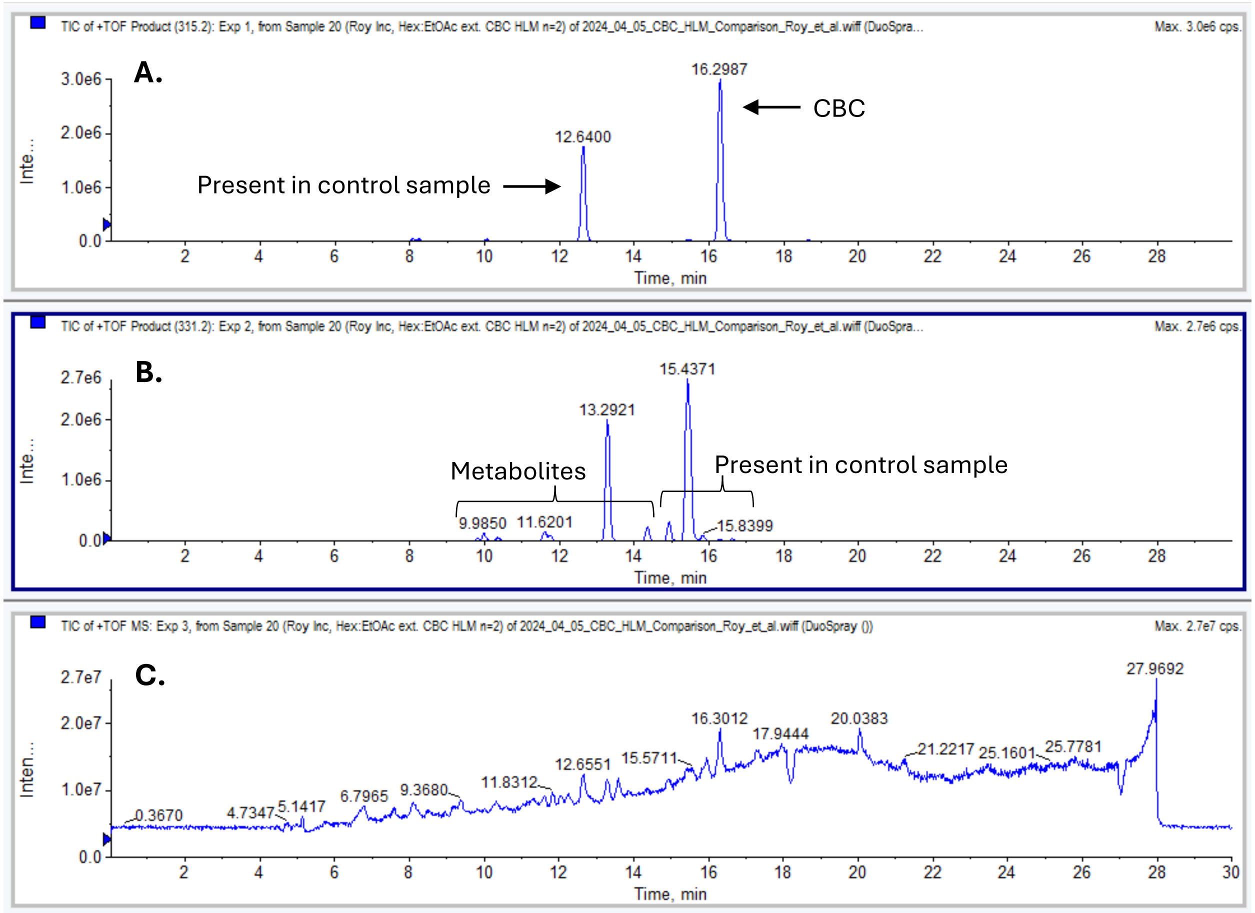

Figure S2.1.3. Representative chromatograms after incubation of CBC with HLM (+ NADPH solution) and extraction following the protocol described by Roy et al. (details on page 4). (A) Extracted ion chromatogram of *m/z* = 315.2, (B) extracted ion chromatogram of *m/z* = 331.2, and (C) total ion chromatogram from scan mode (*m/z* = 50-600).

Representative control samples are shown in Figures S2.1.4-S2.1.6.

These chromatograms display the chromatographic pattern of metabolites generated with the incubation and extraction parameters described by Roy et al. With comparison to the control samples, we could assign extraneous peaks resulting from the matrix.

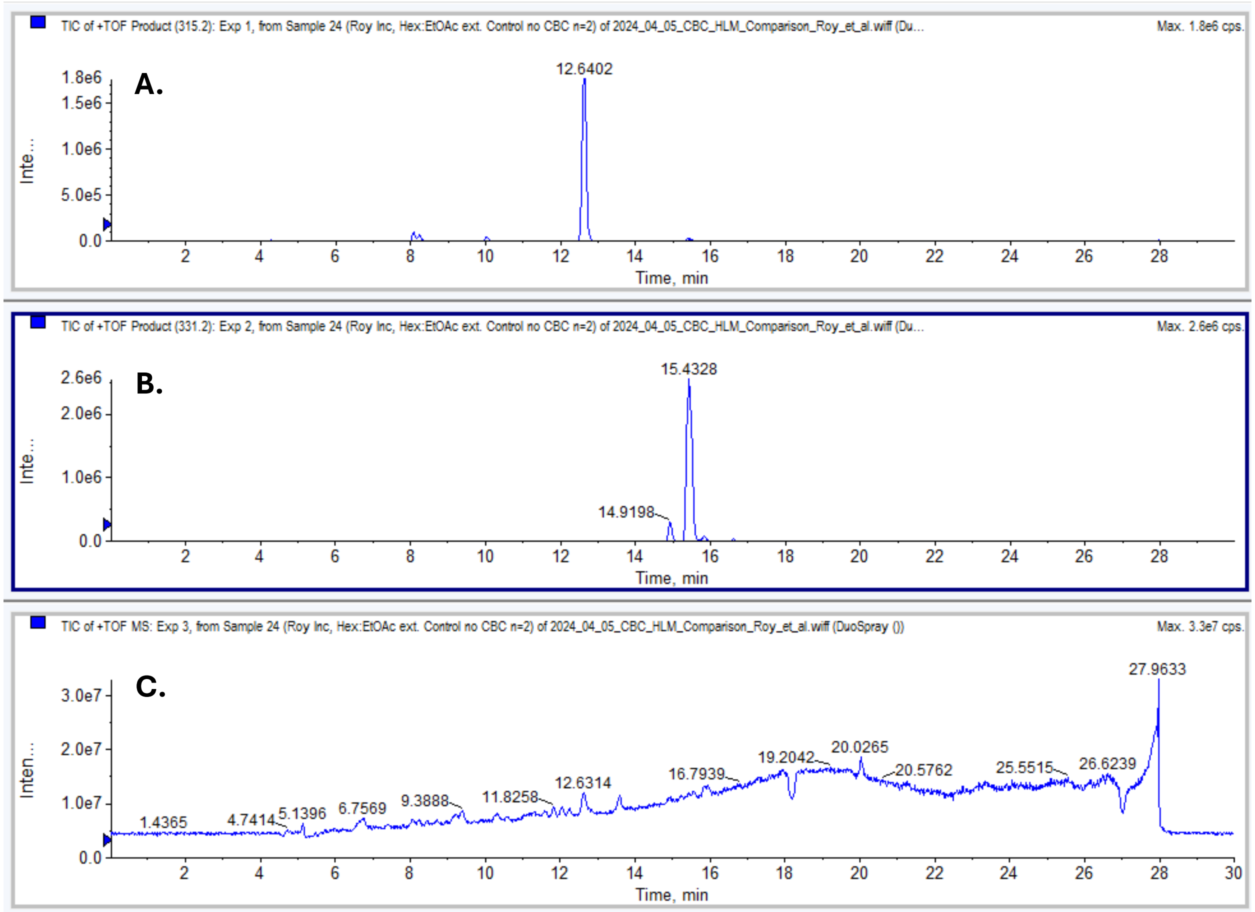

Figure S2.1.4. Representative chromatograms of a control sample (without CBC) after incubation of HLM + NADPH solution and extraction following the protocol described by Roy et al. (additional details on page 4). (A) Extracted ion chromatogram of *m/z* = 315.2, (B) extracted ion chromatogram of *m/z* = 331.2, and (C) total ion chromatogram from scan mode (*m/z* = 50-600).

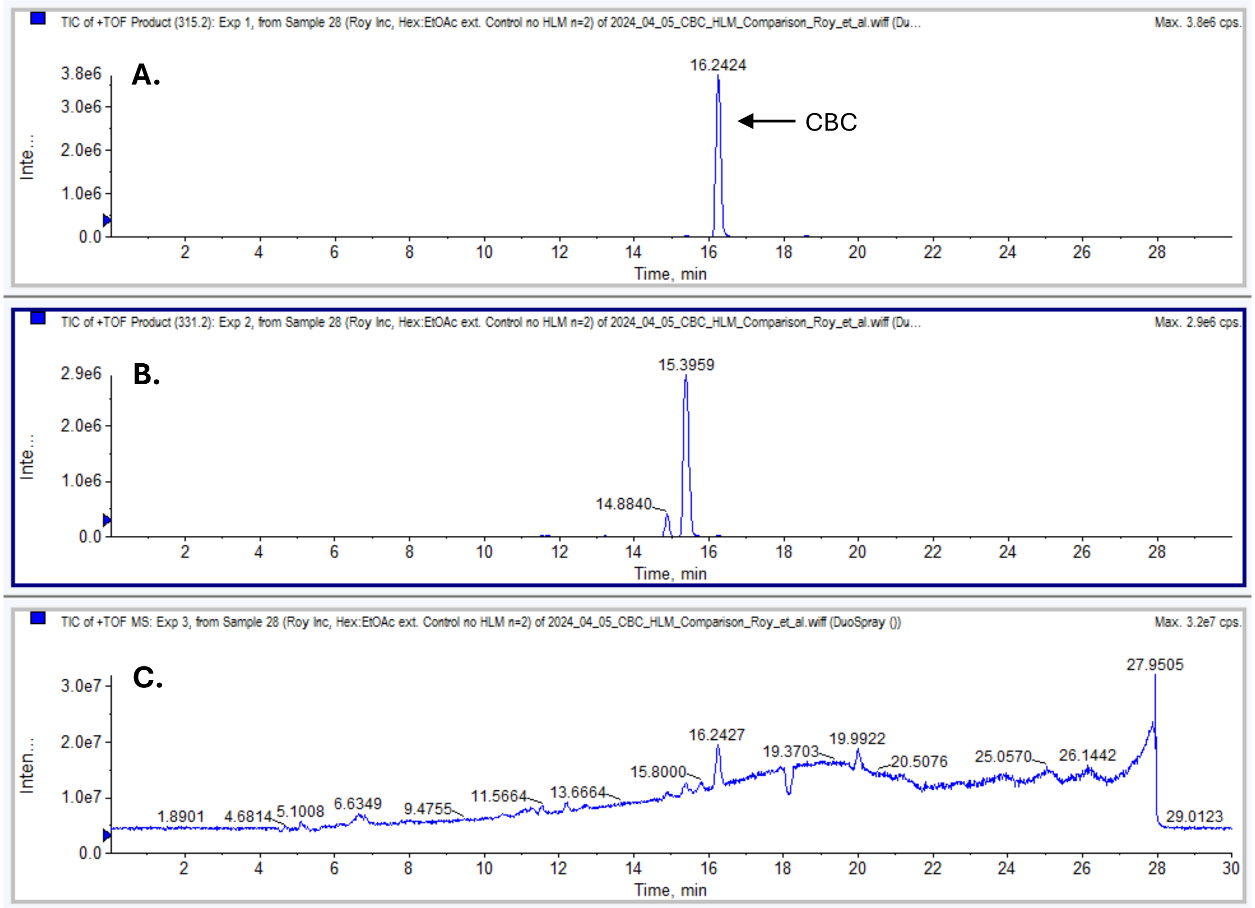

Figure S2.1.5 Representative chromatograms of a control sample (without HLM) after incubation of CBC + NADPH solution and extraction following the protocol described by Roy et al. (additional details on page 4). (A) Extracted ion chromatogram of *m/z* = 315.2, (B) extracted ion chromatogram of *m/z* = 331.2, and (C) total ion chromatogram from scan mode (*m/z* = 50-600).

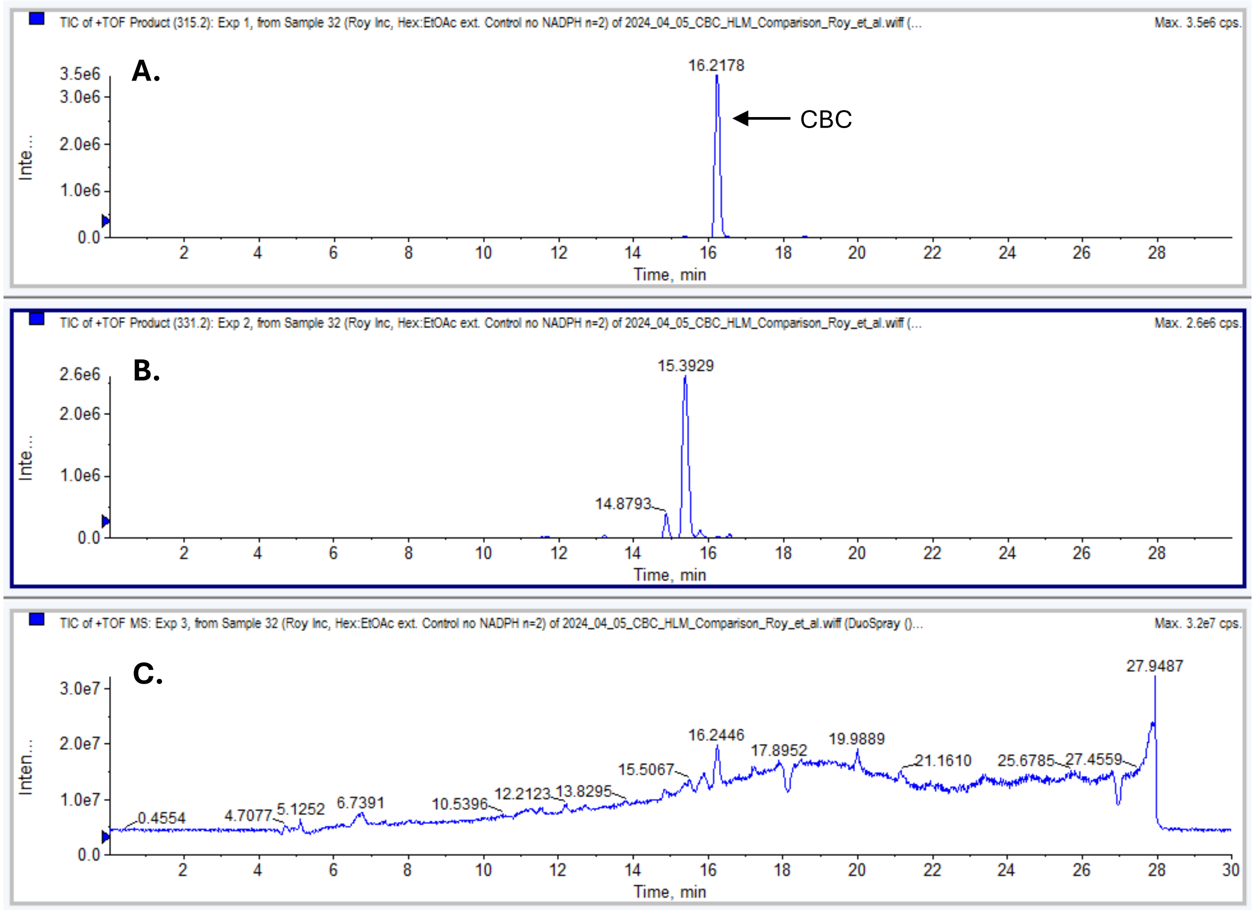

Figure S2.1.6. Representative chromatograms of a control sample (without NADPH solution) after incubation of CBC with HLM and extraction following the protocol described by Roy et al. (additional details on page 4). (A) Extracted ion chromatogram of *m/z* = 315.2, (B) extracted ion chromatogram of *m/z* = 331.2, and (C) total ion chromatogram from scan mode (*m/z* = 50-600).

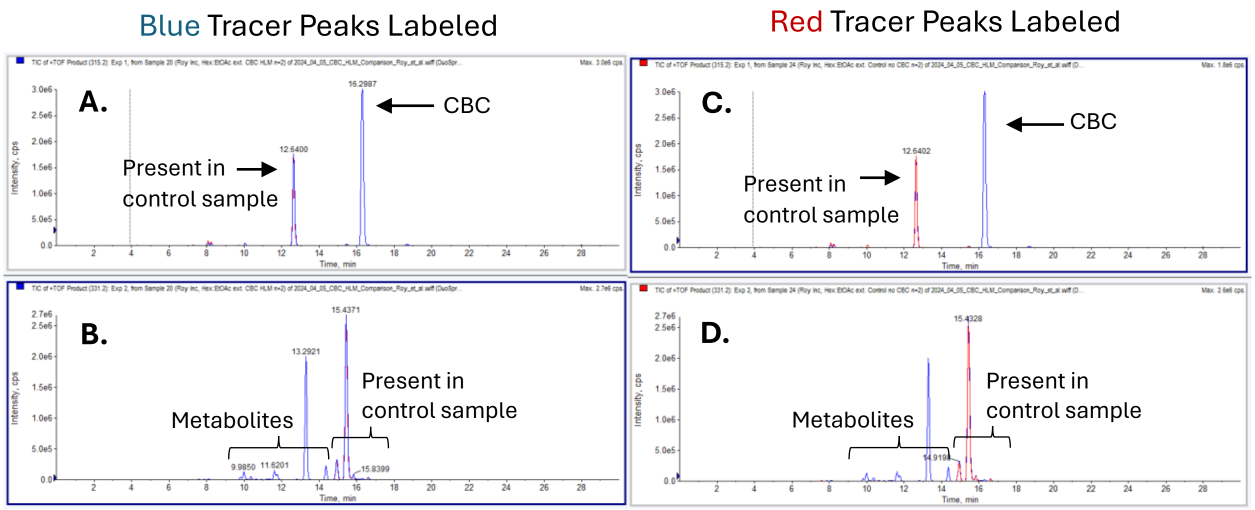

Figure S2.1.7. Overlay of representative extracted ion chromatograms after incubation of CBC with HLM (+ NADPH solution) and extraction following the protocol described by Roy et al. (blue tracer) and control sample without CBC (+ HLM, + NADPH solution) (red tracer); additional details on page 4. (A) Extracted ion chromatogram of *m/z* = 315.2 with blue tracer peaks labeled, (B) extracted ion chromatogram of *m/z* = 331.2 with blue peaks labeled, (C) extracted ion chromatogram of *m/z* = 315.2 with red tracer peaks labeled, and (D) extracted ion chromatogram of *m/z* = 331.2 with red peaks labeled.

With this overlay, peaks resulting from metabolites and HLM background can be identified. The resulting metabolite profiles from incubation/extraction parameters described by Roy et al. can be compared to our study described herein.

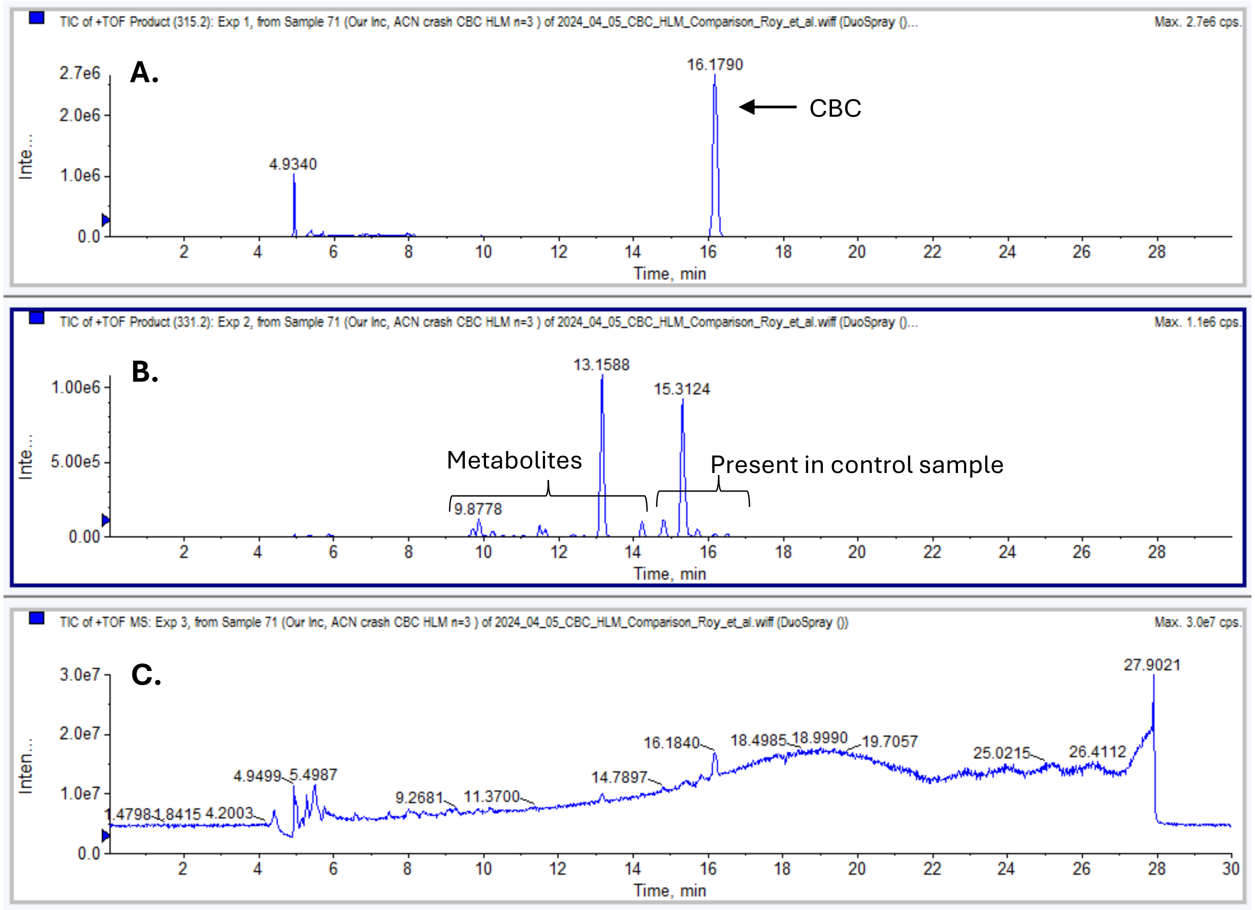

Figure S2.1.8. Representative chromatograms after incubation of CBC with HLM (+ NADPH generating system) and extraction following the present protocol described herein (details on page 4). (A) Extracted ion chromatogram of *m/z* = 315.2, (B) extracted ion chromatogram of *m/z* = 331.2, and (C) total ion chromatogram from scan mode (*m/z* = 50-600).

Representative control samples are shown in Figures S2.1.9-S2.1.11.

These chromatograms display the chromatographic pattern of metabolites generated with the incubation and extraction parameters of the present protocol described herein. With comparison to the control samples, we can assign extraneous peaks resulting from the matrix.

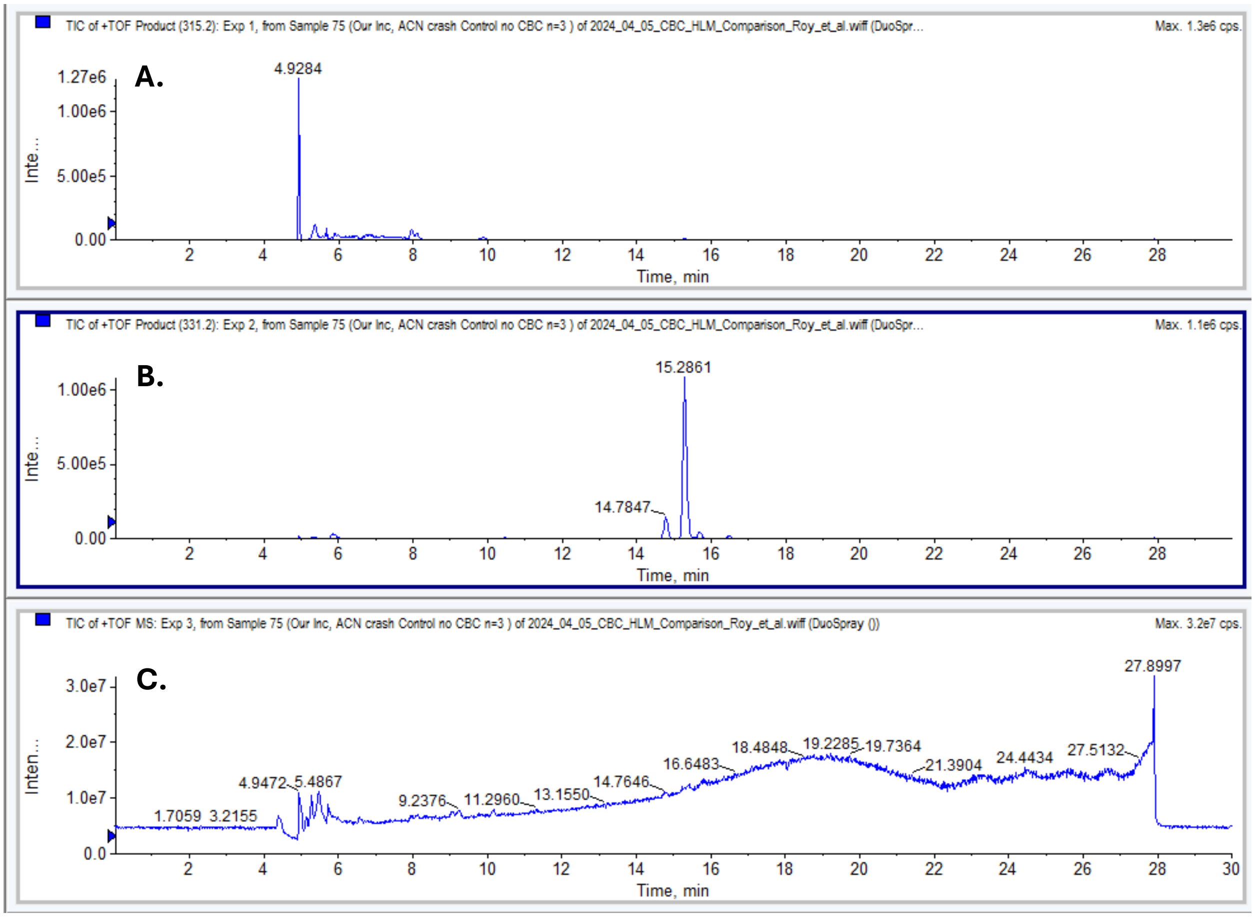

Figure S2.1.9. Representative chromatograms of a control sample (without CBC) after incubation of HLM + NADPH generating system and extraction following the present protocol described herein (details on page 4). (A) Extracted ion chromatogram of *m/z* = 315.2, (B) extracted ion chromatogram of *m/z* = 331.2, and (C) total ion chromatogram from scan mode (*m/z* = 50-600).

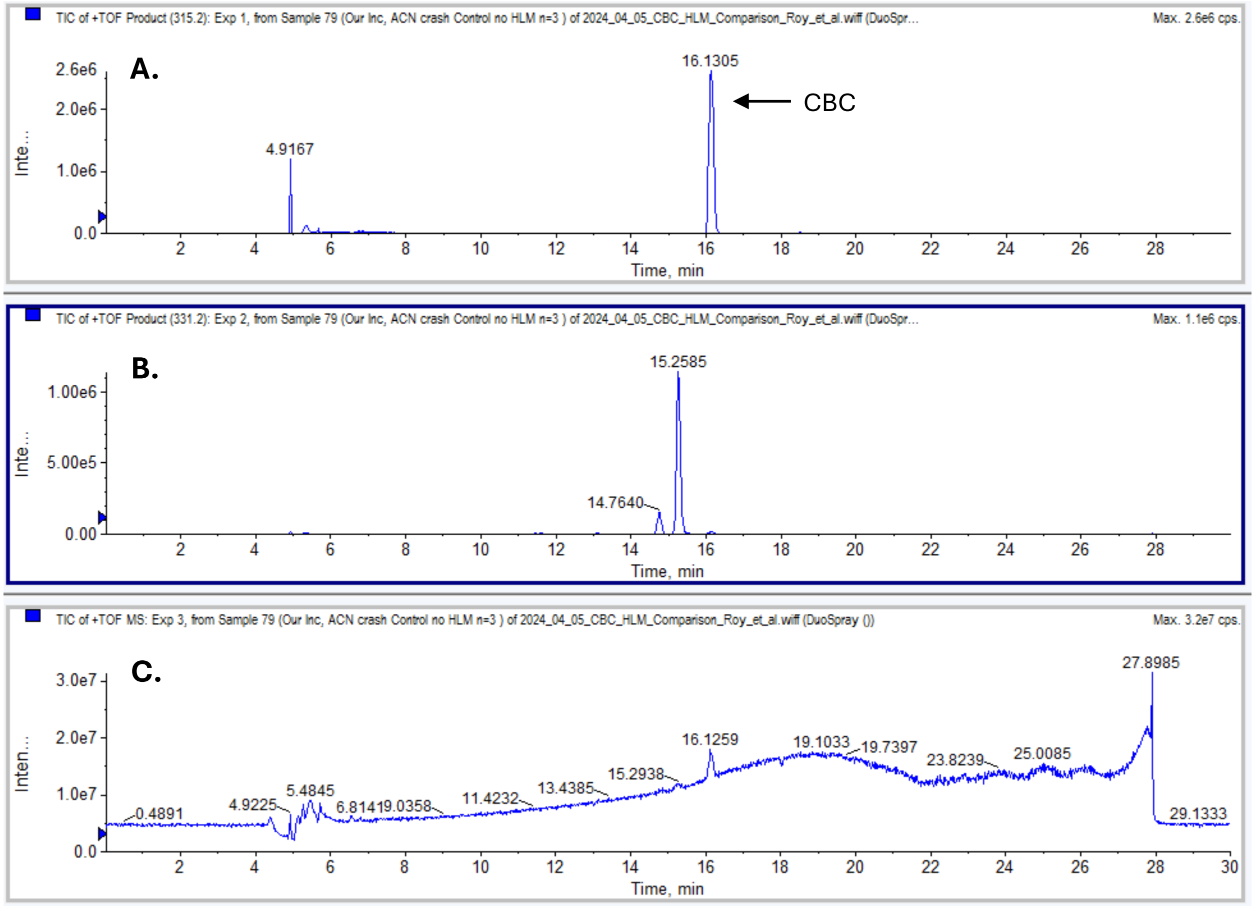

Figure S2.1.10. Representative chromatograms of a control sample (without HLM) after incubation of CBC + NADPH generating system and extraction following the present protocol described herein (details on page 4). (A) Extracted ion chromatogram of *m/z* = 315.2, (B) extracted ion chromatogram of *m/z* = 331.2, and (C) total ion chromatogram from scan mode (*m/z* = 50-600).

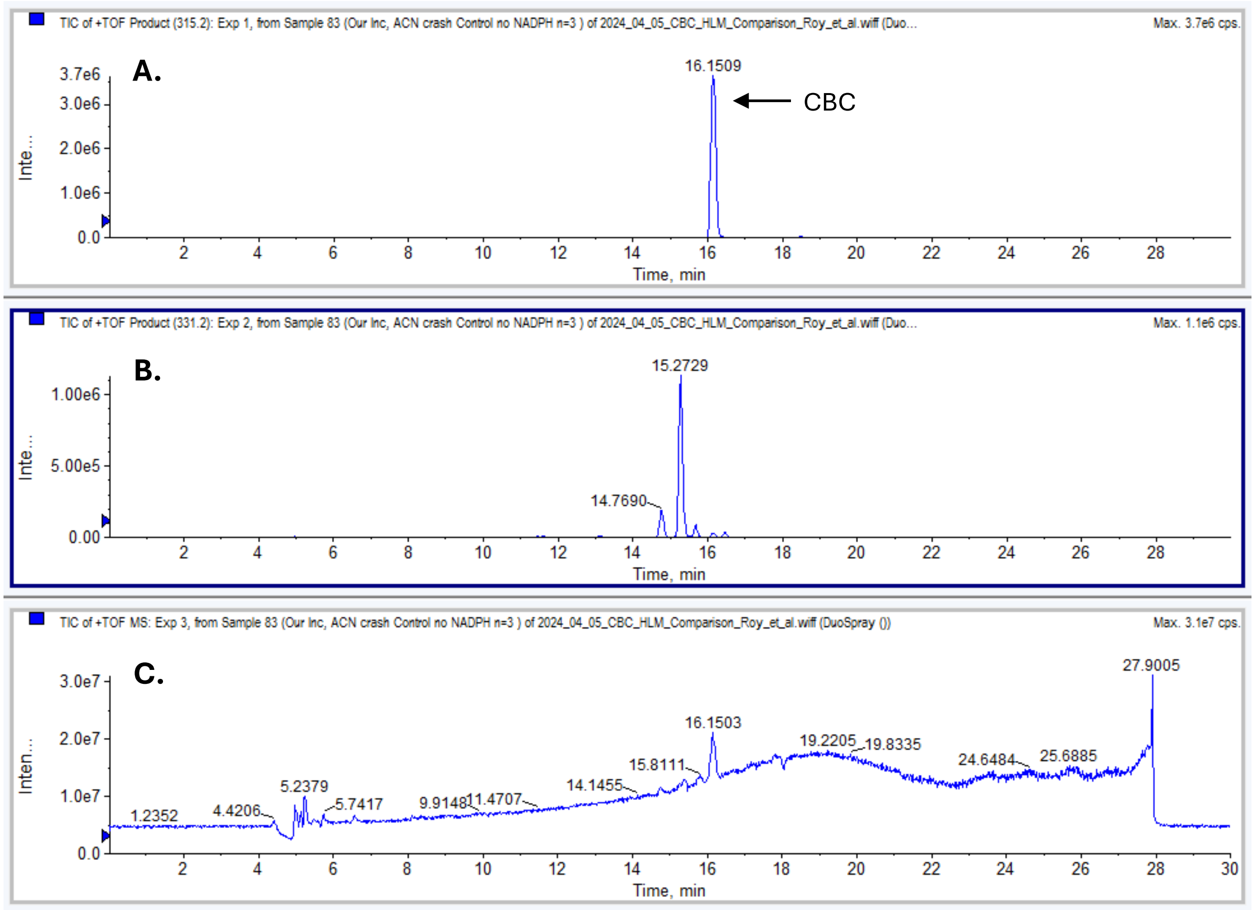

Figure S2.1.11. Representative chromatograms of a control sample (without NADPH generating system) after incubation of CBC with HLM and extraction following the present protocol described herein (details on page 4). (A) Extracted ion chromatogram of *m/z* = 315.2, (B) extracted ion chromatogram of *m/z* = 331.2, and (C) total ion chromatogram from scan mode (*m/z* = 50-600).

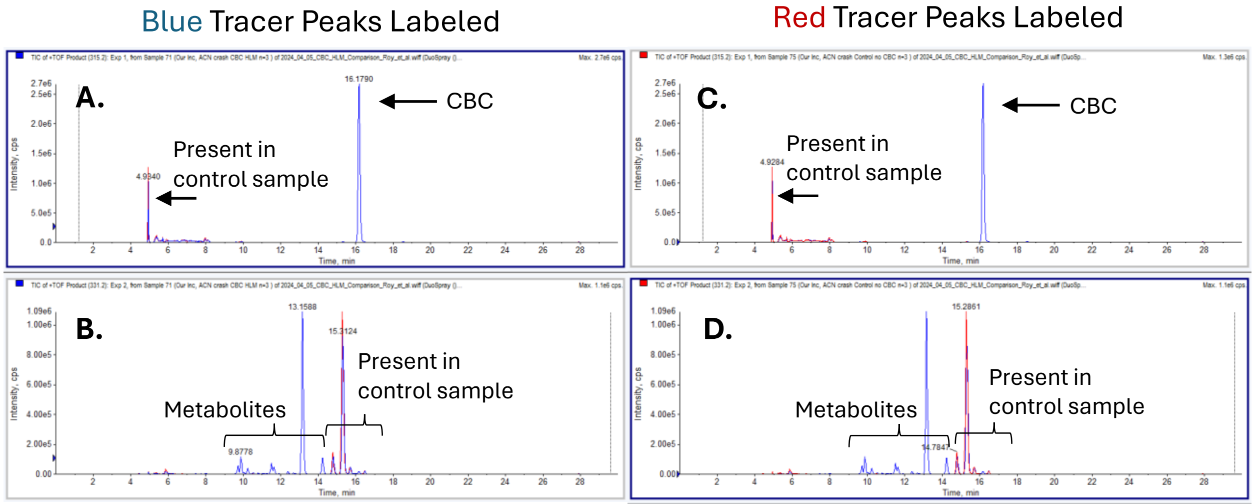

Figure S2.1.12. Overlay of representative extracted ion chromatograms after incubation of CBC with HLM (+ NADPH generating system) and extraction following the present protocol described herein (blue tracer) and control sample without CBC (+ HLM, + NADPH generating system) (red tracer); additional details on page 4. (A) Extracted ion chromatogram of *m/z* = 315.2 with blue tracer peaks labeled, (B) extracted ion chromatogram of *m/z* = 331.2 with blue peaks labeled, (C) extracted ion chromatogram of *m/z* = 315.2 with red tracer peaks labeled, and (D) extracted ion chromatogram of *m/z* = 331.2 with red peaks labeled.

With this overlay, peaks resulting from metabolites and HLM background can be identified. The resulting metabolite profiles from incubation/extraction parameters described by the study herein can be compared to Roy et al. (Figures S2.1.13-S2.1.18).

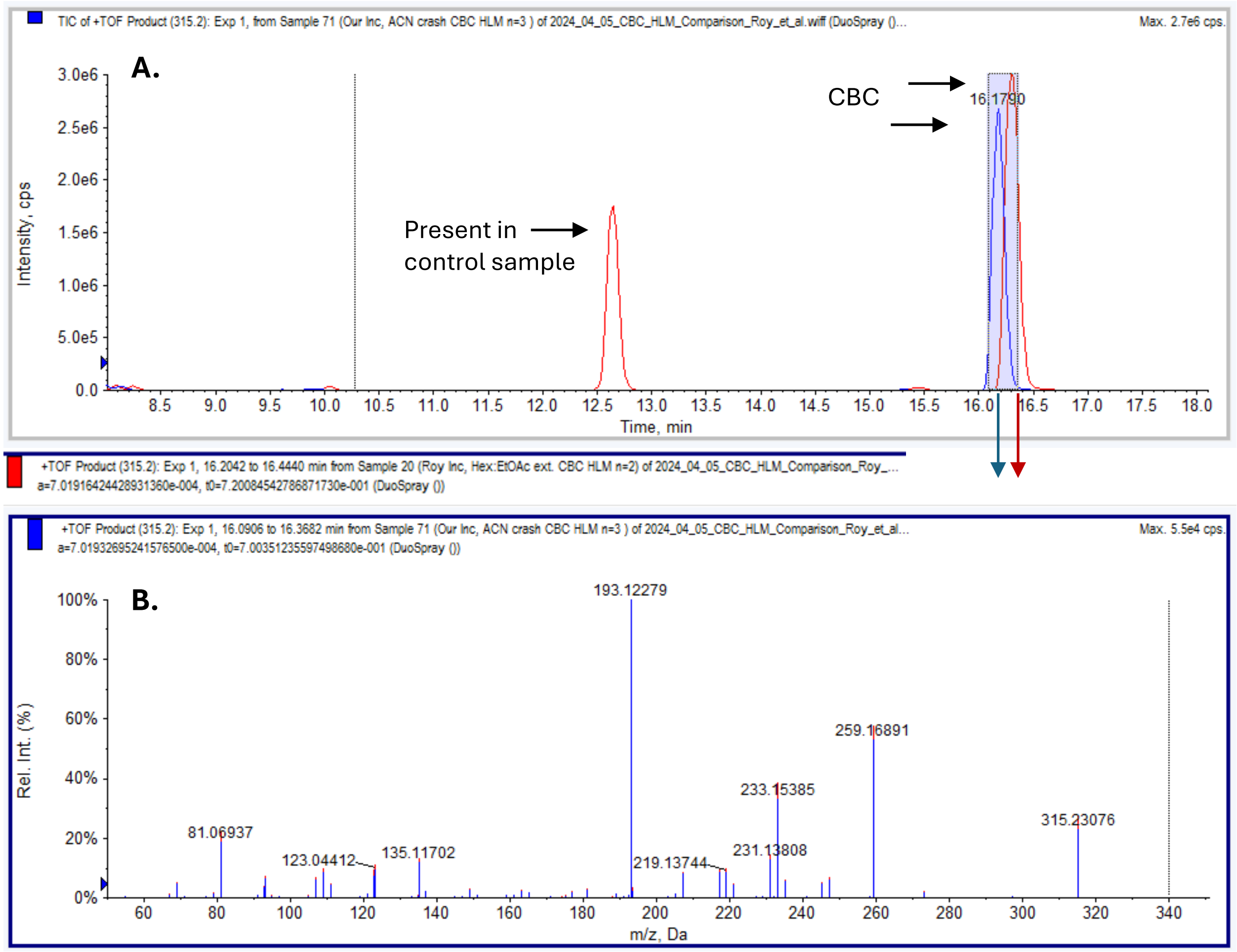

Figure S2.1.13. Overlay of extracted ion chromatogram and mass spectra of representative samples of the study herein incubation and extraction parameters of CBC incubated with HLM (blue tracer) and Roy et al. incubation and extraction parameters of CBC incubated with HLM (red tracer). (A) Extracted ion chromatogram of m/z = 315.2 and (B) overlay of mass spectra of CBC stemming from the primary peaks in (A). Please note that the two spectra are identical so that the red signals are mostly covered by the blue signals. Blue and red arrows emphasize where the mass spectrum originates from for each respective peak.

Comparing the CBC signal from the study herein and Roy et al., there is a slight shift in retention time which may be due to the solvent. Roy et al. reconstituted in 95% ethanol while the study herein is approximately a 50:50 mixture of acetonitrile and PBS/NADPH generating system. However, despite this retention time shift, the fragmentation pattern of CBC is identical.

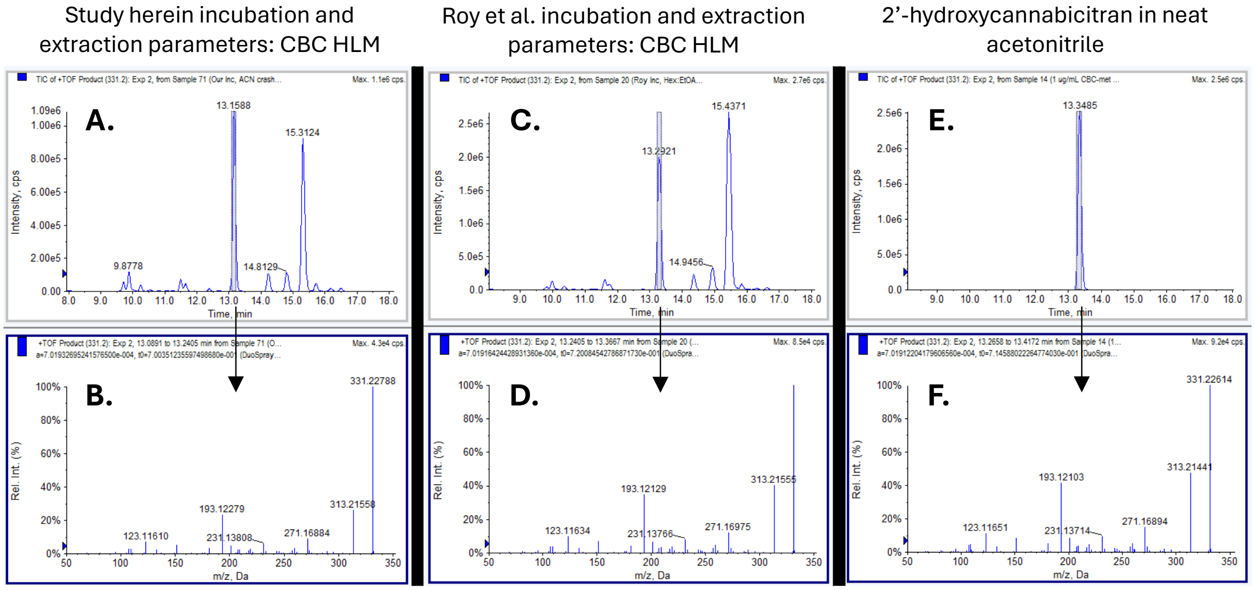

Figure S2.1.14. Side-by-side comparison of chromatography and mass spectra of 2'-hydroxycannabicitran at *m/z* = 331.2 of various test samples. (A) Extracted ion chromatogram of *m/z* = 331.2 and (B) mass spectrum of 2'-hydroxycannabicitran resulting from the incubation and extraction parameters of the study herein. (C) Extracted ion chromatogram of *m/z* = 331.2 and (D) mass spectrum of 2'-hydroxycannabicitran resulting after incubation of CBC with HLM + NADPH solution and extraction as described by Roy et al. (E) Extracted ion chromatogram of *m/z* = 331.2 and (F) mass spectrum of 2'-hydroxycannabicitran resulting from the synthetic generation, verified via NMR spectroscopy. Arrows emphasize where the mass spectrum originates from for each respective peak.

Overlay of these samples is shown in Figure S2.1.15.

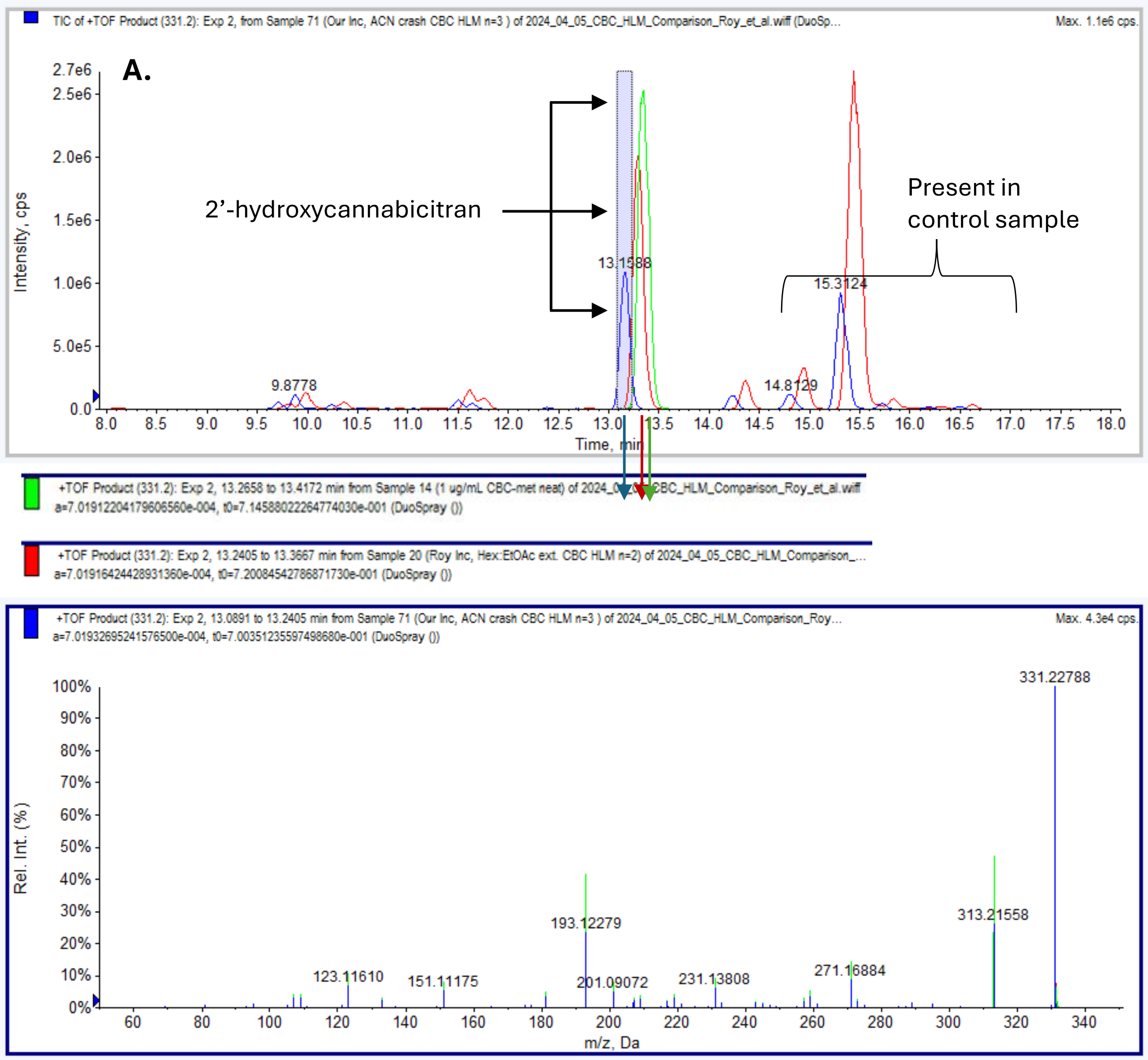

Figure S2.1.15. Overlay of various samples highlighting the major metabolite of CBC (2’-hydroxycannabicitran). (A) Extracted ion chromatogram of *m/z* = 331.2 (blue tracer) is from the incubation of CBC with HLM + NADPH generating system and extraction parameters of the study herein. The red tracer shows the extracted *m/z* = 331.2 resulting after incubation of CBC with HLM + NADPH solution and extraction as described by Roy et al., and green tracer is the extracted *m/z* = 331.2 from the synthesized metabolite as verified by NMR spectroscopy. (B) overlay of mass spectra of 2’-hydroxycannabicitran stemming from the primary metabolite peaks in (A). Blue, red, and green arrows emphasize where the mass spectrum originates from for each respective peak.

Again, there is a slight retention time shift in chromatography with sample number (blue sample 71, red sample 20, and green sample 14) in the batch, or the retention time shift may be a result of differing sample solvents (blue: 50:50 PBS buffer:acetonitrile, red: 95% ethanol, and green: 100% acetonitrile). However, in (B) all three samples are overlaid, and they show an identical fragmentation pattern of 2’-hydroxycannabicitran generated synthetically and with HLMs. Please note that the spectra are almost identical, and the red and green signals are mostly covered by the blue signals. Closer to consecutive reinjection of representative samples are shown in Figure S.2.1.18. Zoomed in overlaid mass spectra is shown in Figure S2.1.16 and S2.1.17.

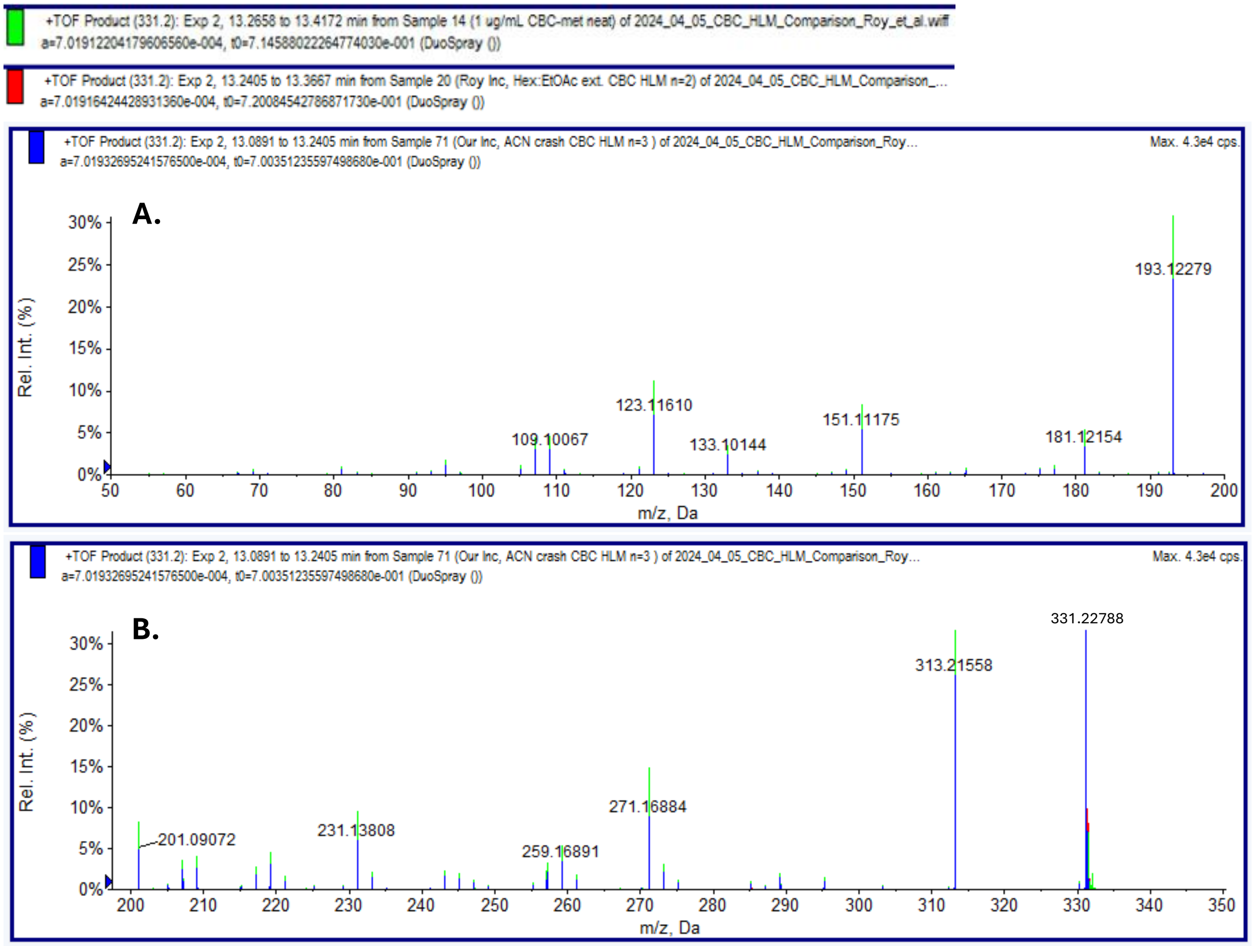

Figure S2.1.16. Zoomed-in overlay of various samples highlighting the major metabolite of CBC (2'-hydroxycannabicitran) from Figure S2.1.15. Mass spectra are stemming from the major metabolite peak observed at *m/z* = 331.2. (A) Zoomed-in to *m/z* = 50-200 and (B) Zoomed-in to *m/z* = 200-350. The blue tracer is after incubation of CBC, HLM + NADPH generating system and extraction as described herein, red tracer is after incubation of CBC, HLM + NADPH solution and extraction as described by Roy et al., and the green tracer is from the synthetic metabolite as verified via NMR spectroscopy.

All three samples are overlaid, and they show identical fragmentation patterns of 2'-hydroxycannabicitran generated synthetically and with HLMs using the two different incubation and extraction methods as described herein and by Roy et al. Specific fragments are shown in Figure S2.1.17.

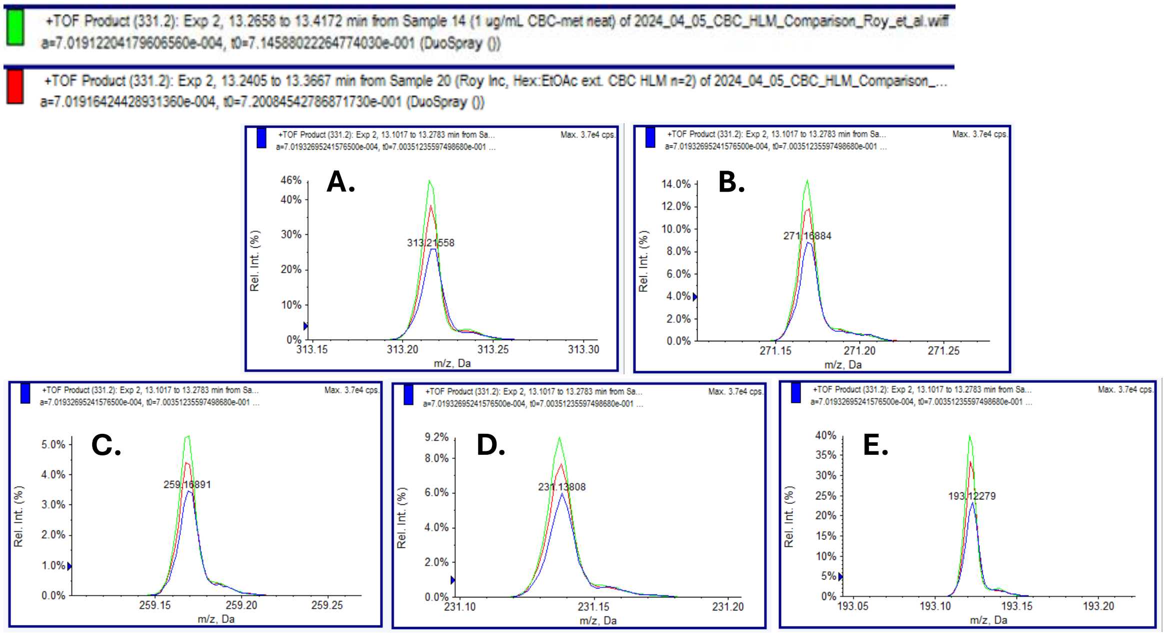

Figure S2.1.17. Zoomed-in overlay of various samples highlighting specific fragments of the major metabolite of CBC (2'-hydroxycannabicitran) from Figure S2.1.15. MS/MS are stemming from the major metabolite peak $[M+H]^+$ *m/z* = 331.2. The blue tracer is after incubation of CBC, HLM + NADPH generating system and extraction as described herein, red tracer is after incubation of CBC, HLM + NADPH solution and extraction as described by Roy et al., and the green tracer is from the synthetic metabolite as verified via NMR spectroscopy. (A) highlights fragment *m/z* = 313.2, (B) *m/z* = 271.2, (C) *m/z* = 259.2, (D) *m/z* = 231.1, and (E) *m/z* = 193.1.

In conclusion, the major metabolite of CBC generated using the incubation/extraction parameters described by Roy et al. and used in the study herein is identical. Not only are the MS/MS fragments identical but also, as shown in this figure, their intensities and ratios are as well. Synthetically generated and NMR verified 2'-hydroxycannabicitran matched the metabolites generated by incubation with HLM chromatographically and based on the overlaid mass spectra.

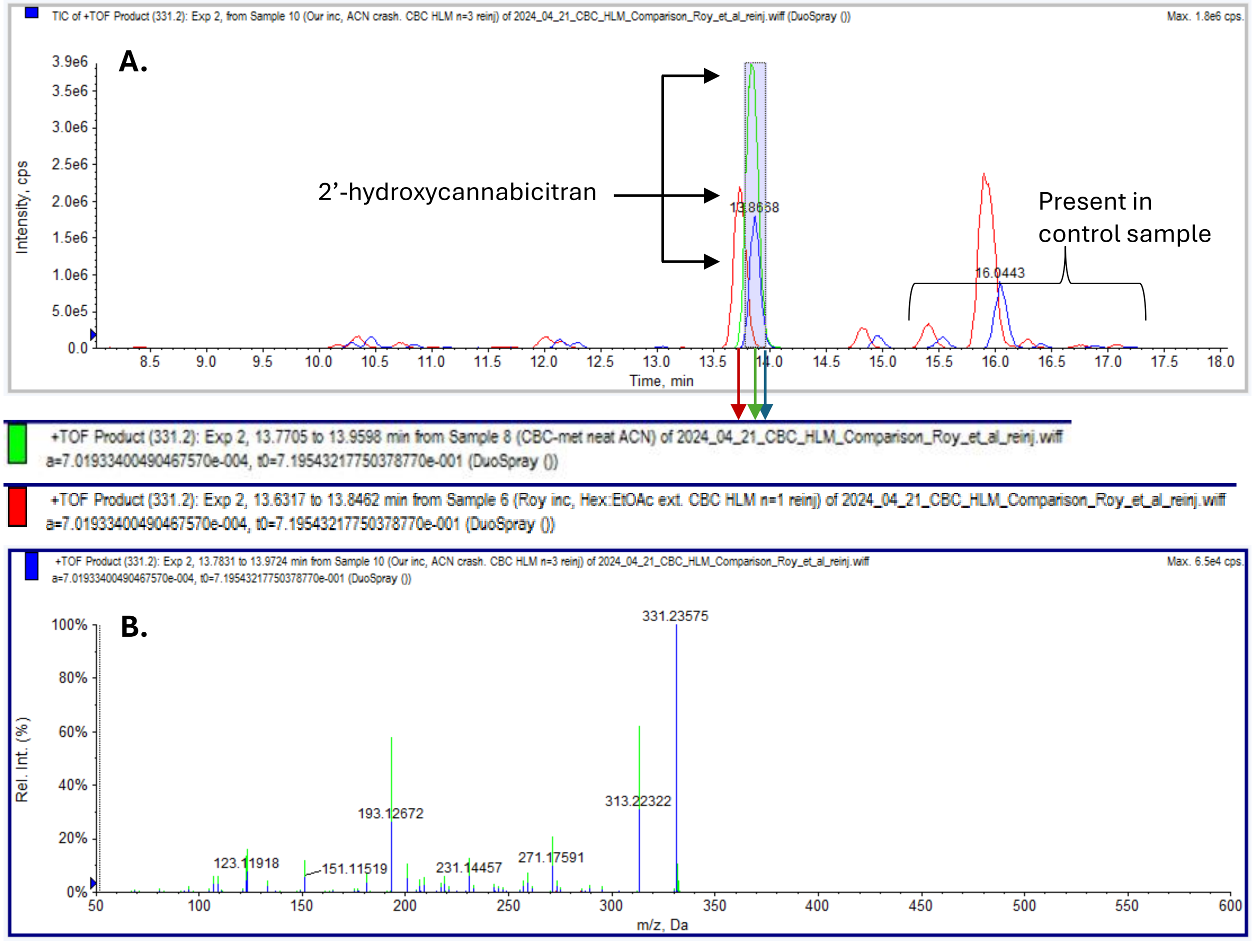

Figure S2.1.18. Overlay of near consecutive injections of various samples highlighting the major metabolite of CBC (2’-hydroxycannabicitran). (A) Extracted ion chromatograms of *m/z* = 331.2. The blue tracer is after incubation of CBC, HLM + NADPH generating system and extraction as described herein, the red tracer is after incubation of CBC, HLM + NADPH solution and extraction as described by Roy et al., and the green tracer is from the synthetic metabolite as verified via NMR spectroscopy. (B) Overlay of mass spectra of 2’-hydroxycannabicitran stemming from the primary metabolite peaks in (A). Blue, red, and green arrows emphasize where the mass spectrum originates from for each respective peak.

The batch queue was: acetonitrile (x5), Roy et al. incubation and extraction: CBC, HLM + NADPH solution, acetonitrile, 2’-hydroxycannabicitran in neat acetonitrile, acetonitrile, incubation and extraction following the protocol as described herein: CBC, HLM + NADPH generating system, acetonitrile.

The retention time shifts are likely a result of differing sample solvents (blue: 50:50 acetonitrile:PBS buffer/NADPH generating system, red: 95% ethanol, and green: 100% acetonitrile). Major and minor peaks show identical retention time shifts relative to one another in the samples extracted after incubation with CBC, HLM and NADPH solution or an NADPH generating system.

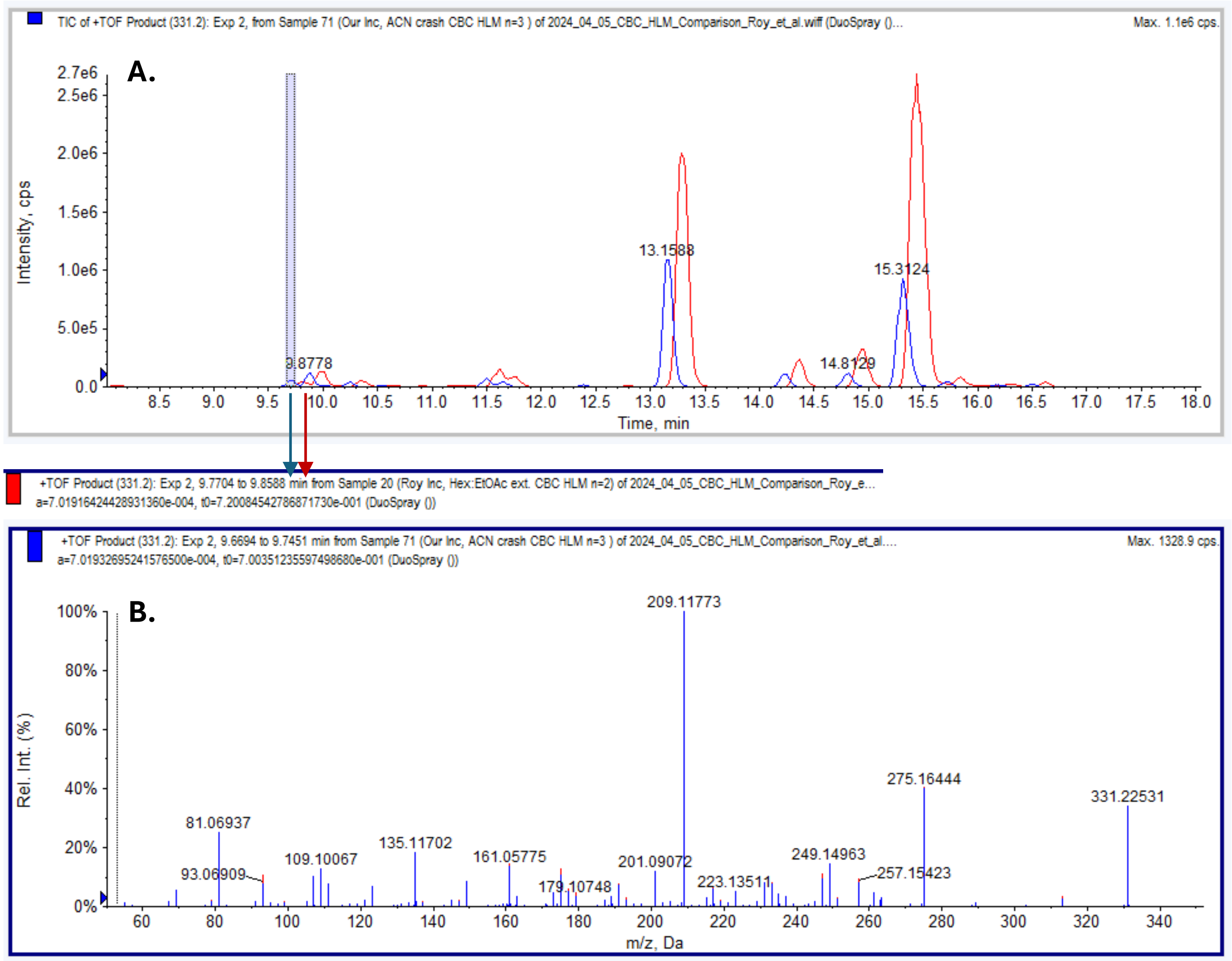

Figure S2.1.19. Overlay of representative extracted ion chromatograms and mass spectra of samples after incubation of CBC, HLM + NADPH generating system and extraction as described herein (blue tracer) and after incubation of CBC, HLM + NADPH solution and extraction as described by Roy et al. (red tracer). (A) Extracted ion chromatogram of *m/z* = 331.2 and (B) overlay of mass spectra of unknown minor metabolite 1 stemming from the indicated peaks in (A). Blue and red arrows emphasize where the mass spectrum originates from for each respective peak.

The overlay of the mass spectra shows an identical fragmentation pattern of the unknown minor metabolite 1 generated by both incubation and extraction methods. Since both mass spectra are almost identical, the red signals are mostly covered by the blue signals.

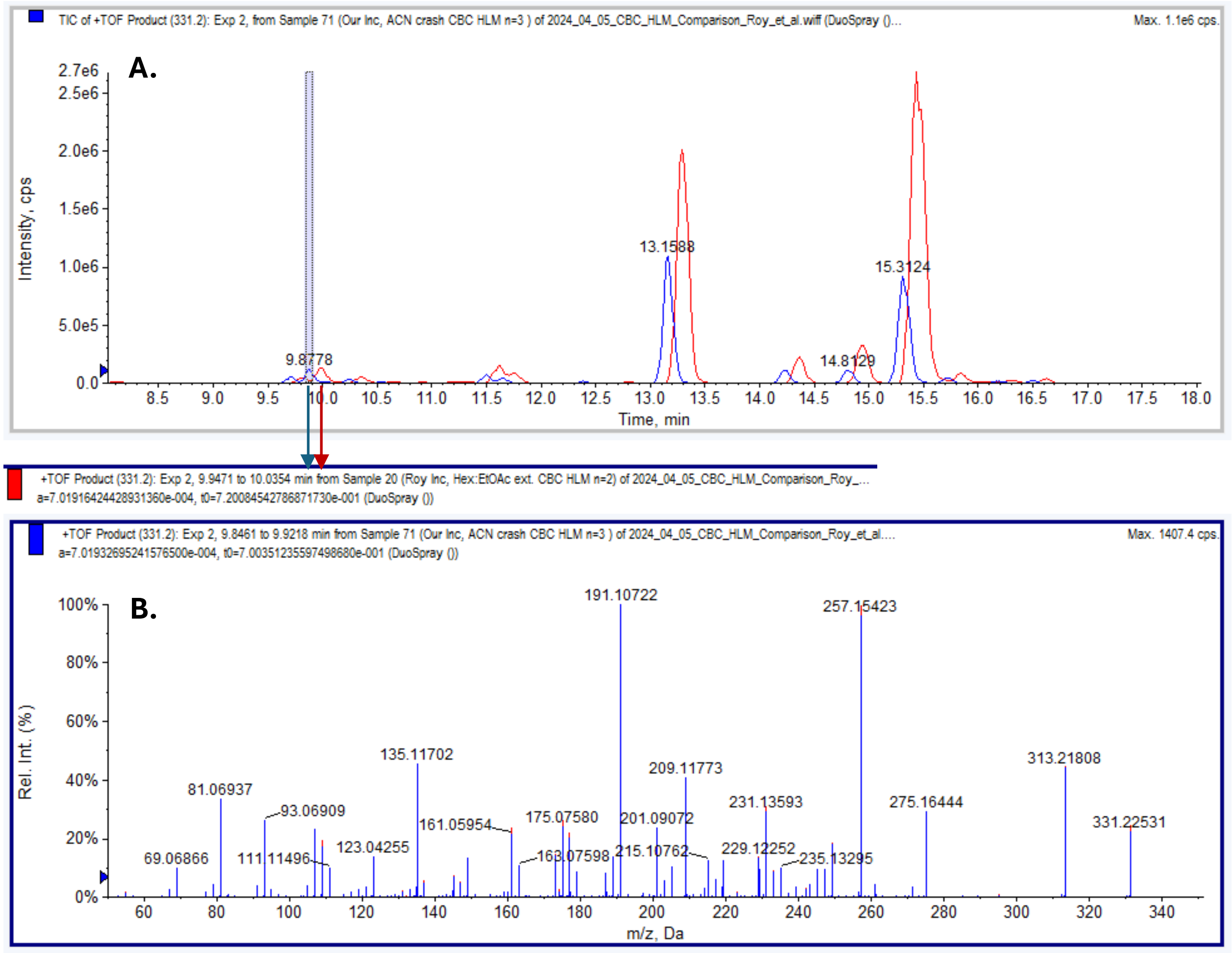

Figure S2.1.20. Overlay of representative extracted ion chromatograms and mass spectra of samples after incubation of CBC, HLM + NADPH generating system and extraction as described herein (blue tracer) and after incubation of CBC, HLM + NADPH solution and extraction as described by Roy et al. (red tracer). (A) Extracted ion chromatogram of *m/z* = 331.2 and (B) overlay of mass spectra of unknown minor metabolite 2 stemming from the indicated peaks in (A). Blue and red arrows emphasize where the mass spectrum originates from for each respective peak.

The overlay of the mass spectra shows an identical fragmentation pattern of the unknown minor metabolite 2 generated by both incubation and extraction methods. Since both mass spectra are almost identical, the red signals are mostly covered by the blue signals.

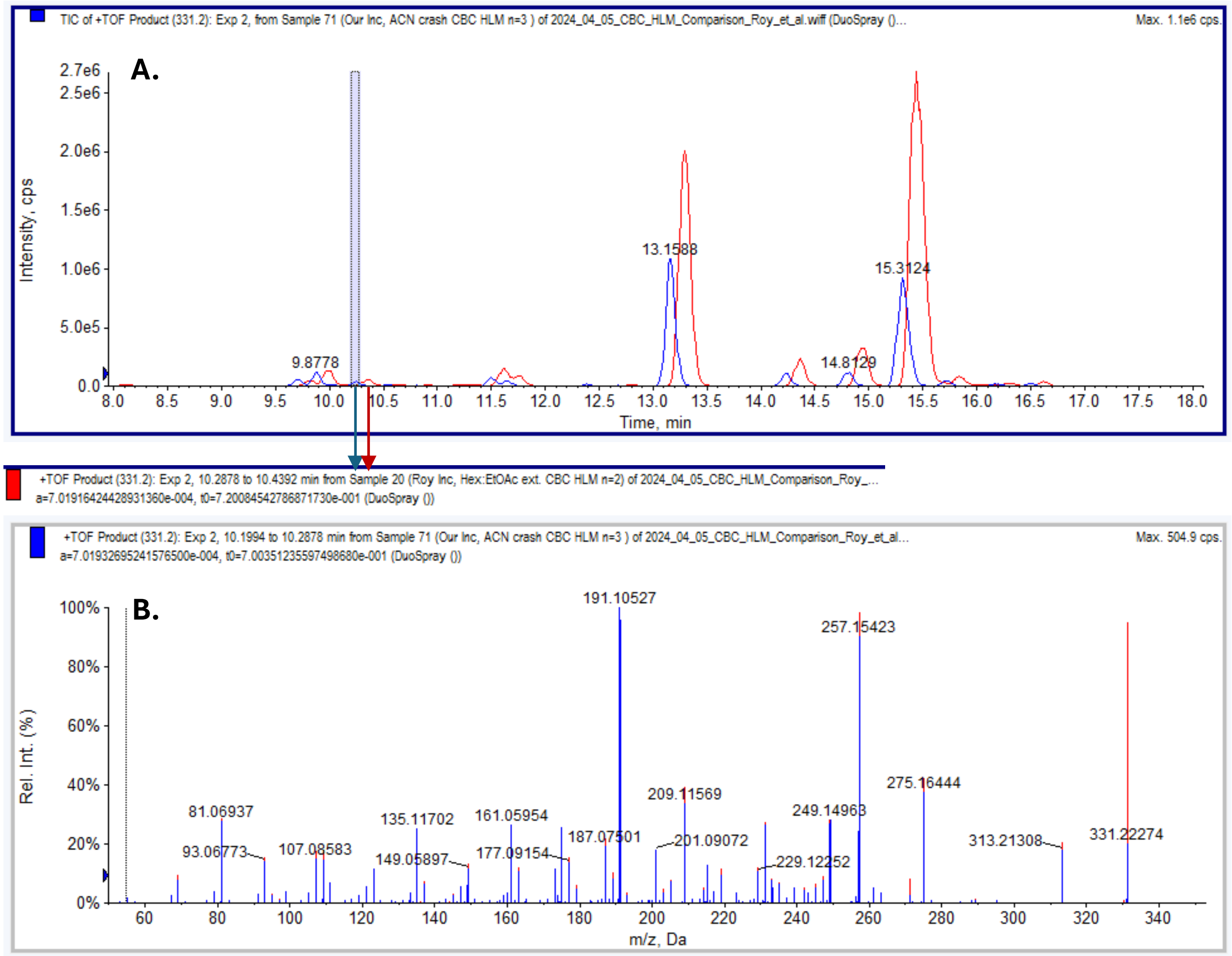

Figure S2.1.21. Overlay of representative extracted ion chromatograms and mass spectra of samples after incubation of CBC, HLM + NADPH generating system and extraction as described herein (blue tracer) and after incubation of CBC, HLM + NADPH solution and extraction as described by Roy et al. (red tracer). (A) Extracted ion chromatogram of *m/z* = 331.2 and (B) overlay of mass spectra of unknown minor metabolite 3 stemming from the indicated peaks in (A). Blue and red arrows emphasize where the mass spectrum originates from for each respective peak.

The overlay of the mass spectra shows an identical fragmentation pattern of the unknown minor metabolite 3 generated by both incubation and extraction methods. Since both mass spectra are almost identical, the red signals are mostly covered by the blue signals.

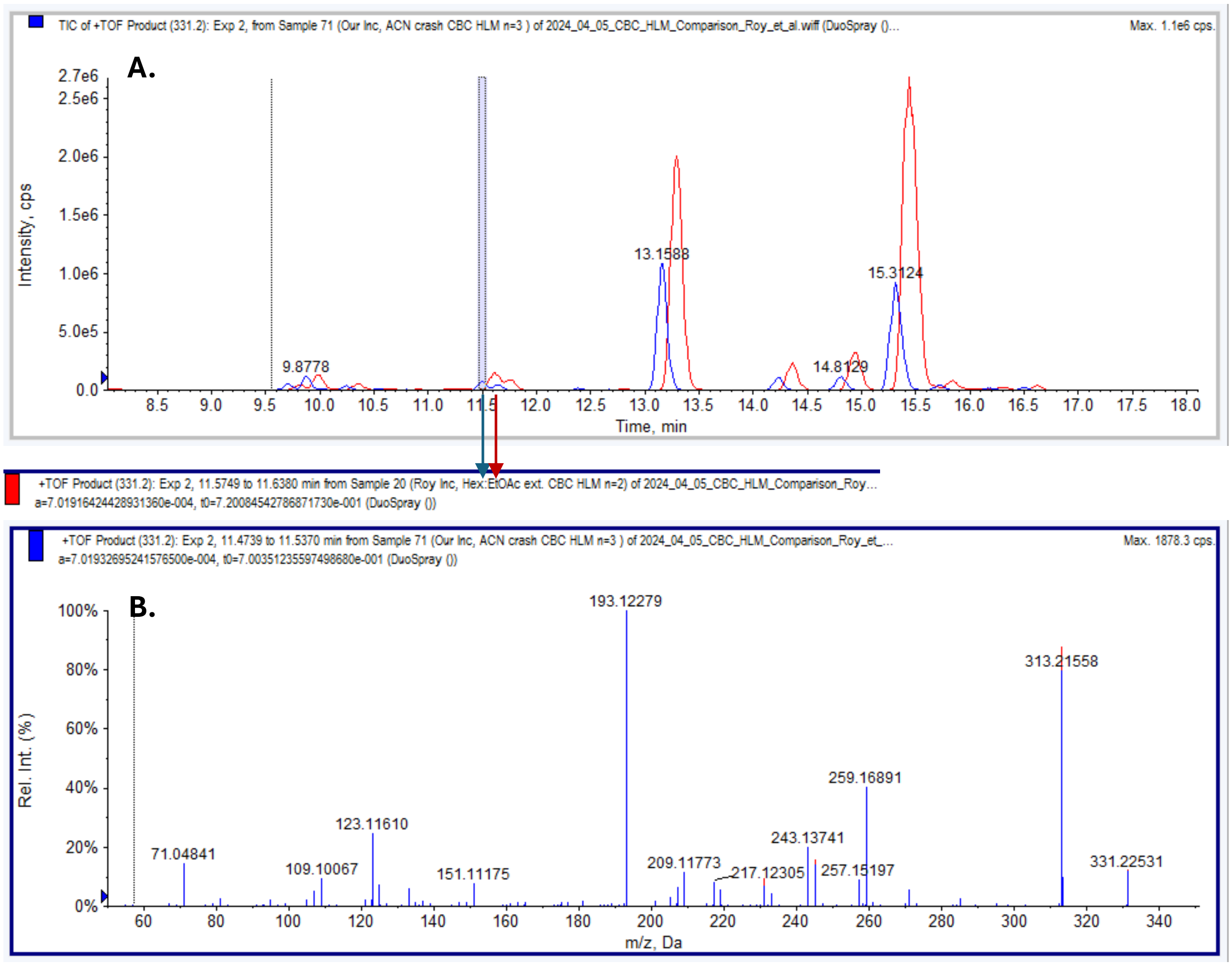

Figure S2.1.22. Overlay of representative extracted ion chromatograms and mass spectra of samples after incubation of CBC, HLM + NADPH generating system and extraction as described herein (blue tracer) and after incubation of CBC, HLM + NADPH solution and extraction as described by Roy et al. (red tracer). (A) Extracted ion chromatogram of *m/z* = 331.2 and (B) overlay of mass spectra of unknown minor metabolite 4 and 6',7'-epoxy-CBC stemming from the indicated peaks in (A). Blue and red arrows emphasize where the mass spectrum originates from for each respective peak.

With both mass spectra overlaid, there is an identical fragmentation pattern of unknown minor metabolite 4 and 6',7'-epoxy-CBC generated with both sample preparation methods.

Baseline chromatographic separation was not achieved with the described method; therefore, fragments cannot be definitively assigned as one species or another.

Fragmentation pattern for 6',7'-epoxy-CBC is shown in Figure 2.1.27, Figure 2.1.28, and Table S2.1.2.

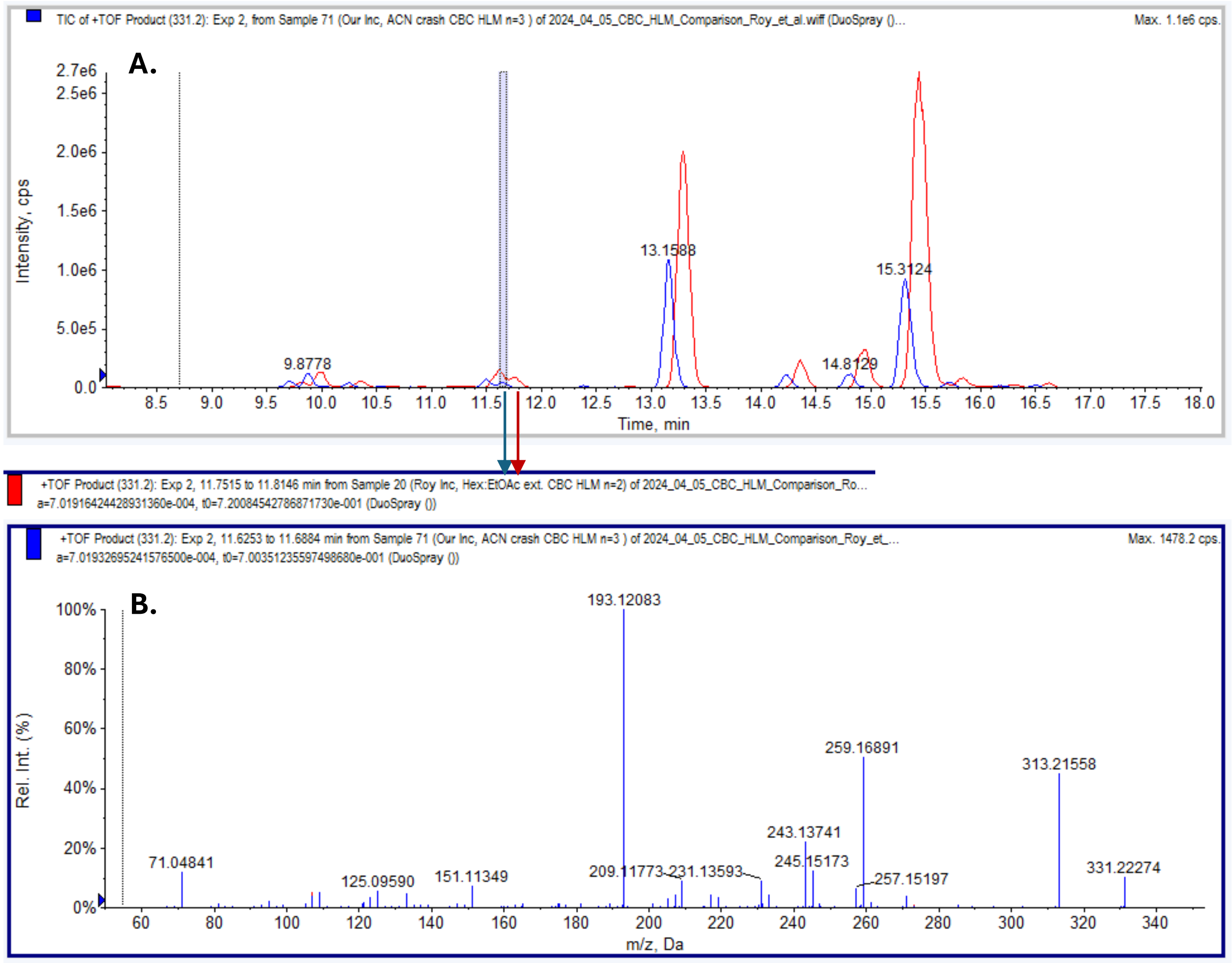

Figure S2.1.23. Overlay of representative extracted ion chromatograms and mass spectra of samples after incubation of CBC, HLM + NADPH generating system and extraction as described herein (blue tracer) and after incubation of CBC, HLM + NADPH solution and extraction as described by Roy et al. (red tracer). (A) Extracted ion chromatogram of *m/z* = 331.2 and (B) overlay of mass spectra of unknown minor metabolite 4 and 6',7'-epoxy-CBC stemming from the indicated peaks in (A). Blue and red arrows emphasize where the mass spectrum originates from for each respective peak.

With both mass spectra overlaid, there is an identical fragmentation pattern of unknown minor metabolite 4 and 6',7'-epoxy-CBC generated with both sample preparation methods.

Baseline chromatographic separation was not achieved with the described method; therefore, fragments cannot be definitively assigned as one species or another.

Fragmentation pattern for 6',7'-epoxy-CBC is shown in Figure 2.1.27, Figure 2.1.28, and Table S2.1.2.

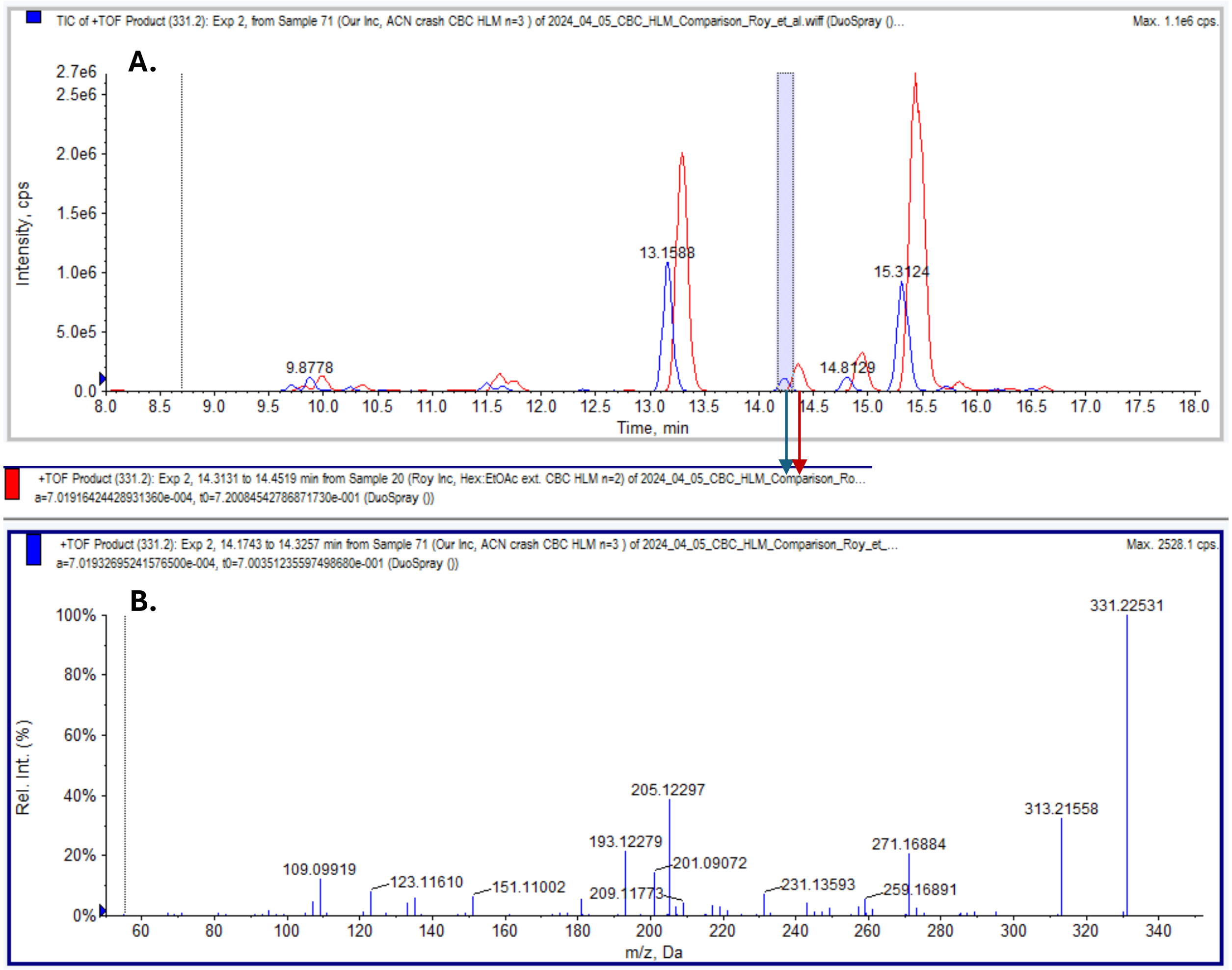

Figure S2.1.24. Overlay of representative extracted ion chromatograms and mass spectra of samples after incubation of CBC, HLM + NADPH generating system and extraction as described herein (blue tracer) and after incubation of CBC, HLM + NADPH solution and extraction as described by Roy et al. (red tracer). (A) Extracted ion chromatogram of *m/z* = 331.2 and (B) overlay of mass spectra of unknown minor metabolite 5 stemming from the indicated peaks in (A). Blue and red arrows emphasize where the mass spectrum originates from for each respective peak.

The overlay of the mass spectra shows an identical fragmentation pattern of the unknown minor metabolite 5 generated by both incubation and extraction methods. Since both mass spectra are almost identical, the red signals are mostly covered by the blue signals.

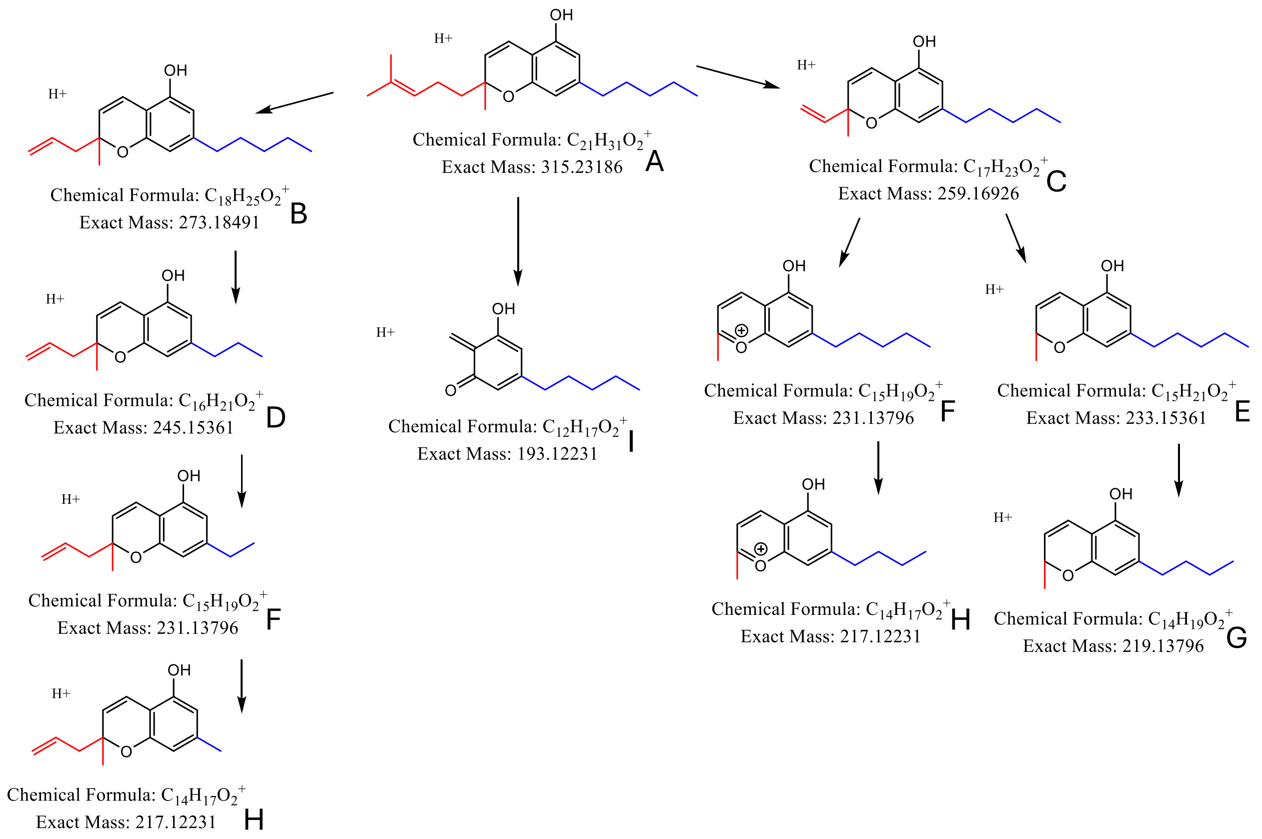

Figure S2.1.25. Fragmentation pattern of CBC. Fragment pathway determined from integrated direct infusion of a 5 µg/mL solution of CBC in pure acetonitrile into the electrospray source 6500 QTRAP mass spectrometer (AB Sciex, Concord, ON, Canada). The flow rate of the direct infusion syringe pump was 10 µL/min. The mass spectrometer parameters were as follows: ion source gas 1 15 psi, curtain gas 12 psi, source temperature 600 °C, ion spray voltage 5500 V, declustering potential 70 V, collision energy 25 eV, scan rate of 1000 Da/sec from 50 to 350 Da, and a product of 315.2 Da.

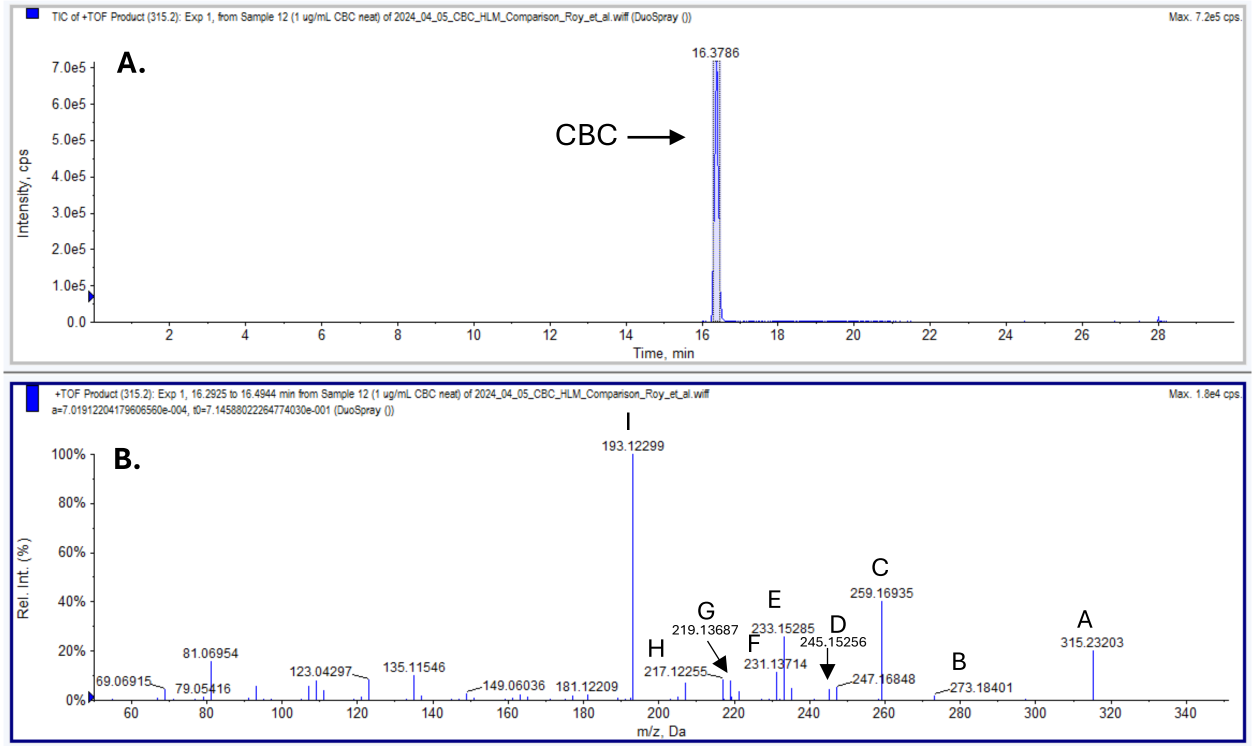

Figure S2.1.26. Extracted ion chromatogram and mass spectrum of CBC (1 µg/mL) in neat acetonitrile. (A) extracted ion chromatogram of *m/z* = 315.2 and (B) mass spectrum of CBC stemming from the primary peak in (A).

Table S2.1.1. Δppm of the exact mass of the proposed CBC fragment structures as shown in Figure S2.1.26 and their measured high-resolution mass.

	Theoretical exact mass (Da)	Measured high resolution mass (Da)	Δppm
A	315.23186	315.23203	0.539
B	273.18491	273.18401	3.294
C	259.16926	259.16935	0.347
D	245.15361	245.15256	4.283
E	233.15361	233.15285	3.26
F	231.13796	231.13714	3.548
G	219.13796	219.13687	4.974
H	217.12231	217.12255	1.105
I	193.12231	193.12299	3.521

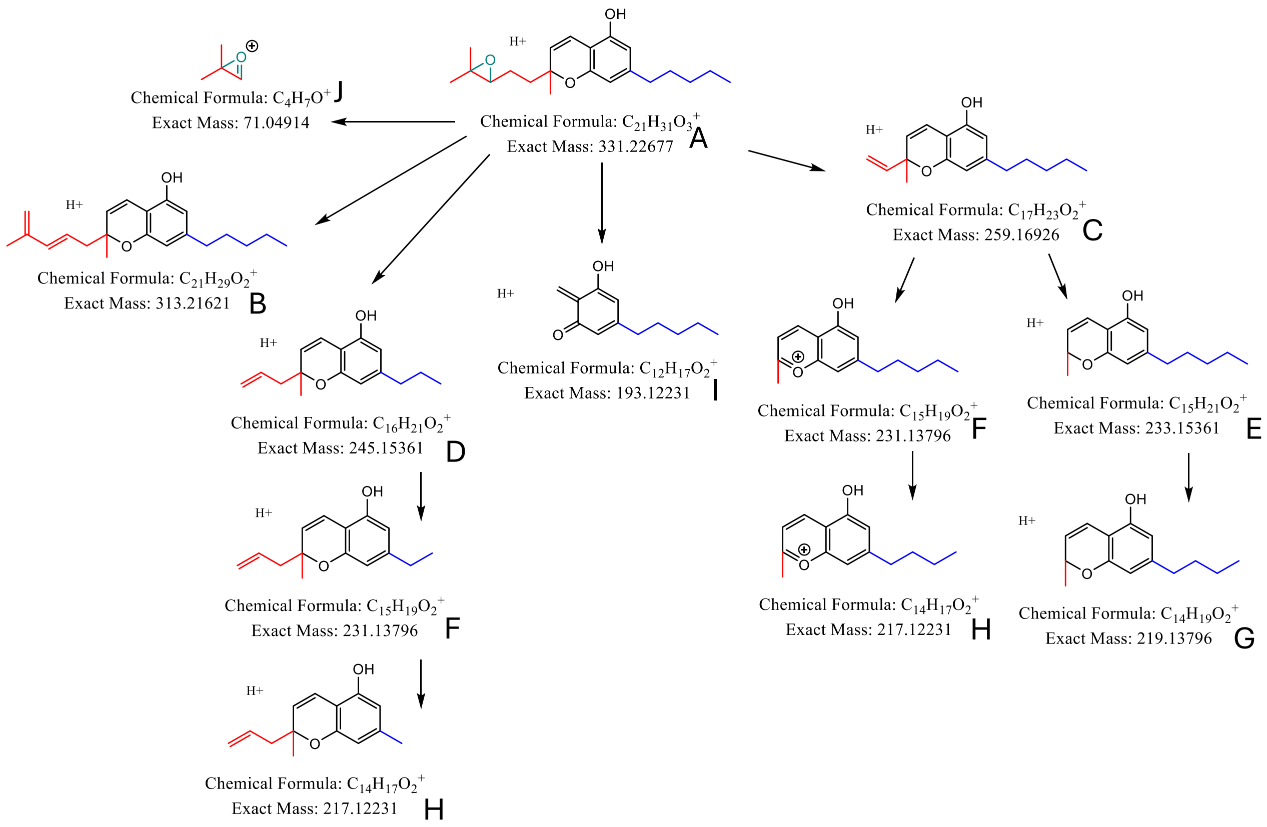

Figure S2.1.27. Fragmentation pattern of 6',7'-epoxy-CBC generated by reference to the fragmentation pattern of CBC (Figure S2.1.25).

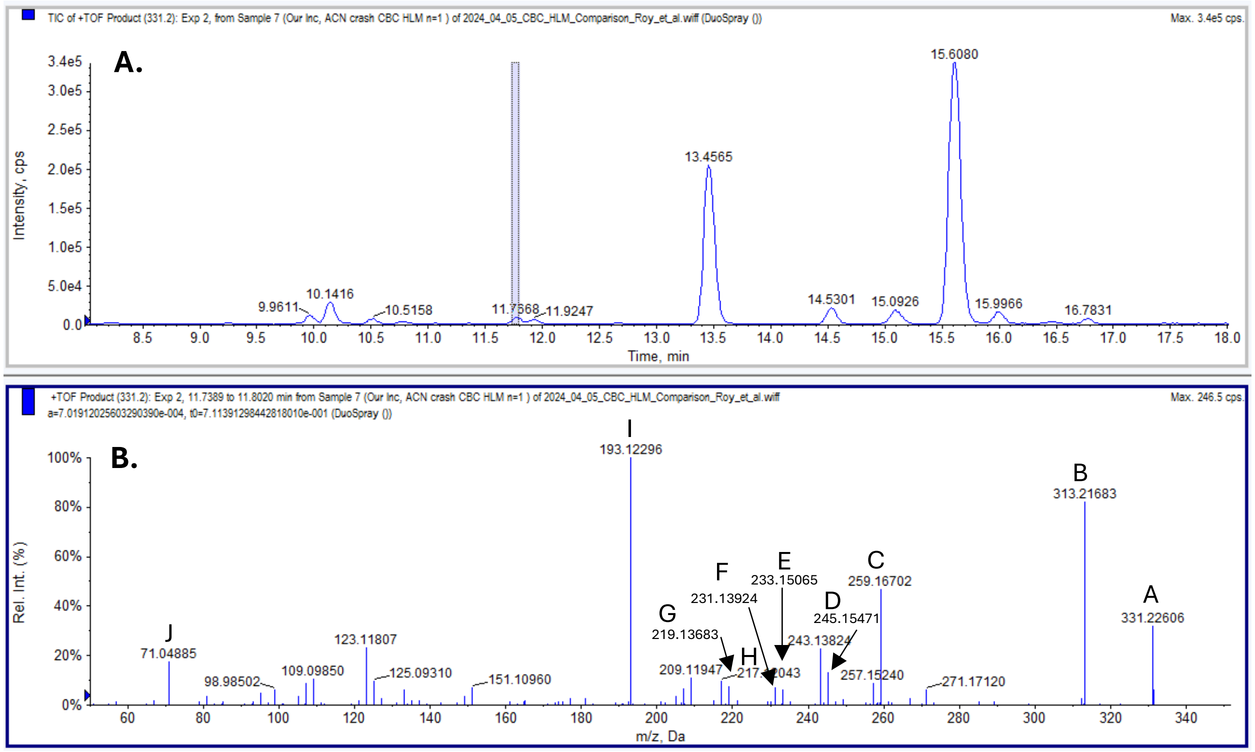

Figure S2.1.28. Representative extracted ion chromatogram of *m/z* = 331.2 and mass spectrum after incubation of CBC, HLM + NADPH generating system and extraction as described herein. (A) Extracted ion chromatogram of *m/z* = 331.2 and (B) mass spectrum of the highlighted peak in (A).

The fragment (71.04914 Da, J) is unique to this metabolite and due to α-cleavage adjacent to the oxidation position and, thus, is a key fragment for the identification of the metabolite. Δppm values of proposed fragments are displayed in Table S2.1.2. Generally, a Δppm less than 5 is thought to be acceptable. Notably, several Δppm values in Table S2.1.2 are greater than 5, this is likely due to the low signal intensity from the sample.

Table S2.1.2. Δppm of the exact mass of the proposed 6',7'-epoxy-CBC fragment structures as shown in Figure S2.1.28 and their measured high-resolution mass.

	Theoretical exact mass (Da)	Measured high-resolution mass (Da)	Δppm
A	331.22677	331.22606	2.144
B	313.21621	313.21683	1.979
C	259.16926	259.16702	8.643
D	245.15361	245.15471	4.487
E	233.15361	233.15065	12.695
F	231.13796	231.13924	5.538
G	219.13796	219.13683	5.157
H	217.12231	217.12043	8.659
I	193.12231	193.12296	3.366
J	71.04914	71.04885	4.082

We were unable to structurally confirm the presence or absence of 8'-hydroxy-CBC, 1''-hydroxy-CBC, and 6', 7'-dihydroxy-CBC as initially identified by Roy et al. [35] utilizing a CBC fragmentation pattern generated using combinatorial ion trap and high-resolution TOF mass spectrometry. However, we confirmed the presence of 6', 7'-epoxy-CBC via a unique α-cleavage fragment (exact mass 71.04914 Da) and supporting fragments (Figure S2.1.27, S2.1.28, and Table S2.1.2).

References

35. Roy P, Maturano J, Hasdemir H, Lopez A, Xu F, Hellman J, et al. Elucidating the Mechanism of Metabolism of Cannabichromene by Human Cytochrome P450s. J Nat Prod. 2024. doi: 10.1021/acs.jnatprod.3c00336.
