# Supplementary material for: Identification and Characterization of Cannabichromene’s Major Metabolite Following Incubation with Human Liver Microsomes
Source: Metabolites. 2024 Jun 13;14(6):329. doi: 10.3390/metabo14060329 (PMC11206029; doi:10.3390/metabo14060329)
Supplement: Supplementary file 1 [file metabolites-14-00329-s001.zip › CBC_Metabolite_Supplementary_Materials_S3.pdf]

Supplemental Materials S3

Identification and Characterization of Cannabichromene's Major Metabolite Following Incubation with Human Liver Microsomes

Alexandra M. Ward[1], Touraj Shokati[2], Jost Klawitter[2], Jelena Klawitter[2], Vu Nguyen[1], Laura Kozell[3, 4, 5], Atheir I. Abbas[3, 4, 5], David Jones[6], and Uwe Christians[2,*]

[1]*Department of Pharmaceutical Sciences, Skaggs School of Pharmacy and Pharmaceutical Sciences, University of Colorado Anschutz Medical Campus, Aurora, CO, USA;* *alexandra.ward@cuanschutz.edu*, *vu.t.nguyen@cuanschutz.edu*.

[2]*iC42 Clinical Research and Development, Department of Anesthesiology, School of Medicine, University of Colorado Anschutz Medical Campus, Aurora, CO, USA;* *touraj.shokati@cuanschutz.edu*, *jost.klawitter@cuanschutz.edu*, *jelena.klawitter@cuanschutz.edu*, *uwe.christians@cuanschutz.edu*.

[3]*Department of Behavioral Neuroscience, Oregon Health & Science University, Portland, OR, USA;* *abbasat@ohsu.edu*, *kozellla@ohsu.edu*.

[4]*Department of Psychiatry, Oregon Health & Science University, Portland, OR, USA.*

[5]*Veterans Affairs Portland Health Care System, Portland, OR, USA.*

[6]*Department of Pharmacology, School of Medicine, University of Colorado Anschutz Medical Campus, Aurora, CO, USA;* *david.jones@cuanschutz.edu*.

*Correspondence: uwe.christians@cuanschutz.edu

Table of Contents Supplemental Materials S3

S3.1
Purity of 2’-hydroxycannabicitran Page 3

S3.2
Cannabichromene NMR Experiments Page 12

S3.3
2’-hydroxycannabicitran NMR Experiments Page 29

S3.4
Cannabicitran and Overlay NMR Experiments Page 51

References Page 62

S3.1

Purity of 2'-hydroxycannabicitran

Prior to running NMR experiments on the synthetically generated metabolite of CBC, we first established the purity of the isolated compound via UV detection and a TOF MS scan. We quantified the metabolite using NMR. Interestingly, we compared this quantification to UV detection methods at two differing wavelengths using CBC as the standard. Neither wavelength measured was representative of the concentration determined from NMR.

We also observed reactivity of the metabolite in $CDCl_3$, and therefore ACN-d_3 was chosen as the NMR solvent for analysis.

Please see supplementary Figure S3.1.1 for the UV and TOF-MS purity of the synthetically generated 2'-hydroxycannabicitran used in NMR analysis. Figure S3.1.2 displays the NMR generated calibration curve used for metabolite quantification, and Figures S3.1.3-S3.1.4 and Tables S3.1.1-S3.1.2 display the comparative UV-generated calibration curves.

Figures S3.1.5-7 and Tables S3.1.3 and S3.1.4 display high-resolution MS data detailing the metabolite reactivity with $CDCl_3$.

Materials and Methods

Additional materials for NMR quantification were deuterium oxide (D_2O) 99.9 atom% D purchased from Thermo Scientific, Waltham, MA, USA and 3-(Trimethylsilyl)propionic-2, 2, 3, 3-d_4 acid sodium salt (TMSP) 98 atom% D, ≥98.0% purchased from Fisher Scientific, Waltham, MA, USA.

Following isolation, 1:100 diluted fractions of synthetically generated 2'-hydroxycannabicitran were checked for purity utilizing HPLC/MS-TOF. The system consisted of the following: 3000 Ultima HPLC components (all Dionex, Thermo Fischer, Palo Alto, CA, USA): WPS-3000 (RS) autosampler, DGP-3600 Pump, Ultimate 3000 Variable Wavelength Detector monitored at 231 nm, and FLM-3000 Flow Manager oven. The mass spectrometer was a 5600+ high-resolution TOF (AB Sciex, Concord, ON, Canada) operated in the positive electrospray ionization (ESI) mode. The HPLC components were controlled by Dionex Chromeleon version 6.80.14.4527 (Thermo Fisher Scientific, Waltham, MA, USA) and the mass spectrometer was controlled by Analyst Software version TF1.8.1 (AB Sciex, Concord, ON, Canada); resulting data was analyzed utilizing Analyst Software version TF1.8.1 (AB Sciex, Concord, ON, Canada).

The injection volume was 25 µL with the column temperature set at 60 °C. Mobile phase A was water + 0.1% formic acid and mobile phase B was acetonitrile + 0.1% formic acid. The pump gradient associated was: 0.0 min, 75% B; 1.0 min, 75% B; 14.0 min, 95% B; 17.0 min, 98% B; 21.0 min, 98% B; 21.1 min, 75% B; and 25.0 min, 75% B; the flow rate was 1000 µL/min throughout the gradient. The analytical columns were 2 x Eclipse XDB-C8 5 µm, 4.6 x 250 mm in series (Agilent Technologies, Santa Clara, CA, USA).

For NMR quantification the following method was used: ^{1}H one dimensional NMR experiments were

collected over a spectral width of 16 ppm using a total of 64K complex data points (R+I) giving an acquisition time of 3.14 s per fid with a 30 s relaxation delay between transients to ensure complete relaxation for accurate integration. There were 8-16 transients per experiment. The instrument was a 600 MHz Bruker Avance Neo spectrometer (Bruker, Billerica, MA, USA). The solvent used for all calibration levels was acetonitrile-d_3.

For UV CBC calibration curves, the following method was used: G1322A degasser, G1312A binary pump, G1329B 1260 ALS, G1315B DAD, (all Agilent Technologies, Santa Clara, CA, USA) and an external column compartment ThermaSphere (Phenomenex, Torrance, CA, USA). The HPLC-DAD system was controlled and data was processed using ChemStation software revision 04.03.087 (Agilent Technologies, Santa Clara, CA, USA). The analytical columns were 2 x Eclipse XDB-C8 5 µm, 4.6 x 250 mm in series (Agilent Technologies, Santa Clara, CA, USA). The flow rate was 1 mL/min and the mobile phases were HPLC grade water (mobile phase A) and HPLC grade acetonitrile (mobile phase B). The elution gradient for was 0.0 min, 80% B; 10.0 min, 80% B; 22.0 min, 99% B; 22.5 min, 99% B; 22.6 min, 80% B; and 30.0 min, 80% B. The injection volume was 25 µL; UV absorbance was monitored at 210 nm and 231 nm.

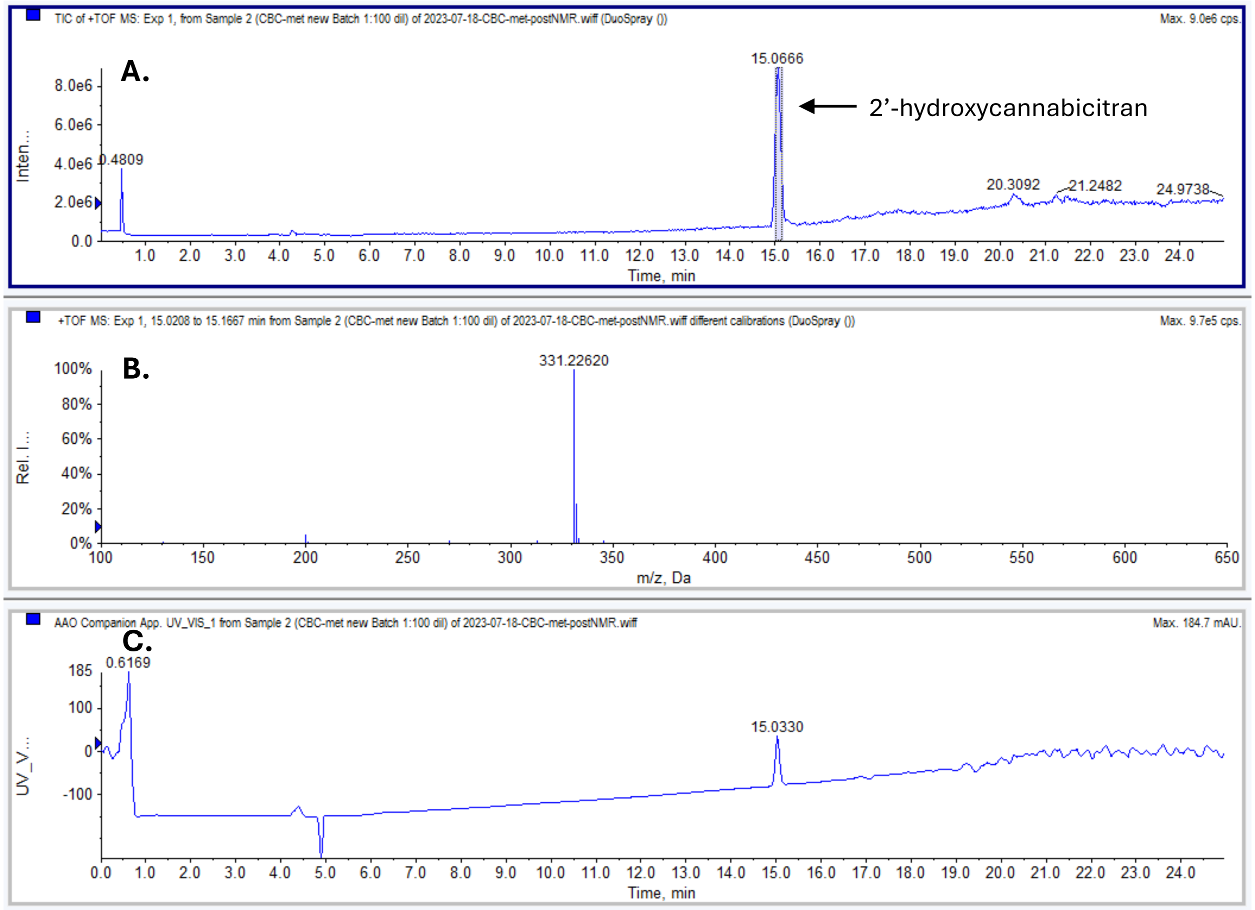

Figure S3.1.1. Purity of 2’-hydroxycannabicitran subject to NMR analysis in acetonitrile-d_3. (A) total ion chromatogram from scan mode (*m/z* = 100-650), (B) mass spectrum of the predominant peak in (A), and (C) UV chromatogram measured at 231 nm.

The total ion chromatogram scan and UV spectra display the synthesized major metabolite of CBC (2’-hydroxycannabicitran) is 100% pure.

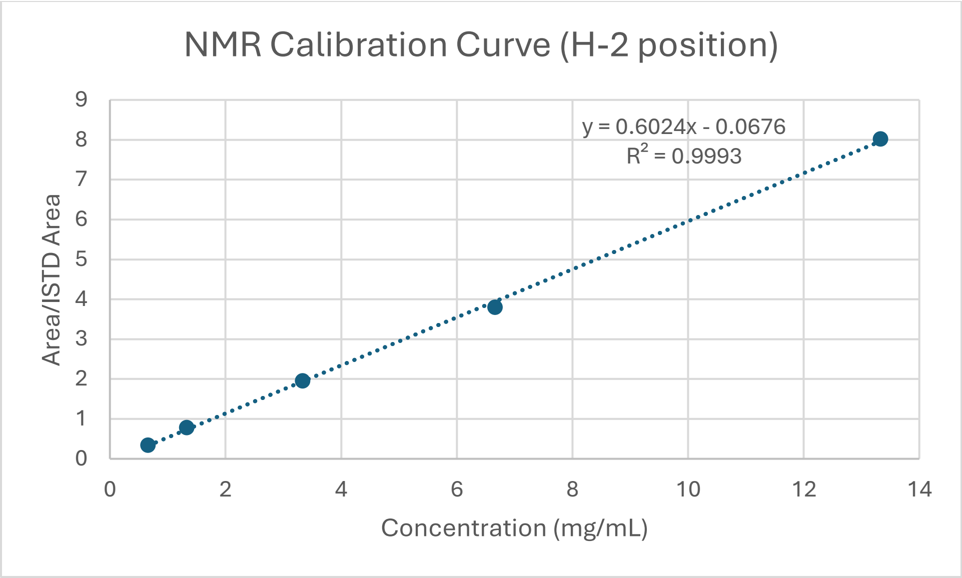

Figure S3.1.2. NMR calibration curved based on the H-2 position of CBC at various concentrations.

We used ACN-d_3 as the solvent for these experiments and inserted a sealed capillary containing 50 mM TMSP (in D_2O). We determined the CBC Area of the specific proton H-2 and divided by the area of internal standard. We placed the capillary in the 2'-hydroxycannabichromene sample to determine its concentration of 2.23 mg/mL using the calibration curve in Figure S3.1.2.

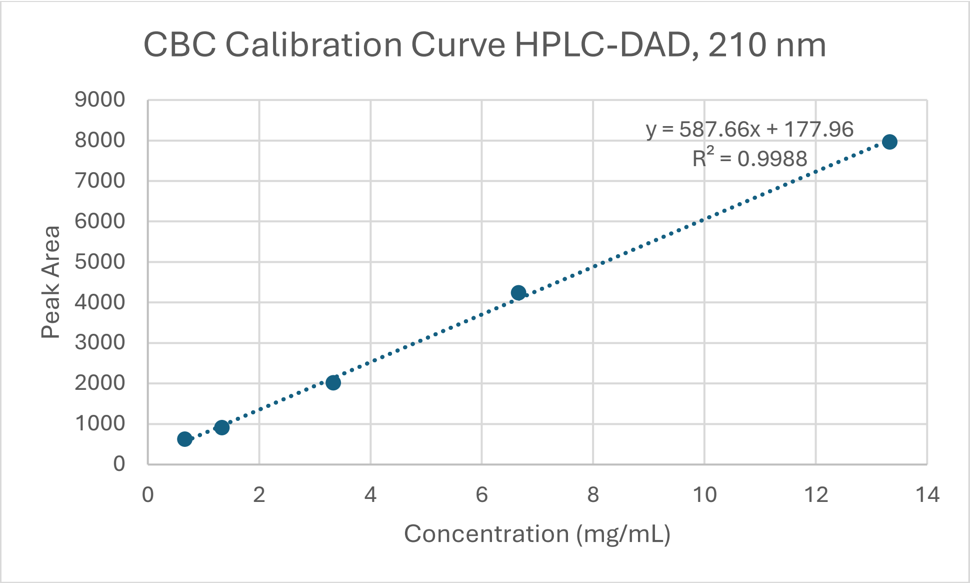

Figure S3.1.3. Calibration curve generated based on the UV signal of CBC measured at 210 nm.

Table S3.1.1. Comparison of calculated concentrations of 2'-hydroxycannacitran based on UV detection at 210 nm and NMR signal of H-2.

	Concentration (mg/mL)
[2'-hydroxycannabicitran] UV @210 nm	4. 60
[2'-hydroxycannabicitran] NMR	2.23
multiplication factor	0.49

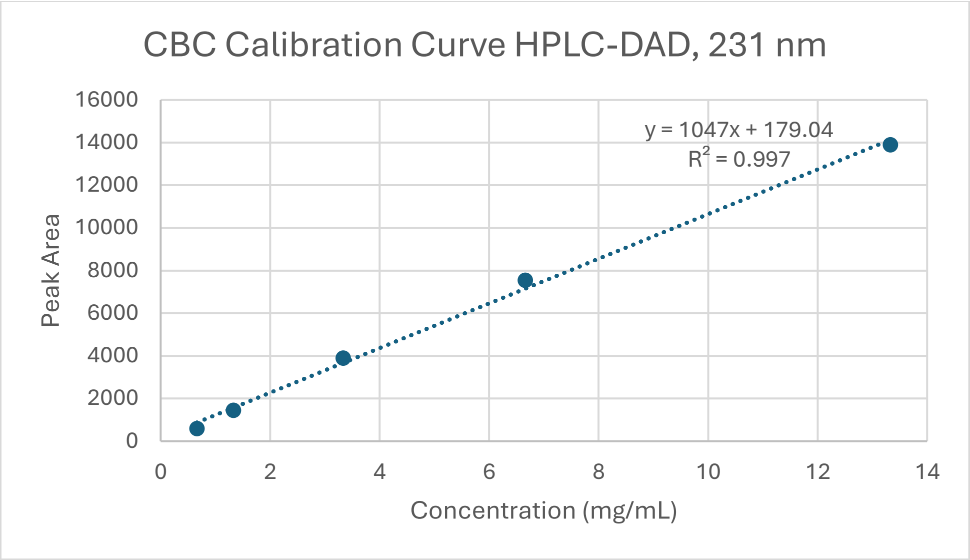

Figure S3.1.4. Calibration curve generated based on the UV signal of CBC measured at 231 nm.

Table S3.1.2. Comparison of calculated concentrations of 2'-hydroxycannacitran based on UV detection at 231 nm and NMR signal of H-2.

	Concentration (mg/mL)
[2'-hydroxycannabicitran] UV @231 nm	0.50
[2'-hydroxycannabicitran] NMR	2.23
multiplaction factor	4.46

The same samples were used to generate all calibration curves. We compared calibration curves of CBC analyzed via HPLC-DAD and determined these curves alone do not accurately convey the concentration of 2'-hydroxycannabicitran in solution. Multiplicative factors were determined from the NMR calibration curve.

When attempting to quantify 2'-hydroxycannabichromene with an external CBC UV-based calibration curve, measuring at 210 nm will overestimate the actual concentration by a factor of 2.06 and measuring at 231 nm will underestimate the actual concentration by a factor of 4.46.

This is likely due to a shift in the UV spectrum compared to CBC following the formation of 2'-hydroxycannabicitran.

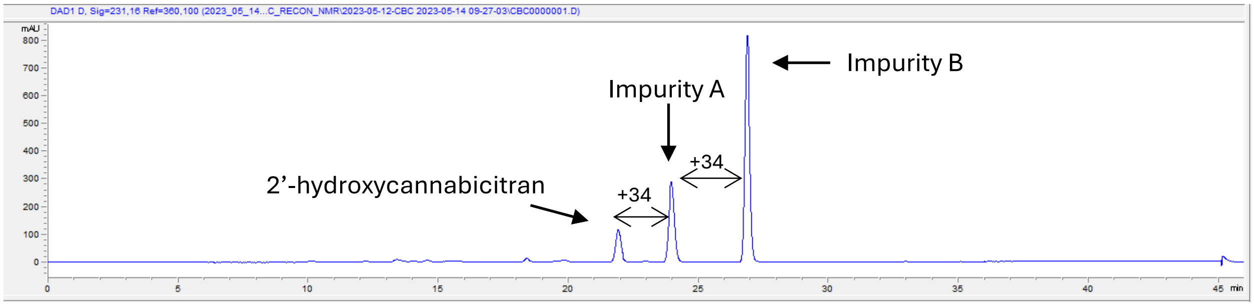

Figure S3.1.5. UV chromatogram of isolated 2’-hydroxycannabicitran after being reconstituted in $CDCl_3$. *m/z* was determined from mass spectrometry: 2’-hydroxycannabicitran *m/z* = 331.2, impurity A *m/z* = 365.2, and impurity B *m/z* = 399.1.

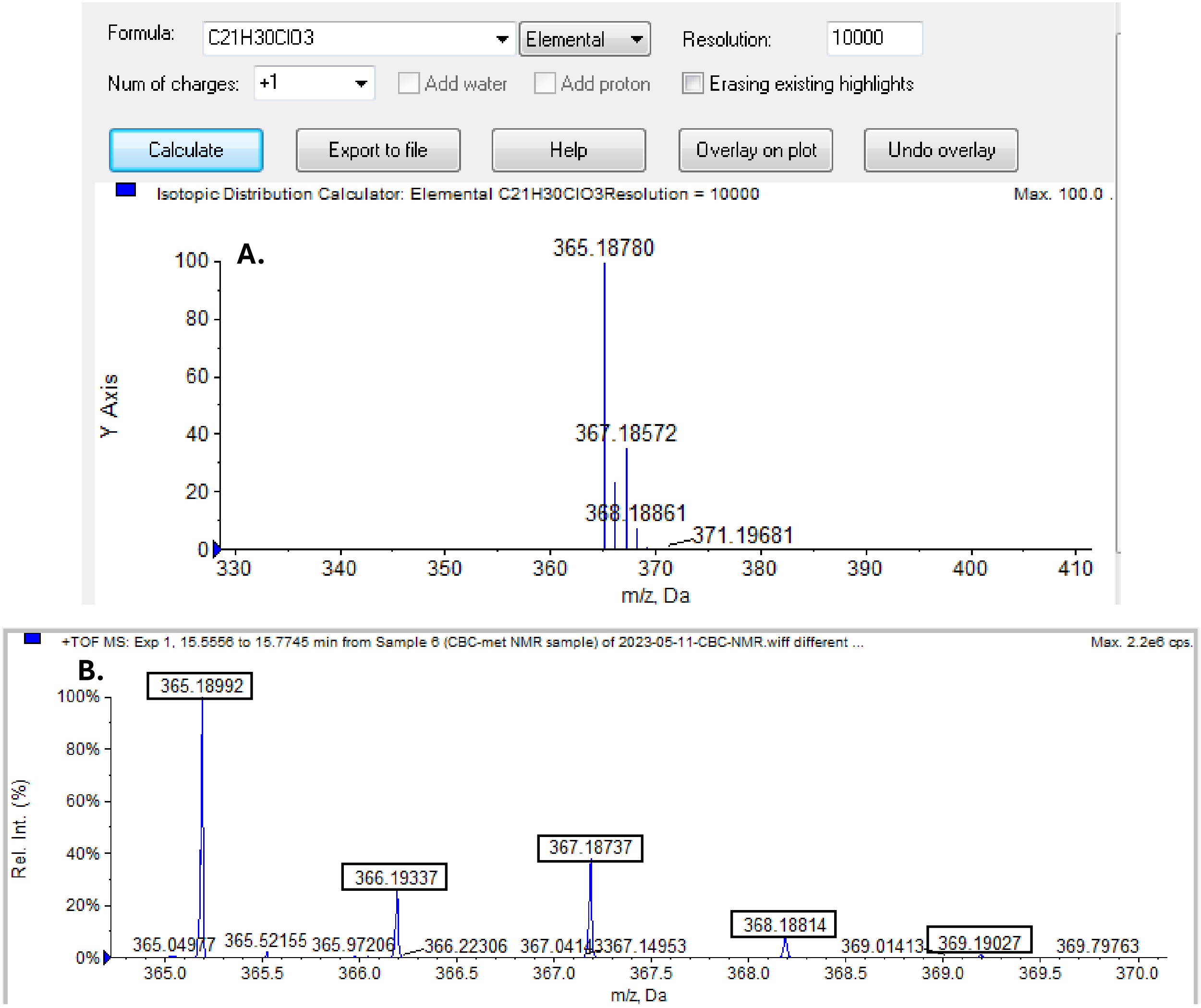

Figure S3.1.6. Analysis of Impurity A on HPLC-MS/TOF. (A) Theoretical isotopic pattern for $C_{21}H_{30}ClO_3^+$ and (B) experimental isotopic pattern of Impurity A.

Table S3.1.3. Comparison of theoretical and measured exact mass of Impurity A, $C_{21}H_{30}ClO_3^+$.

Theoretical		Measured		Δppm
m/z (Da)	%max. intensity	*m/z* (Da)	%max. intensity	
365.18780	100	365.18992	100	5.7505
366.19120	23.77724	366.19337	26.3104	6.0078
367.18572	35.27665	367.18737	37.8494	4.5753
368.18861	7.94044	368.18814	8.2499	1.3852
369.19139	1.08206	369.19027	1.0872	2.9524

Characteristic evidence of a chlorine atom by $[M]^+$ and $[M+2]^+$ in a 3:1 ratio respectively, indicating Impurity A is the result of a chlorine addition to 2'-hydroxycannabicitran.

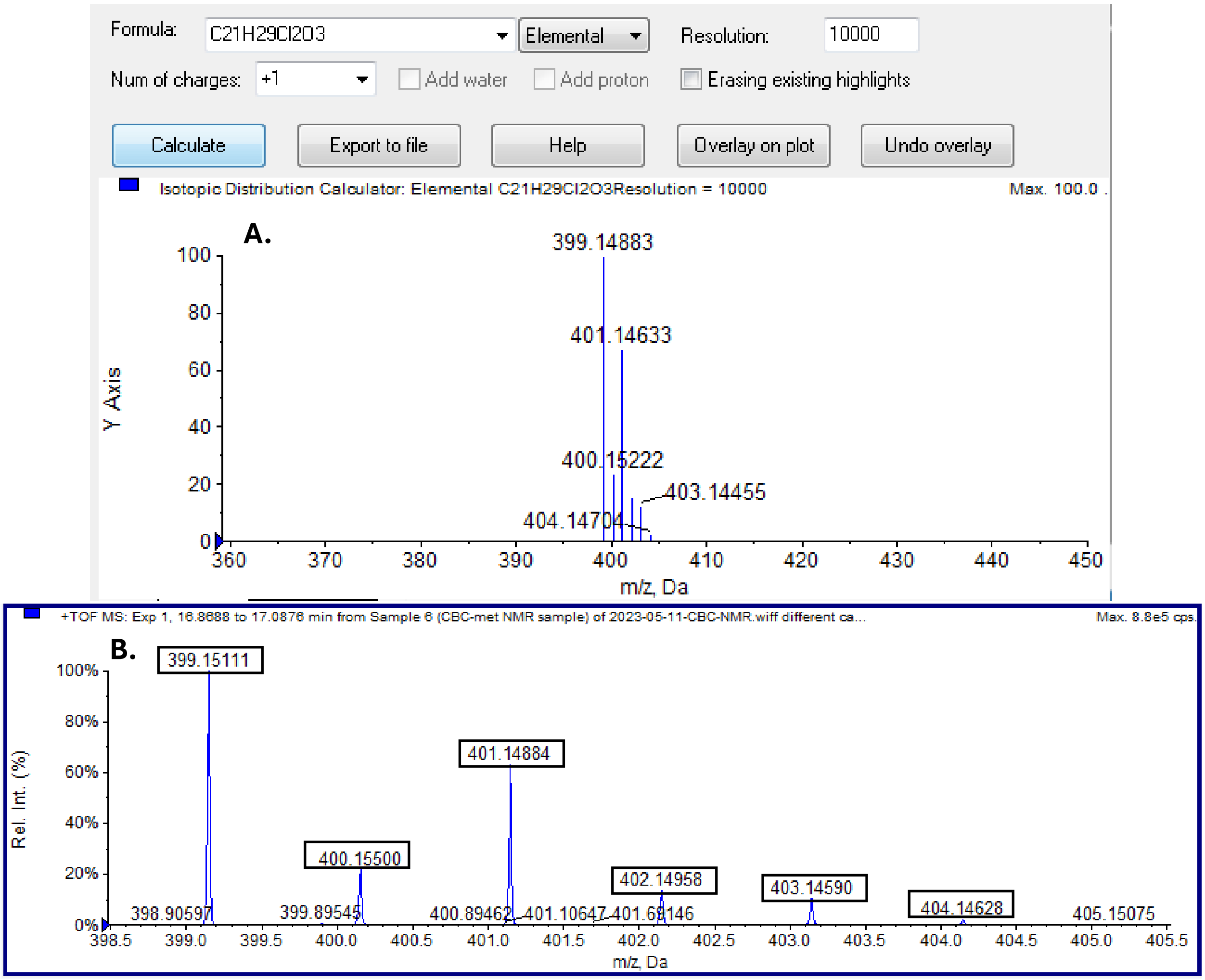

Figure S3.1.7. Analysis of Impurity A on HPLC-MS/TOF. (A) Theoretical isotopic pattern for $C_{21}H_{29}Cl_2O_3^+$ and (B)experimental isotopic pattern of Impurity B.

Table S3.1.4. Comparison of theoretical and measured exact mass of Impurity A, $C_{21}H_{29}Cl_2O_3^+$.

Theoretical		Measured		Δppm
m/z (Da)	%max. intensity	*m/z* (Da)	%max. intensity	
399.14883	100	399.15111	100	5.6871
400.15222	23.76724	400.15500	21.7283	6.9474
401.14633	67.25263	401.14884	63.4462	6.1574
402.14946	15.53729	402.14958	13.8390	0.3481
403.14455	12.36140	403.14590	10.1599	3.3487
404.14704	2.64754	404.14628	2.19150	1.8310

Characteristic evidence of 2 chlorine atoms by $[M]^+$, $[M+2]^+$, and $[M+4]^+$ in a 9:6:1 ratio respectively, indicating Impurity B is the result of the addition of 2 chlorine atoms to 2’-hydroxycannabicitran.

S3.2

Cannabichromene NMR Experiments

A ^{1}H NMR spectrum of CBC was analyzed in $CDCl_3$ for comparison purposes with the literature [46]. Otherwise, CBC was comprehensively examined in ACN-d_3. The basis of this was to then provide a foundation for comparison with the major metabolite of CBC. Figures include NMR parameters, full spectra, and zoomed-in spectra with highlighted signals.

Please see the supplementary figures described below:

Chemical structure and numbering assignment of CBC: Figure S.3.2.1

^{1}H in $CDCl_3$: Figures S3.2.2- S3.2.4

^{1}H in ACN-d_3: Figures S3.2.5- S3.2.7

^{13}C in ACN-d_3: Figures S3.2.8- S3.2.11

COSY in ACN-d_3: Figures S3.2.12- S3.2.15

HSQC in ACN-d_3: Figures S3.2.16- S3.2.18

HMBC in ACN-d_3: Figures S3.2.19- S3.2.25

Methods

^{1}H one dimensional NMR experiments were collected over a spectral width of 16 ppm using a total of 64K complex data points (R+I) giving an acquisition time of 3.14 s per fid with a 30 s relaxation delay between transients to ensure complete relaxation for accurate integration. There were 8-16 transients per experiment.

^{13}C one dimensional experiments were collected using 1024 transients over a spectral width of 216 ppm with 64K complex data points (R+I) giving an acquisition time of 1.002 s per fid with a relaxation time of 2 s between transients. ^{1}H decoupling was achieved using waltz16 with a B1 field strength of 3.6 KHz centered at 4.00 ppm ^{1}H.

^{1}H-^{13}C Heteronuclear Single Quantum Coherence (HSQC) spectra were acquired using 2048 data points (R+I) over a spectral width of 9.8 ppm in the ^{1}H dimension and 256 data points (R+I) over 165 ppm in the C13 dimension. The transmitter offsets were centered at 4.0 ppm and 75 ppm respectively. 16 transients were acquired per FID using a 2 s relaxation delay. C13 decoupling during acquisition was achieved using GARP with a ^{13}C B1 field strength of 4.2 KHz centered at 75 ppm.

For ^{1}H-^{13}C Heteronuclear Multiple Bond Correlation (HMBC) experiments, the spectral widths were 9.8 and 200 pm respectively in the ^{1}H and ^{13}C dimensions and used a total of 4096 by 512 complex data points. The transmitter offsets were set at 4.0 ppm in ^{1}H and 100 ppm in the ^{13}C dimensions. 32 transients were collected per FID using a 2 s relaxation delay between transients.

^{1}H-^{1}H gradient selected Double Quantum Filtered-Homonuclear Correlation Spectroscopy (DQF-COSY) experiments were collected with spectral widths of 10 ppm in both dimensions, with the transmitter centered at 4.0 ppm using 4K data points in the acquired dimension and 1K data points in the indirect dimension.

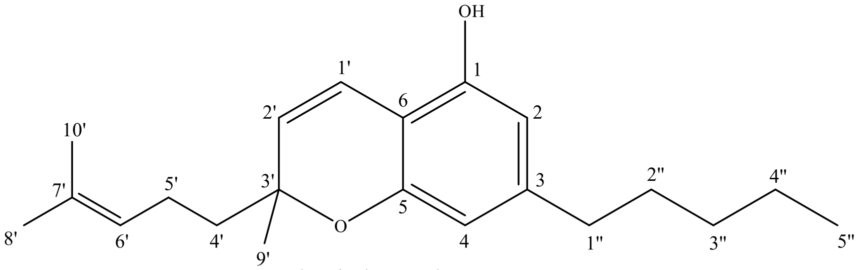

Chemical Formula: $C_{21}H_{30}O_2$
Exact Mass: 314.22458

Figure S3.2.1. Numbered structure of CBC according to a terpenoid system of numbering [36, 37].

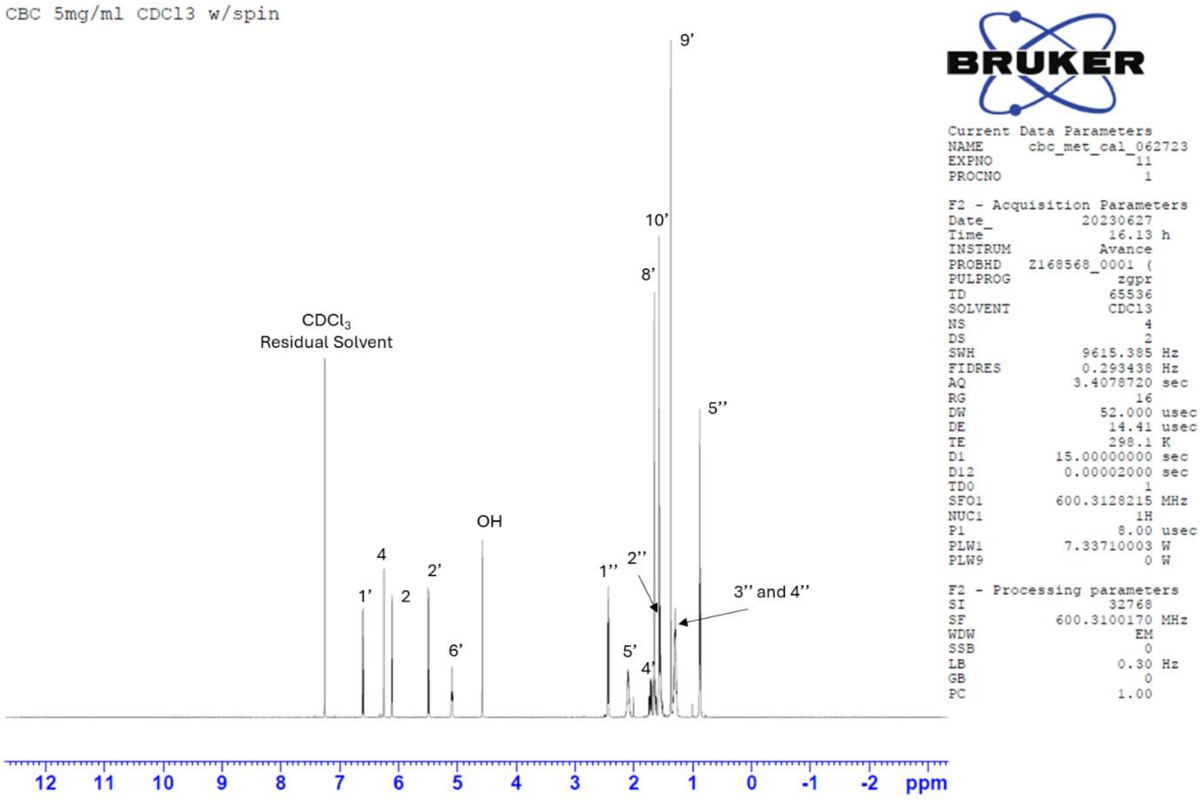

Figure S3.2.2. ^{1}H NMR spectrum of CBC in $CDCl_3$. Assignments based on the structure in Figure S3.2.1.

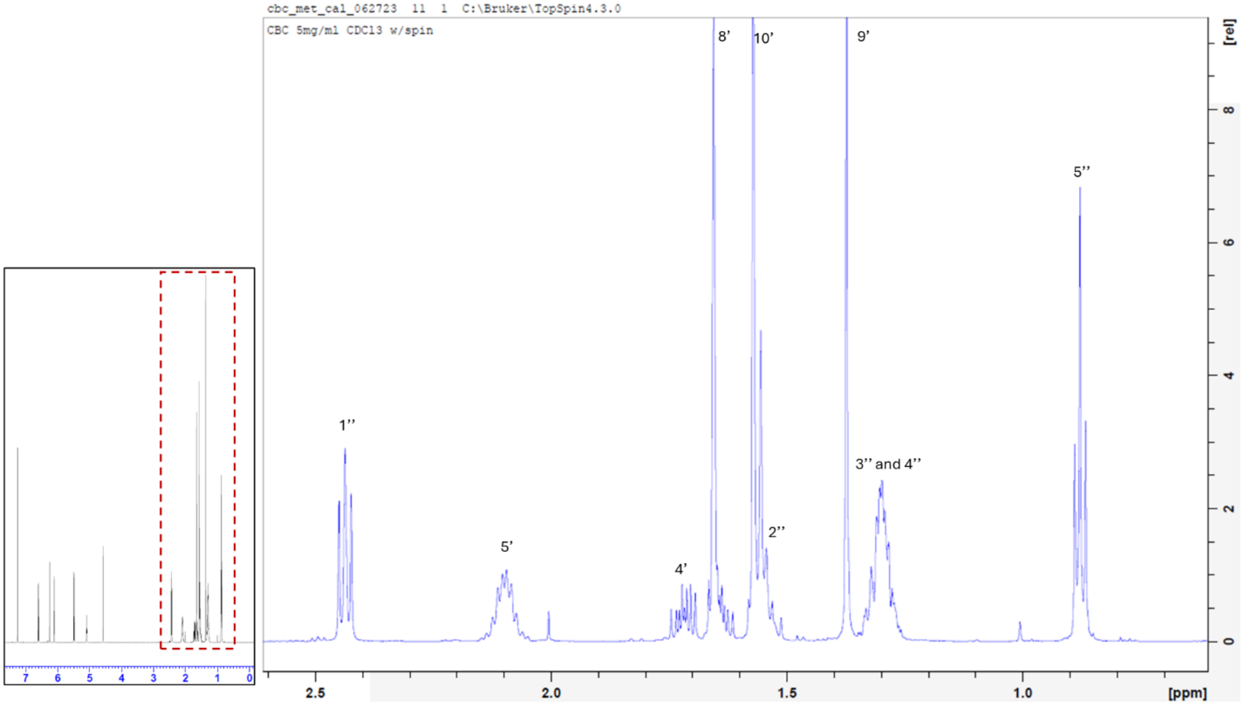

Figure S3.2.3. Zoomed-in ^{1}H NMR spectrum of CBC in $CDCl_3$. Assignments based on the structure in Figure S3.2.1. The red dotted line in the inset shows the zoomed-in range of the full spectrum.

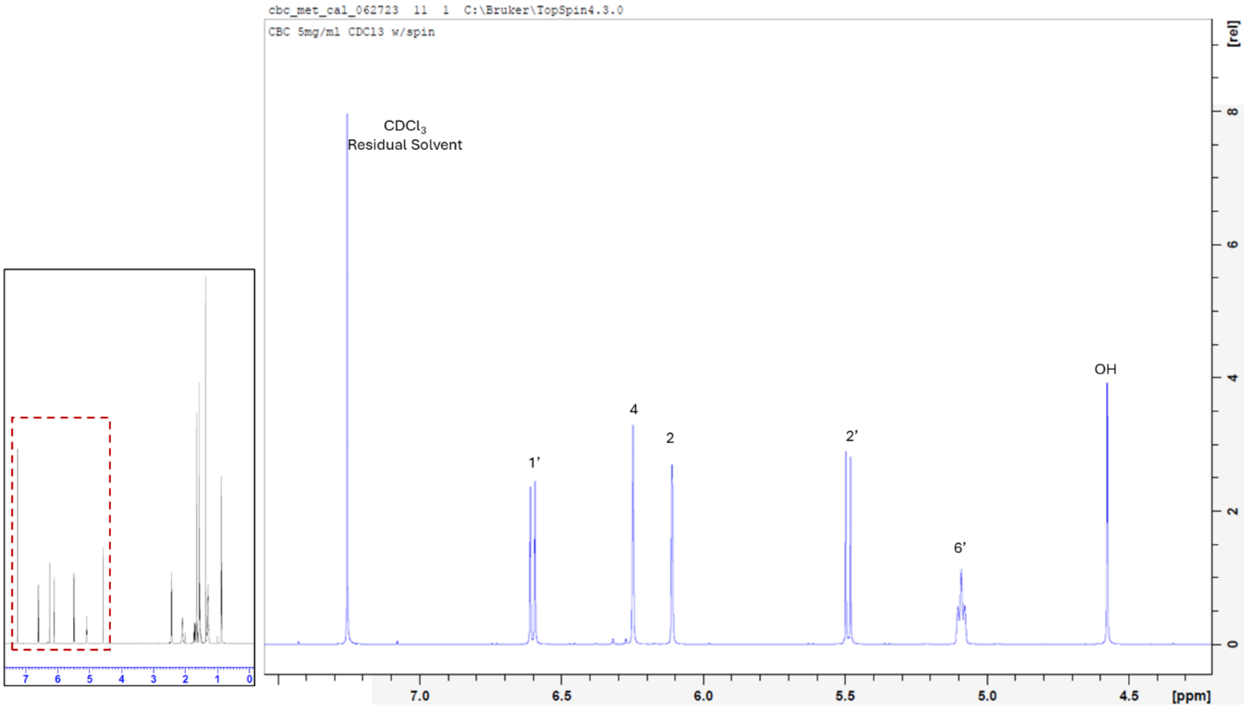

Figure S3.2.4. Zoomed-in ^{1}H NMR spectrum of CBC in $CDCl_3$. Assignments based on the structure in Figure S3.2.1. The red dotted line in the inset shows the zoomed-in range of the full spectrum.

Multiplicity and chemical shifts of CBC in $CDCl_3$ is in agreement with a previously published report [46].

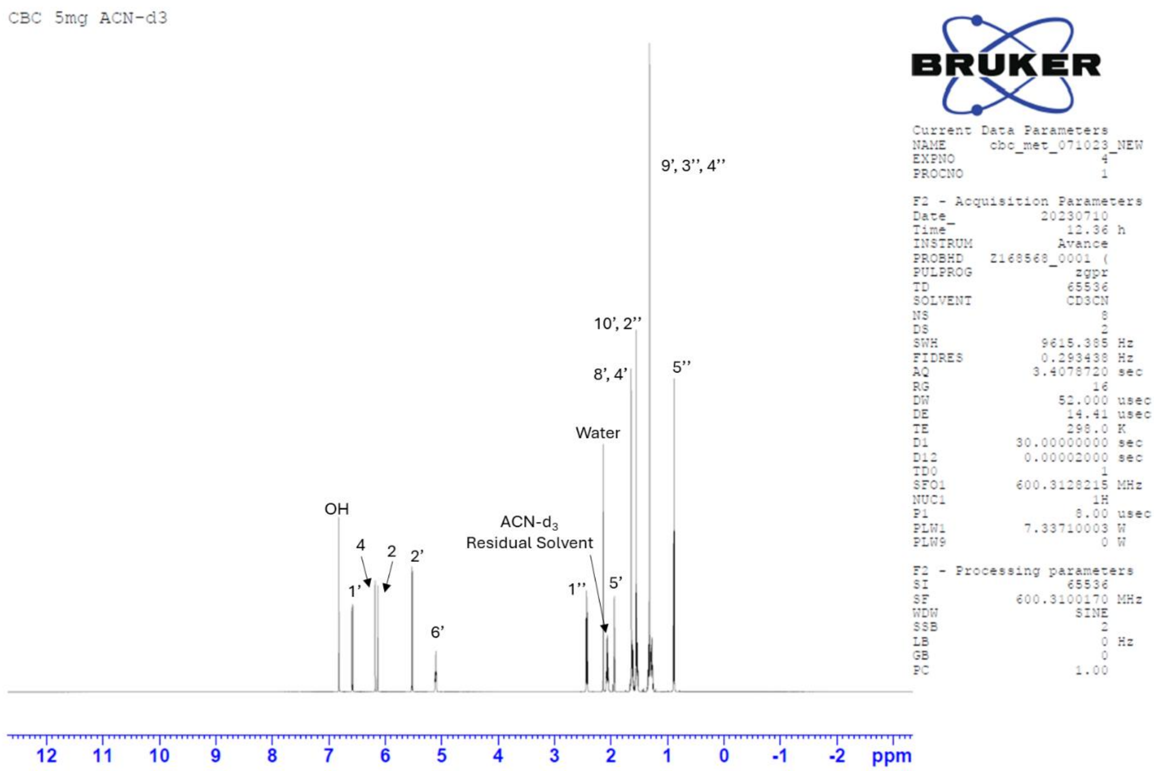

Figure S3.2.5. ^{1}H NMR spectrum of CBC in ACN-d_3. Assignments based on the structure in Figure S3.2.1.

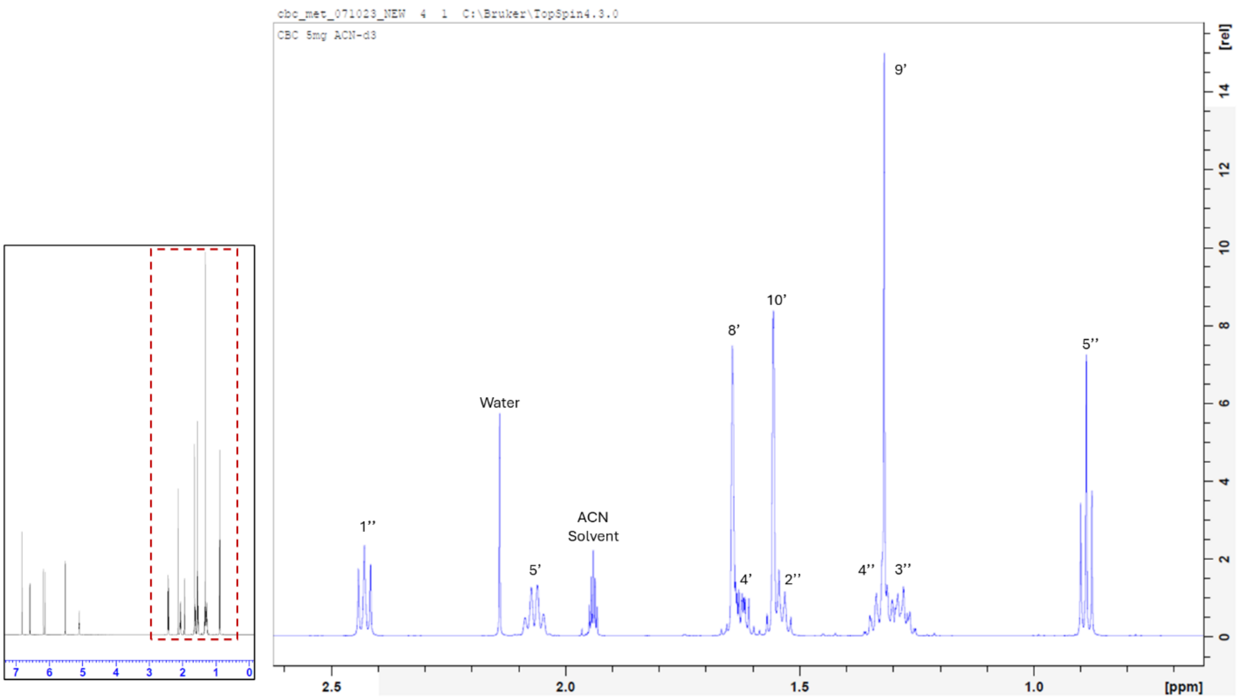

Figure S3.2.6. Zoomed-in ^{1}H NMR spectrum of CBC in ACN-d_3. Assignments based on the structure in Figure S3.2.1. The red dotted line in the inset shows the zoomed-in range of the full spectrum.

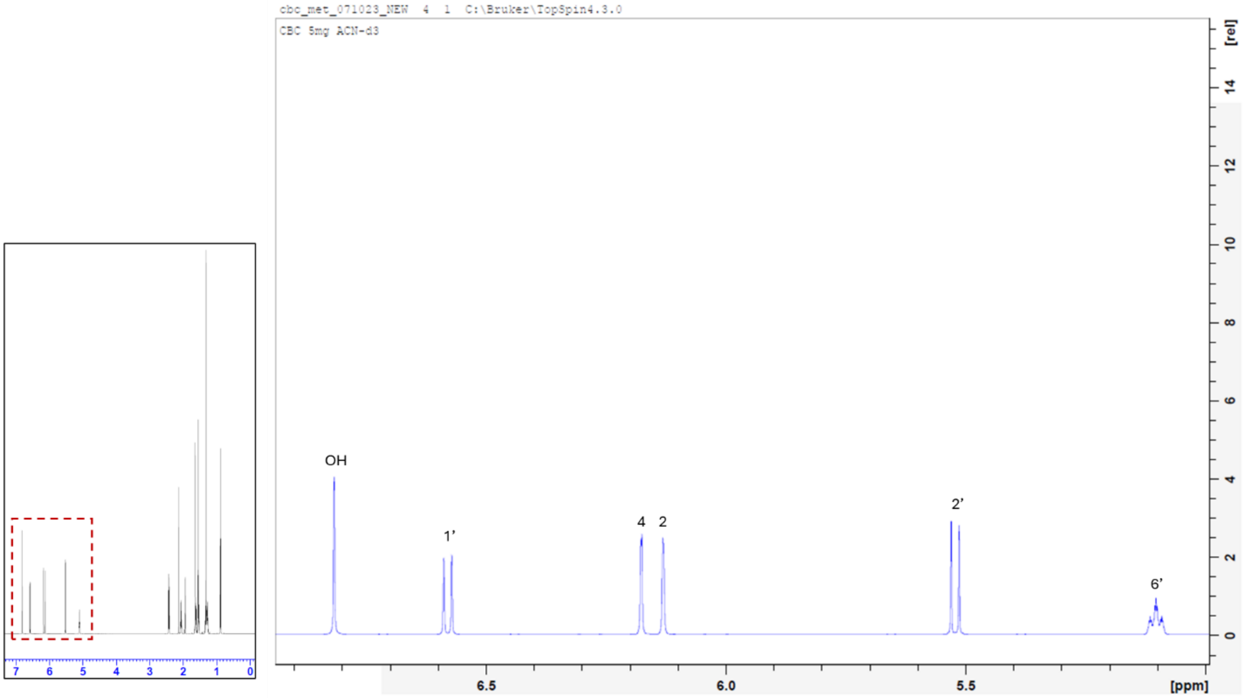

Figure S3.2.7. Zoomed-in ^{1}H NMR spectrum of CBC in ACN-d_3. Assignments based on the structure in Figure S3.2.1. The red dotted line in the inset shows the zoomed-in range of the full spectrum.

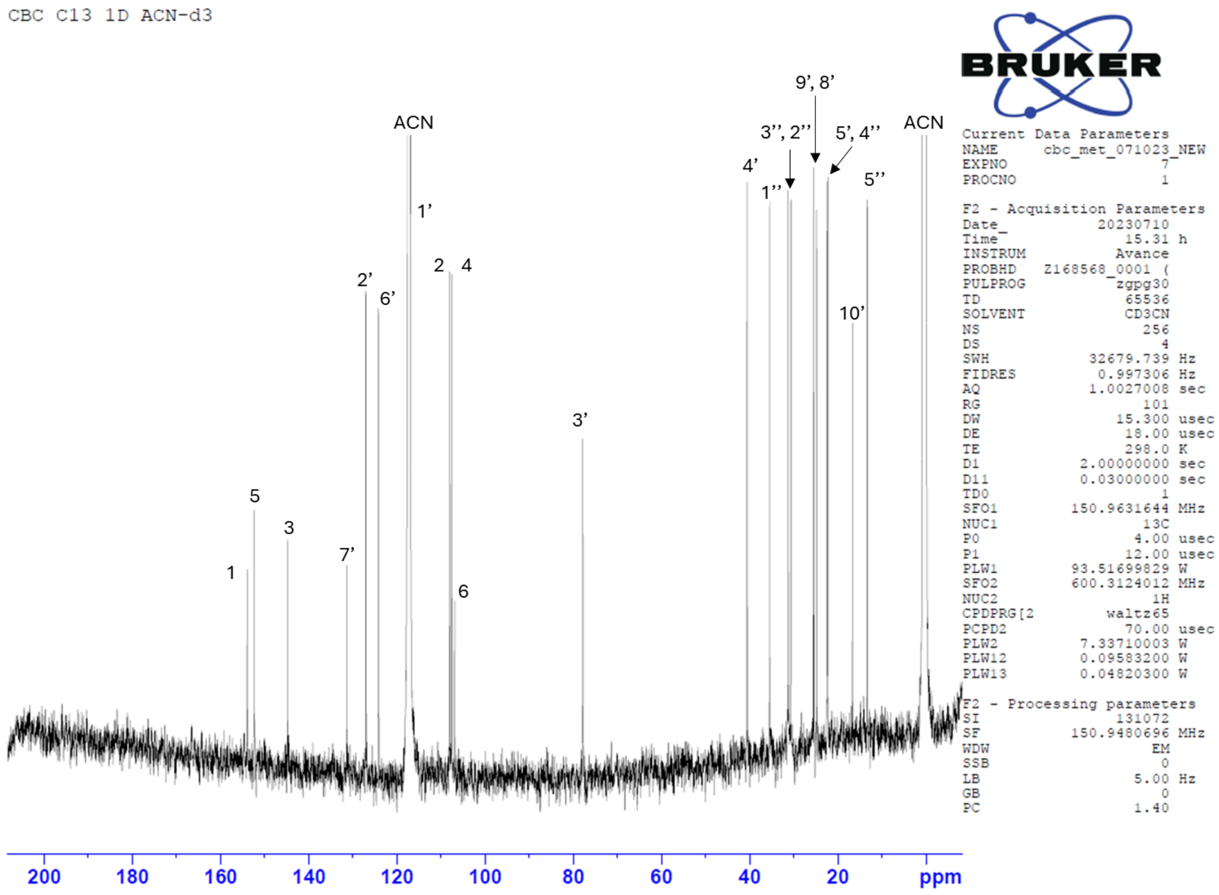

Figure S3.2.8. ^{13}C NMR spectrum of CBC in ACN-d_3. Assignments based on the structure in Figure S3.2.1.

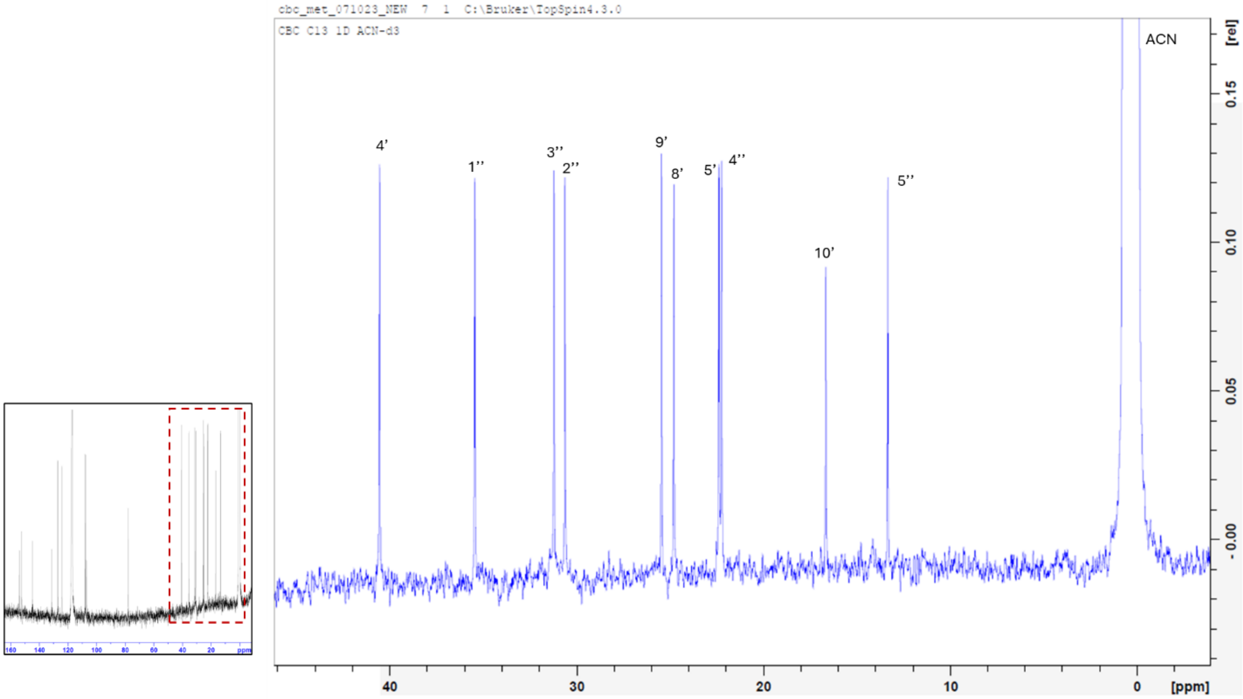

Figure S3.2.9. Zoomed-in ^{13}C NMR spectrum of CBC in ACN-d_3. Assignments based on the structure in Figure S3.2.1. The red dotted line in the inset shows the zoomed-in range of the full spectrum.

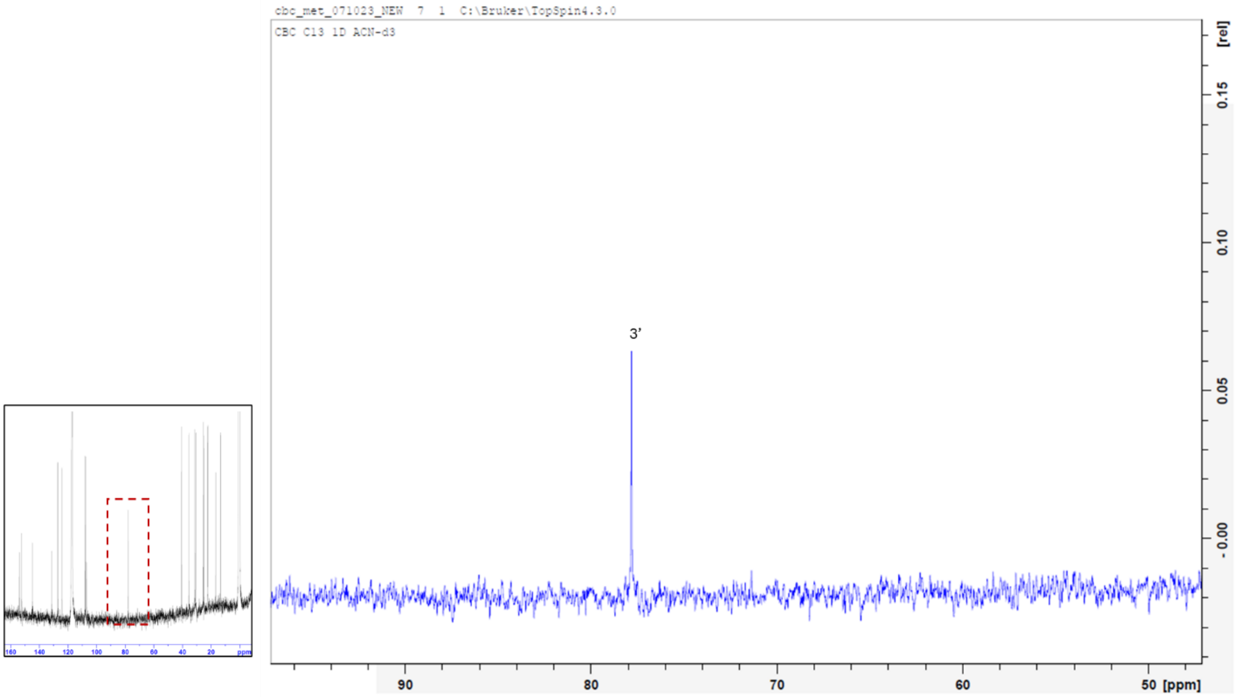

Figure S3.2.10. Zoomed-in ^{13}C NMR spectrum of CBC in ACN-d_3. Assignments based on the structure in Figure S3.2.1. The red dotted line in the inset shows the zoomed-in range of the full spectrum.

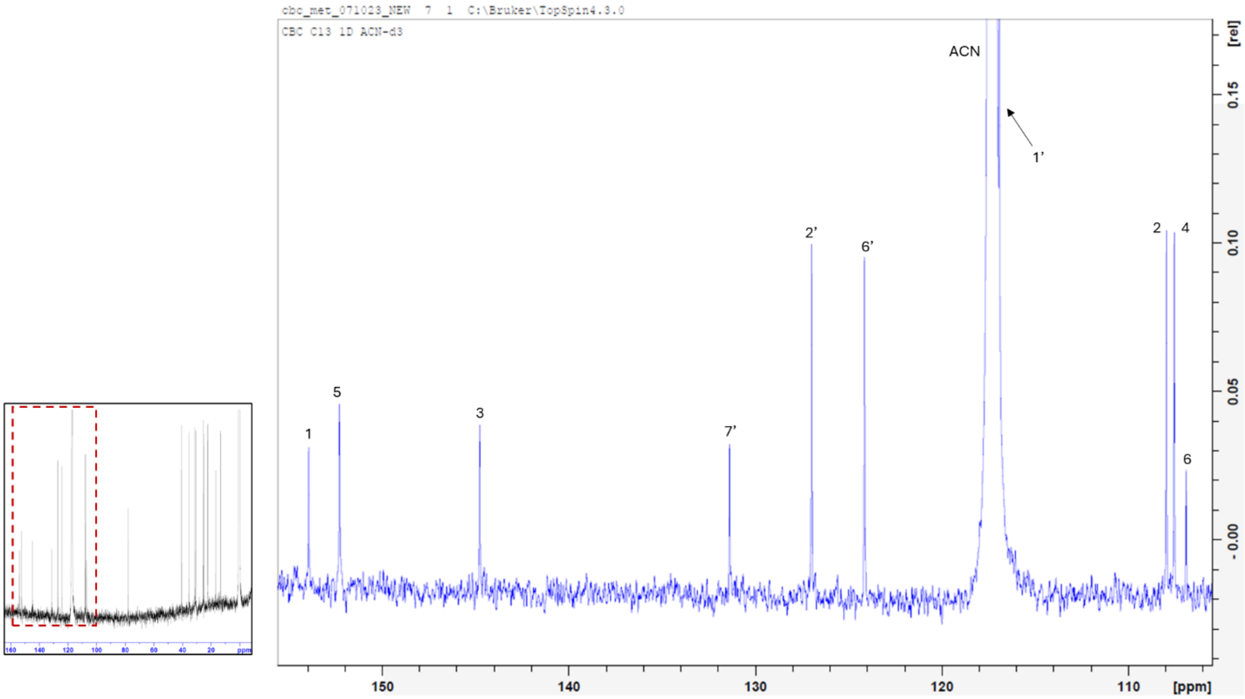

Figure S3.2.11. Zoomed-in ^{13}C NMR spectrum of CBC in ACN-d_3. Assignments based on the structure in Figure S3.2.1. The red dotted line in the inset shows the zoomed-in range of the full spectrum.

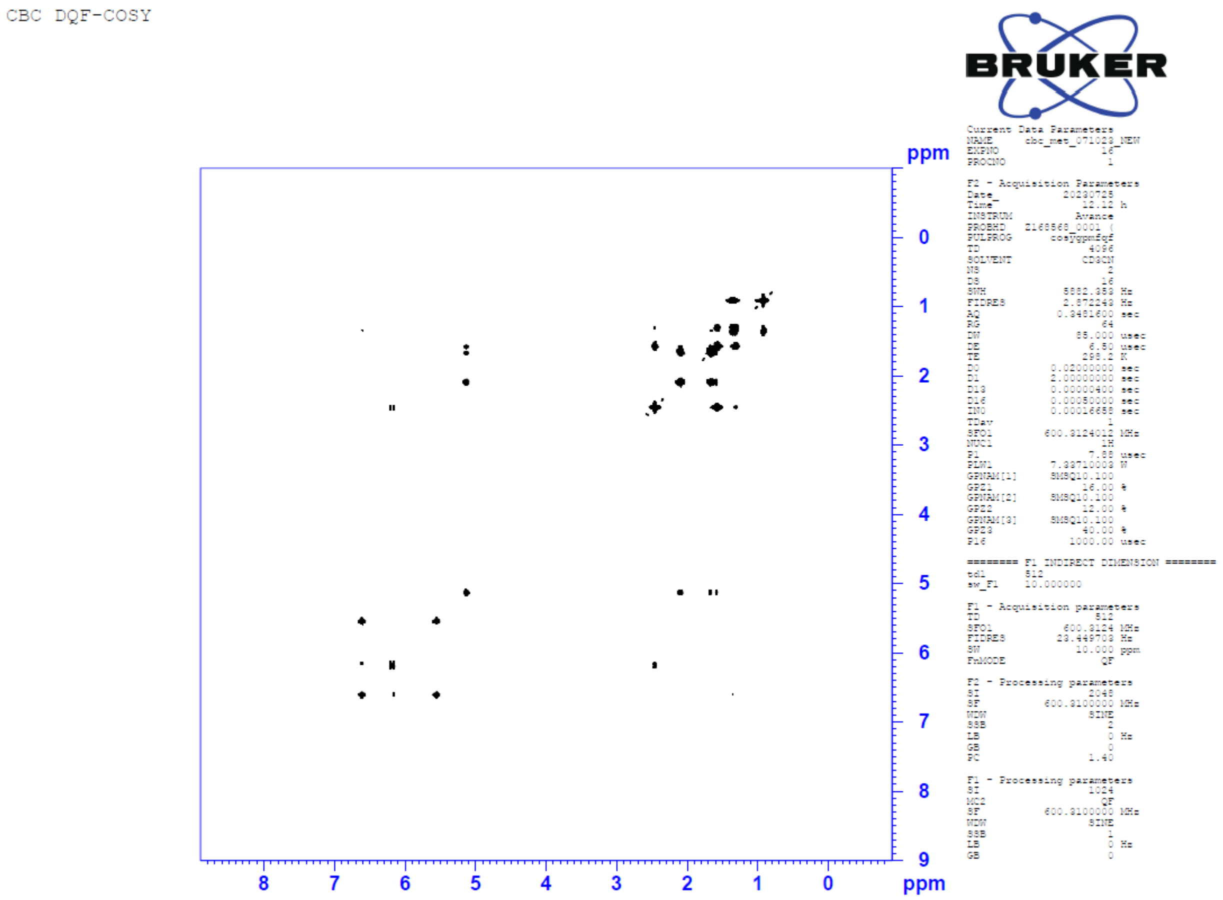

Figure S3.2.12. COSY NMR spectrum of CBC in ACN-d_3.

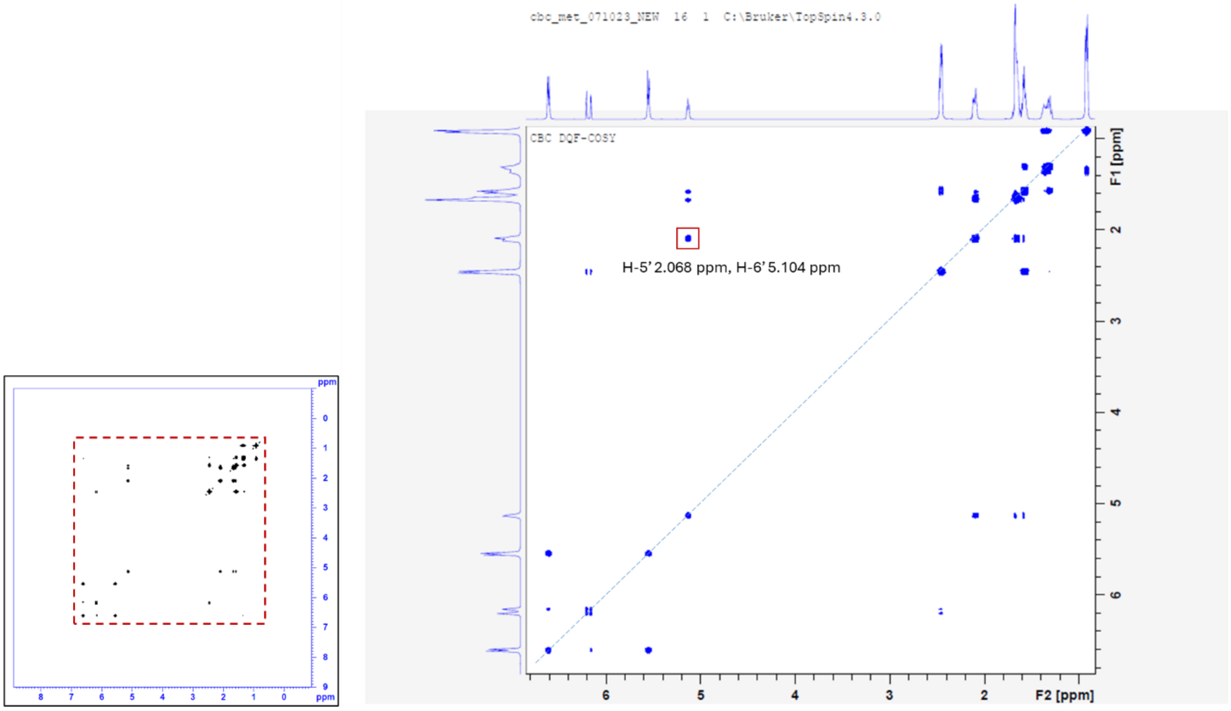

Figure S3.2.13. Zoomed-in COSY NMR spectrum of CBC in ACN-d_3. Assignments based on the structure in Figure S3.2.1. The red dotted line in the inset shows the zoomed-in range of the full spectrum.

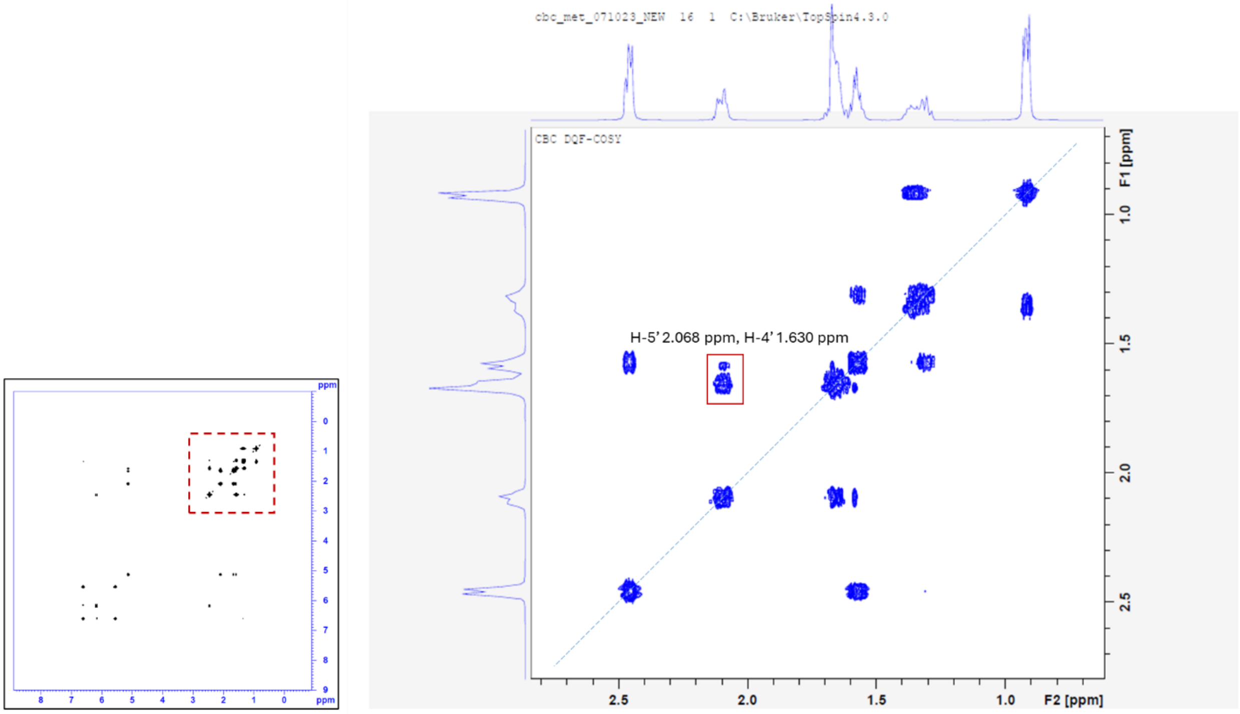

Figure S3.2.14. Zoomed-in COSY NMR spectrum of CBC in ACN-d_3. Assignments based on the structure in Figure S3.2.1. The red dotted line in the inset shows the zoomed-in range of the full spectrum.

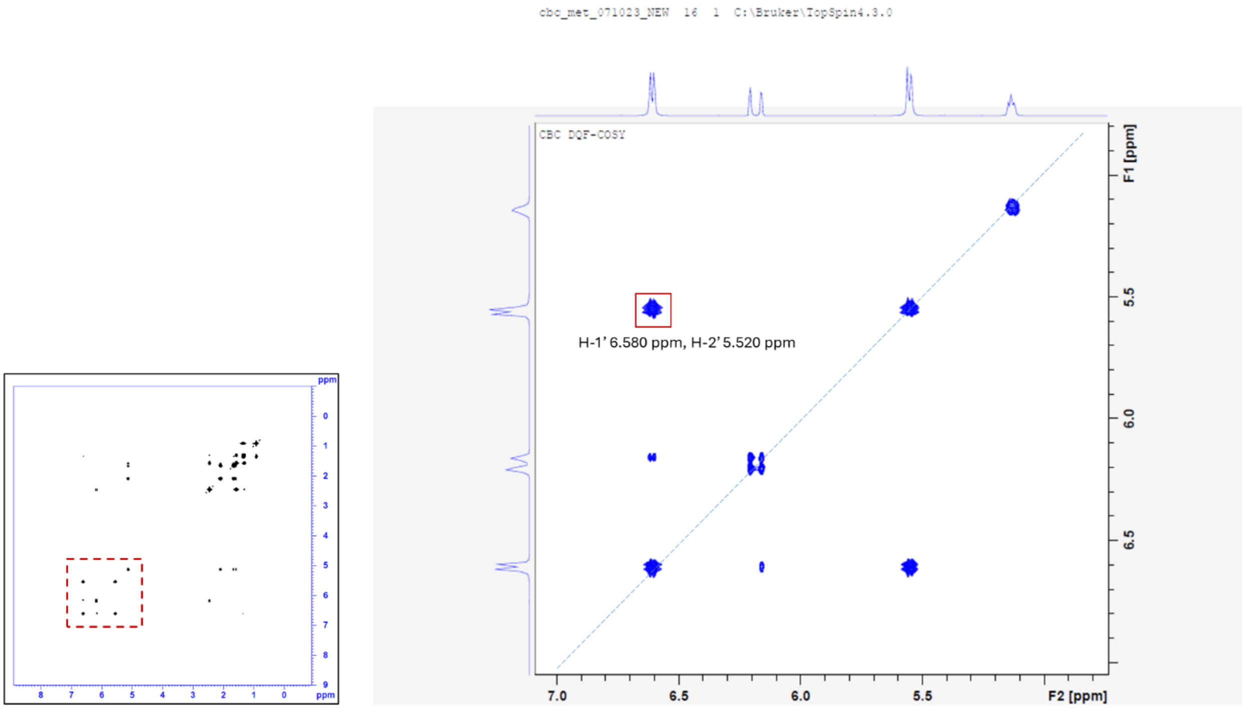

Figure S3.2.15. Zoomed-in COSY NMR spectrum of CBC in ACN-d_3. Assignments based on the structure in Figure S3.2.1. The red dotted line in the inset shows the zoomed-in range of the full spectrum.

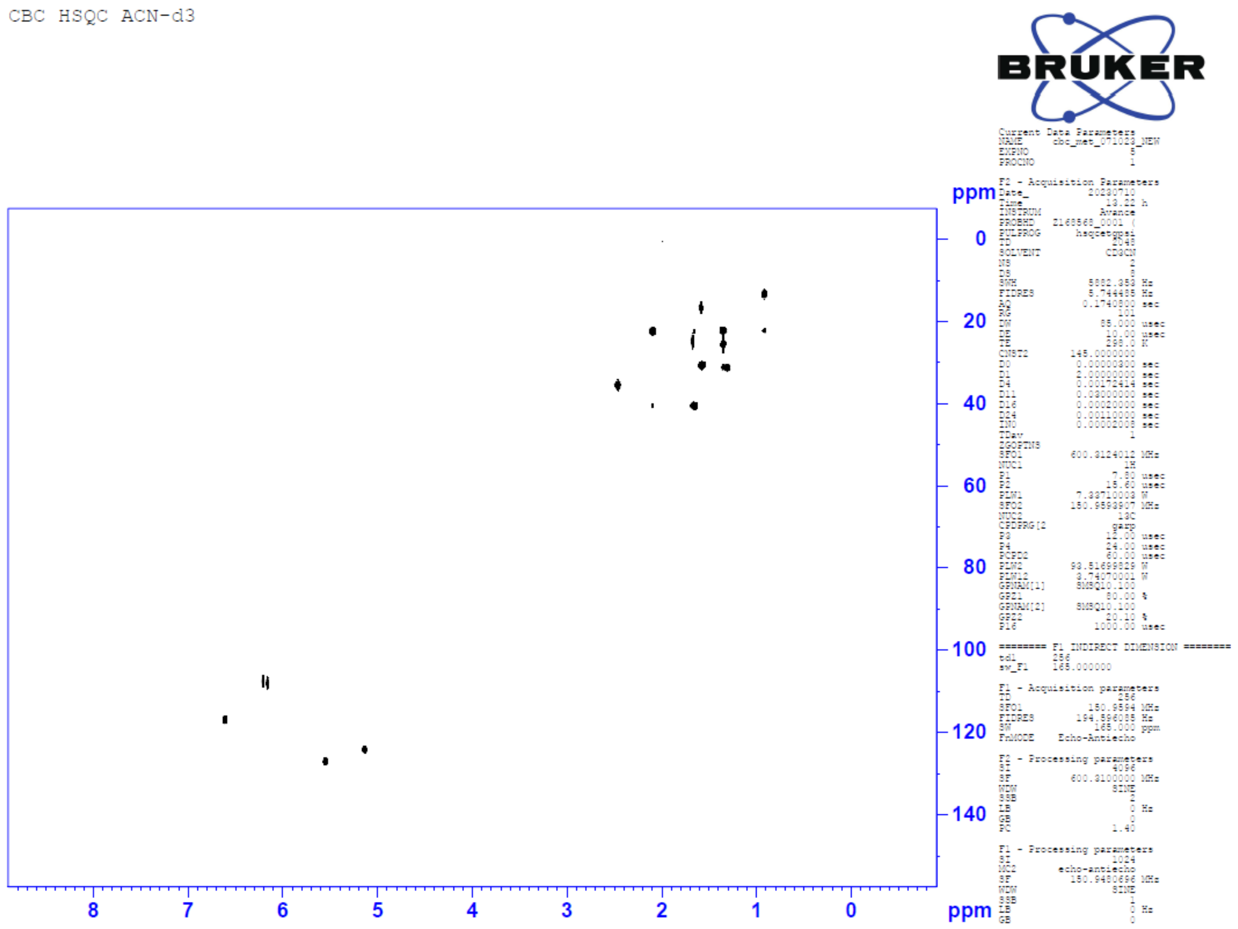

Figure S3.2.16. HSQC NMR spectrum of CBC in ACN-d_3.

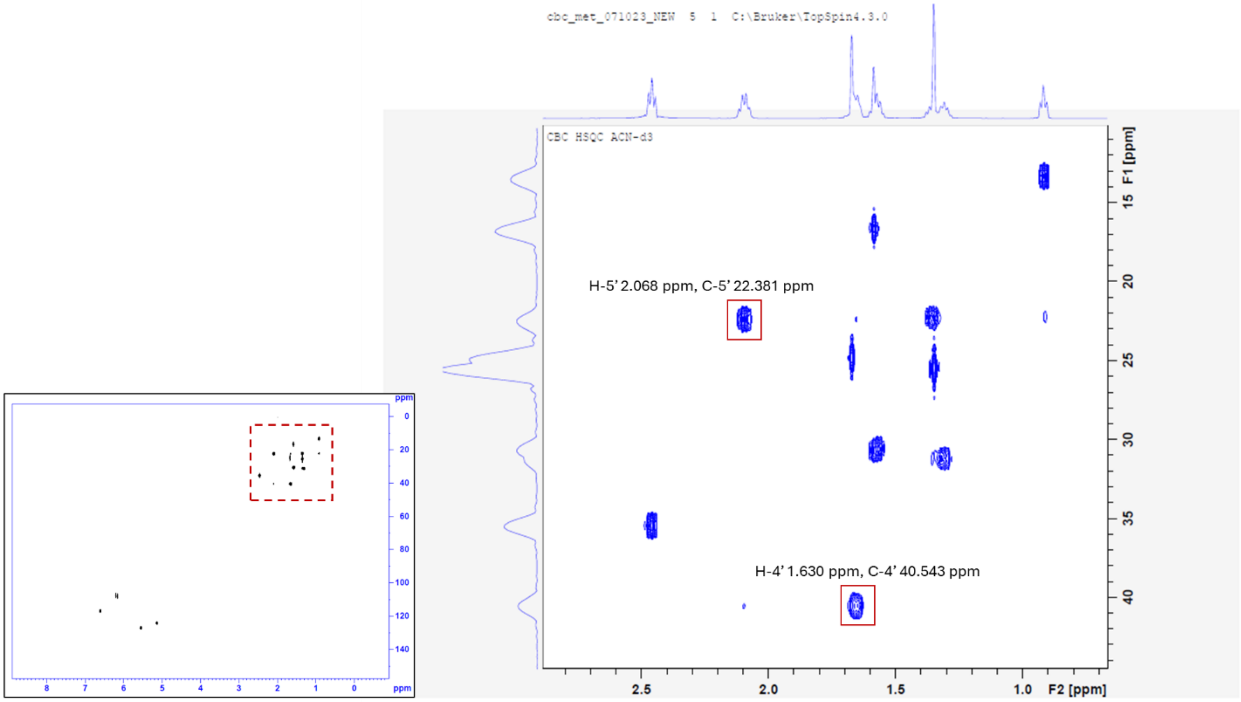

Figure S3.2.17. Zoomed-in HSQC NMR spectrum of CBC in ACN-d_3. Assignments based on the structure in Figure S3.2.1. The red dotted line in the inset shows the zoomed-in range of the full spectrum.

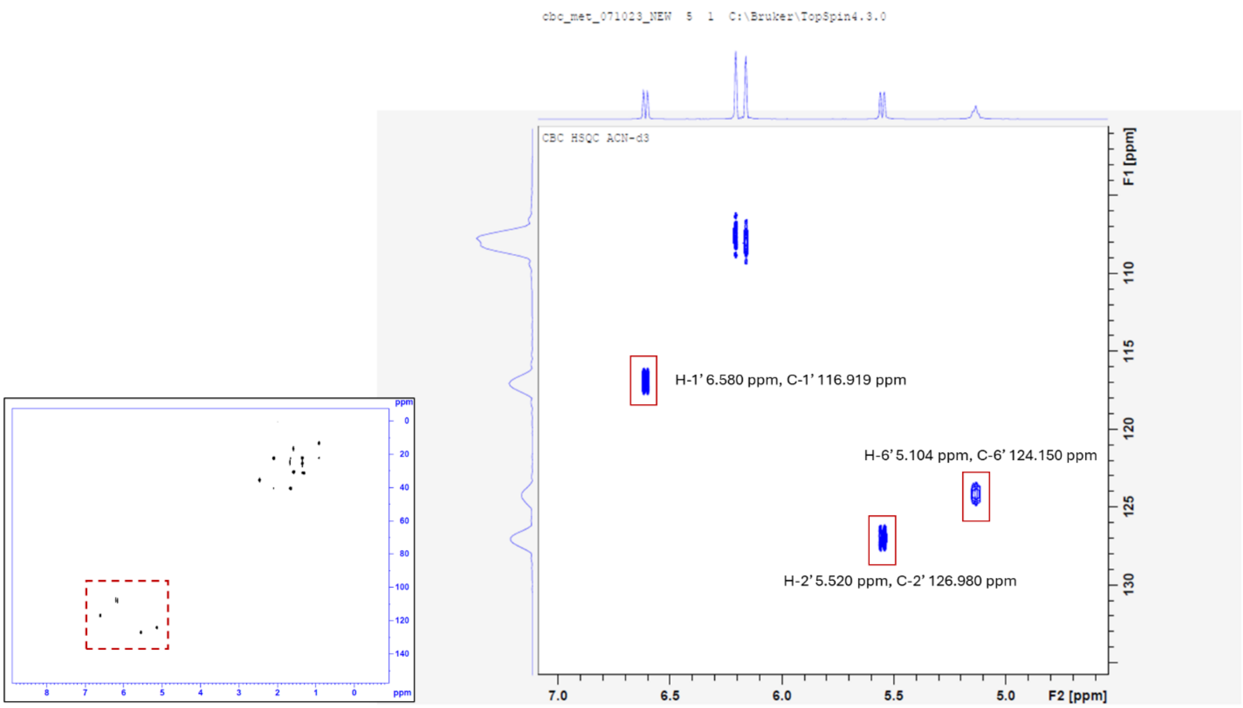

Figure S3.2.18. Zoomed-in HSQC NMR spectrum of CBC in ACN-d_3. Assignments based on the structure in Figure S3.2.1. The red dotted line in the inset shows the zoomed-in range of the full spectrum.

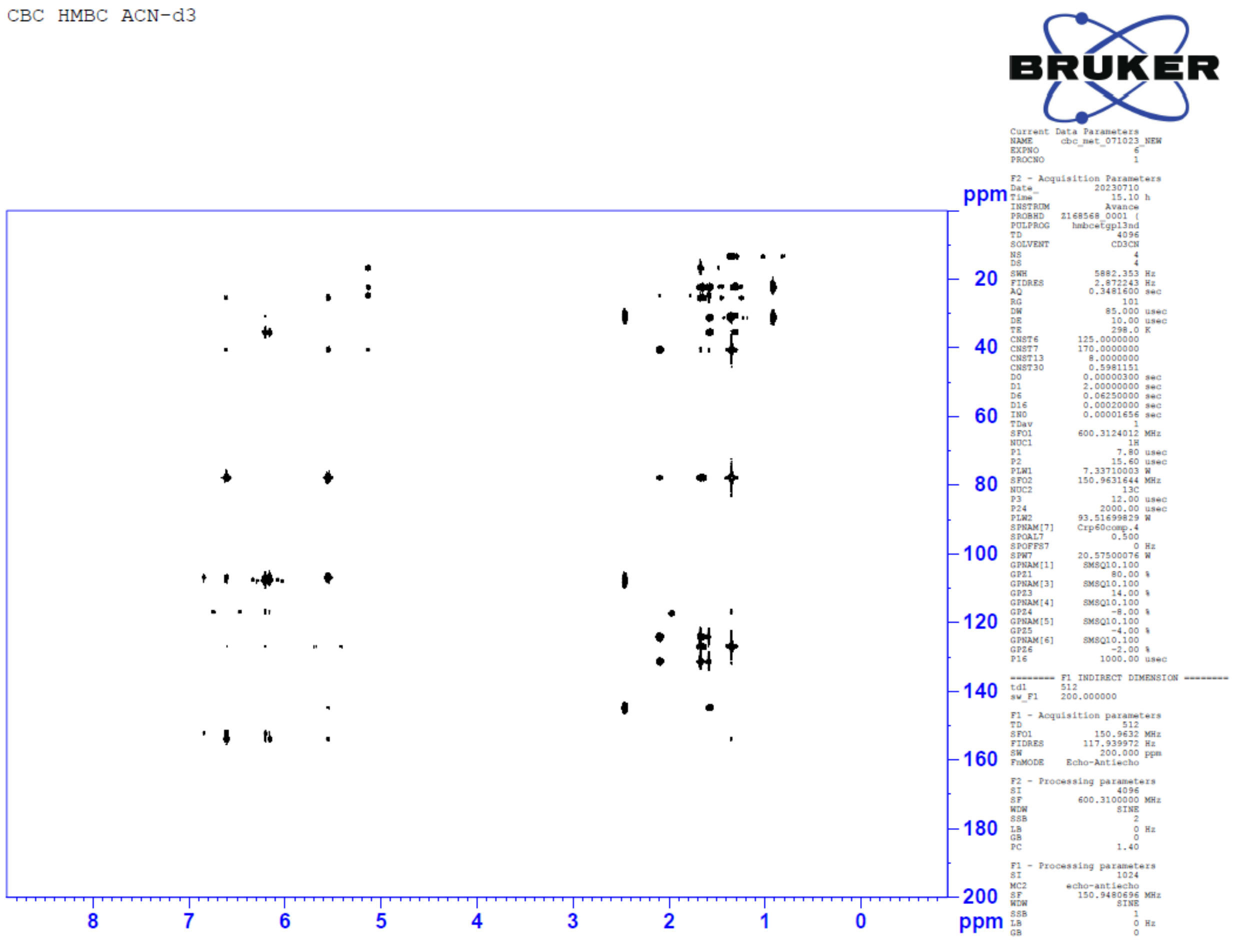

Figure S3.2.19. HMBC NMR spectrum of CBC in ACN-d$_3$.

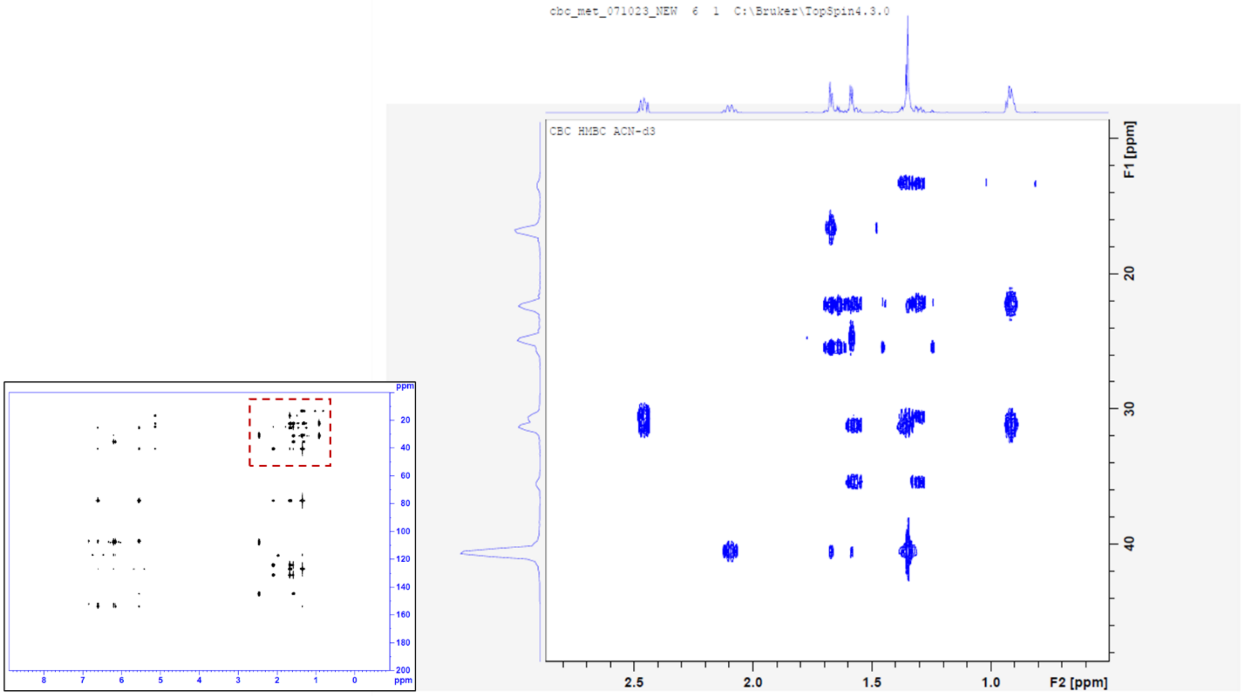

Figure S3.2.20. Zoomed-in HMBC NMR spectrum of CBC in ACN-d_3. The red dotted line in the inset shows the zoomed-in range of the full spectrum.

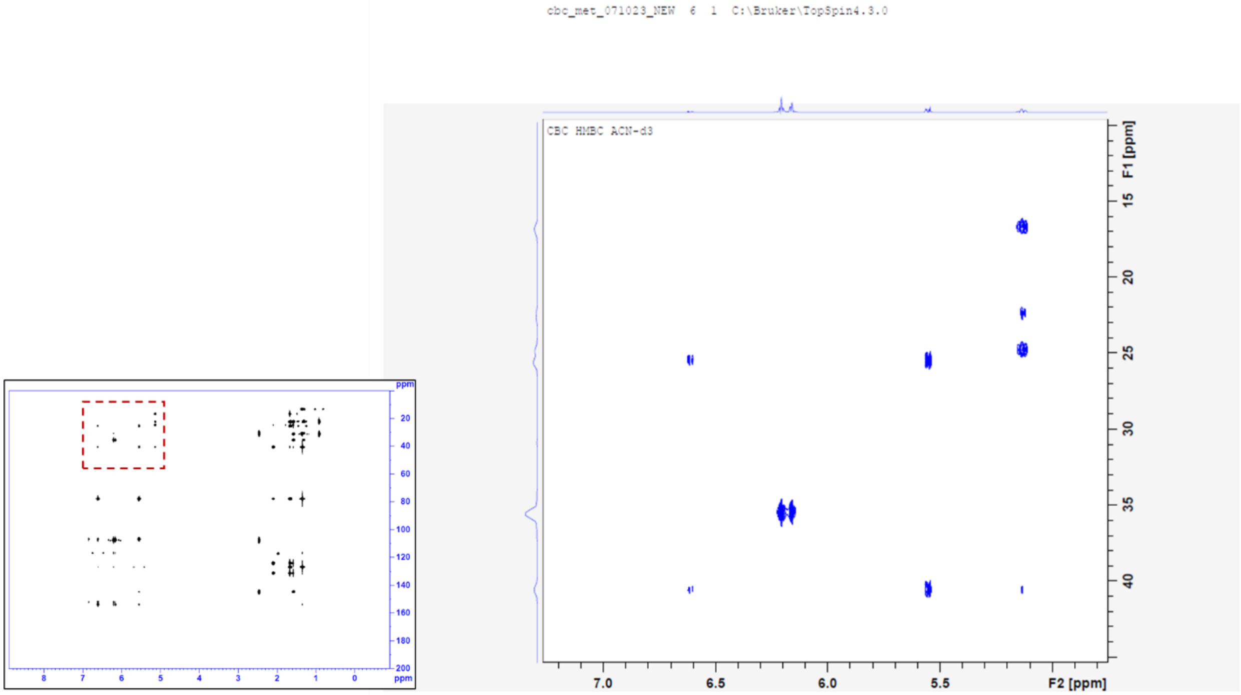

Figure S3.2.21. Zoomed-in HMBC NMR spectrum of CBC in ACN-d_3. The red dotted line in the inset shows the zoomed-in range of the full spectrum.

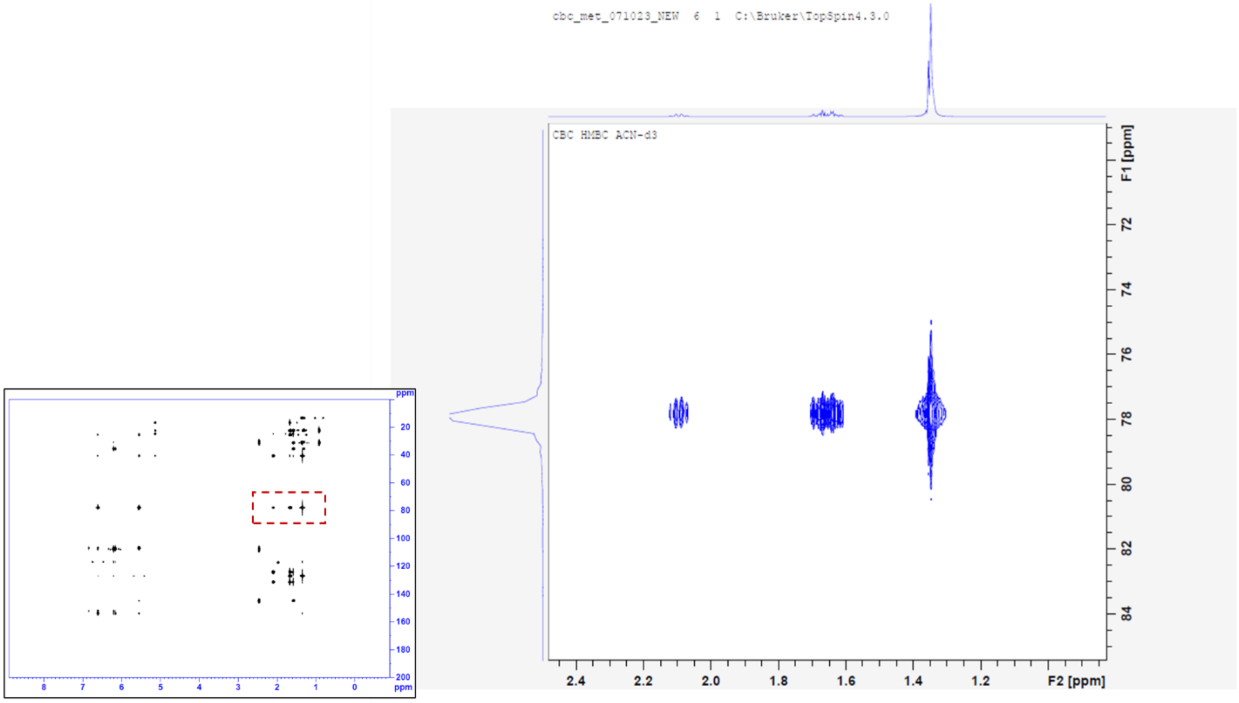

Figure S3.2.22. Zoomed-in HMBC NMR spectrum of CBC in ACN-d_3. The red dotted line in the inset shows the zoomed-in range of the full spectrum.

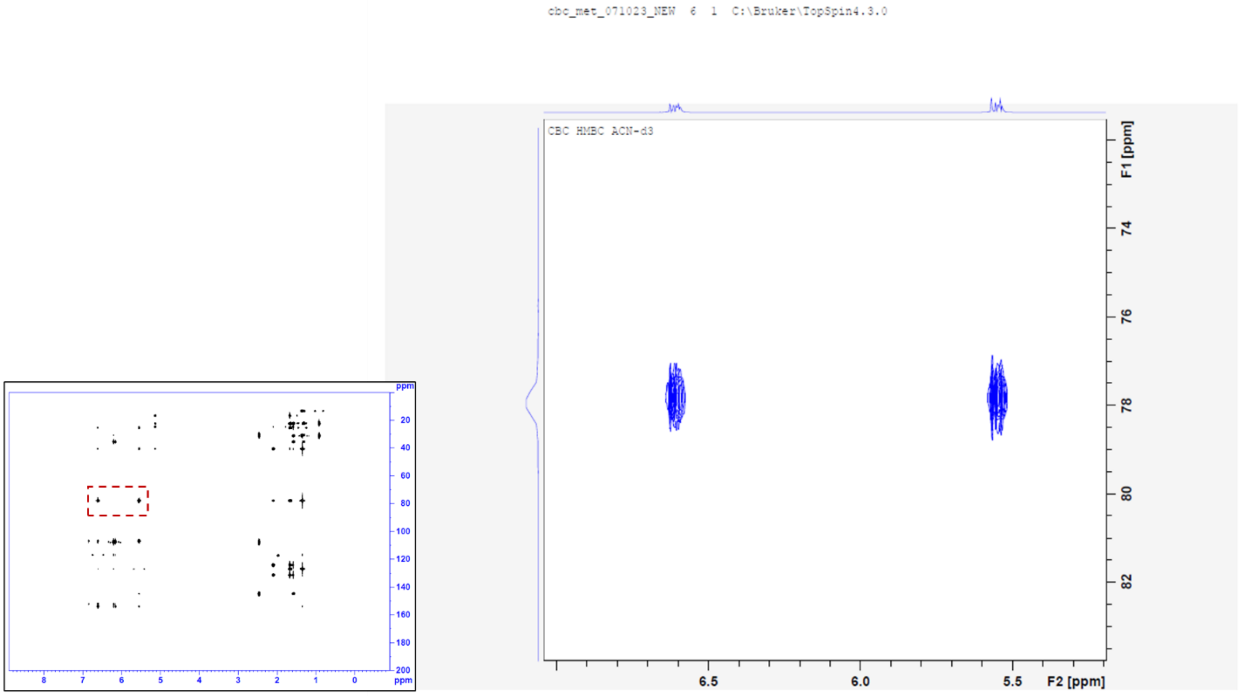

Figure S3.2.23. Zoomed-in HMBC NMR spectrum of CBC in ACN-d_3. The red dotted line in the inset shows the zoomed-in range of the full spectrum.

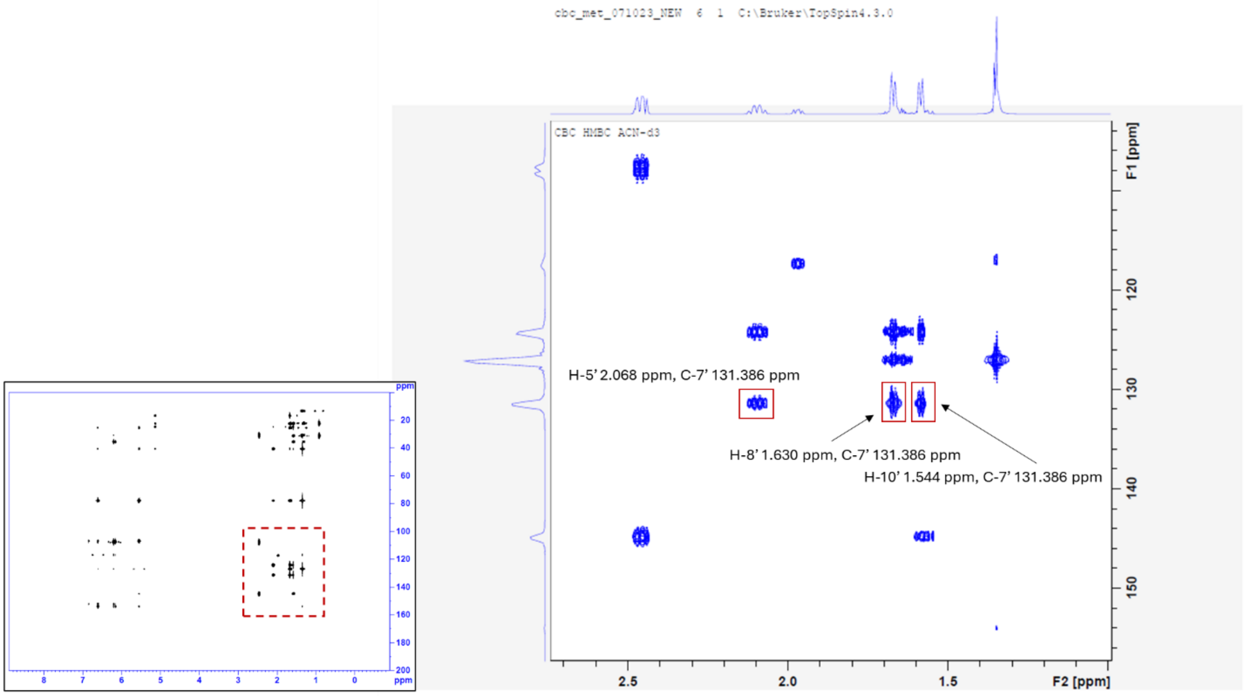

Figure S3.2.24. Zoomed-in HMBC NMR spectrum of CBC in ACN-d$_3$. Assignments based on the structure in Figure S3.2.1. The red dotted line in the inset shows the zoomed-in range of the full spectrum.

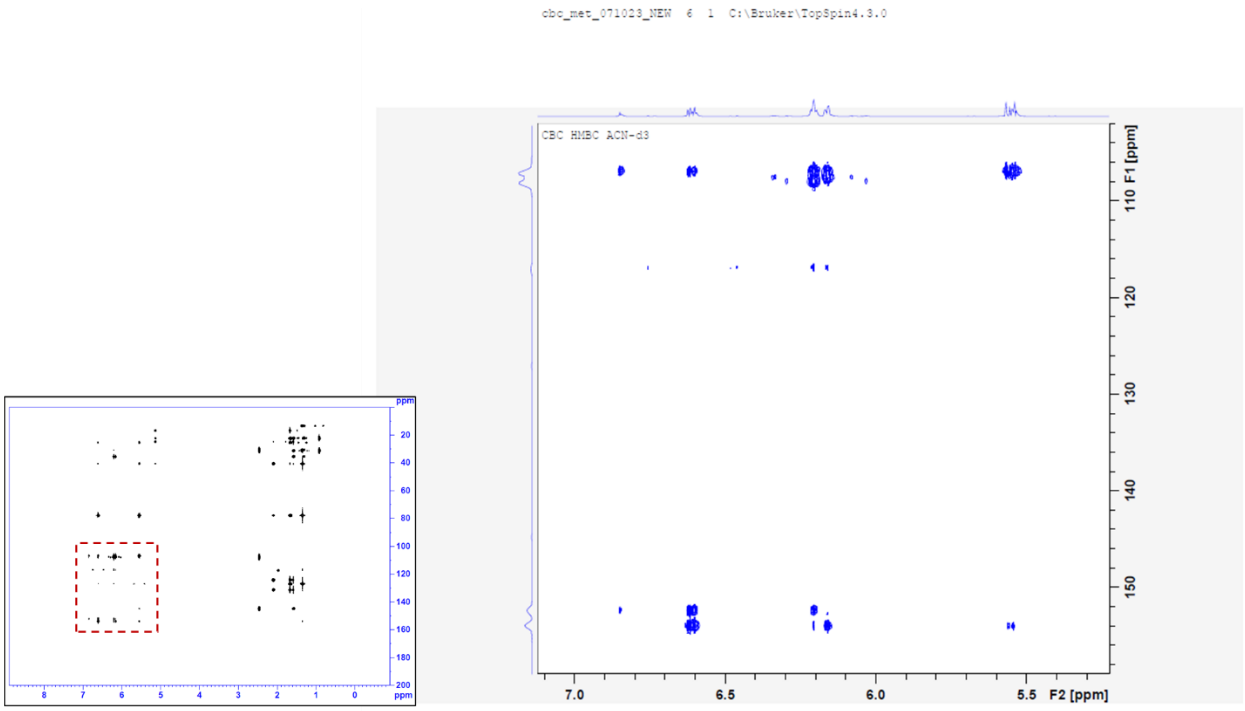

Figure S3.2.25. Zoomed-in HMBC NMR spectrum of CBC in ACN-d$_3$. The red dotted line in the inset shows the zoomed-in range of the full spectrum.

S3.3

2'-hydroxycannabicitran NMR Experiments

2'-hydroxycannabicitran was comprehensively examined in ACN-d_3. Figures include NMR parameters, full spectra, and zoomed-in spectra with highlighted signals.

Please see the supplementary figures described below:

Chemical structure and numbering assignment of 2'-hydroxycannabicitran: Figure S.3.3.1

^{1}H in ACN-d_3: Figures S.3.3.2- S3.3.5

^{13}C in ACN-d_3: Figures S.3.3.6- S3.3.10

DEPT-135 in ACN-d_3: Figures S.3.3.11- S3.3.16

COSY in ACN-d_3: Figures S.3.3.17- S3.3.19

HSQC in ACN-d_3: Figures S.3.3.20- S3.3.23

HMBC in ACN-d_3: Figures S.3.3.24- S3.3.28

NOESY in ACN-d_3: Figures S.3.3.29 and S3.3.30

Methods

^{1}H one dimensional NMR experiments were collected over a spectral width of 16 ppm using a total of 64K complex data points (R+I) giving an acquisition time of 3.14 s per fid with a 30 s relaxation delay between transients to ensure complete relaxation for accurate integration. There were 8-16 transients per experiment.

^{13}C one dimensional experiments were collected using 1024 transients over a spectral width of 216 ppm with 64K complex data points (R+I) giving an acquisition time of 1.002 s per fid with a relaxation time of 2 s between transients. ^{1}H decoupling was achieved using waltz16 with a B1 field strength of 3.6 KHz centered at 4.00 ppm ^{1}H.

^{13}C Distortionless Enhancement by Polarization Transfer (DEPT-135) spectra were acquired over a C13 spectral width of 180 ppm using a total of 32K data points (R+I) giving an acquisition time of 0.61 s per fid with a relaxation time of 2 s between transients. ^{1}H decoupling was achieved using waltz16 with a B1 field strength of 3.6 KHz centered at 4.00 ppm ^{1}H.

^{1}H-^{13}C Heteronuclear Single Quantum Coherence (HSQC) spectra were acquired using 2048 data points (R+I) over a spectral width of 9.8 ppm in the ^{1}H dimension and 256 data points (R+I) over 165 ppm in the C13 dimension. The transmitter offsets were centered at 4.0 ppm and 75 ppm respectively. 16 transients were acquired per FID using a 2 s relaxation delay. C13 decoupling during acquisition was achieved using GARP with a ^{13}C B1 field strength of 4.2 KHz centered at 75 ppm.

For ^{1}H-^{13}C Heteronuclear Multiple Bond Correlation (HMBC) experiments, the spectral widths were

9.8 and 200 pm respectively in the ^{1}H and ^{13}C dimensions and used a total of 4096 by 512 complex data points. The transmitter offsets were set at 4.0 ppm in ^{1}H and 100 ppm in the ^{13}C dimensions. 32 transients were collected per FID using a 2 s relaxation delay between transients.

^{1}H-^{1}H gradient selected Double Quantum Filtered-Homonuclear Correlation Spectroscopy (DQF-COSY) and Nuclear Overhauser Effect Spectroscopy (NOESY) experiments were collected with spectral widths of 10 ppm in both dimensions, with the transmitter centered at 4.0 ppm using 4K data points in the acquired dimension and 1K data points in the indirect dimension. For NOESY the mixing time was set at 300 ms to minimize spin diffusion.

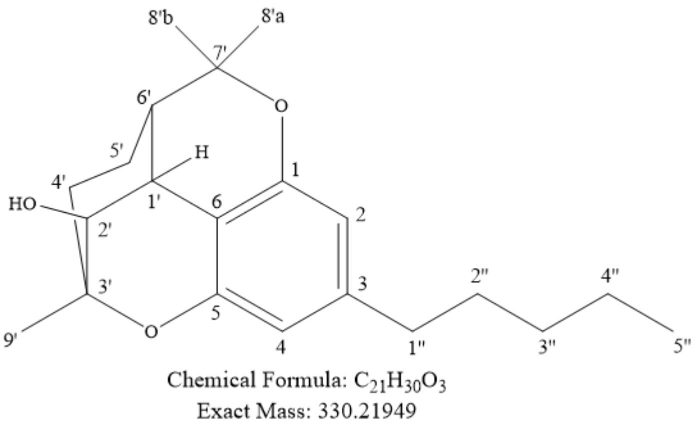

Figure S3.3.1. Numbered structure of 2'-hydroxycannabicitran according to a terpenoid system of numbering [36, 37].

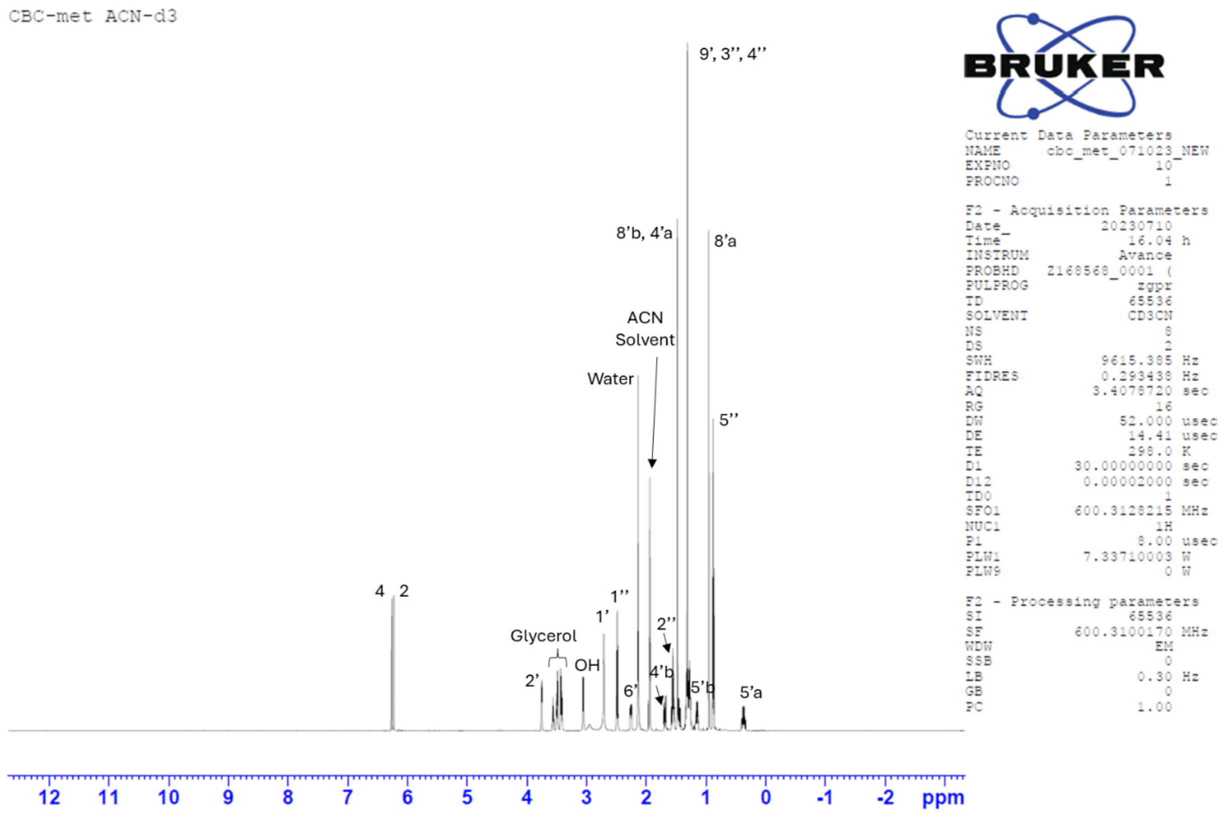

Figure S3.3.2. ^{1}H NMR spectrum of 2'-hydroxycannabicitran in ACN-d_3. Assignments based on the structure in Figure S3.3.1.

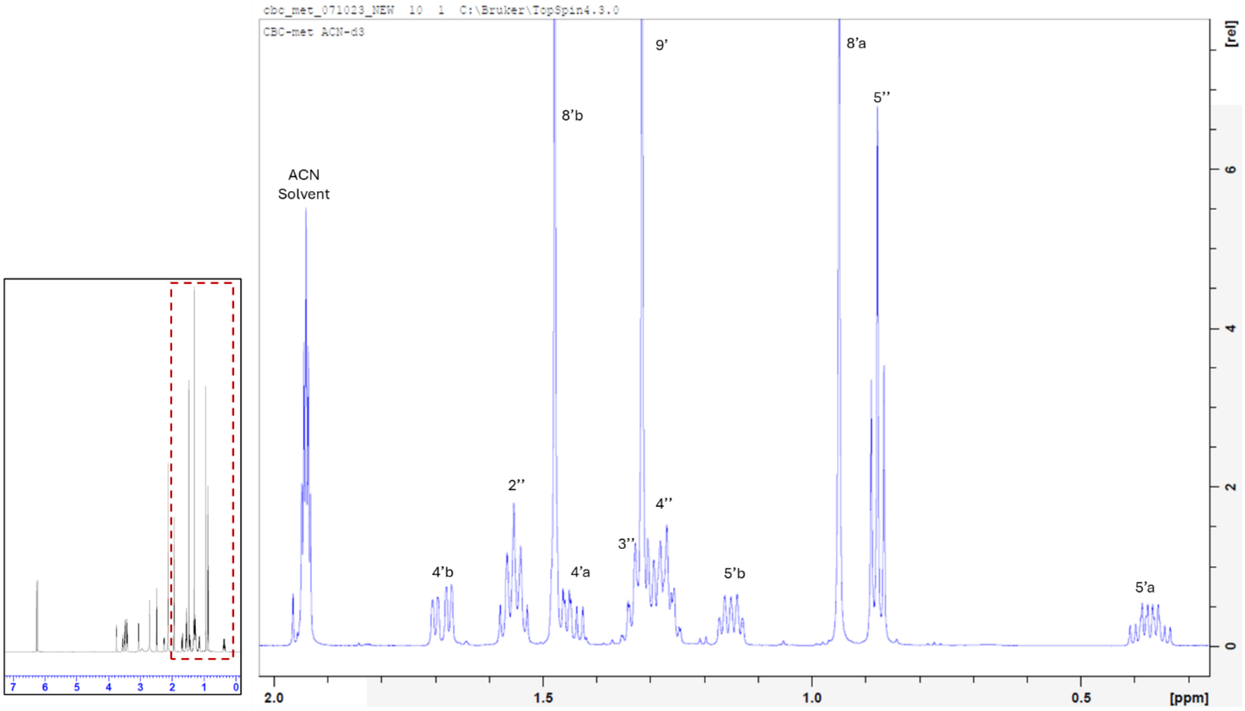

Figure S3.3.3. Zoomed-in ^{1}H NMR spectrum of 2'-hydroxycannabicitran in ACN-d_3. Assignments based on the structure in Figure S3.3.1. The red dotted line in the inset shows the zoomed-in range of the full spectrum.

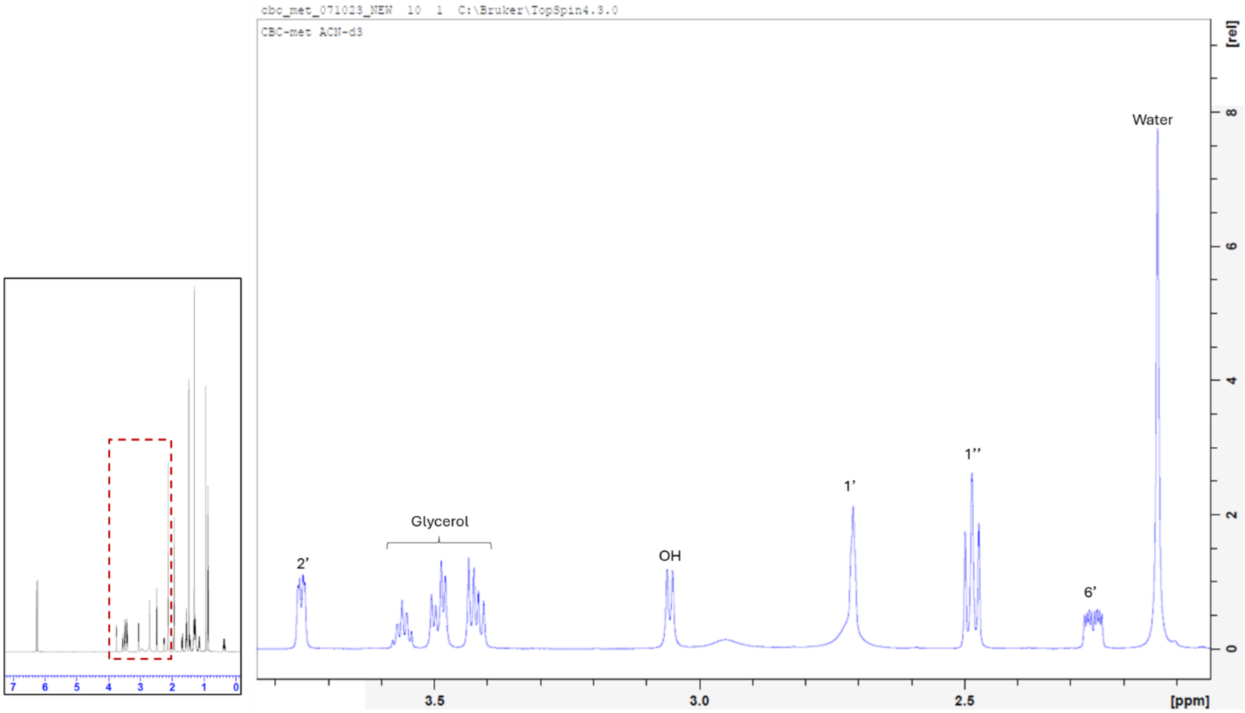

Figure S3.3.4. Zoomed-in ^{1}H NMR spectrum of 2'-hydroxycannabicitran in ACN-d_3. Assignments based on the structure in Figure S3.3.1. The red dotted line in the inset shows the zoomed-in range of the full spectrum.

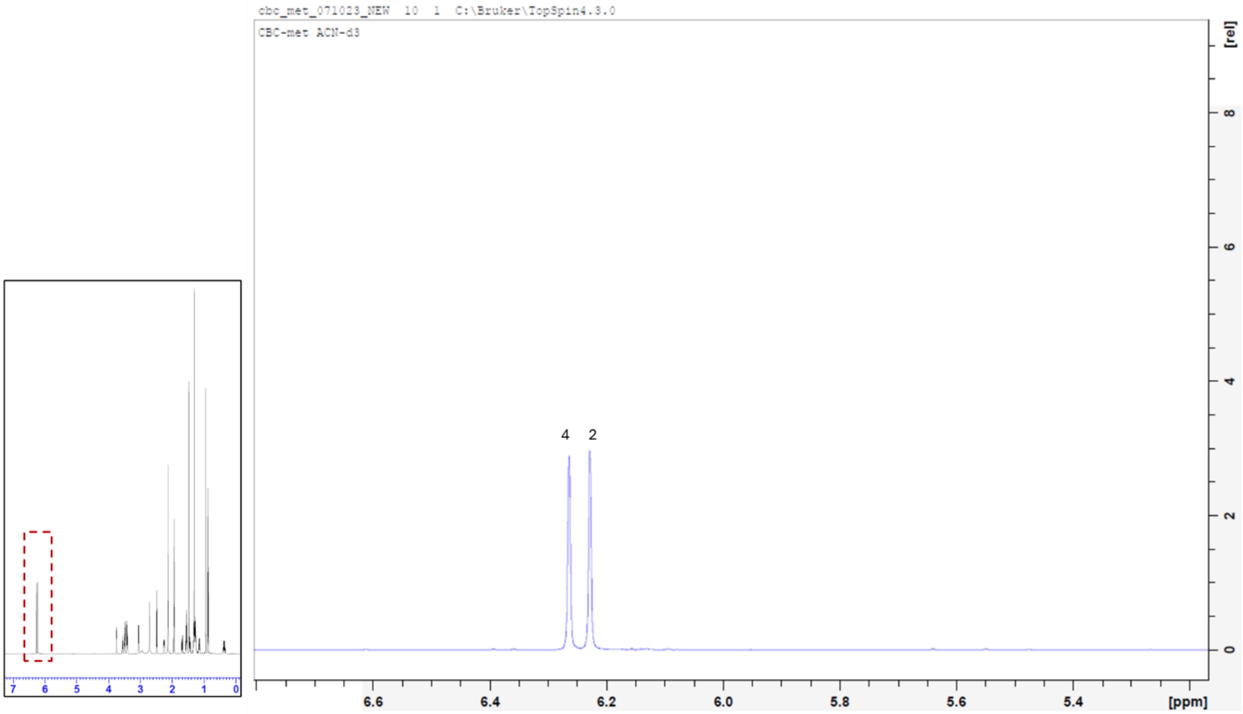

Figure S3.3.5. Zoomed-in ^{1}H NMR spectrum of 2'-hydroxycannabicitran in ACN-d_3. Assignments based on the structure in Figure S3.3.1. The red dotted line in the inset shows the zoomed-in range of the full spectrum.

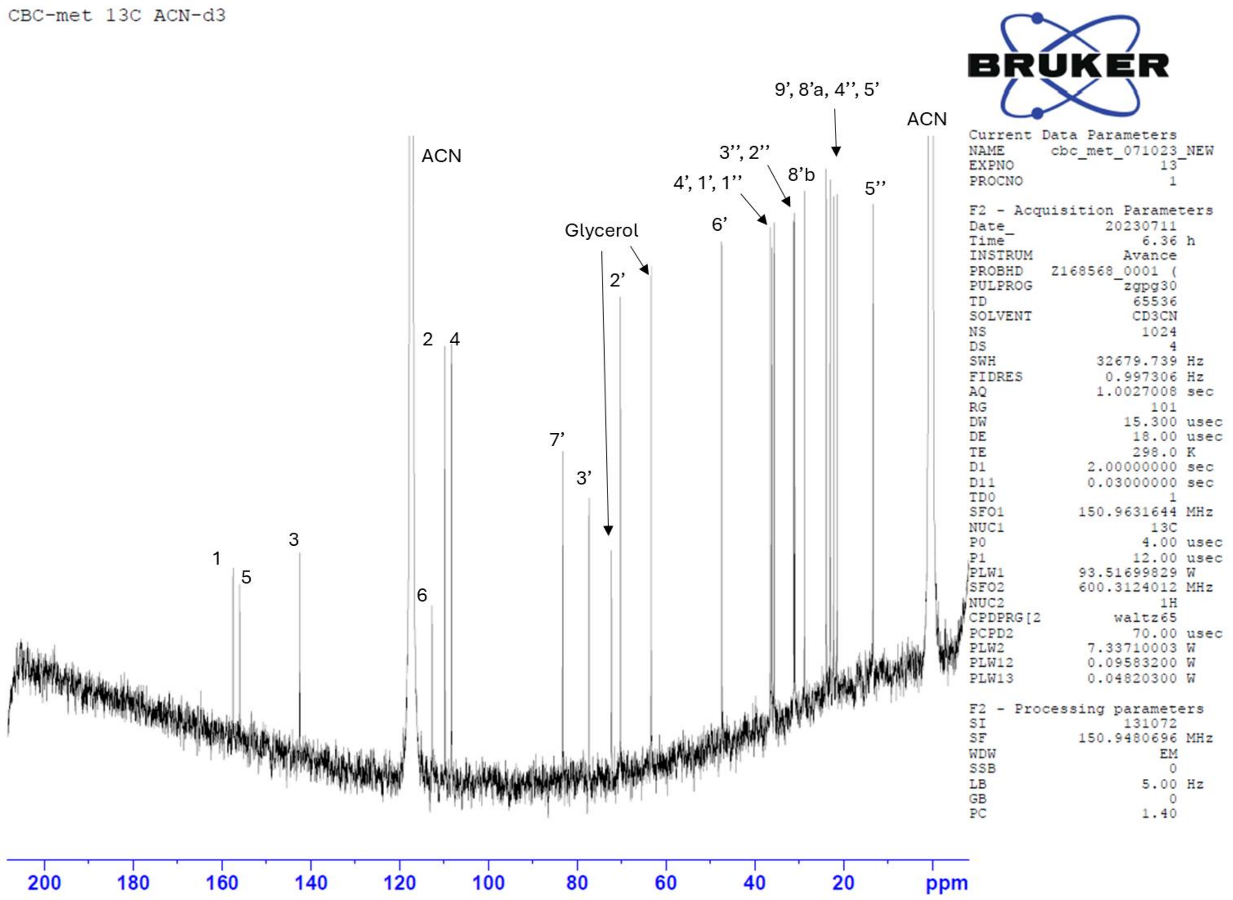

Figure S3.3.6. ^{13}C NMR spectrum of 2’-hydroxycannabicitran in ACN-d_3. Assignments based on the structure in Figure S3.3.1.

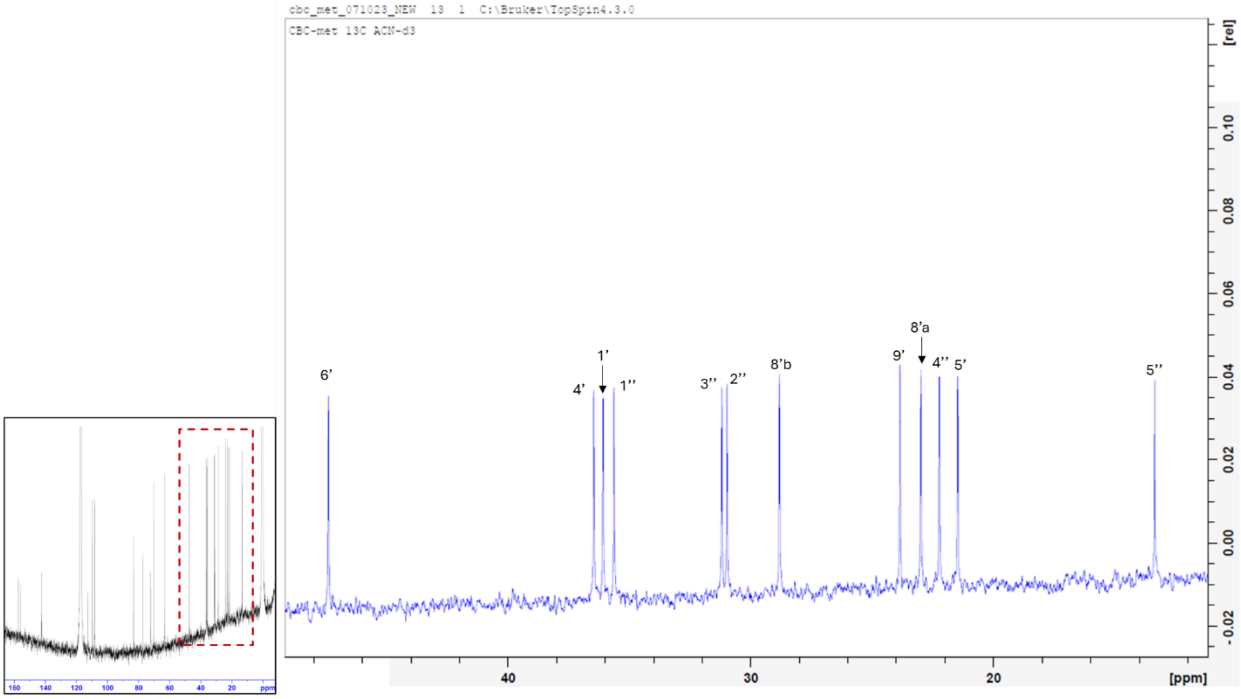

Figure S3.3.7. Zoomed-in ^{13}C NMR spectrum of 2'-hydroxycannabicitran in ACN-d_3. Assignments based on the structure in Figure S3.3.1. The red dotted line in the inset shows the zoomed-in range of the full spectrum.

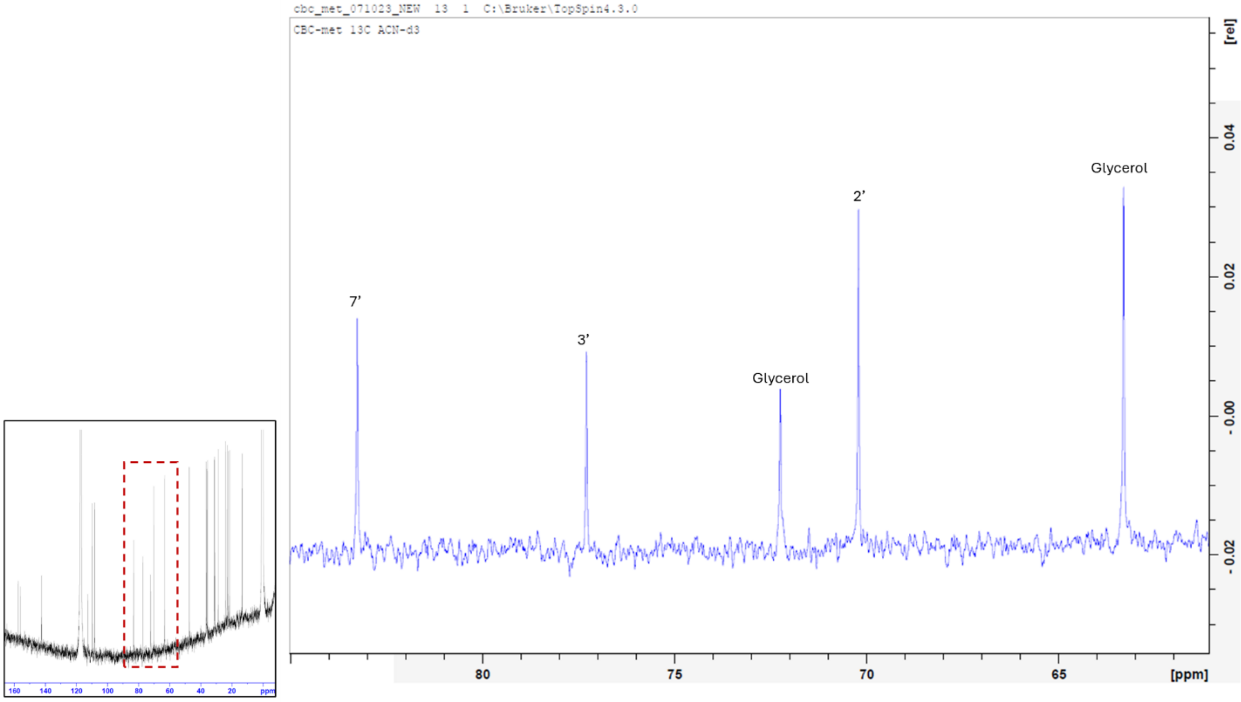

Figure S3.3.8. Zoomed-in ^{13}C NMR spectrum of 2'-hydroxycannabicitran in ACN-d_3. Assignments based on the structure in Figure S3.3.1. The red dotted line in the inset shows the zoomed-in range of the full spectrum.

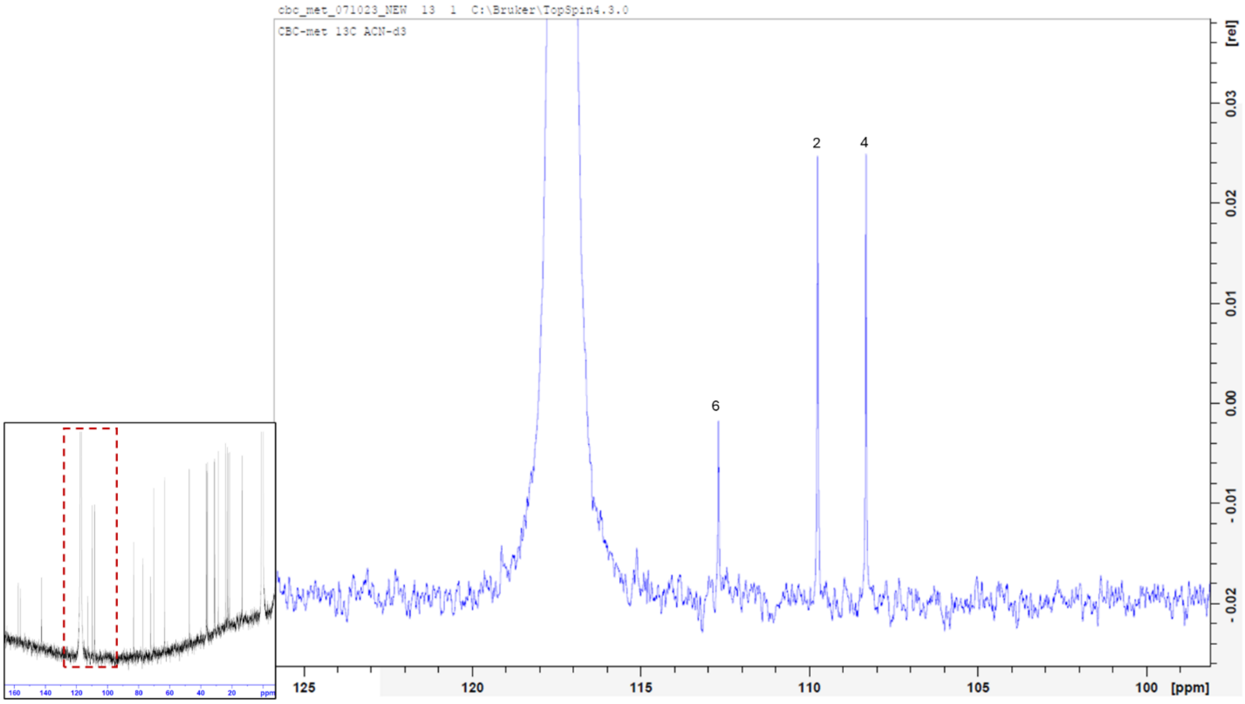

Figure S3.3.9. Zoomed-in ^{13}C NMR spectrum of 2’-hydroxycannabicitran in ACN-d_3. Assignments based on the structure in Figure S3.3.1. The red dotted line in the inset shows the zoomed-in range of the full spectrum.

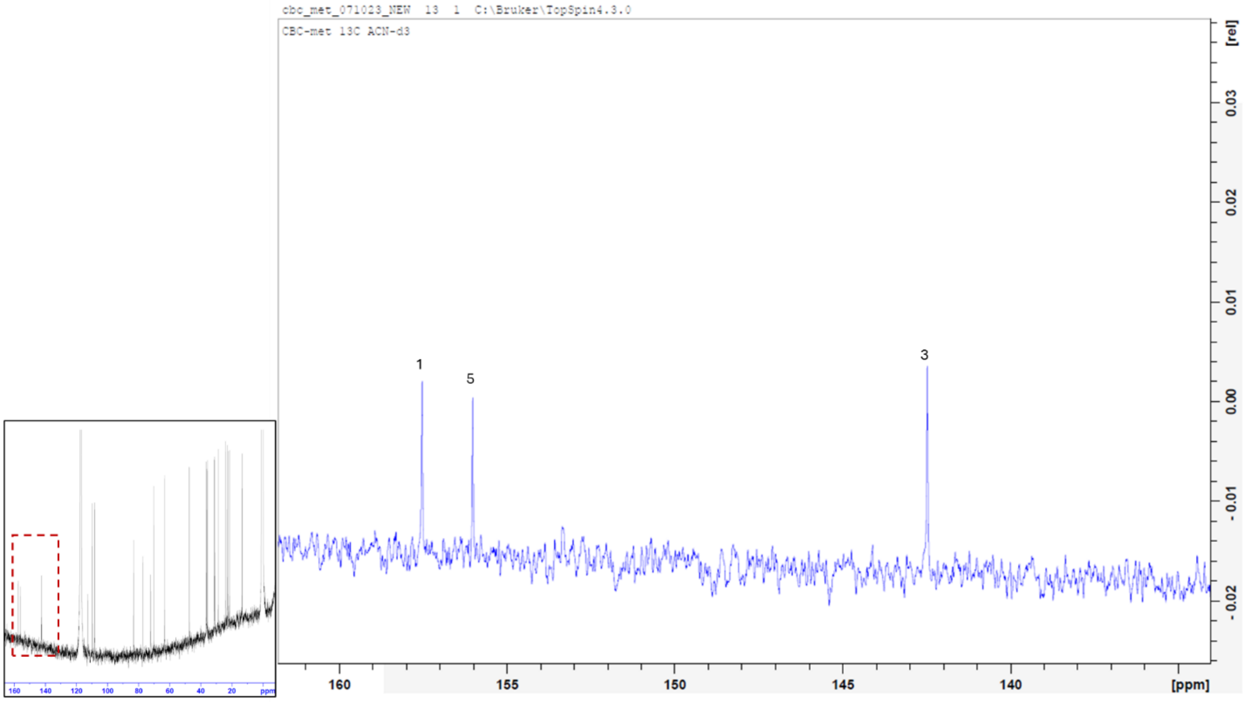

Figure S3.3.10. Zoomed-in ^{13}C NMR spectrum of 2’-hydroxycannabicitran in ACN-d_3. Assignments based on the structure in Figure S3.3.1. The red dotted line in the inset shows the zoomed-in range of the full spectrum.

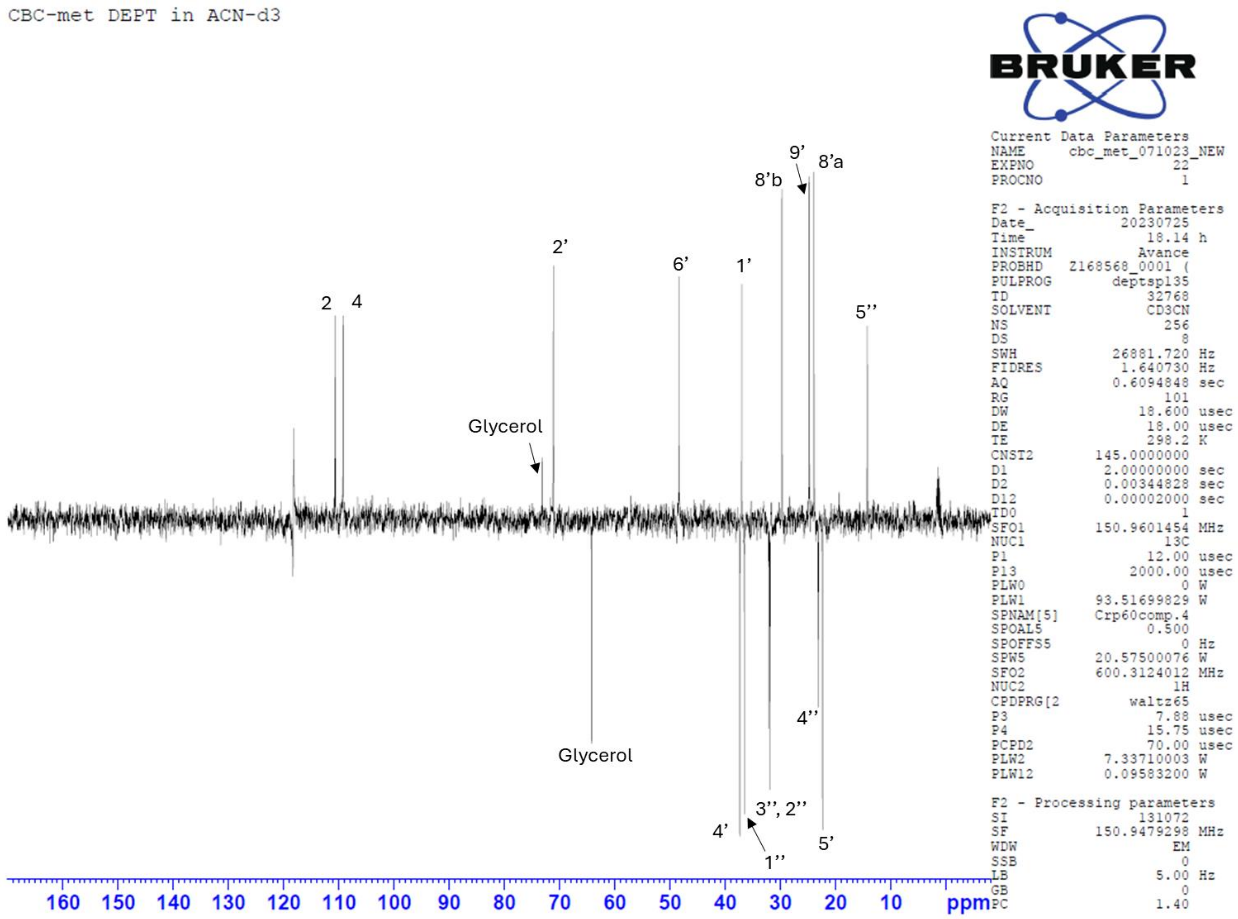

Figure S3.3.11. DEPT-135 NMR spectrum of 2'-hydroxycannabicitran in ACN-d$_3$. Assignments based on the structure in Figure S3.3.1.

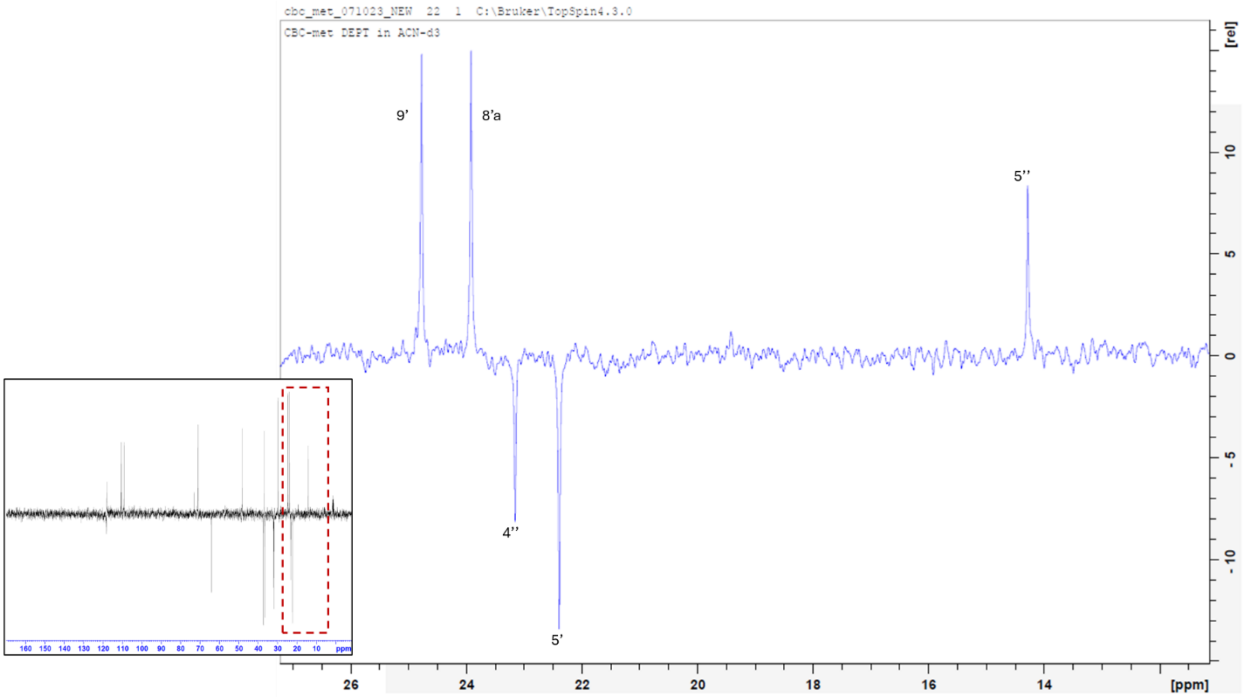

Figure S3.3.12. Zoomed-in DEPT-135 NMR spectrum of 2'-hydroxycannabicitran in ACN-d_3. Assignments based on the structure in Figure S3.3.1. The red dotted line in the inset shows the zoomed-in range of the full spectrum.

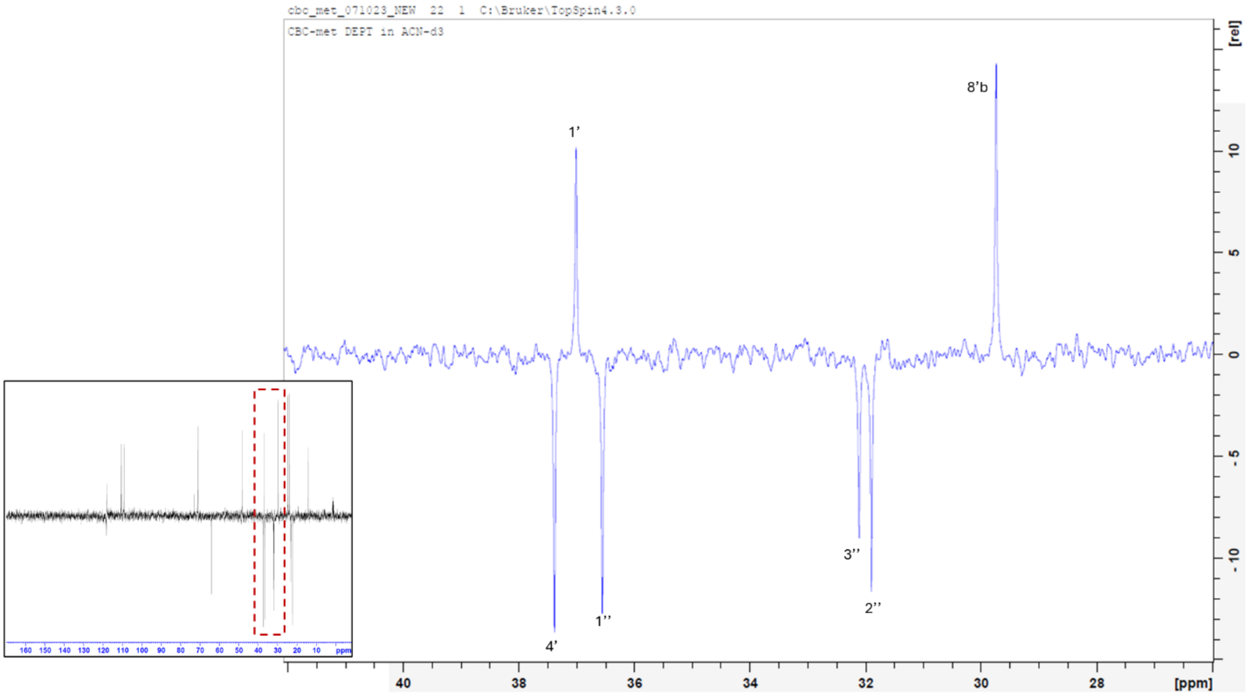

Figure S3.3.13. Zoomed-in DEPT-135 NMR spectrum of 2'-hydroxycannabicitran in ACN-d_3. Assignments based on the structure in Figure S3.3.1. The red dotted line in the inset shows the zoomed-in range of the full spectrum.

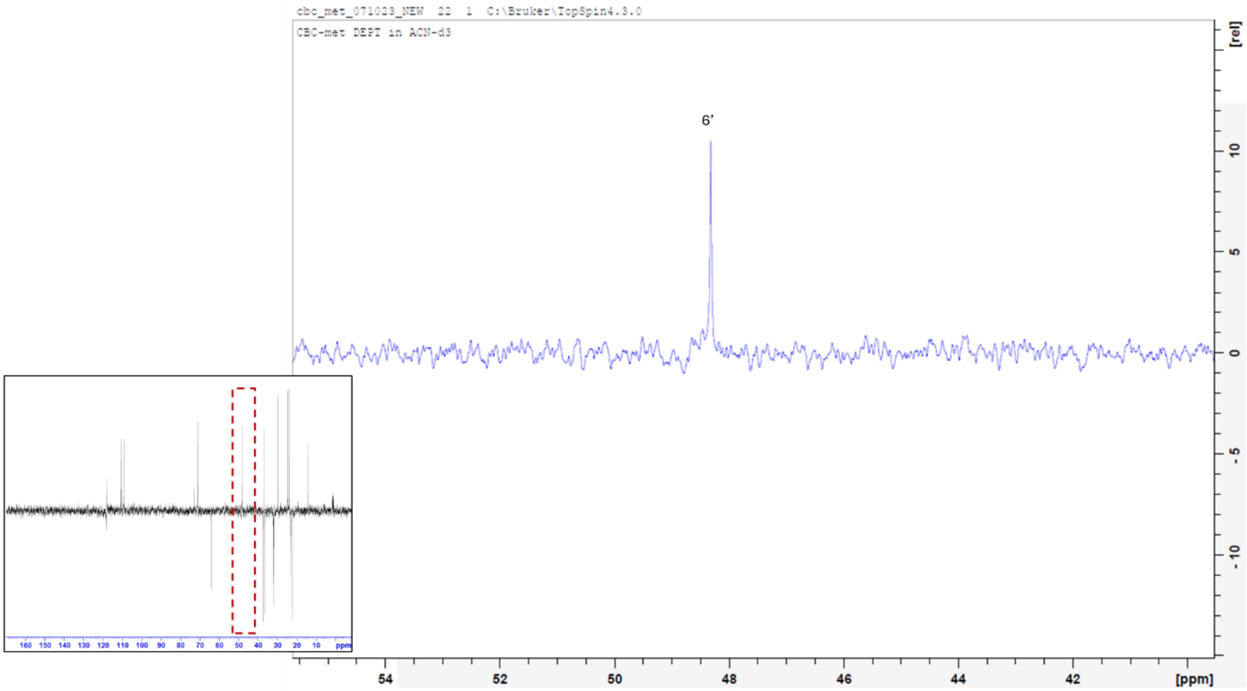

Figure S3.3.14. Zoomed-in DEPT-135 NMR spectrum of 2’-hydroxycannabicitran in ACN-d_3. Assignments based on the structure in Figure S3.3.1. The red dotted line in the inset shows the zoomed-in range of the full spectrum.

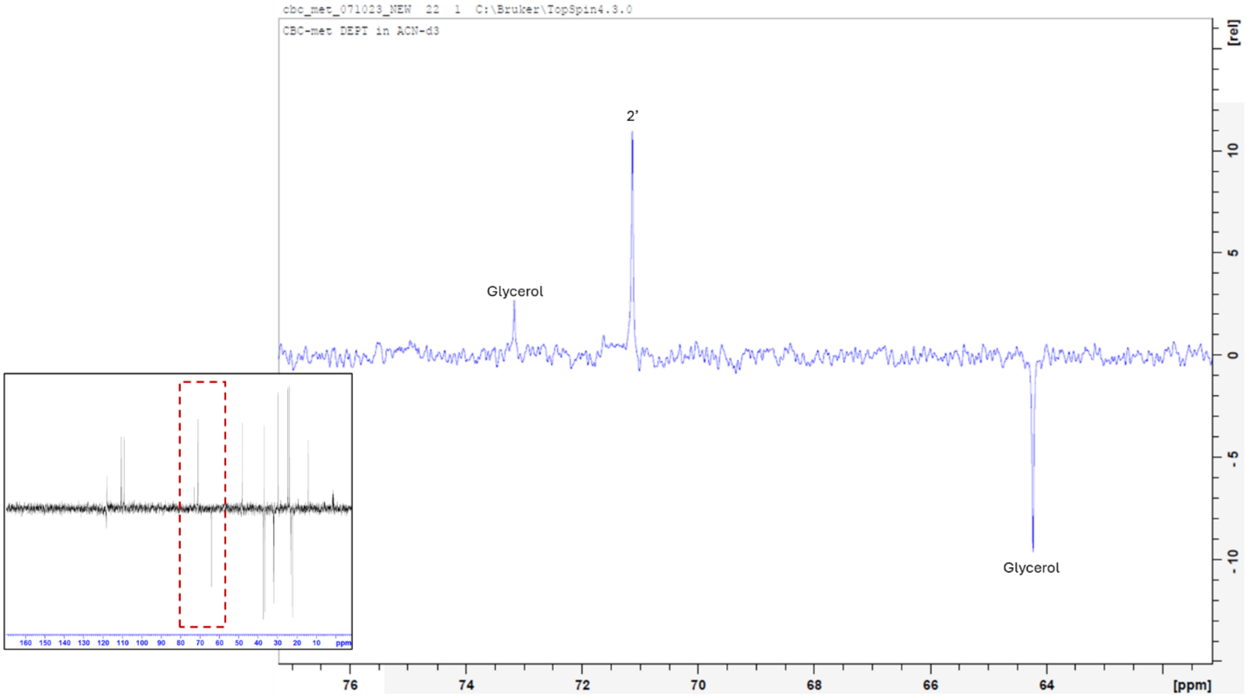

Figure S3.3.15. Zoomed-in DEPT-135 NMR spectrum of 2’-hydroxycannabicitran in ACN-d_3. Assignments based on the structure in Figure S3.3.1. The red dotted line in the inset shows the zoomed-in range of the full spectrum.

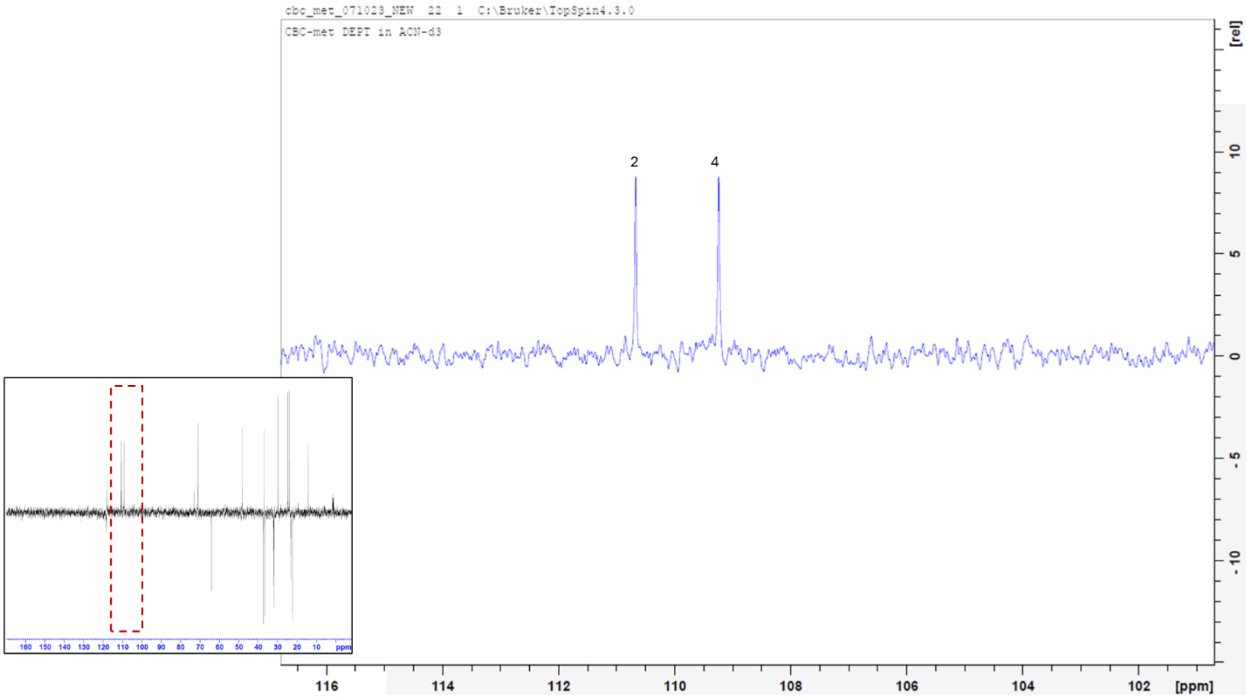

Figure S3.3.16. Zoomed-in DEPT-135 NMR spectrum of 2'-hydroxycannabicitran in ACN-d_3. Assignments based on the structure in Figure S3.3.1. The red dotted line in the inset shows the zoomed-in range of the full spectrum.

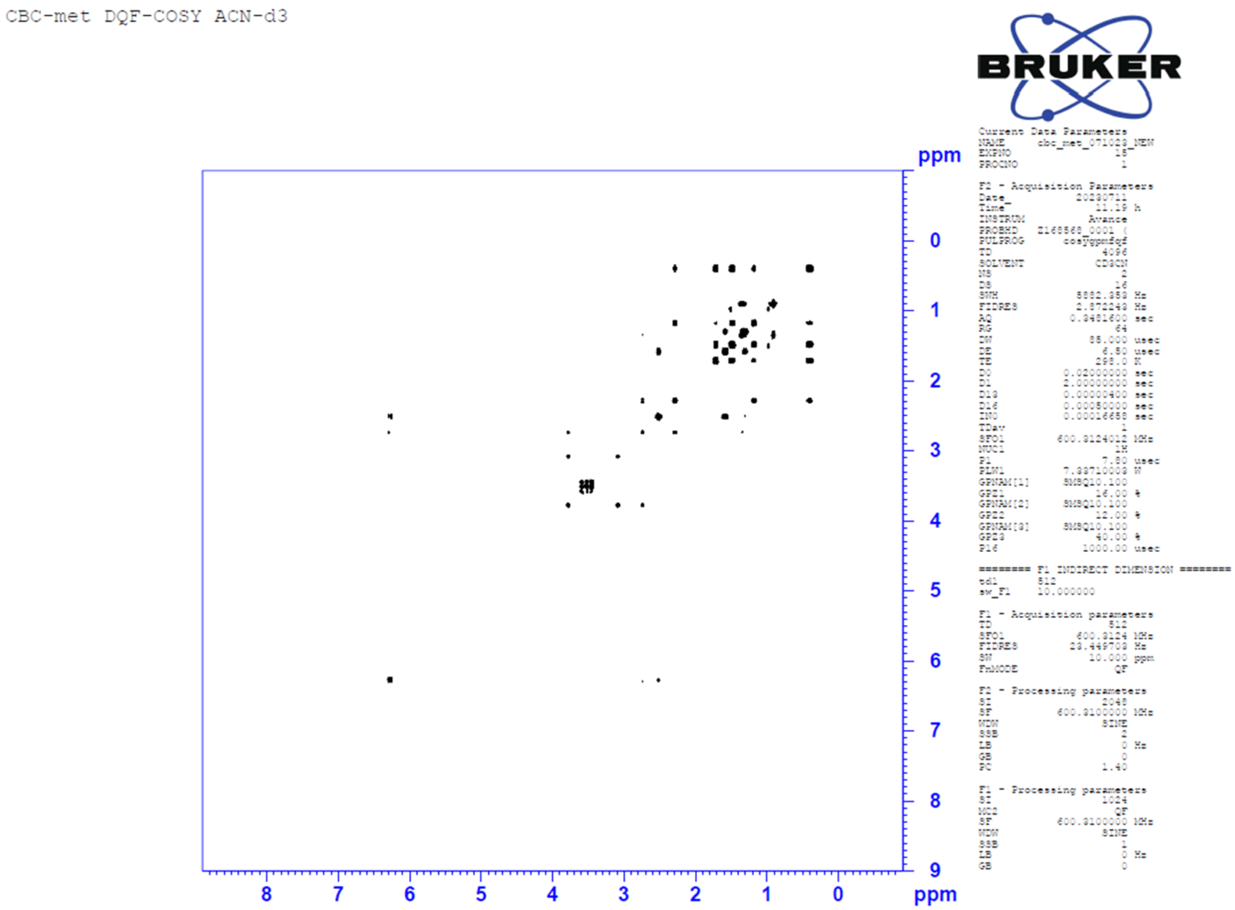

Figure S3.3.17. COSY NMR spectrum of 2’-hydroxycannabicitran in ACN-d_3.

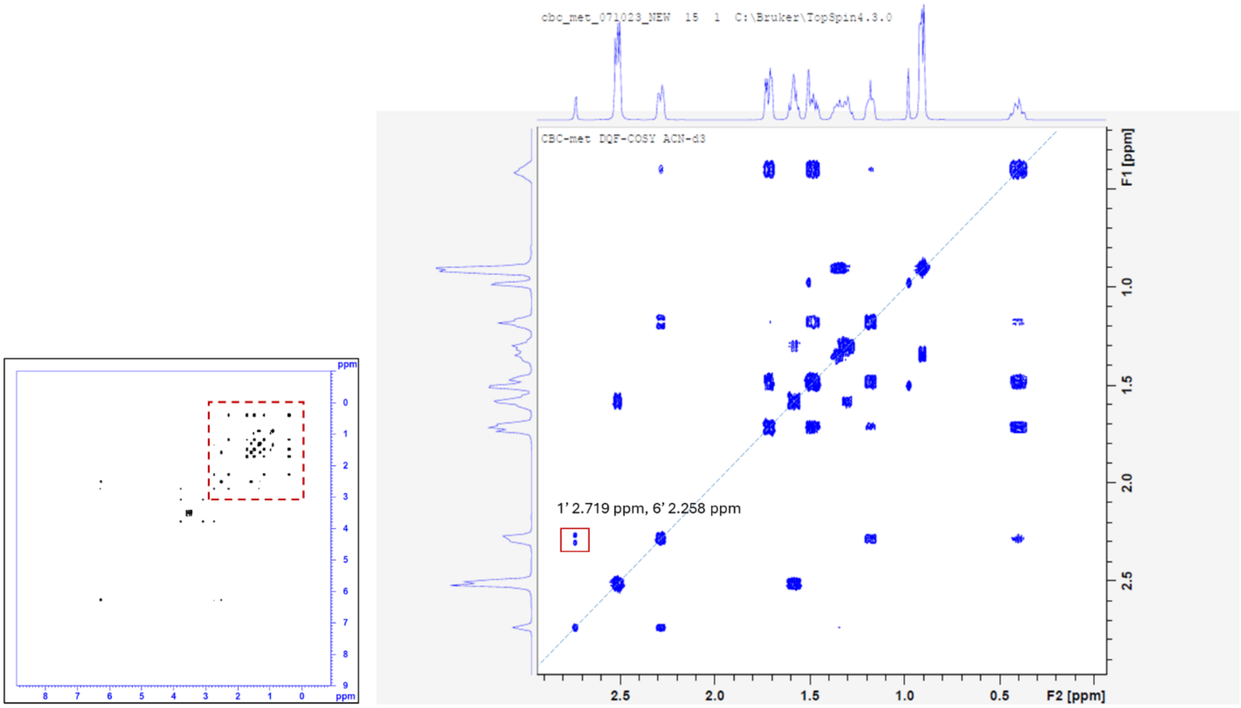

Figure S3.3.18. Zoomed-in COSY NMR spectrum of 2'-hydroxycannabicitran in ACN-d_3. Assignments based on the structure in Figure S3.3.1. The red dotted line in the inset shows the zoomed-in range of the full spectrum.

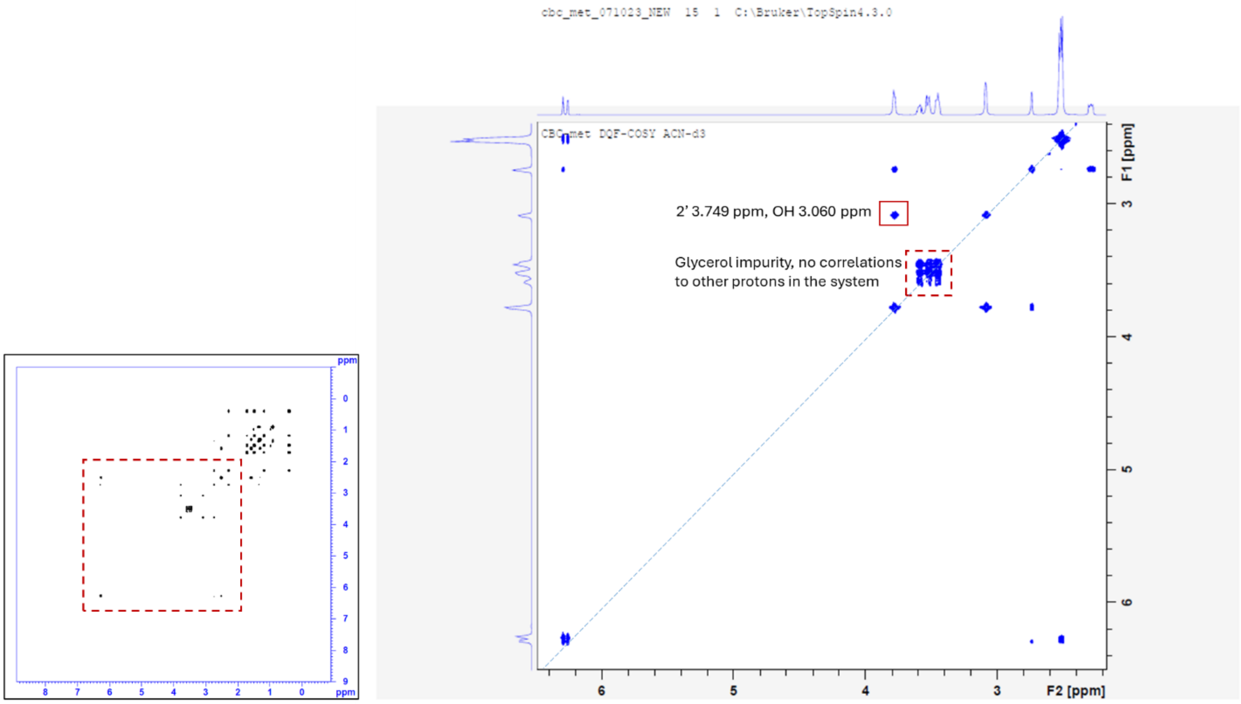

Figure S3.3.19. Zoomed-in COSY NMR spectrum of 2'-hydroxycannabicitran in ACN-d_3. Assignments based on the structure in Figure S3.3.1. The red dotted line in the inset shows the zoomed-in range of the full spectrum.

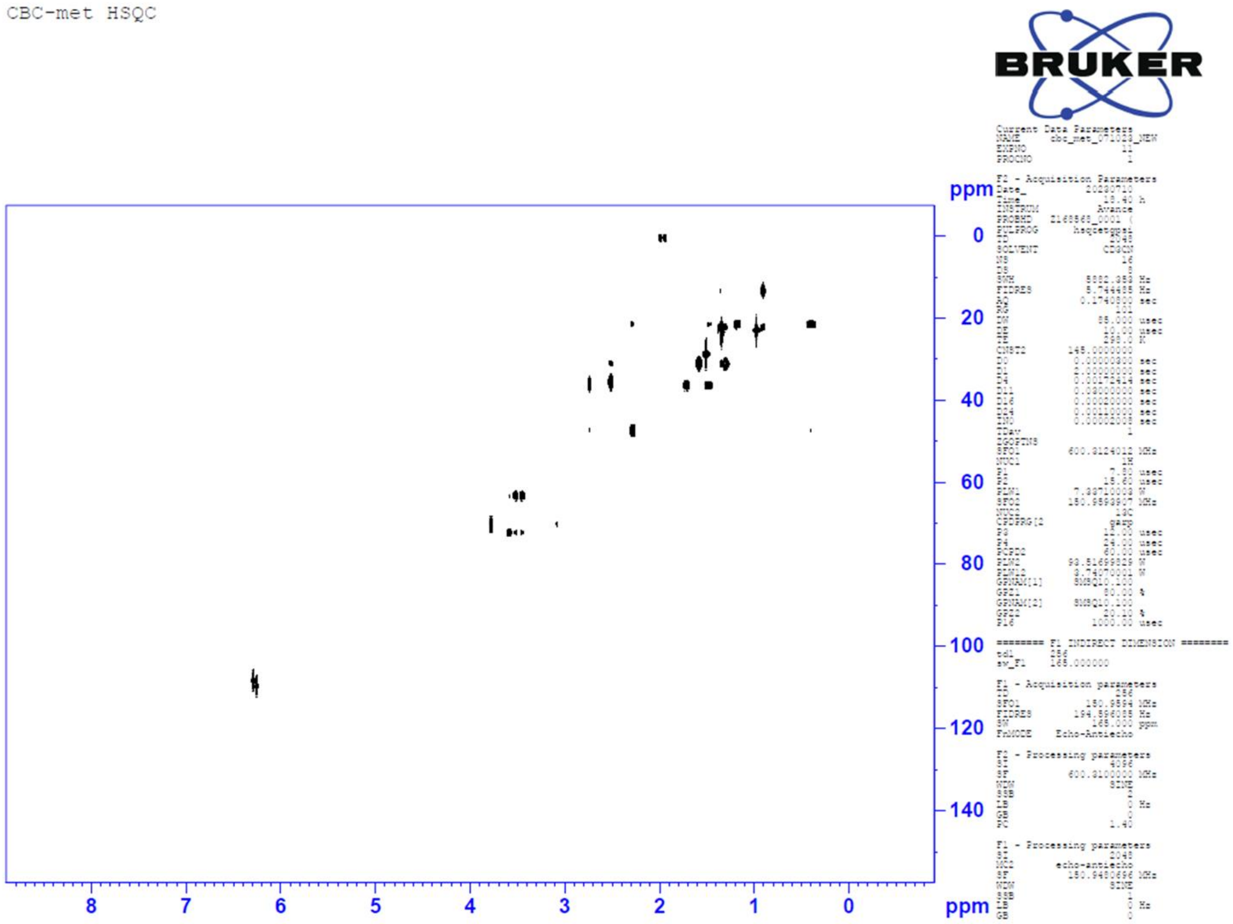

Figure S3.3.20. HSQC NMR spectrum of 2'-hydroxycannabicitran in ACN-d_3.

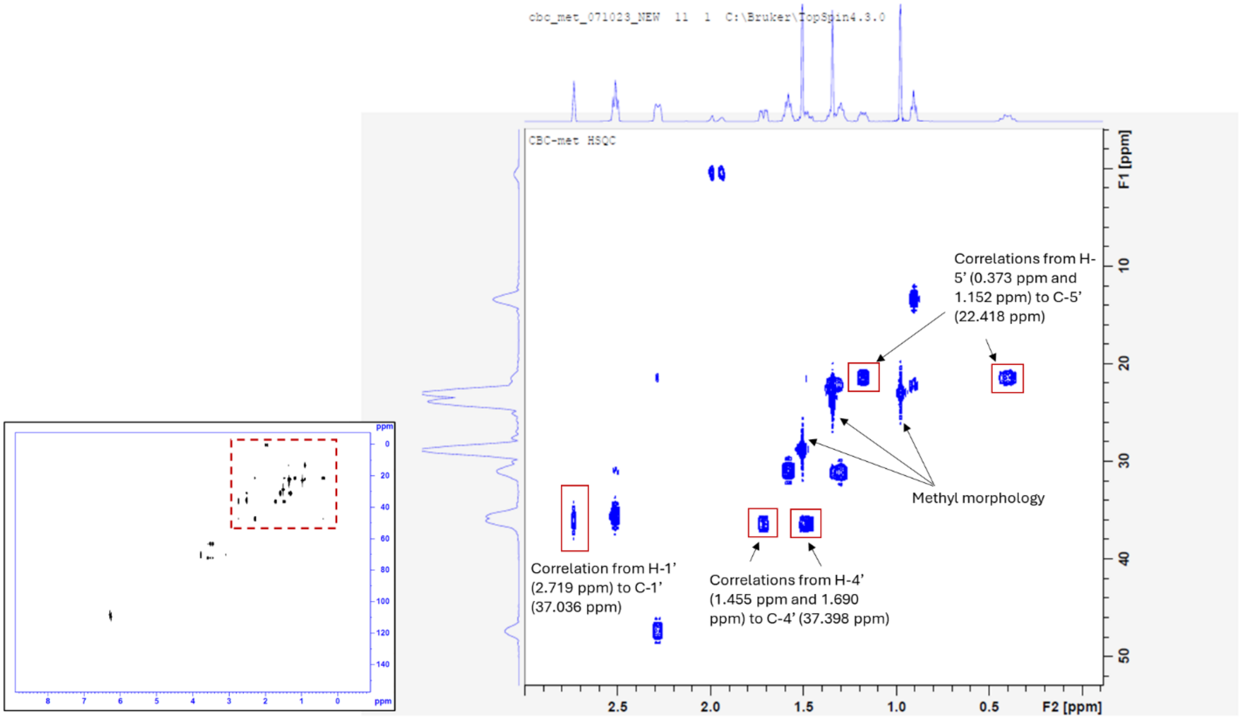

Figure S3.3.21. Zoomed-in HSQC NMR spectrum of 2'-hydroxycannabicitran in ACN-d_3. Assignments based on the structure in Figure S3.3.1. The red dotted line in the inset shows the zoomed-in range of the full spectrum.

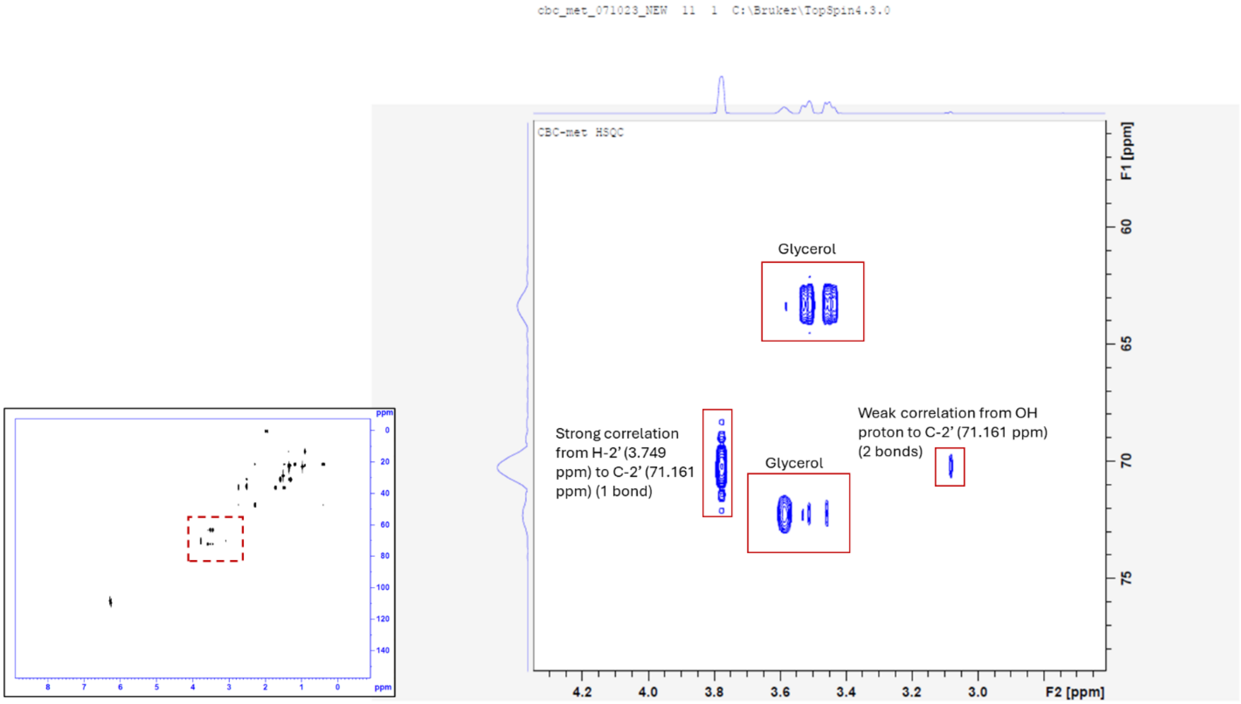

Figure S3.3.22. Zoomed-in HSQC NMR spectrum of 2'-hydroxycannabicitran in ACN-d_3. Assignments based on the structure in Figure S3.3.1. The red dotted line in the inset shows the zoomed-in range of the full spectrum.

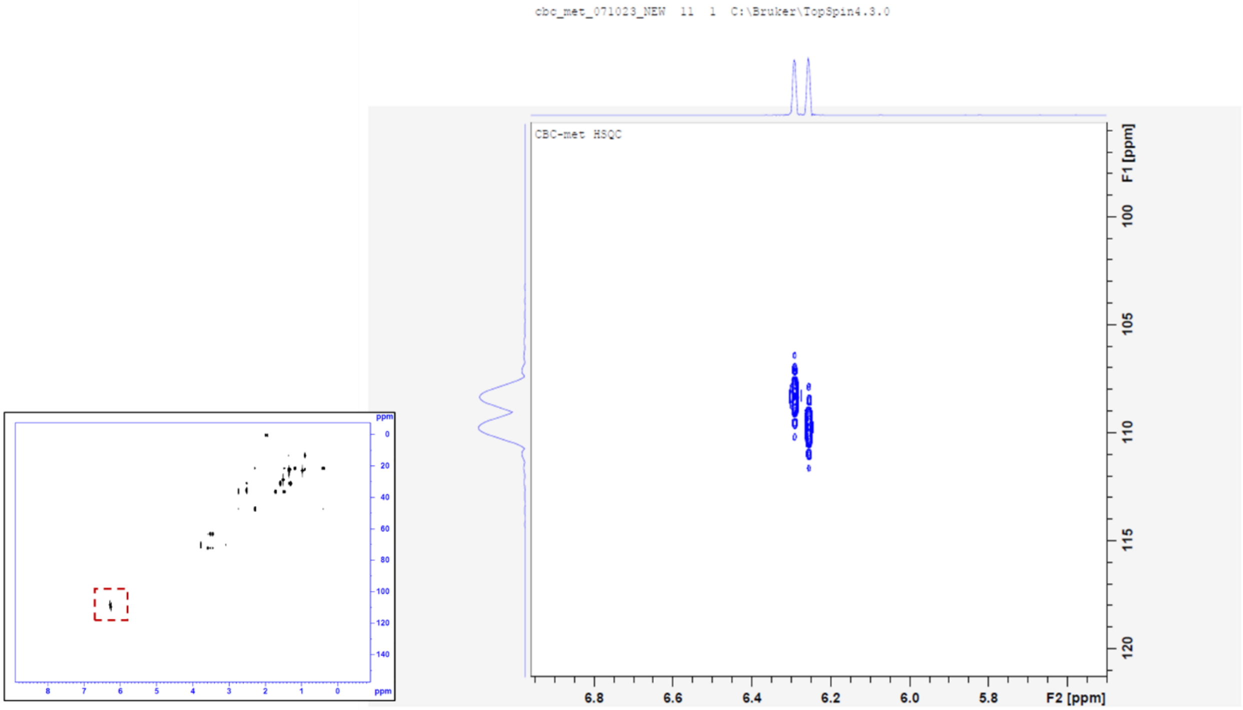

Figure S3.3.23. Zoomed-in HSQC NMR spectrum of 2’-hydroxycannabicitran in ACN-d_3. The red dotted line in the inset shows the zoomed-in range of the full spectrum.

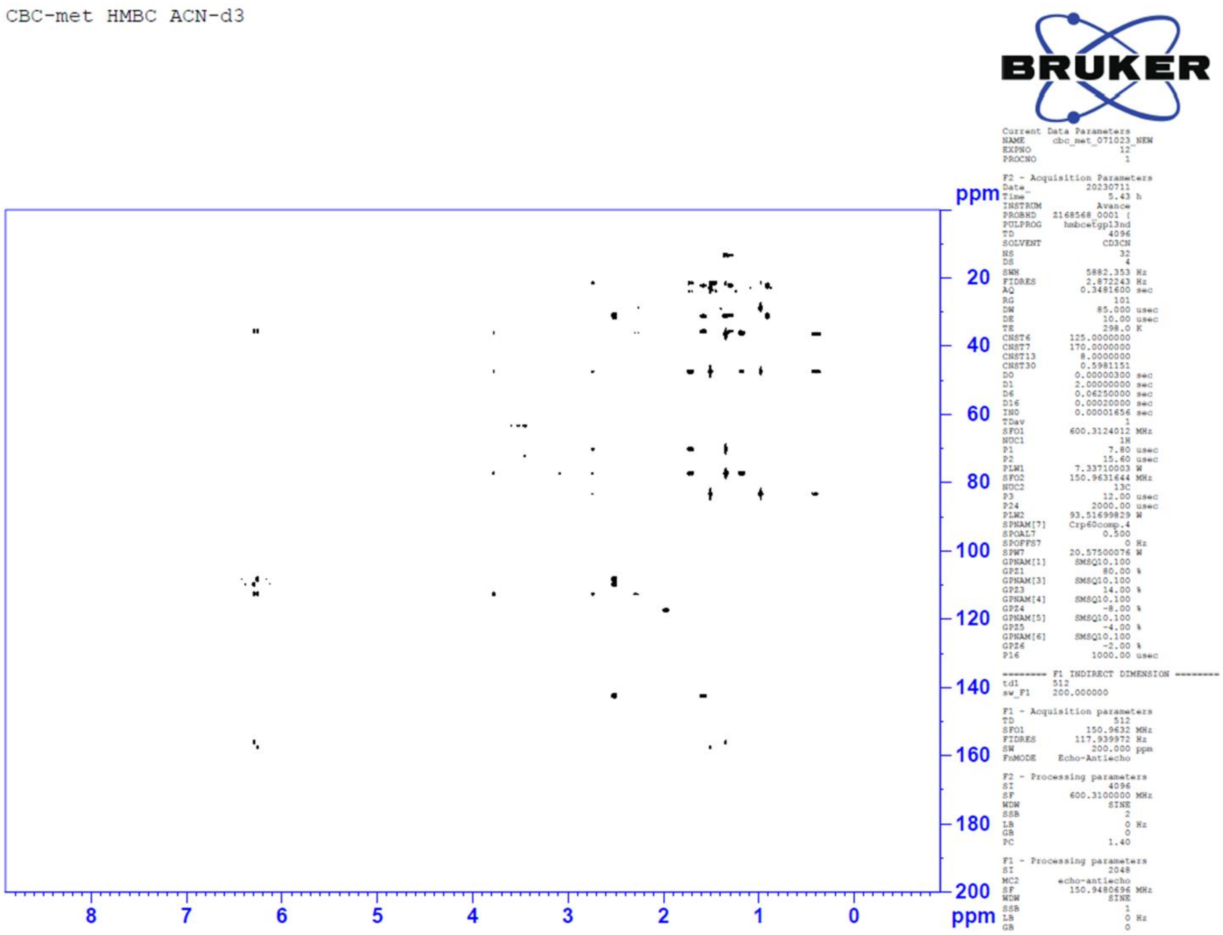

Figure S3.3.24. HMBC NMR spectrum of 2’-hydroxycannabicitran in ACN-d_3.

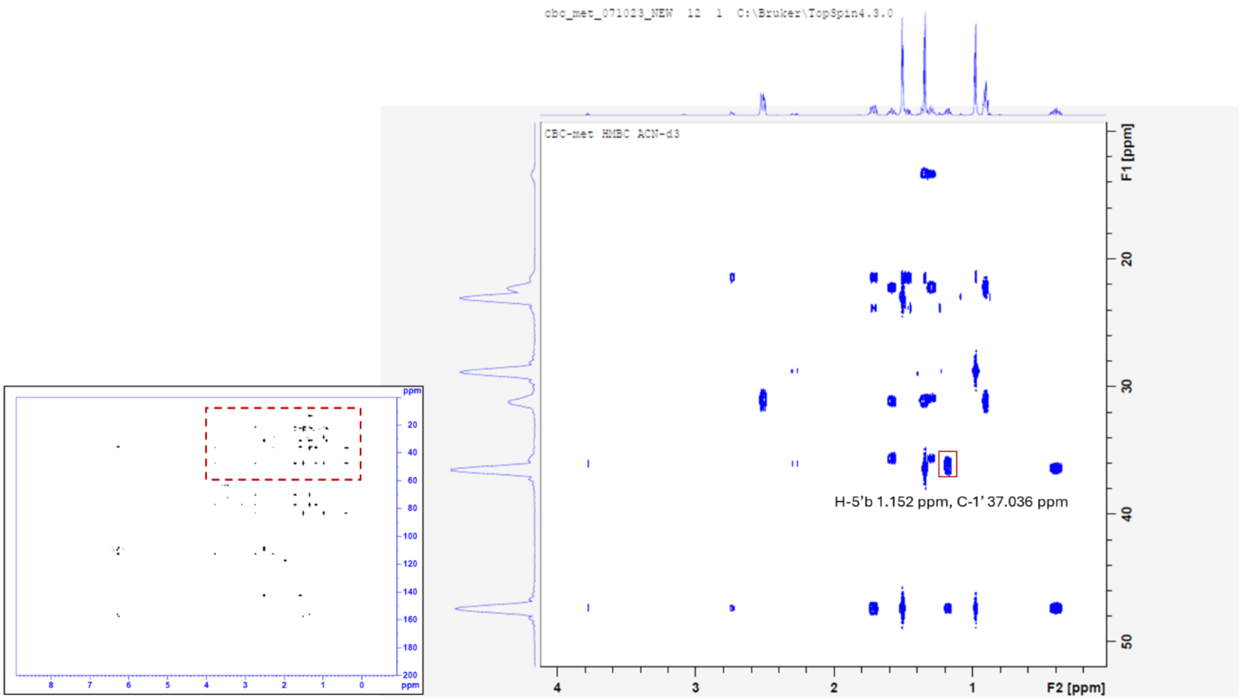

Figure S3.3.25. Zoomed-in HMBC NMR spectrum of 2'-hydroxycannabicitran in ACN-d_3. Assignments based on the structure in Figure S3.3.1. The red dotted line in the inset shows the zoomed-in range of the full spectrum.

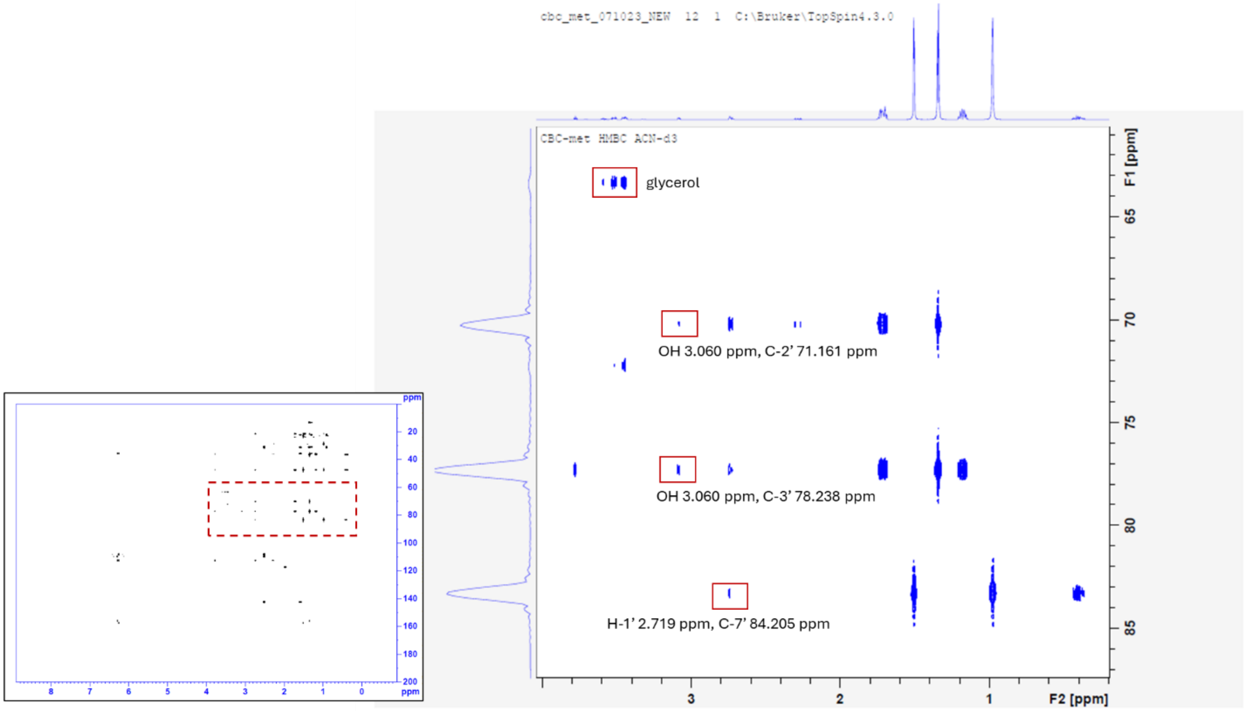

Figure S3.3.26. Zoomed-in HMBC NMR spectrum of 2'-hydroxycannabicitran in ACN-d_3. Assignments based on the structure in Figure S3.3.1. The red dotted line in the inset shows the zoomed-in range of the full spectrum.

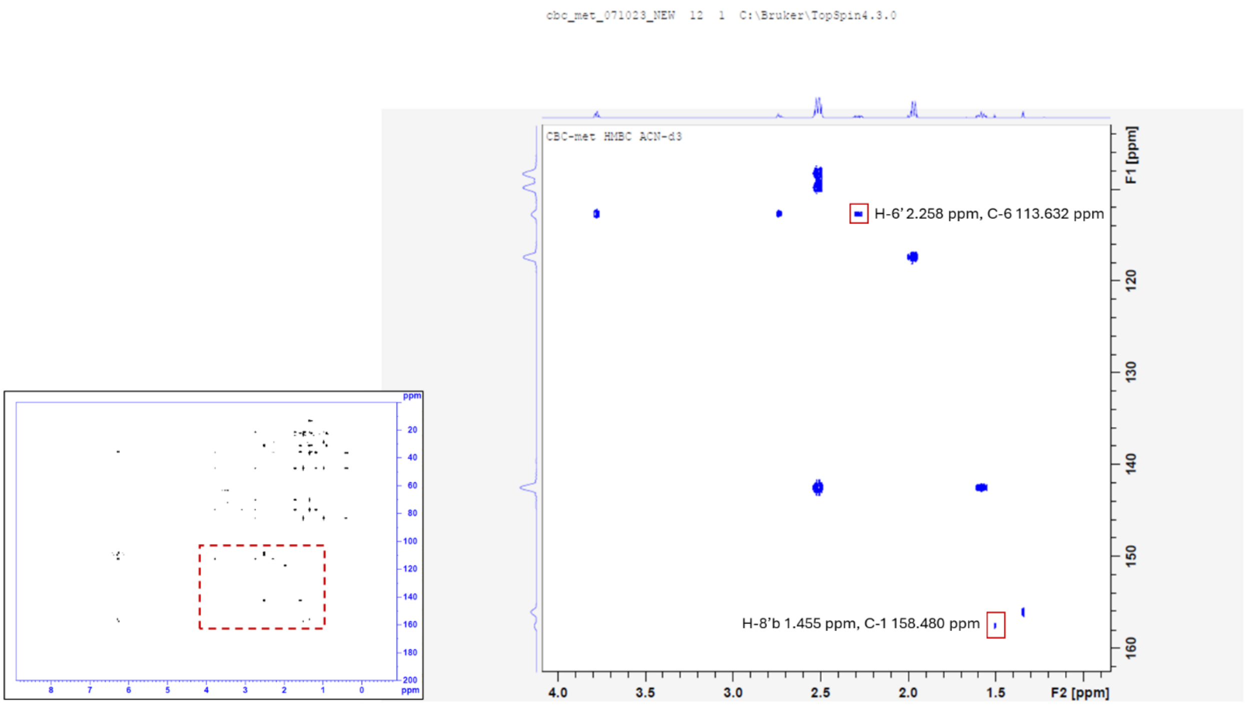

Figure S3.3.27. Zoomed-in HMBC NMR spectrum of 2'-hydroxycannabicitran in ACN-d_3. Assignments based on the structure in Figure S3.3.1. The red dotted line in the inset shows the zoomed-in range of the full spectrum.

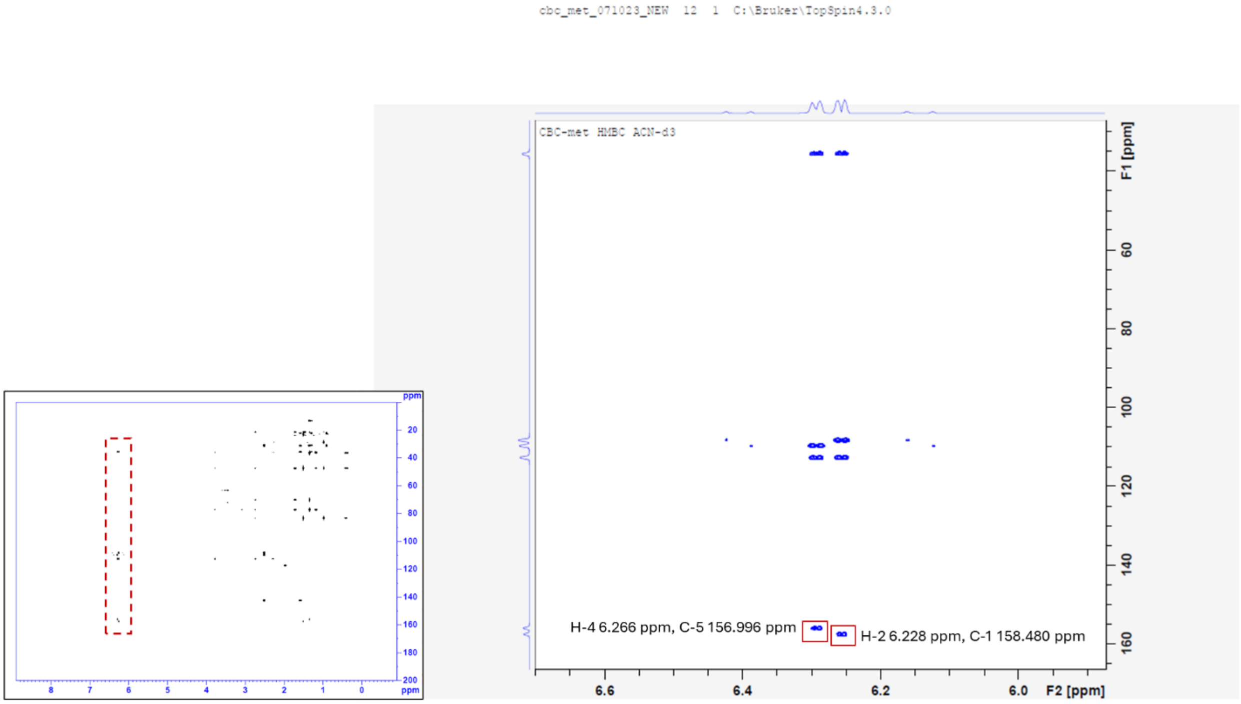

Figure S3.3.28. Zoomed-in HMBC NMR spectrum of 2'-hydroxycannabicitran in ACN-d_3. Assignments based on the structure in Figure S3.3.1. The red dotted line in the inset shows the zoomed-in range of the full spectrum.

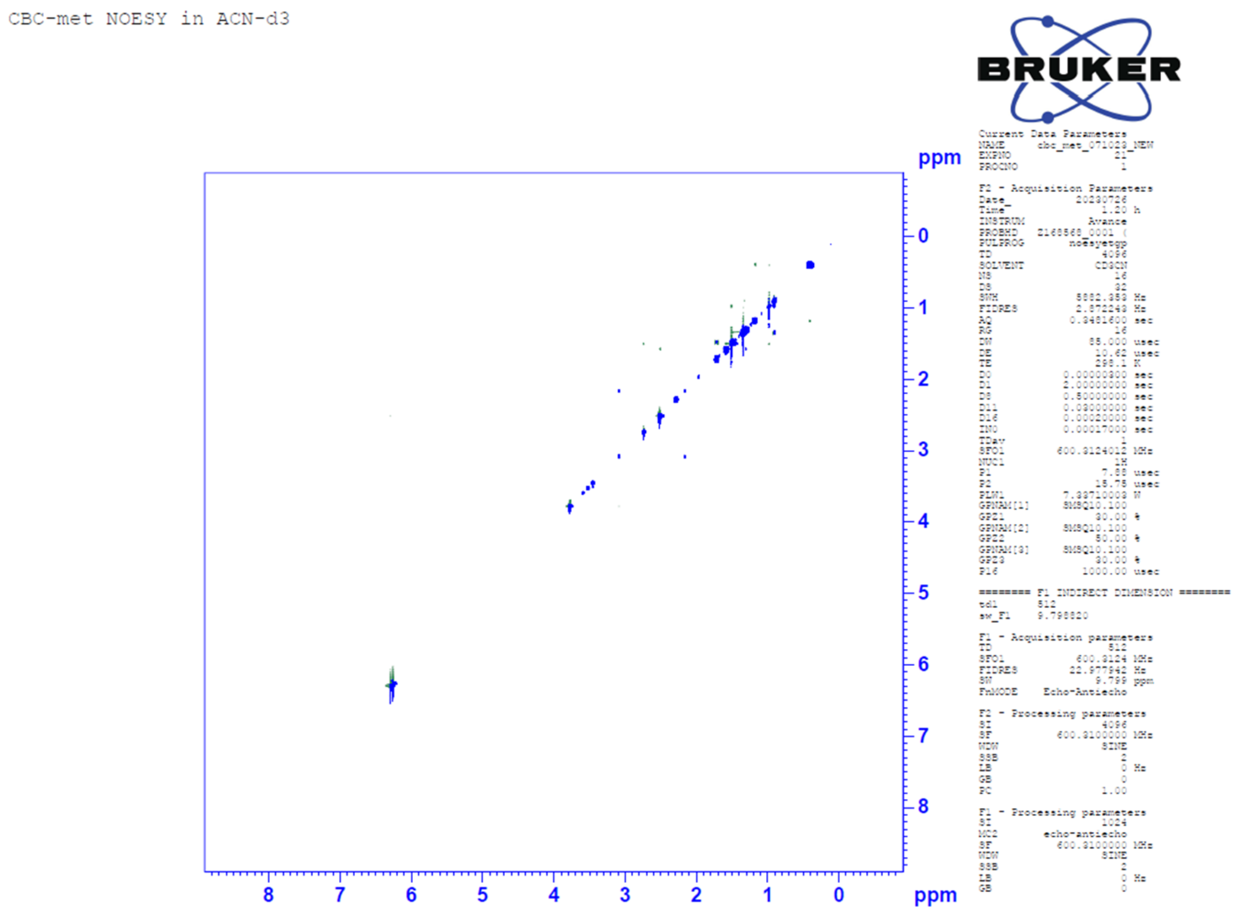

Figure S3.3.29. NOESY NMR spectrum of 2’-hydroxycannabicitran in ACN-d_3.

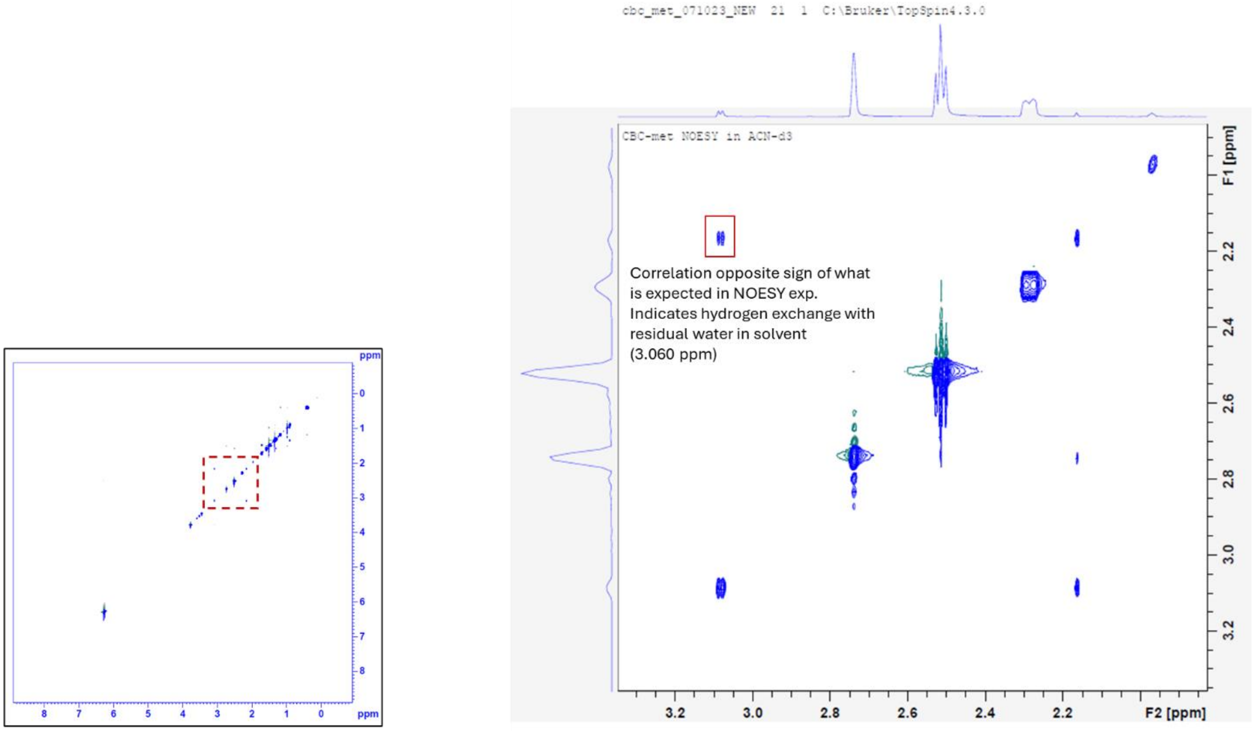

Figure S3.3.30. Zoomed-in NOESY NMR spectrum of 2'-hydroxycannabicitran in ACN-d_3. Assignments based on the structure in Figure S3.3.1. The red dotted line in the inset shows the zoomed-in range of the full spectrum.

S3.4

Cannabicitran and Overlay NMR Experiments

Due to 2’-hydroxycannabicitran’s structural similarity to CBT-C, we also analyzed CBT-C in ACN-d_3. Figures include NMR parameters, full spectra, and zoomed-in spectra with highlighted signals. Overlays of the spectra are shown and the major difference between the two spectra is at the C-2’ position, providing confidence for our structure assignment.

Please see the supplementary figures described below:

Chemical structure and numbering assignment of CBT-C: Figure S.3.4.1

^{1}H in ACN-d_3: Figures S3.4.2- S3.4.5, and Table 3.4.1

HSQC in ACN-d_3: Figures S3.4.6- S3.4.7

Overlay of 2’-hydroxycannabicitran and CBT-C: Figures S3.4.8- S3.4.11, and Table 3.4.2

Methods

^{1}H one dimensional NMR experiments were collected over a spectral width of 16 ppm using a total of 64K complex data points (R+I) giving an acquisition time of 3.14 s per fid with a 30 s relaxation delay between transients to ensure complete relaxation for accurate integration. There were 8-16 transients per experiment.

^{1}H-^{13}C Heteronuclear Single Quantum Coherence (HSQC) spectra were acquired using 2048 data points (R+I) over a spectral width of 9.8 ppm in the ^{1}H dimension and 256 data points (R+I) over 165 ppm in the C13 dimension. The transmitter offsets were centered at 4.0 ppm and 75 ppm respectively. 16 transients were acquired per FID using a 2 s relaxation delay. C13 decoupling during acquisition was achieved using GARP with a ^{13}C B1 field strength of 4.2 KHz centered at 75 ppm.

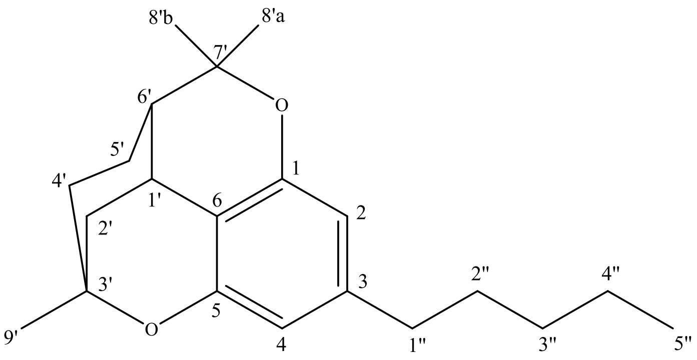

Chemical Formula: $C_{21}H_{30}O_2$
Exact Mass: 314.22458

Figure S3.4.1. Numbered structure of CBT-C according to a terpenoid system of numbering [36, 37].

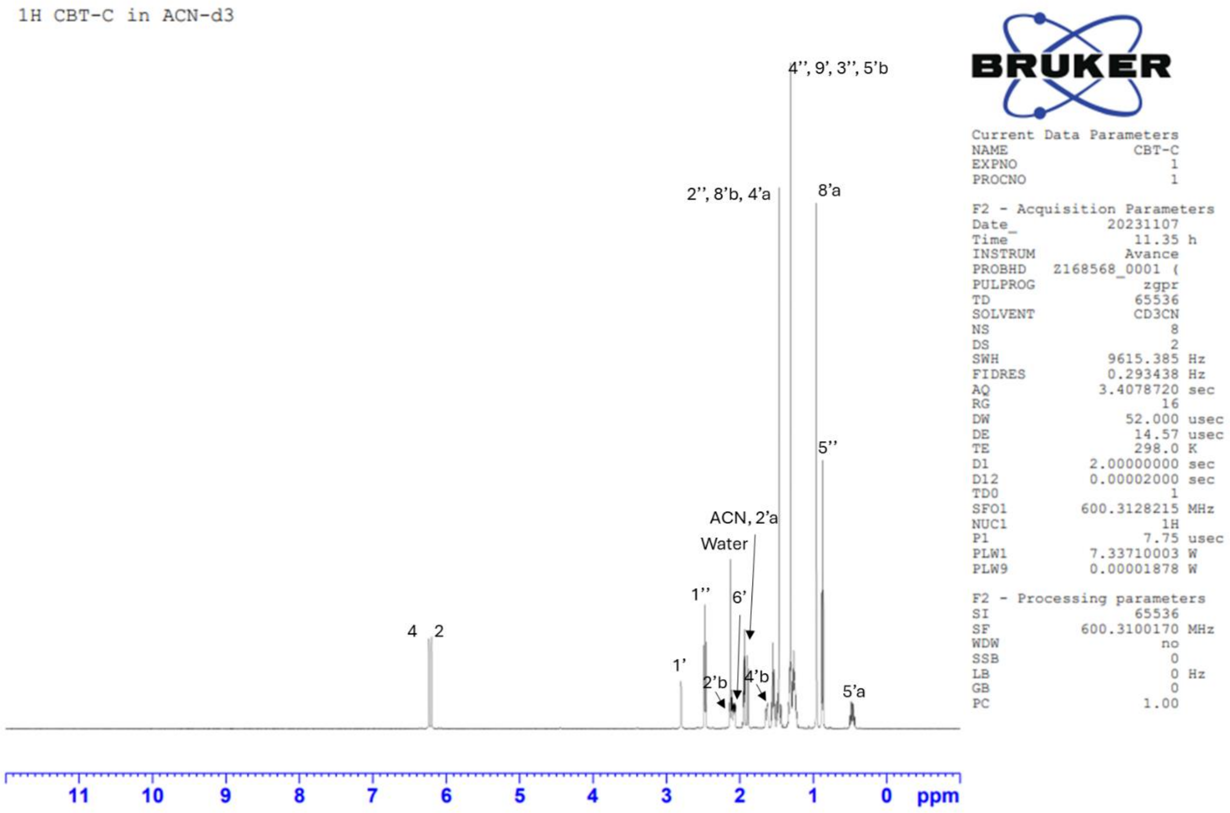

Figure S3.4.2. ^{1}H NMR spectrum of CBT-C in ACN-d_3. Assignments based on the structure in Figure S3.4.1.

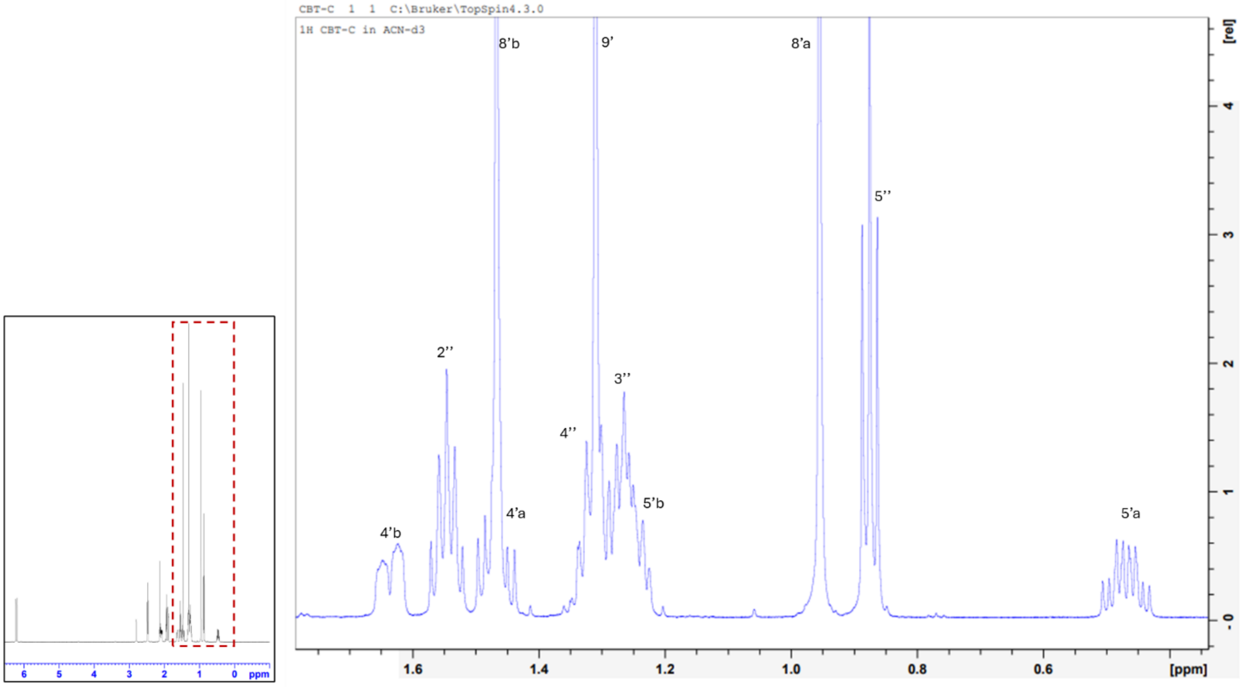

Figure S3.4.3. Zoomed-in ¹H NMR spectrum of CBT-C in ACN-d_3. Assignments based on the structure in Figure S3.4.1. The red dotted line in the inset shows the zoomed-in range of the full spectrum.

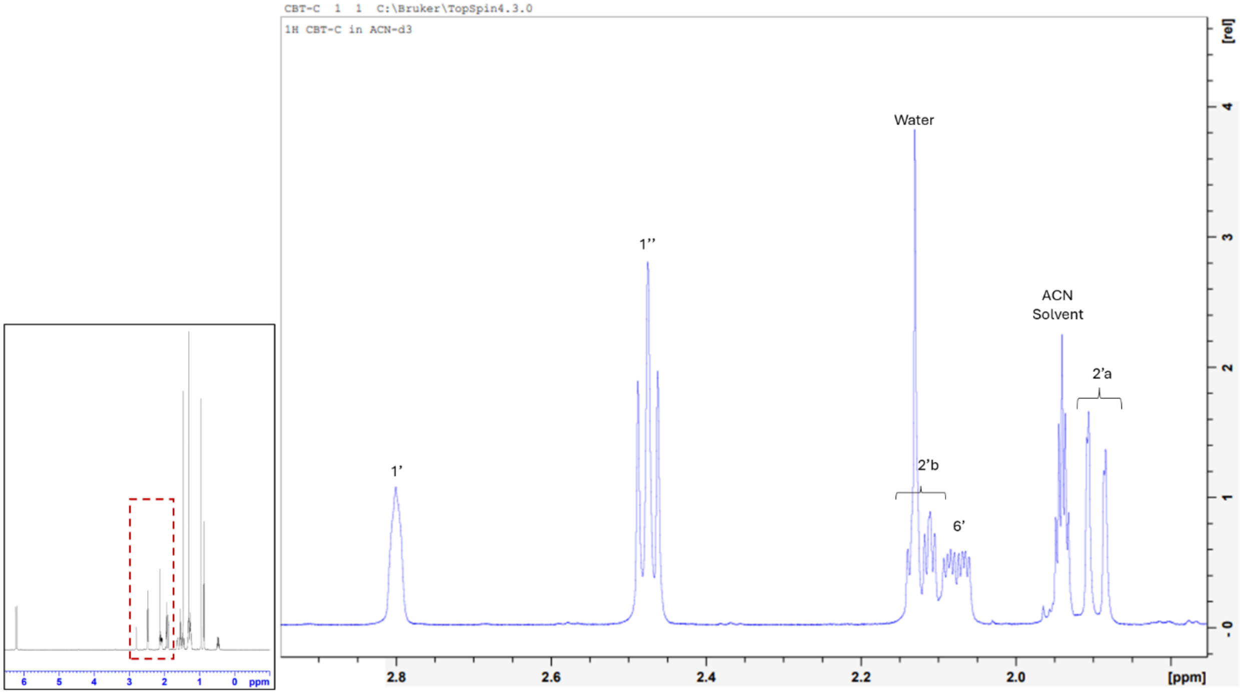

Figure S3.4.4. Zoomed-in ¹H NMR spectrum of CBT-C in ACN-d_3. Assignments based on the structure in Figure S3.4.1. The red dotted line in the inset shows the zoomed-in range of the full spectrum.

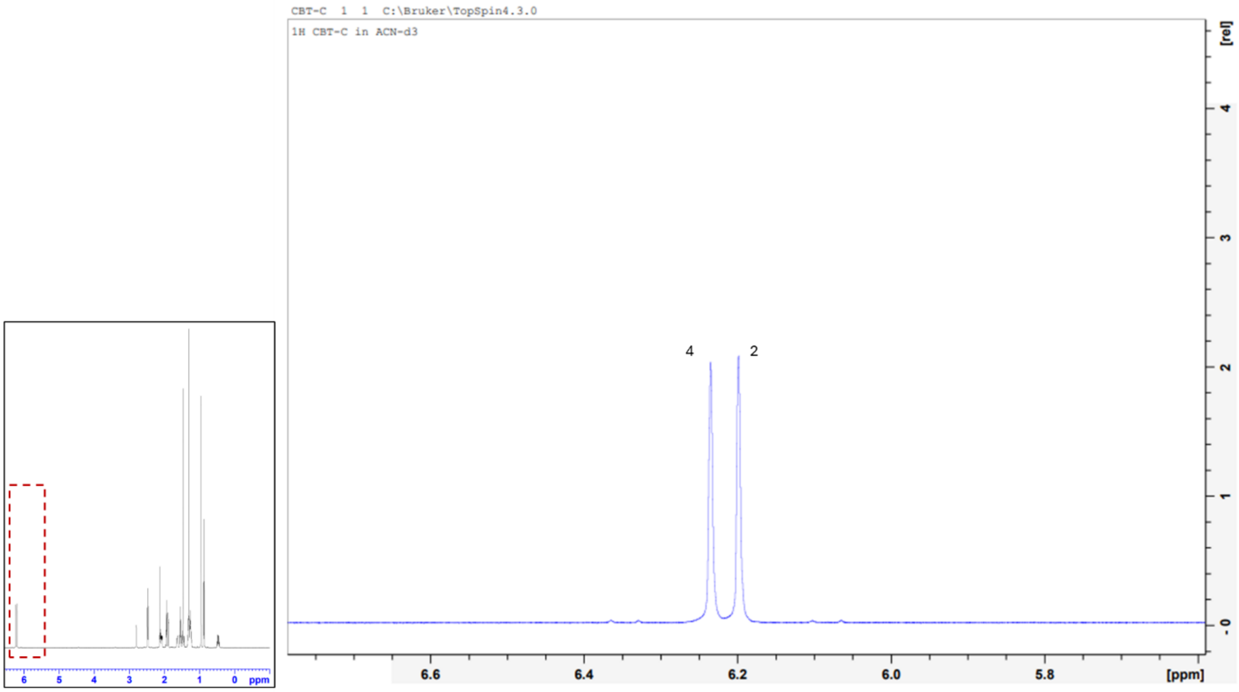

Figure S3.4.5. Zoomed-in ^{1}H NMR spectrum of CBT-C in ACN-d_3. Assignments based on the structure in Figure S3.4.1. The red dotted line in the inset shows the zoomed-in range of the full spectrum.

Table S3.4.1. ^{1}H peak assignments for CBT-C in ACN-d_3. Chemical shifts were aligned to the residual solvent peak of acetonitrile.

Cannabicitran ^{1}H NMR Peak Assignments

^{1}H Chemical Shift (δ ppm)	^{1}H Multiplicity *J* (Hz)	Assignment	Integration
6.234	s	4	1H
6.198	s	2	1H
2.803	t (2.5)	1'	1H
2.477	dd (7.7)	1''	2H
2.127	m	2'b	1H
2.075	ddd (3.0, 5.3, 11.5)	6'	1H
1.893	m	2'a	1H
1.638	m	4'b	1H
1.546	m	2''	2H
1.468	s	8'b	4H
1.468	m	4'a	
1.321	m	4''	7H
1.319	s	9'	
1.274	m	3''	
1.255	m	5'b	
0.957	s	8'a	3H
0.874	t (7.1)	5''	3H
0.469	tdd (6.1, 11.5, 13.4)	5'a	1H

5’b was buried under the 7H multiplet and should have integrated for 8H. The 5’b proton was resolved in HSQC data.

A selected portion of the HSQC spectra is shown in Figure S3.4.7.

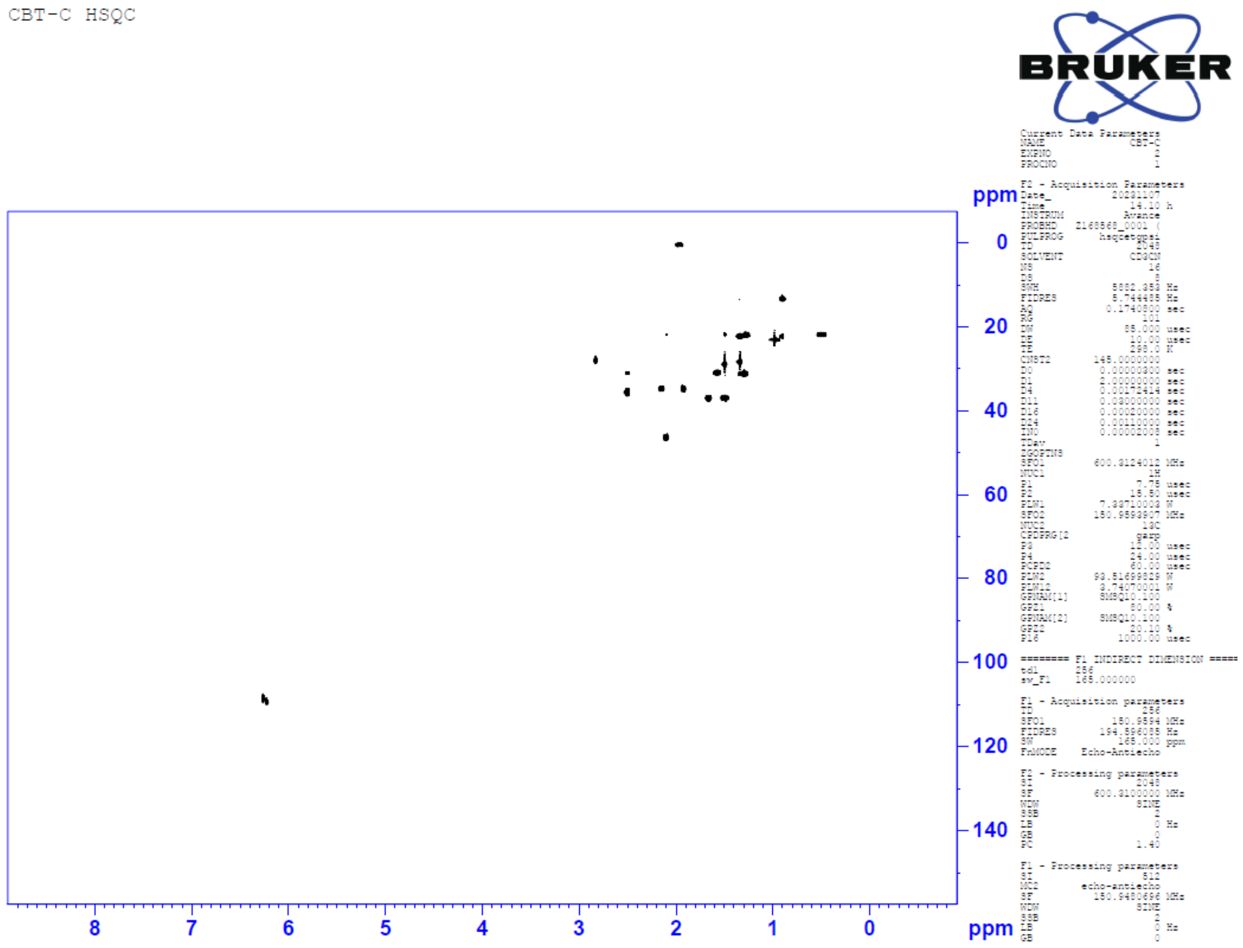

Figure S3.4.6. HSQC NMR spectrum of CBT-C in ACN-d_3.

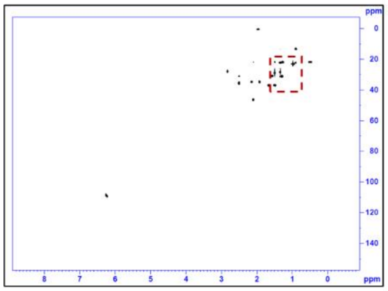

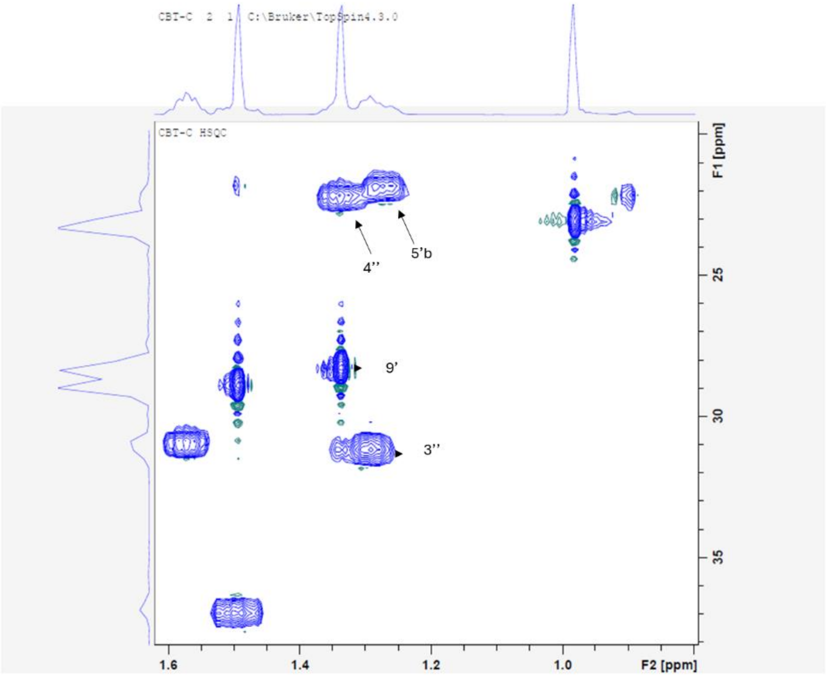

Figure S3.4.7. Zoomed-in HSQC NMR spectrum of CBT-C in ACN-d_3. Assignments based on the structure in Figure S3.4.1. The red dotted line in the inset shows the zoomed-in range of the full spectrum.

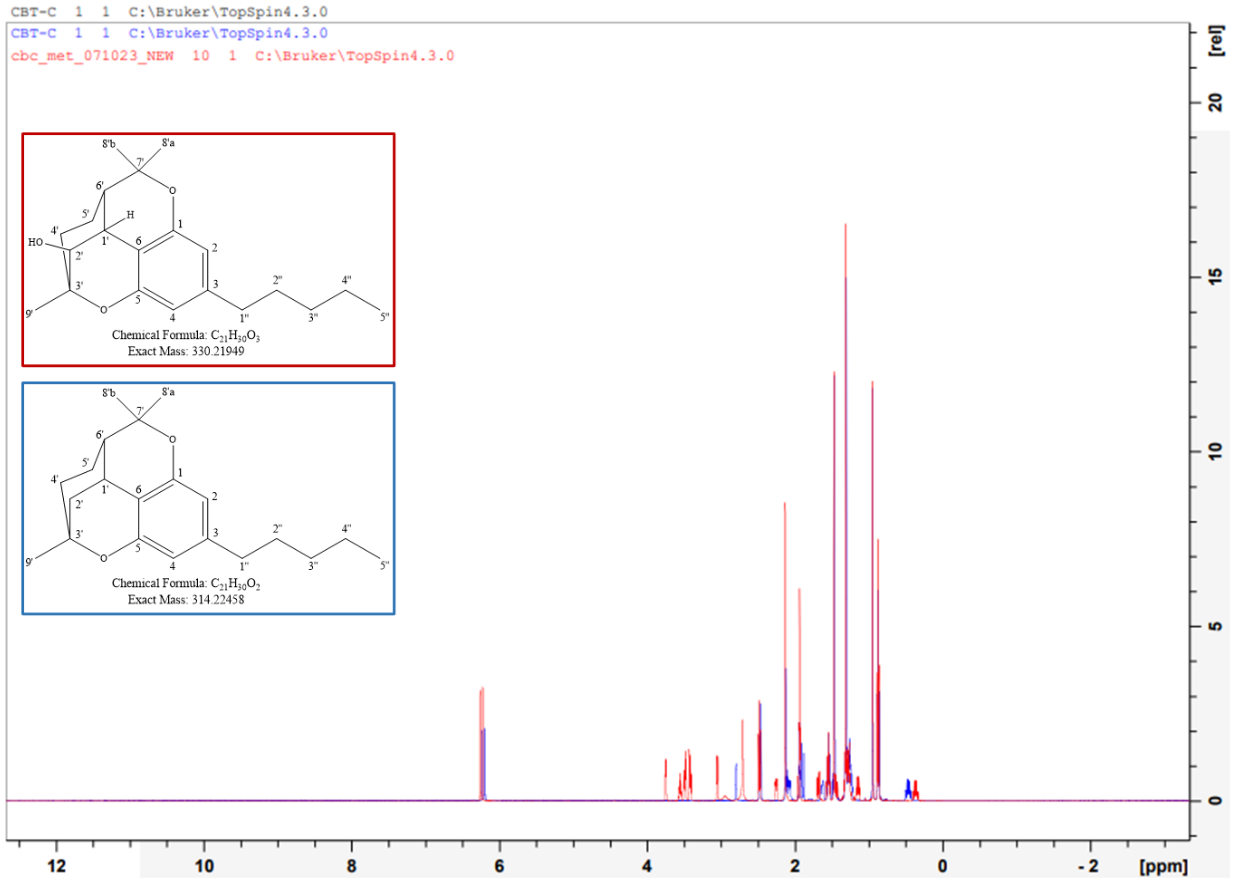

Figure S3.4.8. Overlaid ^{1}H NMR spectra from 2’-hydroxycannabicitran (red) and CBT-C (blue) in ACN-d_3.

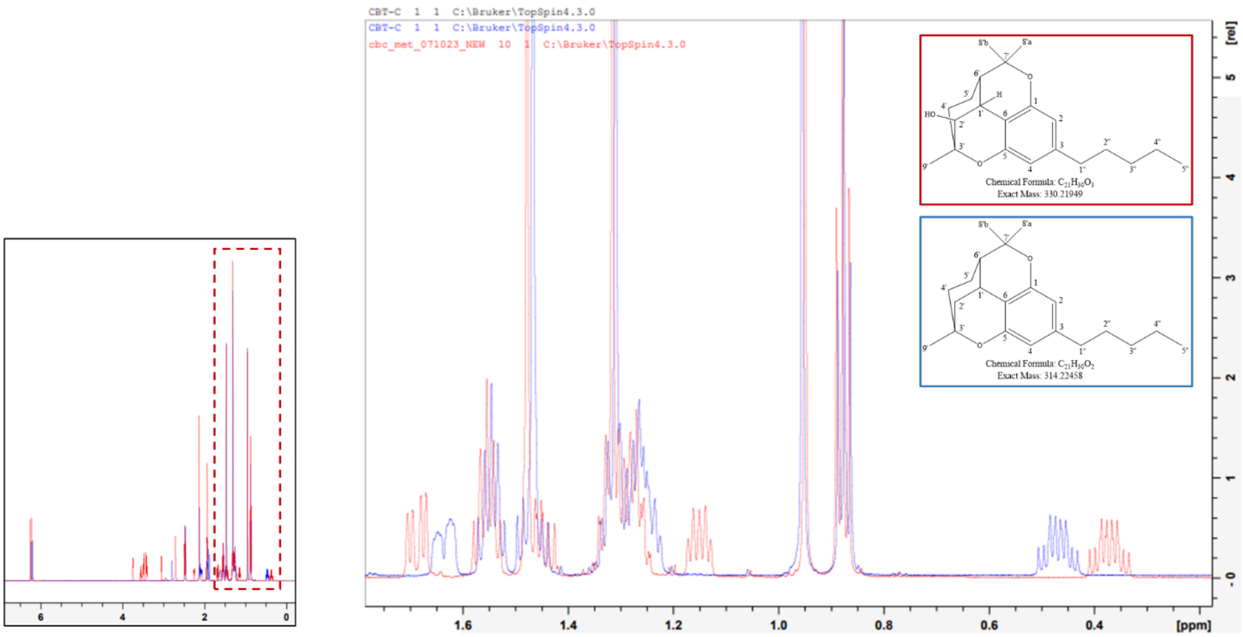

Figure S3.4.9. Overlaid ^{1}H NMR spectra from 2'-hydroxycannabicitran (red) and CBT-C (blue) in ACN-d_3. The red dotted line in the inset shows the zoomed-in range of the full spectrum.

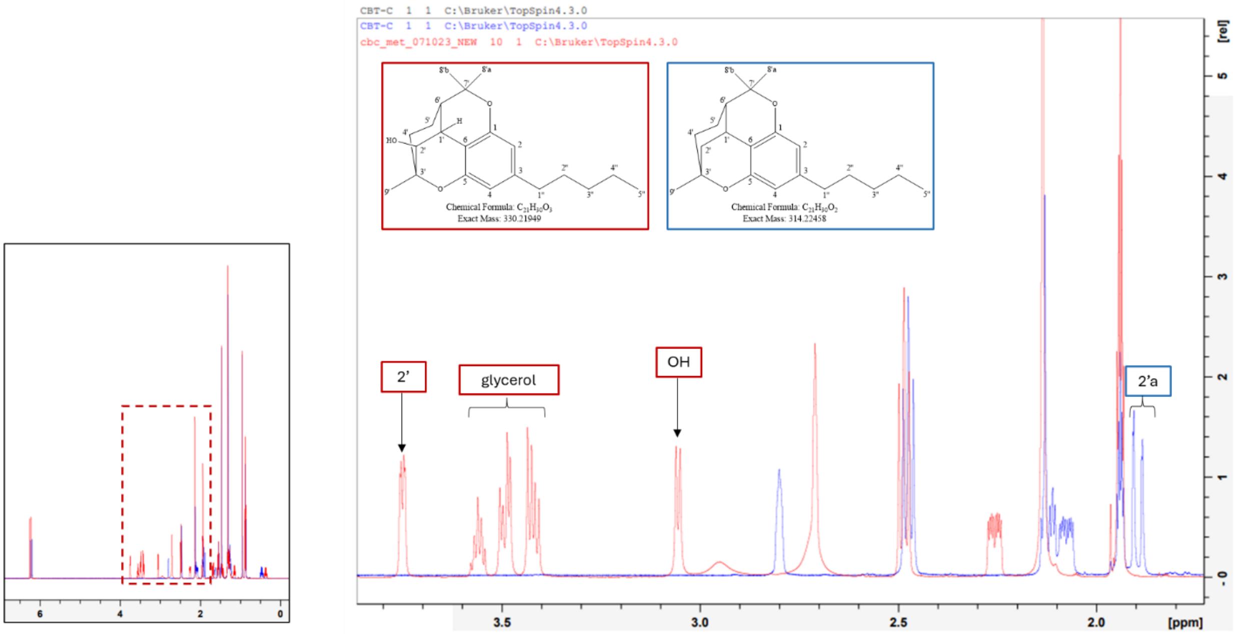

Figure S3.4.10. Overlaid ^{1}H NMR spectra from 2'-hydroxycannabicitran (red) and CBT-C (blue) in ACN-d_3. The red dotted line in the inset shows the zoomed-in range of the full spectrum.

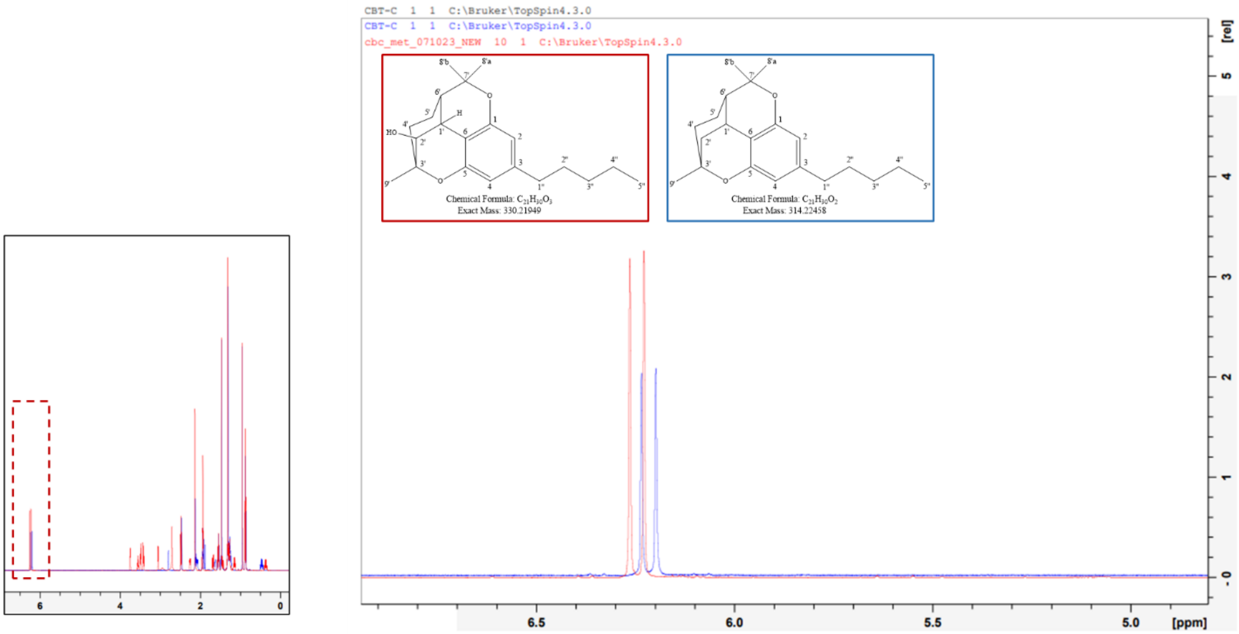

Figure S3.4.11. Overlaid ^{1}H NMR spectra from 2'-hydroxycannabicitran (red) and CBT-C (blue) in ACN-d_3. The red dotted line in the inset shows the zoomed-in range of the full spectrum.

Table S3.4.2. Comparison of ^{1}H peak assignments for 2'-hydroxycannabicitran and CBT-C in ACN-d_3. Chemical shifts were aligned to the residual solvent peak of acetonitrile. Highlighted rows indicate major differences between the two compounds.

2'-hydroxycannabicitran ^{1}H NMR Peak Assignments

[1]H Chemical Shift (δ ppm)	[1]H Multiplicity *J* (Hz)	Assignment	Integration
6.266	s	4	1H
6.228	s	2	1H
3.749	dd (1.9, 5.8)	2'	1H
3.06	d (6.1)	OH	1H
2.719	t (2.3)	1'	1H*
2.485	dd (7.4)	1''	2H
2.258	ddd (2.8, 5.3, 11.5)	6'	1H
1.69	ddd (0.9, 6.1, 15.4)	4'b	1H
1.554	m	2''	2H
1.478	s	8'b	4H
1.455	td (7.1, 15.3)	4'a	
1.321	m	4''	7H
1.315	s	9'	
1.27	m	3''	
1.152	dt (5.9, 12.8)	5'b	1H
0.948	s	8'a	3H
0.88	t (7.1)	5''	3H
0.373	tdd (6.2, 11.9, 13.4)	5'a	1H

Cannabicitran ^{1}H NMR Peak Assignments

[1]H Chemical Shift (δ ppm)	[1]H Multiplicity *J* (Hz)	Assignment	Integration
6.234	s	4	1H
6.198	s	2	1H
2.803	t (2.5)	1'	1H
2.477	dd (7.7)	1''	2H
2.127	m	2'b	1H
2.075	ddd (3.0, 5.3, 11.5)	6'	1H
1.893	m	2'a	1H
1.638	m	4'b	1H
1.546	m	2''	2H
1.468	s	8'b	4H
1.468	m	4'a	
1.321	m	4''	7H
1.319	s	9'	
1.274	m	3''	
1.255	m	5'b	
0.957	s	8'a	3H
0.874	t (7.1)	5''	3H
0.469	tdd (6.1, 11.5, 13.4)	5'a	1H

1H* refers to a peak that integrated on the 1H spectra for 2 protons but upon the results of the DEPT-135 experiment, was rectified to representing 1 proton.

References

36. Hanus LO, Meyer SM, Munoz E, Taglialatela-Scafati O, Appendino G. Phytocannabinoids: a unified critical inventory. Nat Prod Rep. 2016;33(12):1357-92. doi: 10.1039/c6np00074f.

37. Elsohly MA, Slade D. Chemical constituents of marijuana: the complex mixture of natural cannabinoids. Life Sci. 2005;78(5):539-48. doi: 10.1016/j.lfs.2005.09.011.

46. Ohtsuki T, Friesen JB, Chen S, McAlpine JB, Pauli GF. Selective Preparation and High Dynamic-Range Analysis of Cannabinoids in "CBD Oil" and Other Cannabis sativa Preparations. J Nat Prod. 2022;85(3):634-46. doi: 10.1021/acs.jnatprod.1c00976.
