# Supplementary material for: Identification and Characterization of Cannabichromene’s Major Metabolite Following Incubation with Human Liver Microsomes
Source: Metabolites. 2024 Jun 13;14(6):329. doi: 10.3390/metabo14060329 (PMC11206029; doi:10.3390/metabo14060329)
Supplement: Supplementary file 1 [file metabolites-14-00329-s001.zip › CBC_Metabolite_Supplementary_Materials_S4.pdf]

Supplemental Materials S4

Identification and Characterization of Cannabichromene's Major Metabolite Following Incubation with Human Liver Microsomes

Alexandra M. Ward[1], Touraj Shokati[2], Jost Klawitter[2], Jelena Klawitter[2], Vu Nguyen[1], Laura Kozell[3, 4, 5], Atheir I. Abbas[3, 4, 5], David Jones[6], and Uwe Christians[2,*]

[1]*Department of Pharmaceutical Sciences, Skaggs School of Pharmacy and Pharmaceutical Sciences, University of Colorado Anschutz Medical Campus, Aurora, CO, USA; alexandra.ward@cuanschutz.edu, vu.t.nguyen@cuanschutz.edu.*

[2]*iC42 Clinical Research and Development, Department of Anesthesiology, School of Medicine, University of Colorado Anschutz Medical Campus, Aurora, CO, USA; touraj.shokati@cuanschutz.edu, jost.klawitter@cuanschutz.edu, jelena.klawitter@cuanschutz.edu, uwe.christians@cuanschutz.edu.*

[3]*Department of Behavioral Neuroscience, Oregon Health & Science University, Portland, OR, USA; abbasat@ohsu.edu, kozellla@ohsu.edu.*

[4]*Department of Psychiatry, Oregon Health & Science University, Portland, OR, USA.*

[5]*Veterans Affairs Portland Health Care System, Portland, OR, USA.*

[6]*Department of Pharmacology, School of Medicine, University of Colorado Anschutz Medical Campus, Aurora, CO, USA; david.jones@cuanschutz.edu.*

*Correspondence: uwe.christians@cuanschutz.edu

Table of Contents Supplemental Materials S4

S4.1
Structures Imported for Molecular Docking Page 3

S4.2
Cannabinoid 1 Receptor Molecular Docking Page 5

S4.3
Cannabinoid 2 Receptor Molecular Docking Page 30

References Page 51

S4.1

Structures Imported for Molecular Docking

Structures were drawn of various test ligands to be docked into the orthosteric site of CB_1R and CB_2R.

Please see supplementary Figure S.4.1.1 for the structures.

Methods

Chemical structures were drawn using ChemDraw (version 19.0, PerkinElmer, Waltham, MA, USA).

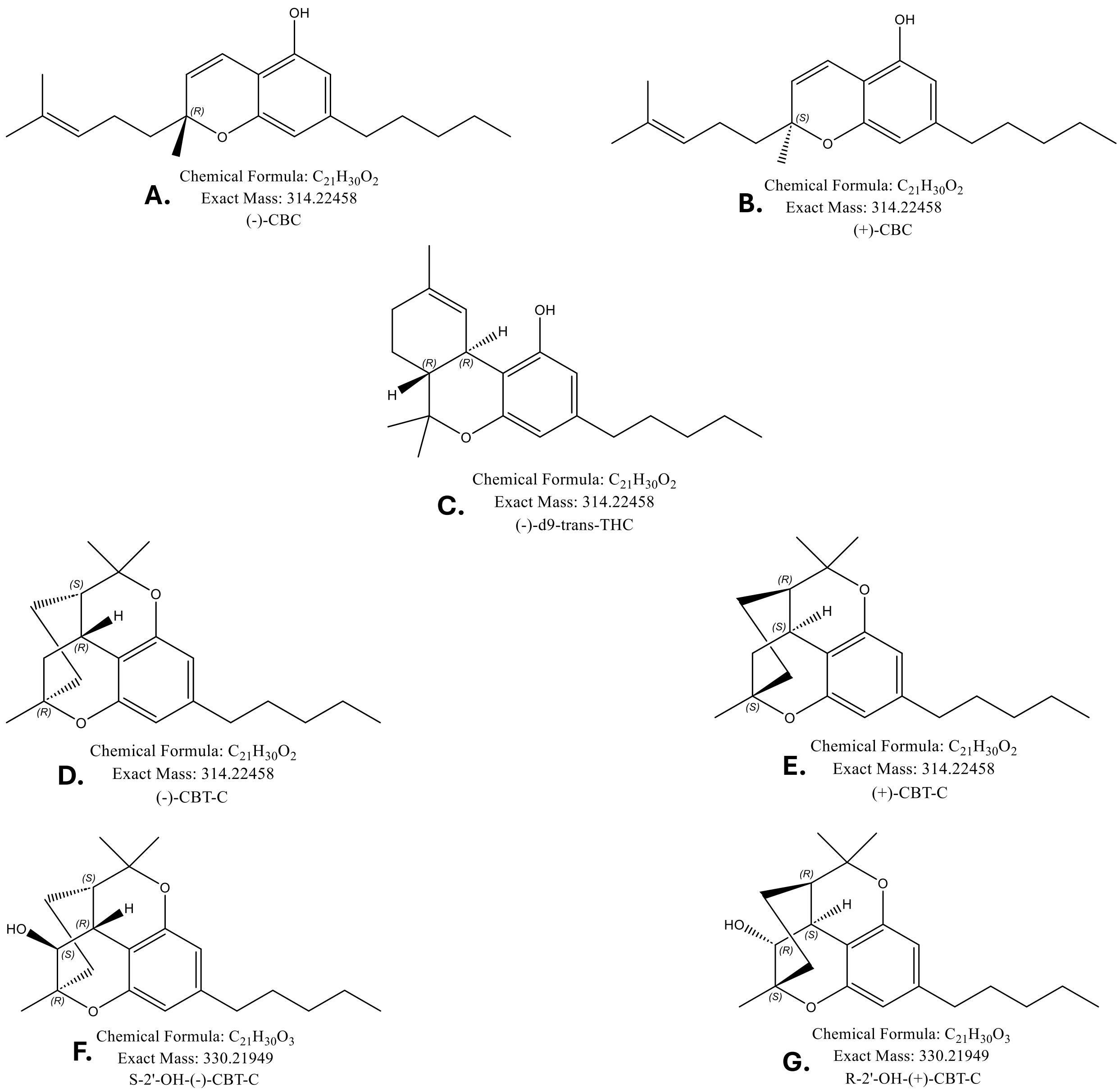

Figure S4.1.1. Chemical structures docked into the orthosteric site of CB_1R and CB_2R. (A) (-)-CBC, (B) (+)-CBC, (C) (-)-Δ^9-trans-THC, (D) (-)-CBT-C, (E) (+)-CBT-C, (F) S-2'-hydroxy-(-)-cannabicitran, and (G) R-2'-hydroxy-(+)-cannabicitran.

S4.2

Cannabinoid 1 Receptor Molecular Docking

Structures were docked into the orthosteric site of CB_1R. Both Standard-Precision (SP) and Extra-Precision (XP) glide models were used to rank the test ligands.

Predicted interactions could be used to determine if there is potential for binding of 2'-hydroxycannabicitran at the cannabinoid receptors. The results of this experiment would inform if *in vitro* testing would be valuable to move forward with.

Please refer to the main manuscript text for a discussion of the molecular docking results.

Please see supplementary Table S4.2.1 for an SP model of ligands and interactions ranked in the orthosteric site of CB_1R. Tables S4.2.2-S4.2.8 highlight each tested ligand and Figures S4.2.1-S4.2.14 display two- and three- dimensional figures of the ligands docked into the active site.

Please see supplementary Table S4.2.9 for an XP model of ligands and interactions ranked in the orthosteric site of CB_1R. Tables S4.2.10-S4.2.13 highlight each tested ligand and Figures S4.2.15-S4.2.22 display two- and three- dimensional figures of the ligands docked into the active site.

Methods

Schrödinger Suite (release 2023-3, Schrödinger, New York, NY, USA) was used for all ligand and protein preparation, and the Glide Module for all docking calculations [39-41]. Using LigPrep, the following compounds were prepared at physiological pH: (-)-Δ9-THC, (+)-CBC, (-)-CBC, (R)-2'-hydroxy-(+)-cannabicitran, (S)-2'-hydroxy-(-)-cannabicitran, (+)-CBT-C, and (-)-CBT-C (structures are shown in Figure S4.1.1.). CB_1R was imported from Protein Data Bank (PDB code: 5XR8) [42]. These protein structures were prepared by removing all water molecules and co-crystallized ligands, and then minimized with OPLS4 force fields and the VSGB solvation model [44]. CB_1R were scanned and mapped to confirm their binding site. Computational grids were then formed around the mapped binding sites to have the test compounds docked into. This docking was completed with Standard-Precision (SP) and Extra-Precision (XP) glide models for comparison. Top-ranked ligand-protein conformations were produced with corresponding scoring function values.

Table S4.2.1. Ligand ranking and interactions in the orthosteric site of the CB_1R: SP model.

CB_1R SP model						
Computer Ranking	Ligand	Docking Score	Glide emodel	Highlighted Residue-Ligand Interactions	Distance of Interaction (Å)	Type of Interaction
1	(+)-CBC	-10.600	-69.166	Ser505-OH	1.69	H-bond
				Phe170-AR	3.70	π-π stacking
				Phe170-P	3.70	π-π stacking
				Phe268-AR	3.78	π-π stacking
				Phe268-P	3.78	π-π stacking
2	(-)-Δ^9-THC	-10.569	-70.434	Ser505-OH	1.84	H-bond
				Phe268-AR	3.75	π-π stacking
				Phe170-AR	3.84	π-π stacking
3	(-)-CBC	-9.668	-63.781	Ser505-OH	1.81	H-bond
				Phe174-AR	3.33	π-π stacking
				Phe268-P	3.46	π-π stacking
				Phe268-AR	3.62	π-π stacking
				Phe170-AR	3.70	π-π stacking
				Phe170-P	3.70	π-π stacking
4	(R)-2'-OH-(+)-cannabicitran	-9.254	-52.811	Ile267-OH	2.49	H-bond
5	(+)-CBT-C	-8.345	-35.785	Phe170-AR	3.38	π-π stacking
6	(-)-CBT-C	-7.967	-38.936	Phe170-AR	3.55	π-π stacking
				Phe268-AR	3.55	π-π stacking
7	(S)-2'-OH-(-)-cannabicitran	-6.796	-27.572	Phe170-AR	3.23	π-π stacking

Distances were measured based on the closest atom to atom distance between a given residue and the ligand. Abbreviations: Aromatic ring (AR), pyran-type ring (P), and hydrogen bond (H-bond).

Table S4.2.2. (+)-CBC in the orthosteric site of the CB_1R: SP model.

				**CB_1R SP model**		
Computer Ranking	Ligand	Docking Score	Glide emodel	Highlighted Residue-Ligand Interactions	Distance of Interaction (Å)	Type of Interaction
1	(+)-CBC	-10.600	-69.166	Ser505-OH	1.69	H-bond
				Phe170-AR	3.70	π-π stacking
				Phe170-P	3.70	π-π stacking
				Phe268-AR	3.78	π-π stacking
				Phe268-P	3.78	π-π stacking

Distances were measured based on the closest atom to atom distance between a given residue and the ligand. Abbreviations: Aromatic ring (AR), pyran-type ring (P), hydrogen bond (H-bond).

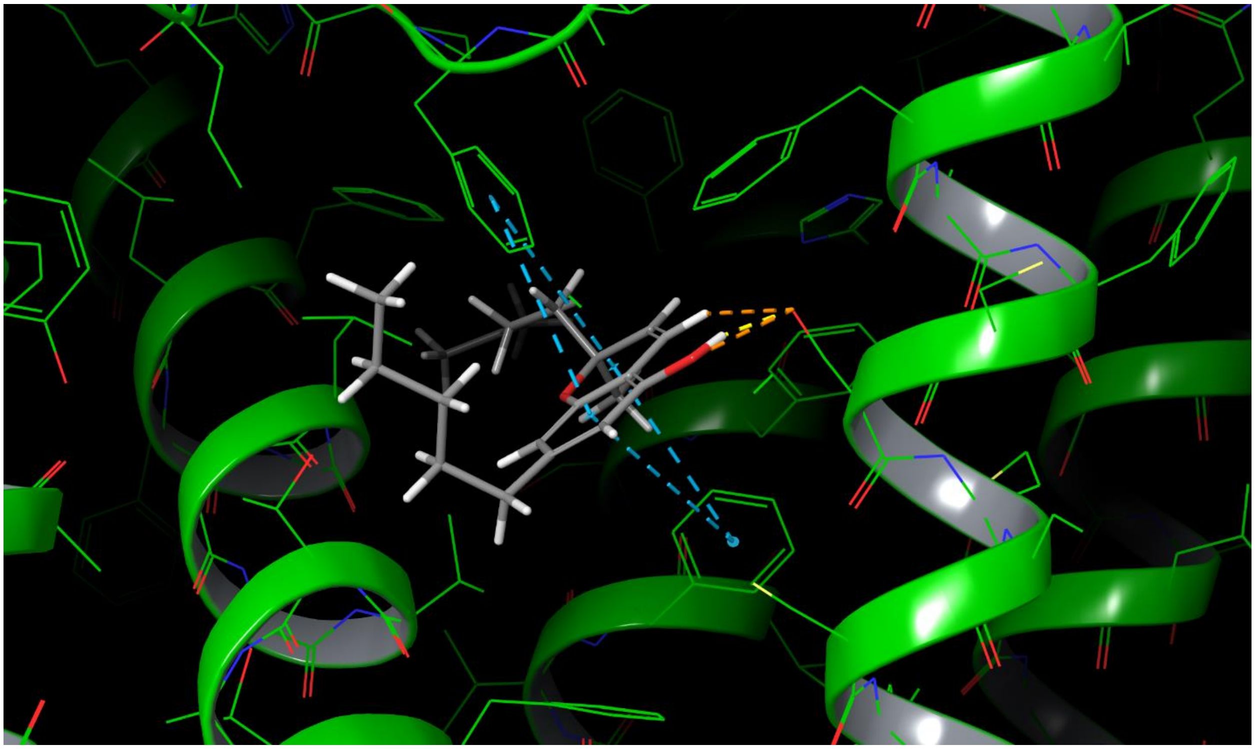

Figure S4.2.1. Three-dimensional rendering of (+)-CBC in the orthosteric site of the CB_1R utilizing SP modeling constraints. Blue dotted lines indicate π-π stacking, yellow dotted line indicates a hydrogen bond, and orange dotted lines indicate negative interactions.

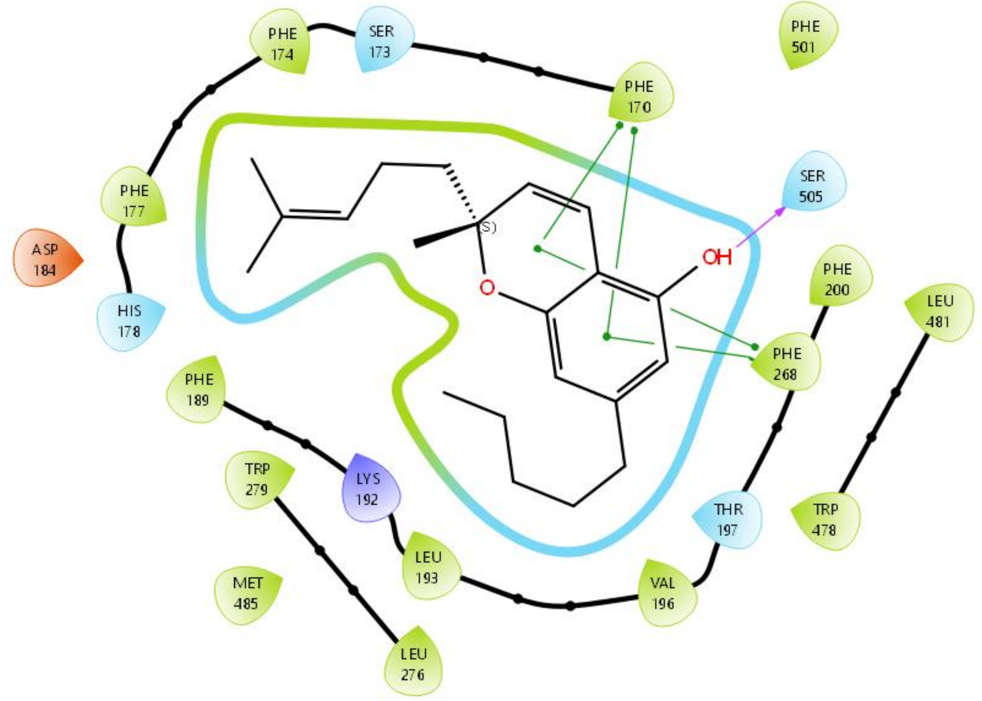

Figure S4.2.2. Two-dimensional rendering of (+)-CBC in the orthosteric site of the CB_1R utilizing SP modeling constraints. Green lines indicate π-π stacking and the pink arrow indicates a hydrogen bond.

Table S4.2.3. (-)-Δ^9-THC in the orthosteric site of the CB_1R: SP model.

Computer Ranking	Ligand	Docking Score	Glide emodel	Highlighted Residue-Ligand Interactions	Distance of Interaction (Å)	Type of Interaction
			**CB_1R SP model**			
2	(-)-Δ^9-THC	-10.569	-70.434	Ser505-OH	1.84	H-bond
				Phe268-AR	3.75	π-π stacking
				Phe170-AR	3.84	π-π stacking

Distances were measured based on the closest atom to atom distance between a given residue and the ligand. Abbreviations: Aromatic ring (AR), pyran-type ring (P), hydrogen bond (H-bond).

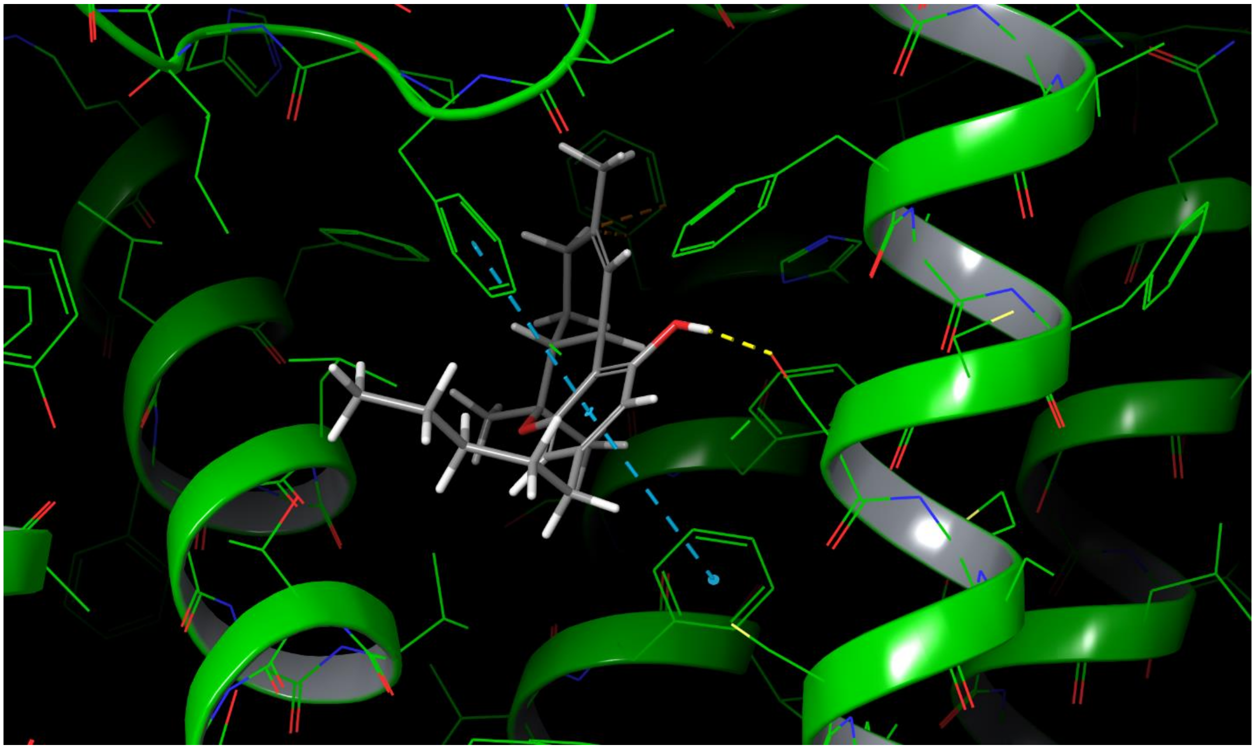

Figure S4.2.3. Three-dimensional rendering of (-)-Δ^9-THC in the orthosteric site of the CB_1R utilizing SP modeling constraints. Blue dotted lines indicate π-π stacking, yellow dotted line indicates a hydrogen bond, and orange dotted lines indicate negative interactions.

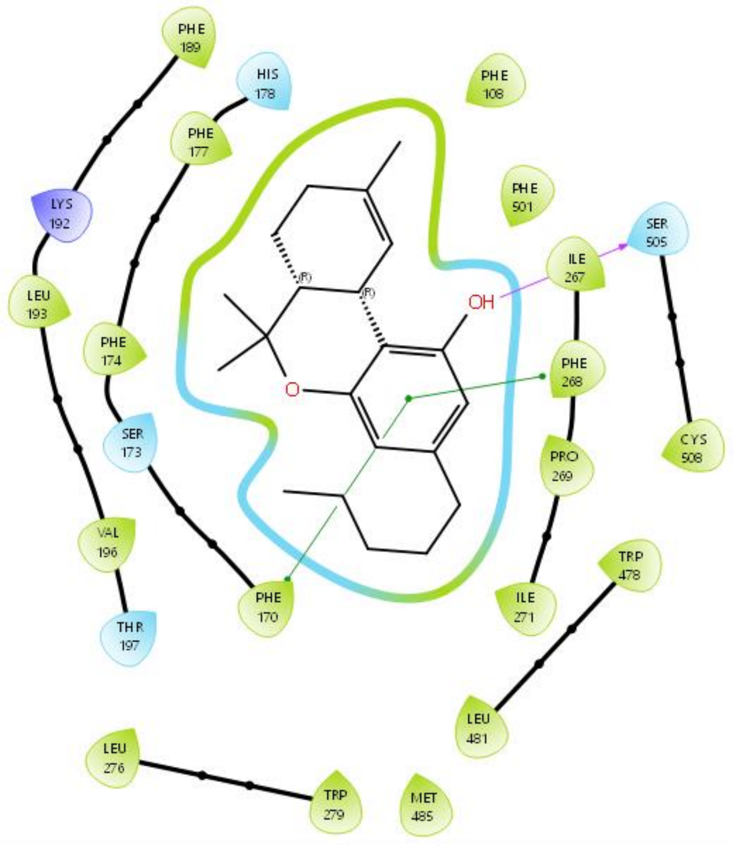

Figure S4.2.4. Two-dimensional rendering of (-)-Δ^9-THC in the orthosteric site of the CB_1R utilizing SP modeling constraints. Green lines indicate π-π stacking and the pink arrow indicates a hydrogen bond.

Table S4.2.4. (-)-CBC in the orthosteric site of the CB_1R: SP model.

				**CB_1R SP model**		
Computer Ranking	Ligand	Docking Score	Glide emodel	Highlighted Residue-Ligand Interactions	Distance of Interaction (Å)	Type of Interaction
3	(-)-CBC	-9.668	-63.781	Ser505-OH	1.81	H-bond
				Phe174-AR	3.33	π-π stacking
				Phe268-P	3.46	π-π stacking
				Phe268-AR	3.62	π-π stacking
				Phe170-AR	3.70	π-π stacking
				Phe170-P	3.70	π-π stacking

Distances were measured based on the closest atom to atom distance between a given residue and the ligand. Abbreviations: Aromatic ring (AR), pyran-type ring (P), hydrogen bond (H-bond).

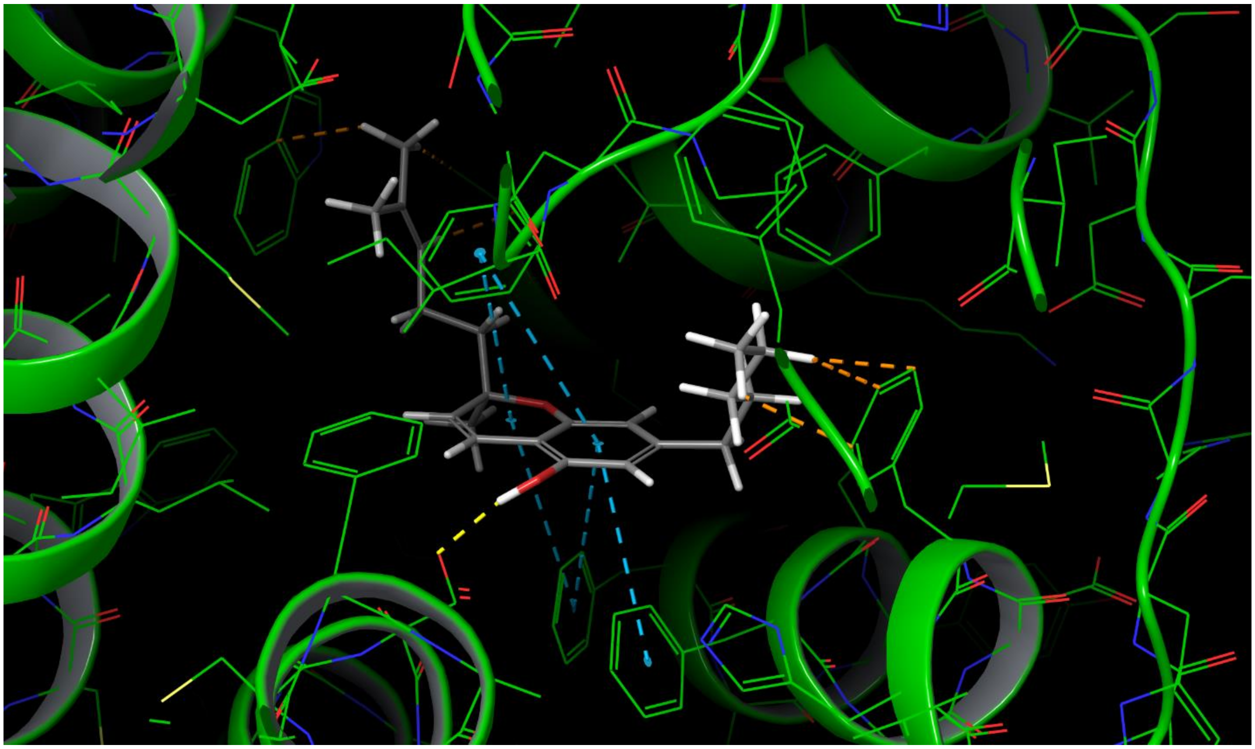

Figure S4.2.5. Three-dimensional rendering of (-)-CBC in the orthosteric site of the CB_1R utilizing SP modeling constraints. Blue dotted lines indicate π-π stacking, yellow dotted line indicates a hydrogen bond, and orange dotted lines indicate negative interactions.

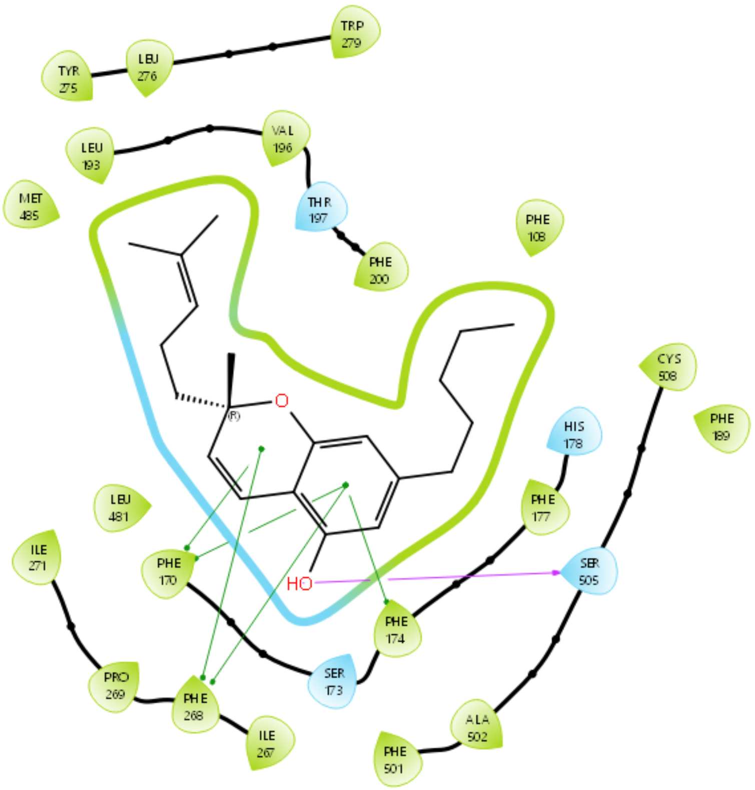

Figure S4.2.6. Two-dimensional rendering of (-)-CBC in the orthosteric site of the CB_1R utilizing SP modeling constraints. Green lines indicate π-π stacking and the pink arrow indicates a hydrogen bond.

Table S4.2.5. (R)-2’-hydroxy-(+)-cannabicitran in the orthosteric site of the CB_1R: SP model.

CB$_1$R SP model						
Computer Ranking	Ligand	Docking Score	Glide emodel	Highlighted Residue-Ligand Interactions	Distance of Interaction (Å)	Type of Interaction
4	(R)-2’-OH-(+)-cannabicitran	-9.254	-52.811	Ile267-OH	2.49	H-bond

Distances were measured based on the closest atom to atom distance between a given residue and the ligand. Abbreviations: hydrogen bond (H-bond).

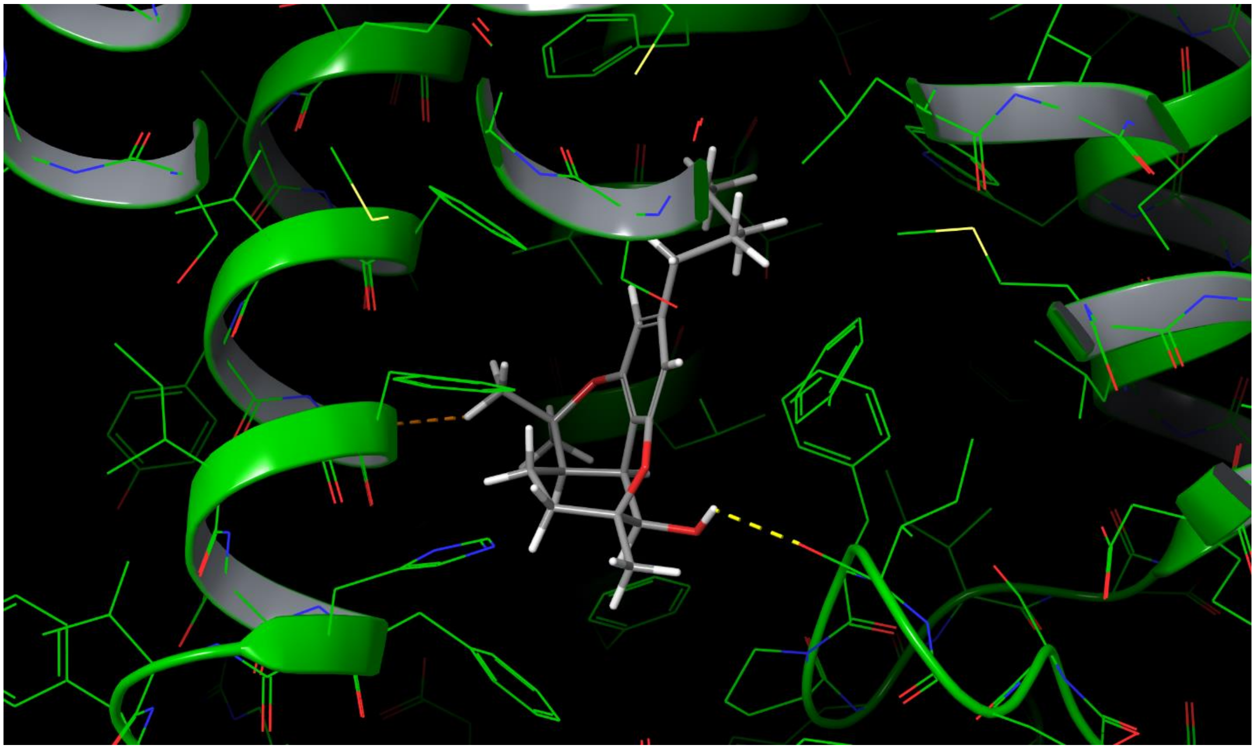

Figure S4.2.7. Three-dimensional rendering of (R)-2’-hydroxy-(+)-cannabicitran in the orthosteric site of the CB_1R utilizing SP modeling constraints. Yellow dotted line indicates a hydrogen bond and orange dotted lines indicate negative interactions.

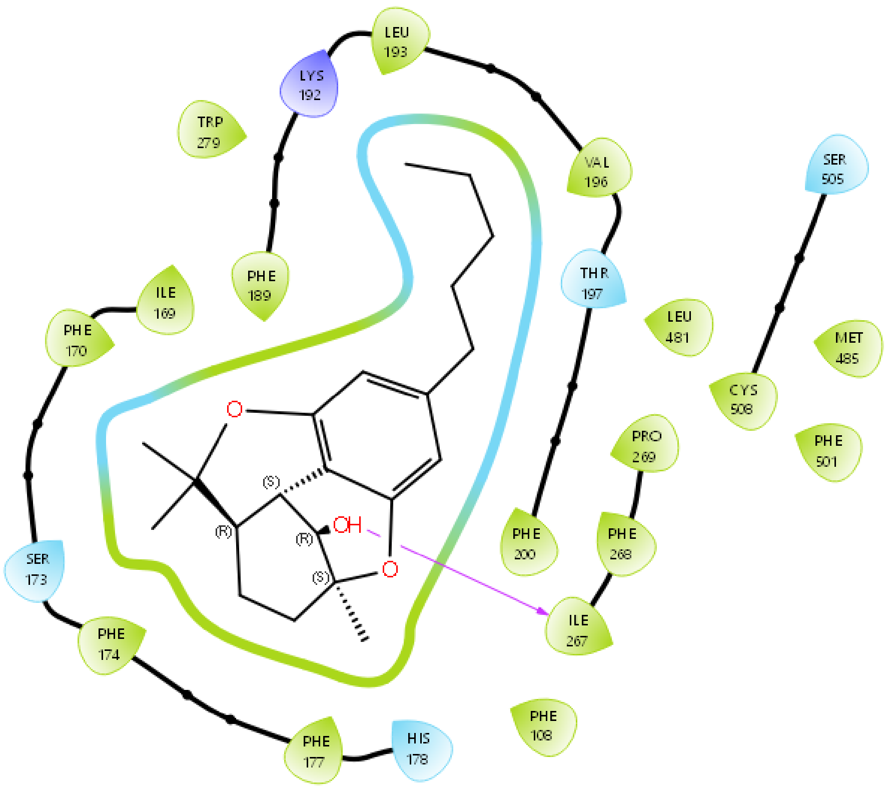

Figure S4.2.8. Two-dimensional rendering of (R)-2'-hydroxy-(+)-cannabicitran in the orthosteric site of the CB_1R utilizing SP modeling constraints. The pink arrow indicates a hydrogen bond.

Table S4.2.6. (+)-CBT-C in the orthosteric site of the CB_1R: SP model.

CB_1R SP model						
Computer Ranking	Ligand	Docking Score	Glide emodel	Highlighted Residue-Ligand Interactions	Distance of Interaction (Å)	Type of Interaction
5	(+)-CBT-C	-8.345	-35.785	Phe170-AR	3.38	π-π stacking

Distances were measured based on the closest atom to atom distance between a given residue and the ligand. Abbreviations: Aromatic ring (AR).

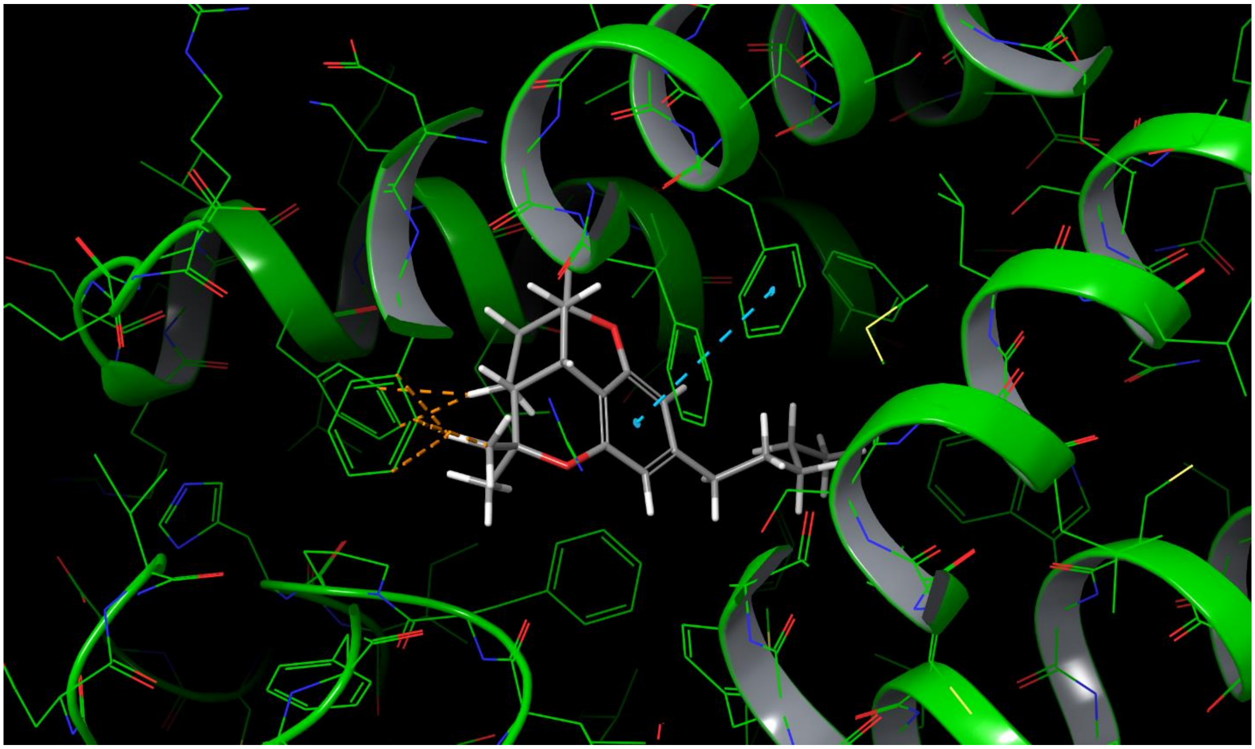

Figure S4.2.9. Three-dimensional rendering of (+)-CBT-C in the orthosteric site of the CB_1R utilizing SP modeling constraints. Blue dotted lines indicate π-π stacking and orange dotted lines indicate negative interactions.

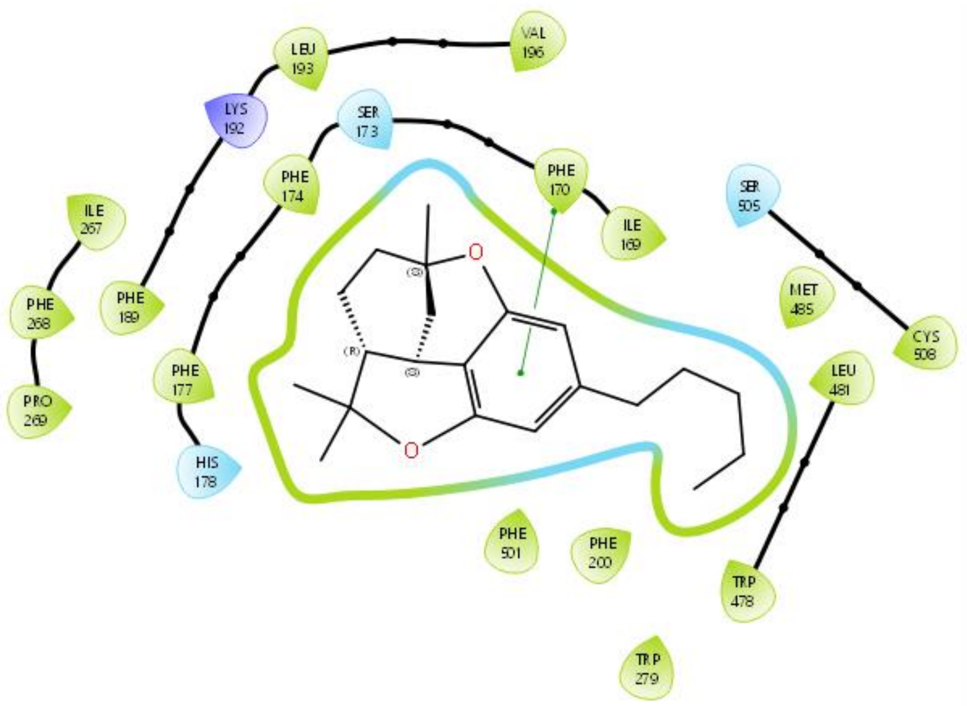

Figure S4.2.10. Two-dimensional rendering of (+)-CBT-C in the orthosteric site of the CB_1R utilizing SP modeling constraints. The green line indicates π-π stacking.

Table S4.2.7. (-)-CBT-C in the orthosteric site of the CB_1R: SP model.

				**CB_1R SP model**		
Computer Ranking	Ligand	Docking Score	Glide emodel	Highlighted Residue-Ligand Interactions	Distance of Interaction (Å)	Type of Interaction
6	(-)-CBT-C	-7.967	-38.936	Phe170-AR	3.55	π-π stacking
				Phe268-AR	3.55	π-π stacking

Distances were measured based on the closest atom to atom distance between a given residue and the ligand. Abbreviations: Aromatic ring (AR).

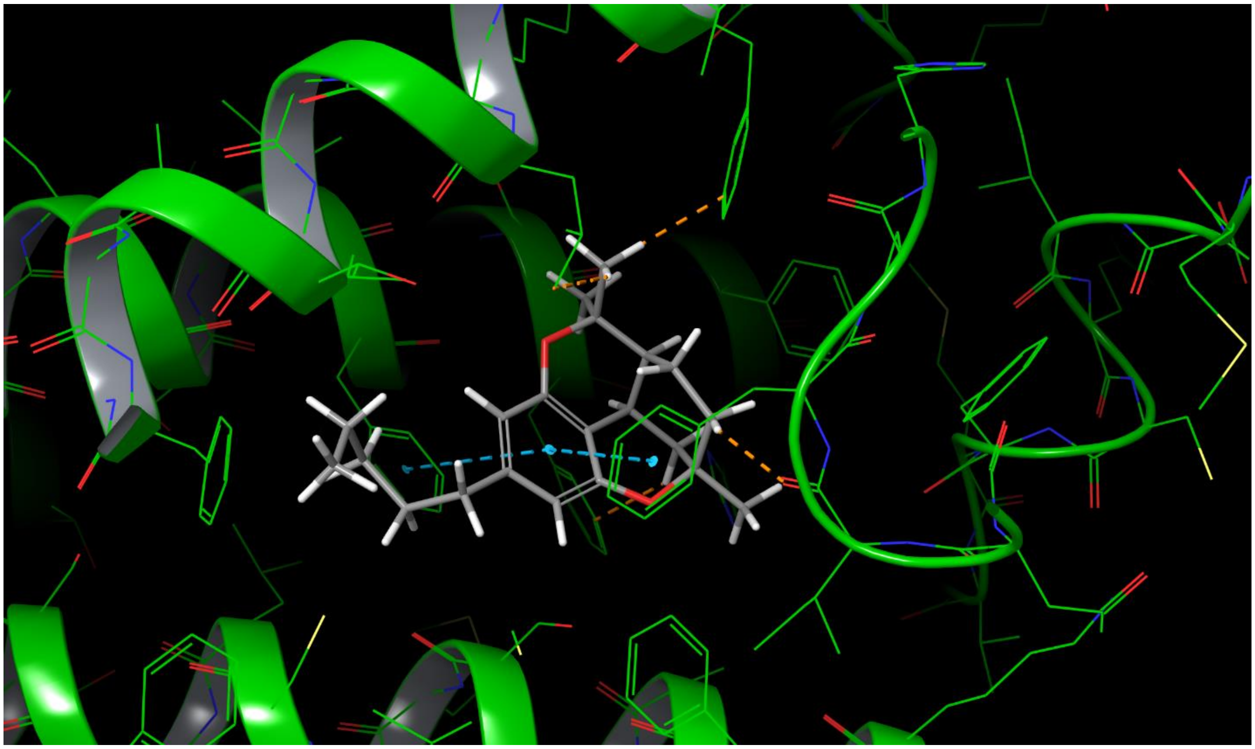

Figure S4.2.11. Three-dimensional rendering of (-)-CBT-C in the orthosteric site of the CB_1R utilizing SP modeling constraints. Blue dotted lines indicate π-π stacking and orange dotted lines indicate negative interactions.

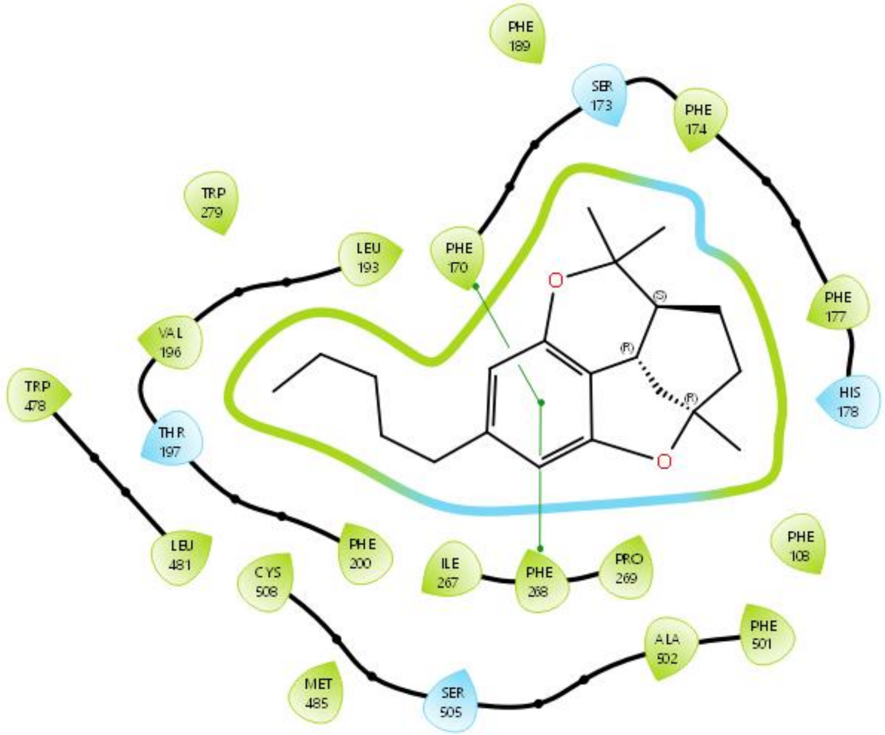

Figure S4.2.12. Two-dimensional rendering of (-)-CBT-C in the orthosteric site of the CB_1R utilizing SP modeling constraints. The green lines indicate π-π stacking.

Table S4.2.8. (S)-2'-hydroxy-(-)-cannabicitran in the orthosteric site of the CB_1R: SP model.

		**CB_1R SP model**				
Computer Ranking	Ligand	Docking Score	Glide emodel	Highlighted Residue-Ligand Interactions	Distance of Interaction (Å)	Type of Interaction
7	(S)-2'-OH-(-)-cannabicitran	-6.796	-27.572	Phe170-AR	3.23	π-π stacking

Distances were measured based on the closest atom to atom distance between a given residue and the ligand. Abbreviations: Aromatic ring (AR).

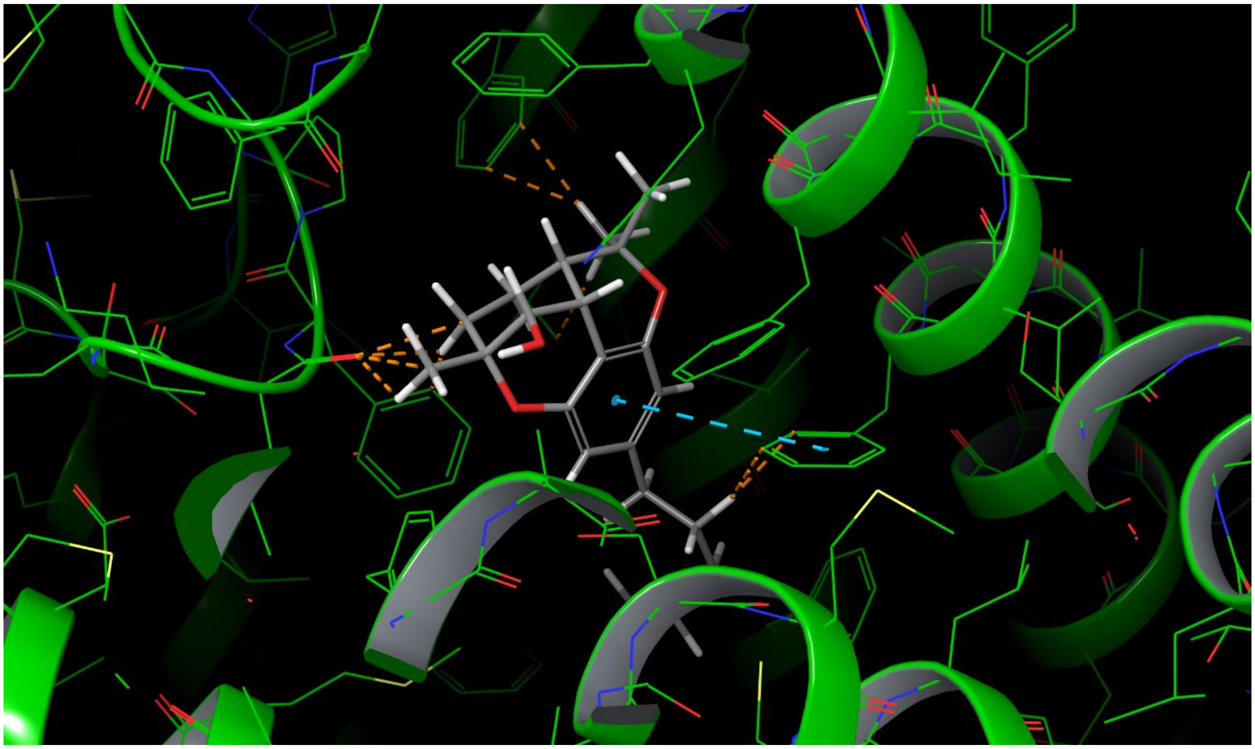

Figure S4.2.13. Three-dimensional rendering of (S)-2'-hydroxy-(-)-cannabicitran in the orthosteric site of the CB_1R utilizing SP modeling constraints. Blue dotted lines indicate π-π stacking and orange dotted lines indicate negative interactions.

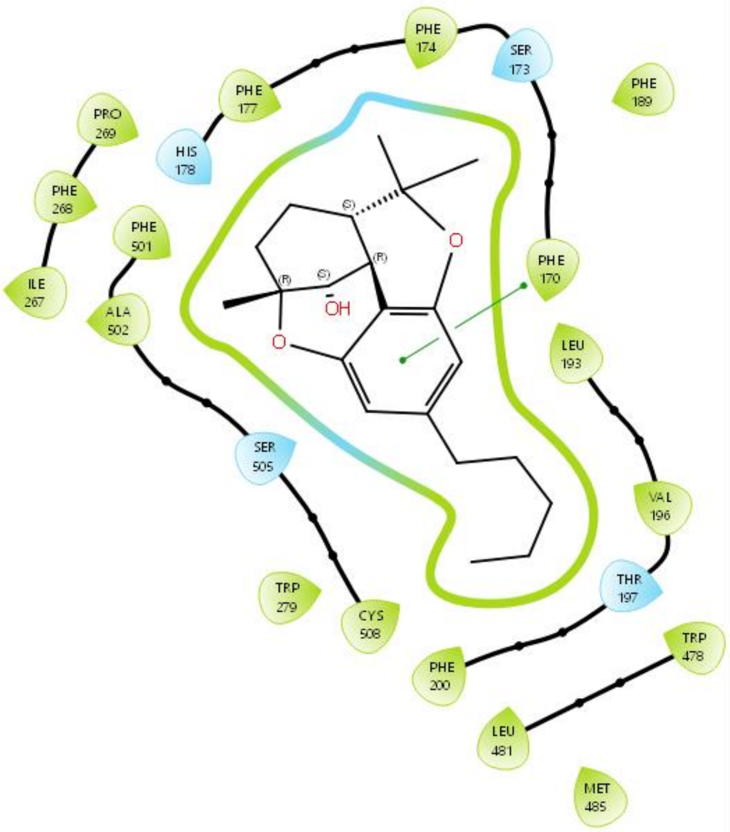

Figure S4.2.14. Two-dimensional rendering of (S)-2'-hydroxy-(-)-cannabicitran in the orthosteric site of the CB_1R utilizing SP modeling constraints. The green line indicates π-π stacking.

Table S4.2.9. Ligand ranking and interactions in the orthosteric site of the CB_1R: XP model.

CB_1R XP model						
Computer Ranking	Ligand	Docking Score	Glide emodel	Highlighted Residue-Ligand Interactions	Distance of Interaction (Å)	Type of Interaction
1	(-)-CBC	-11.924	-62.738	Ser505-OH	1.65	H-bond
				Phe170-AR	3.70	π-π stacking
				Phe268-AR	3.70	π-π stacking
				Phe268-P	3.81	π-π stacking
2	(+)-CBC	-11.681	-67.236	Ser505-OH	1.75	H-bond
				Phe170-P	3.47	π-π stacking
				Phe268-AR	3.66	π-π stacking
				Phe268-P	3.66	π-π stacking
3	(-)-Δ^9-THC	-11.457	-70.266	Ser505-OH	1.81	H-bond
				Phe268-AR	3.73	π-π stacking
				Phe170-AR	3.81	π-π stacking
4	(R)-2'-OH-(+)-cannabicitran	-10.687	-41.065	Ile267-OH	1.99	H-bond

Distances were measured based on the closest atom to atom distance between a given residue and the ligand. Abbreviations: Aromatic ring (AR), pyran-type ring (P), hydrogen bond (H-bond).

Table S4.2.10. (-)-CBC in the orthosteric site of the CB_1R: XP model.

		CB_1R XP model				
Computer Ranking	Ligand	Docking Score	Glide emodel	Highlighted Residue-Ligand Interactions	Distance of Interaction (Å)	Type of Interaction
1	(-)-CBC	-11.924	-62.738	Ser505-OH	1.65	H-bond
				Phe170-AR	3.70	π-π stacking
				Phe268-AR	3.70	π-π stacking
				Phe268-P	3.81	π-π stacking

Distances were measured based on the closest atom to atom distance between a given residue and the ligand. Abbreviations: Aromatic ring (AR), pyran-type ring (P), hydrogen bond (H-bond).

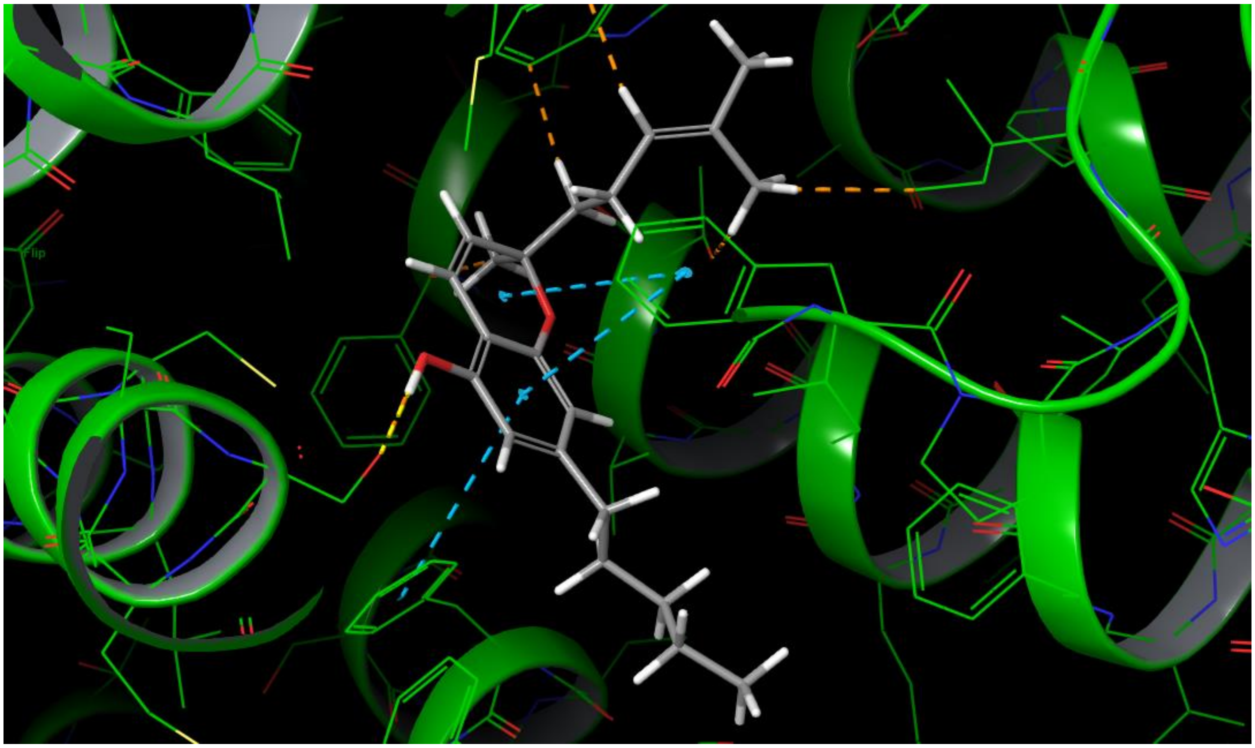

Figure S4.2.15. Three-dimensional rendering of (-)-CBC in the orthosteric site of the CB_1R utilizing XP modeling constraints. Blue dotted lines indicate π-π stacking, the yellow dotted line indicates a hydrogen bond, and orange dotted lines indicate negative interactions.

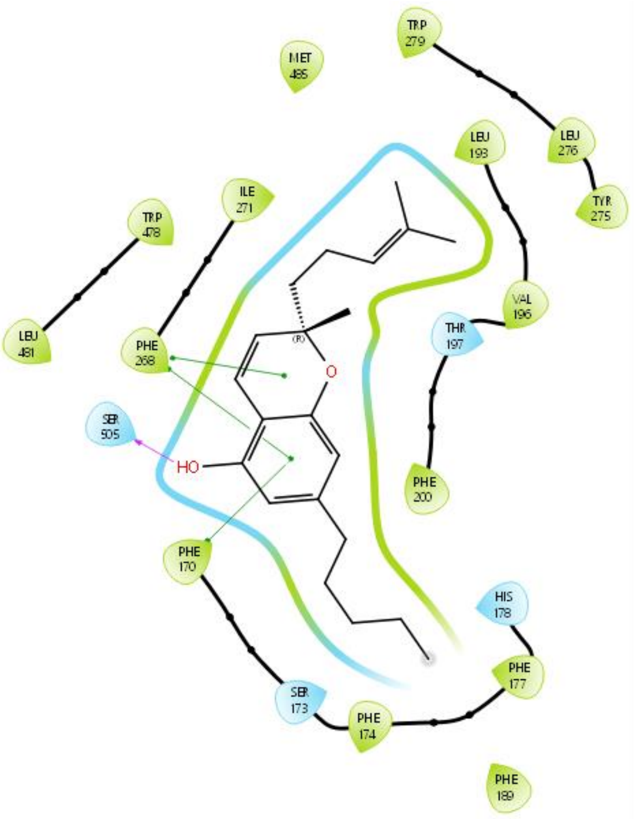

Figure S4.2.16. Two-dimensional rendering of (-)-CBC in the orthosteric site of the CB_1R utilizing XP modeling constraints. The green line indicates π-π stacking and the pink arrow indicates a hydrogen bond.

Table S4.2.11. (+)-CBC in the orthosteric site of the CB_1R: XP model.

		**CB_1R XP model**				
Computer Ranking	Ligand	Docking Score	Glide emodel	Highlighted Residue-Ligand Interactions	Distance of Interaction (Å)	Type of Interaction
2	(+)-CBC	-11.681	-67.236	Ser505-OH	1.75	H-bond
				Phe170-P	3.47	π-π stacking
				Phe268-AR	3.66	π-π stacking
				Phe268-P	3.66	π-π stacking

Distances were measured based on the closest atom to atom distance between a given residue and the ligand. Abbreviations: Aromatic ring (AR), pyran-type ring (P), hydrogen bond (H-bond).

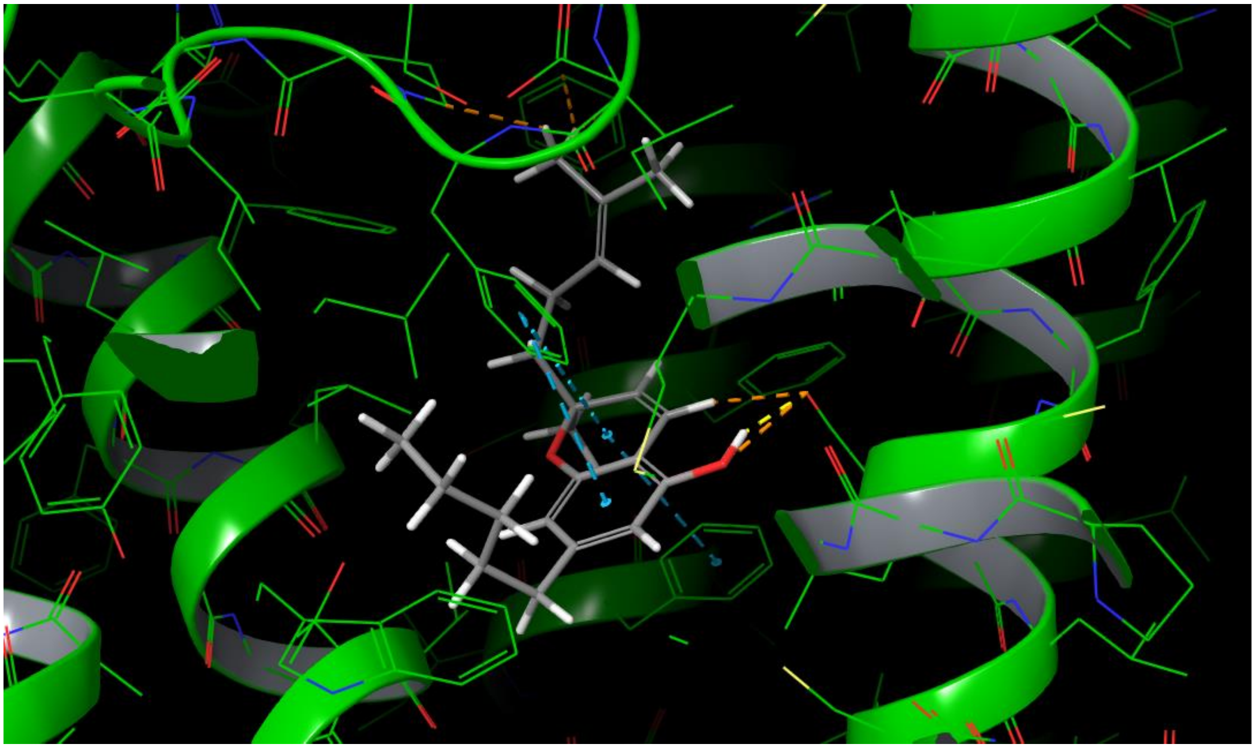

Figure S4.2.17. Three-dimensional rendering of (+)-CBC in the orthosteric site of the CB_1R utilizing XP modeling constraints. Blue dotted lines indicate π-π stacking, the yellow dotted line indicates a hydrogen bond, and orange dotted lines indicate negative interactions.

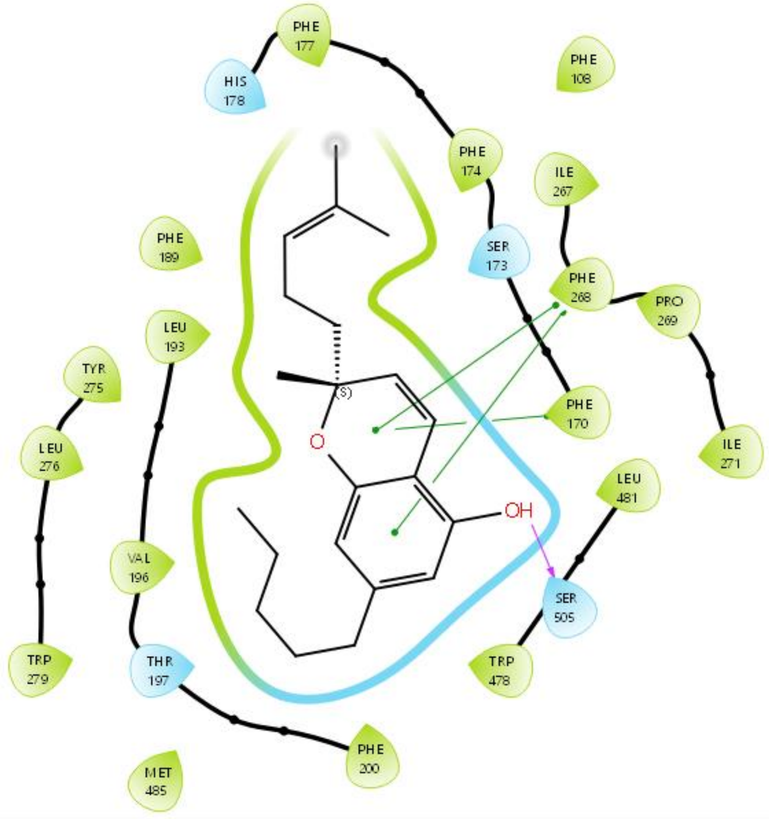

Figure S4.2.18. Two-dimensional rendering of (+)-CBC in the orthosteric site of the CB_1R utilizing XP modeling constraints. The green line indicates π-π stacking and the pink arrow indicates a hydrogen bond.

Table S4.2.12. (-)-Δ[9]-THC in the orthosteric site of the CB_1R: XP model.

		**CB_1R XP model**				
Computer Ranking	Ligand	Docking Score	Glide emodel	Highlighted Residue-Ligand Interactions	Distance of Interaction (Å)	Type of Interaction
3	(-)-Δ[9]-THC	-11.457	-70.266	Ser505-OH	1.81	H-bond
				Phe268-AR	3.73	π-π stacking
				Phe170-AR	3.81	π-π stacking

Distances were measured based on the closest atom to atom distance between a given residue and the ligand. Abbreviations: Aromatic ring (AR) and hydrogen bond (H-bond).

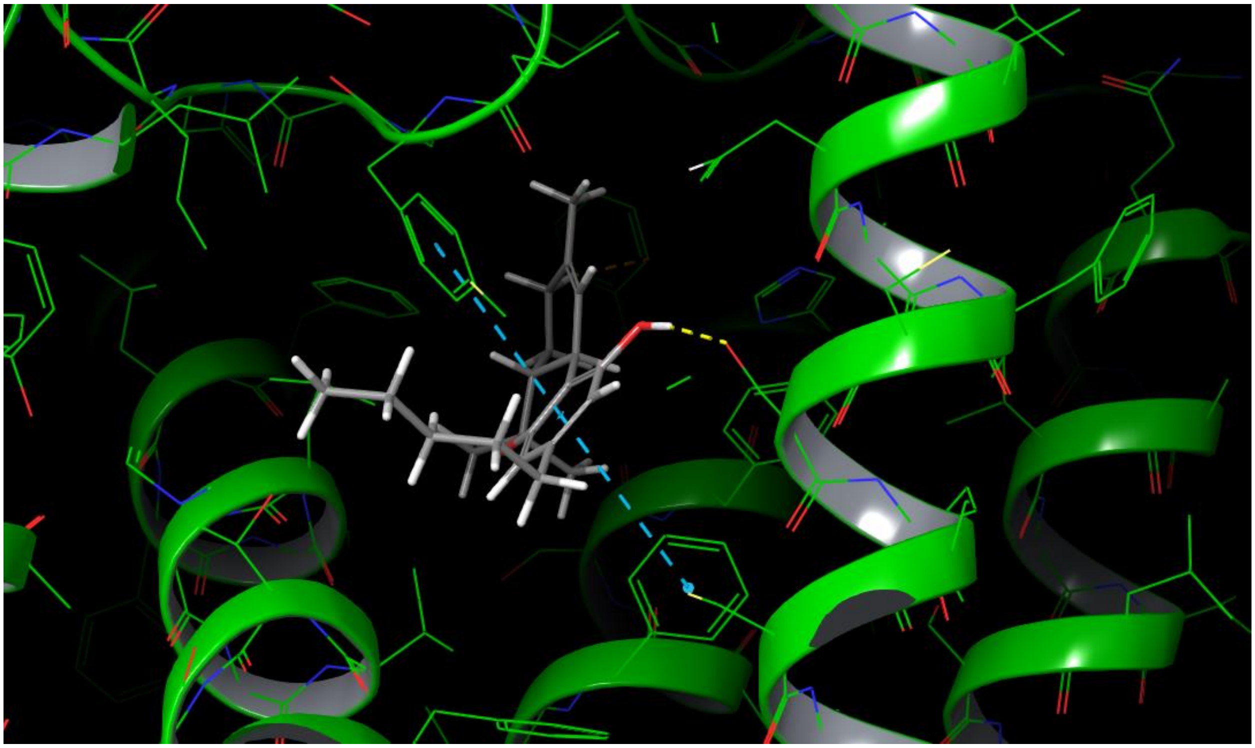

Figure S4.2.19. Three-dimensional rendering of (-)-Δ[9]-THC in the orthosteric site of the CB_1R utilizing XP modeling constraints. Blue dotted lines indicate π-π stacking and the yellow dotted line indicates a hydrogen bond.

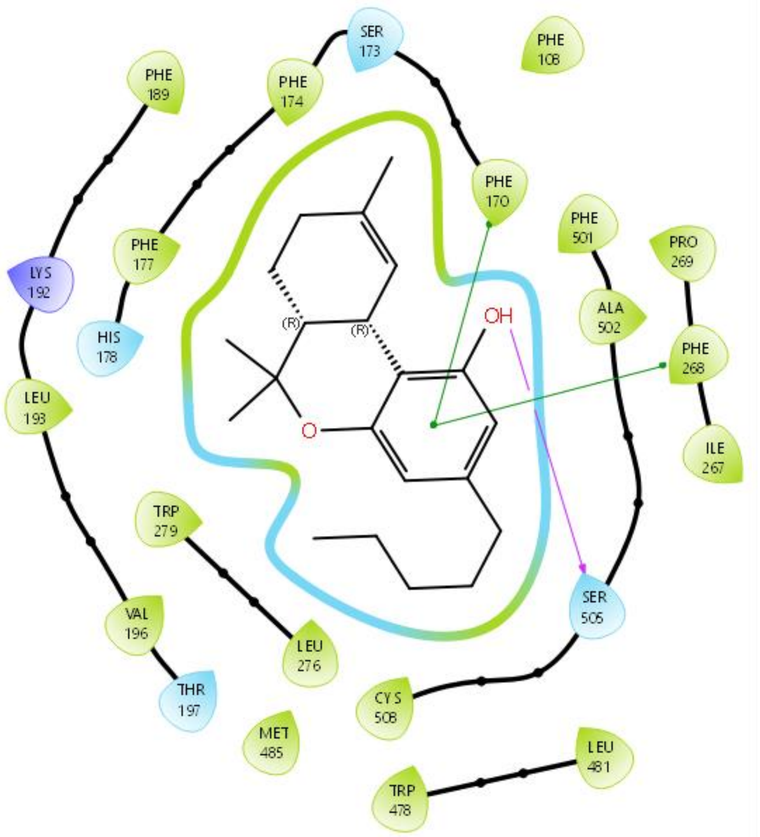

Figure S4.2.20. Two-dimensional rendering of (-)-Δ^9-THC in the orthosteric site of the CB_1R utilizing XP modeling constraints. The green line indicates π-π stacking and the pink arrow indicates a hydrogen bond.

Table S4.2.13. (R)-2'-hydroxy-(+)-cannabicitran in the orthosteric site of the CB_1R: XP model.

CB₁R XP model						
Computer Ranking	Ligand	Docking Score	Glide emodel	Highlighted Residue-Ligand Interactions	Distance of Interaction (Å)	Type of Interaction
4	(R)-2'-OH-(+)-cannabicitran	-10.687	-41.065	Ile267-OH	1.99	H-bond

Distances were measured based on the closest atom to atom distance between a given residue and the ligand. Abbreviations: hydrogen bond (H-bond).

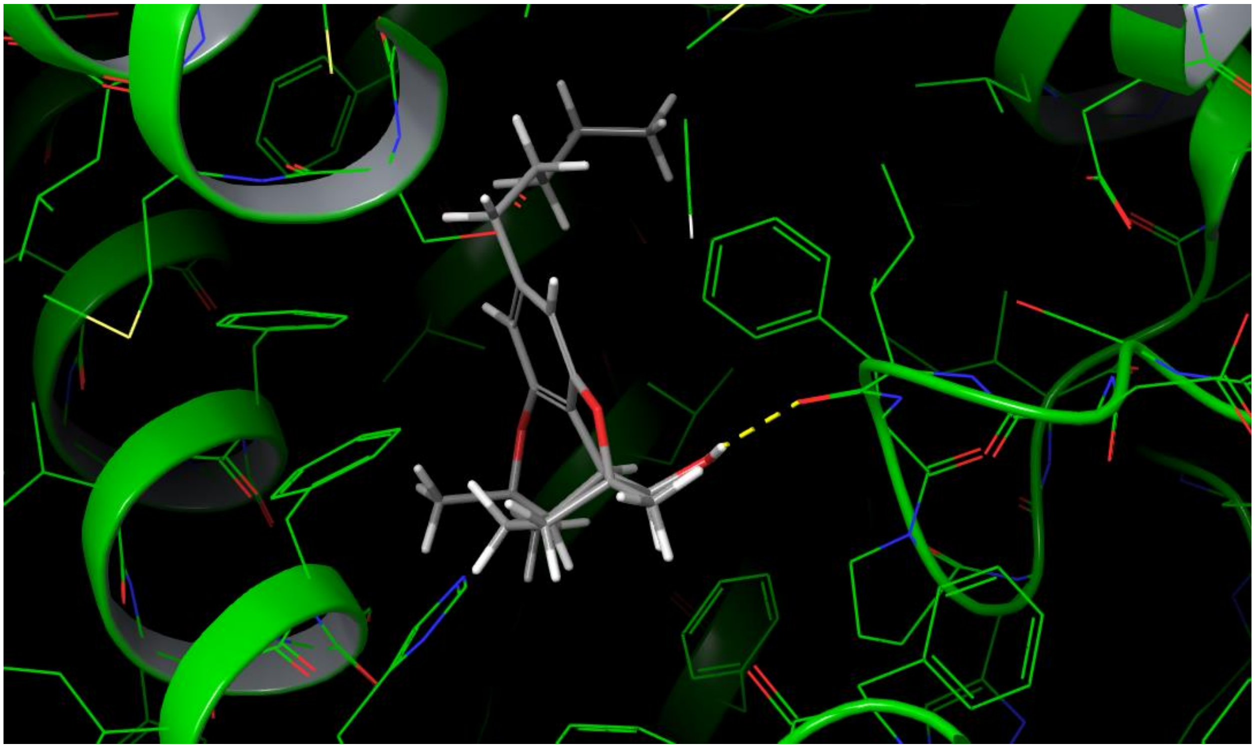

Figure S4.2.21. Three-dimensional rendering of (R)-2'-hydroxy-(+)-cannabicitran in the orthosteric site of the CB_1R utilizing XP modeling constraints. The yellow dotted line indicates a hydrogen bond.

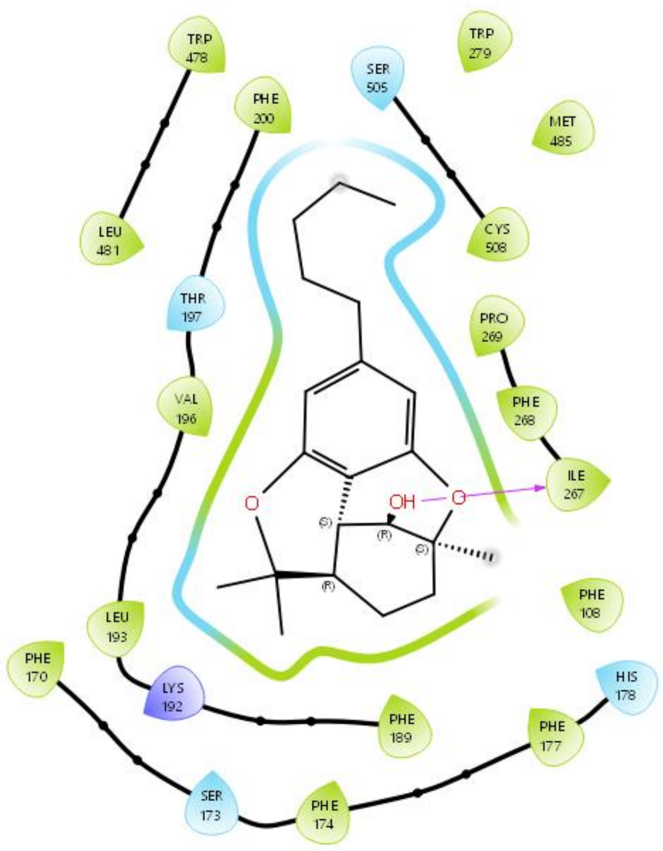

Figure S4.2.22. Two-dimensional rendering of (R)-2’-hydroxy-(+)-cannabicitran in the orthosteric site of the CB_1R utilizing XP modeling constraints. The pink arrow indicates a hydrogen bond.

S4.3

Cannabinoid 2 Receptor Molecular Docking

Structures were docked into the orthosteric site of CB_2R. Both Standard-Precision (SP) and Extra-Precision (XP) glide models were used to rank the test ligands.

Predicted interactions could be used to determine if there is potential for binding of 2'-hydroxycannabicitran at the cannabinoid receptors. The results of this experiment would inform if *in vitro* testing would be valuable to move forward with.

Please refer to the main manuscript text for a discussion of the molecular docking results.

Please see supplementary Table 4.3.1 for an SP model of ligands and interactions ranked in the orthosteric site of CB_2R. Tables 4.3.2-4.3.8 highlight each tested ligand and Figures 4.3.1-4.3.14 display two- and three- dimensional figures of the ligands docked into the active site.

Please see supplementary Table 4.3.9 for an XP model of ligands and interactions ranked in the orthosteric site of CB_2R. Tables 4.3.10 and 4.3.11 highlight each tested ligand and Figures 4.3.15-4.3.18 display two- and three- dimensional figures of the ligands docked into the active site.

Methods

Schrödinger Suite (release 2023-3, Schrödinger, New York, NY, USA) was used for all ligand and protein preparation, and the Glide Module for all docking calculations [39-41]. Using LigPrep, the following compounds were prepared at physiological pH: (-)-Δ9-THC, (+)-CBC, (-)-CBC, (R)-2'-hydroxy-(+)-cannabicitran, (S)-2'-hydroxy-(-)-cannabicitran, (+)-CBT-C, and (-)-CBT-C (structures are shown in Figure S4.1.1.). CB_2R was imported from Protein Data Bank (PDB code: 5ZTY) [43]. These protein structures were prepared by removing all water molecules and co-crystallized ligands, and then minimized with OPLS4 force fields and the VSGB solvation model [44]. CB_2R were scanned and mapped to confirm their binding site. Computational grids were then formed around the mapped binding sites to have the test compounds docked into. This docking was completed with Standard-Precision (SP) and Extra-Precision (XP) glide models for comparison. Top-ranked ligand-protein conformations were produced with corresponding scoring function values.

Table S4.3.1. Ligand ranking and interactions in the orthosteric site of the CB_2R: SP model.

CB_2R SP model						
Computer Ranking	Ligand	Docking Score	Glide emodel	Highlighted Residue-Ligand Interactions	Distance of Interaction (Å)	Type of Interaction
1	(+)-CBC	-8.545	-55.068	Phe87-P	3.81	π-π stacking
				Phe183-P	3.99	π-π stacking
2	(+)-CBT-C	-8.472	-49.049	-	-	-
3	(-)-Δ^9-THC	-8.312	-52.083	Phe87-AR	3.68	π-π stacking
				Phe183-AR	3.99	π-π stacking
4	(S)-2'-OH-(-)-cannabicitran	-8.042	-37.567	-	-	-
5	(-)-CBT-C	-7.949	-39.069	Phe183-AR	3.28	π-π stacking
6	(-)-CBC	-7.892	-51.054	Phe87-P	3.57	π-π stacking
				Phe87-AR	3.57	π-π stacking
				Phe183-P	4.17	π-π stacking
7	(R)-2'-OH-(+)-cannabicitran	-7.81	-44.543	Ser90-OH	2.67	H-bond
				Phe183-AR	3.63	π-π stacking
				Phe87-AR	3.91	π-π stacking

Distances were measured based on the closest atom to atom distance between a given residue and the ligand. Abbreviations: Aromatic ring (AR), pyran-type ring (P), hydrogen bond (H-bond).

Table S4.3.2. (+)-CBC in the orthosteric site of the CB_2R: SP model.

		**CB_2R SP model**				
Computer Ranking	Ligand	Docking Score	Glide emodel	Highlighted Residue-Ligand Interactions	Distance of Interaction (Å)	Type of Interaction
1	(+)-CBC	-8.545	-55.068	Phe87-P	3.81	π-π stacking
				Phe183-P	3.99	π-π stacking

Distances were measured based on the closest atom to atom distance between a given residue and the ligand. Abbreviations: pyran-type ring (P).

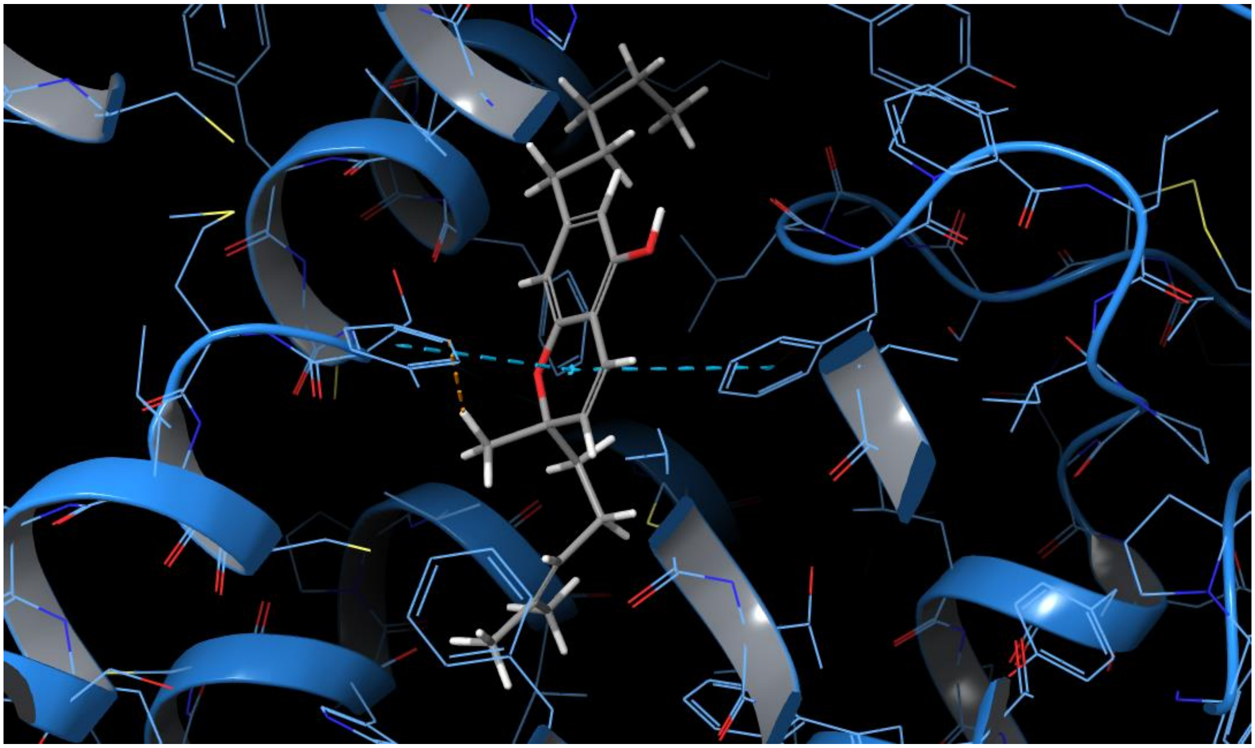

Figure S4.3.1. Three-dimensional rendering of (+)-CBC in the orthosteric site of the CB_2R utilizing SP modeling constraints. Blue dotted lines indicate π-π stacking and orange dotted lines indicate negative interactions.

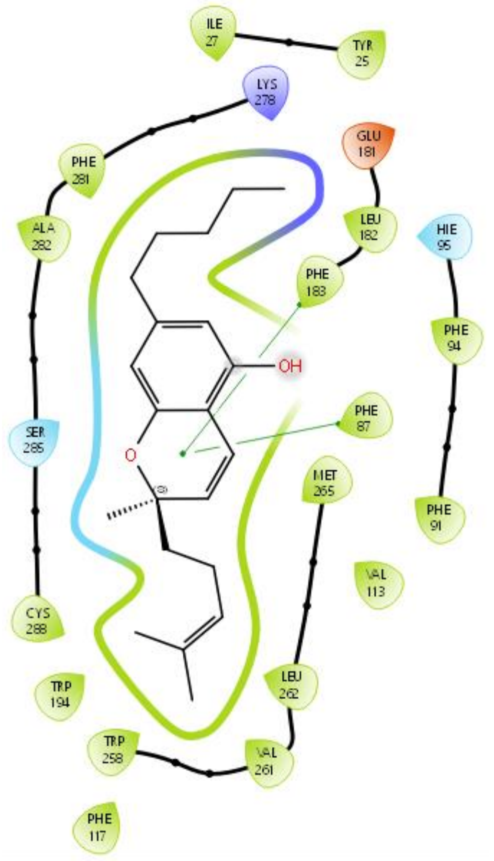

Figure S4.3.2. Two-dimensional rendering of (+)-CBC in the orthosteric site of the CB_2R utilizing SP modeling constraints. Green lines indicate π-π stacking.

Table S4.3.3. (+)-CBT-C in the orthosteric site of the CB_2R: SP model.

			**CB_2R SP model**			
Computer Ranking	Ligand	Docking Score	Glide emodel	Highlighted Residue-Ligand Interactions	Distance of Interaction (Å)	Type of Interaction
2	(+)-CBT-C	-8.472	-49.049	-	-	-

Distances were measured based on the closest atom to atom distance between a given residue and the ligand.

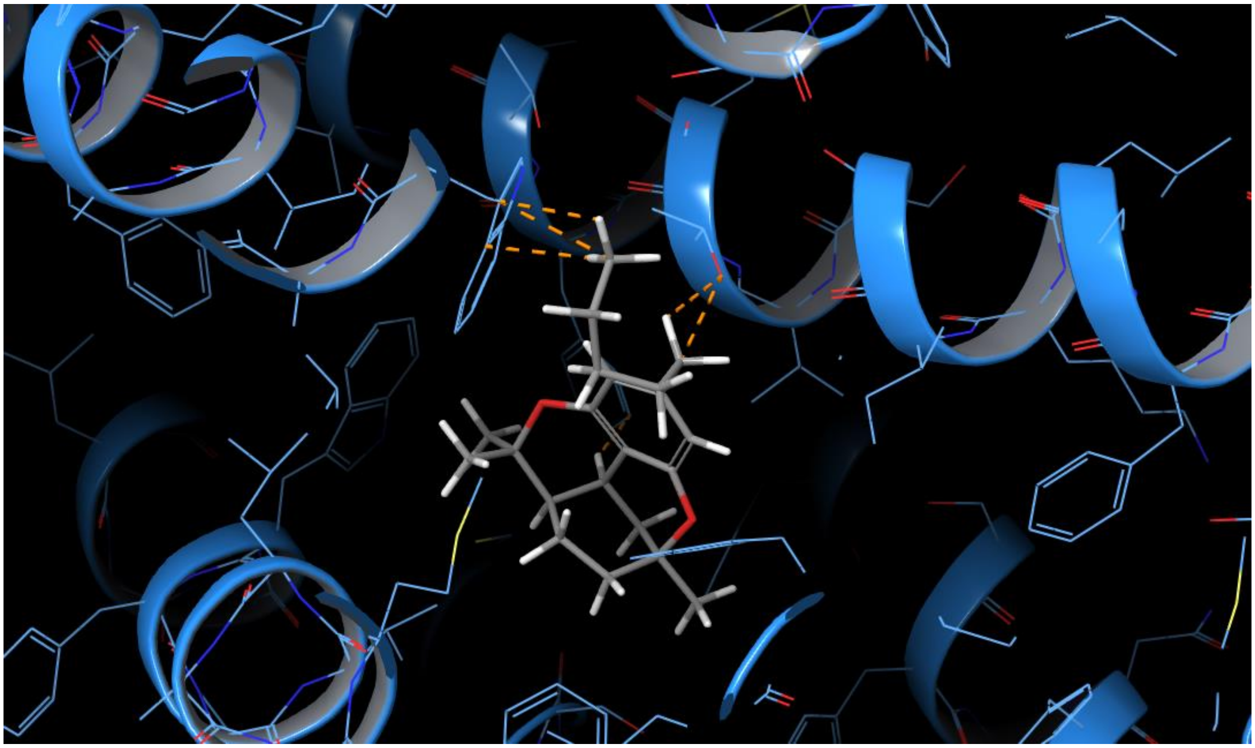

Figure S4.3.3. Three-dimensional rendering of (+)-CBT-C in the orthosteric site of the CB_2R utilizing SP modeling constraints. Orange dotted lines indicate negative interactions.

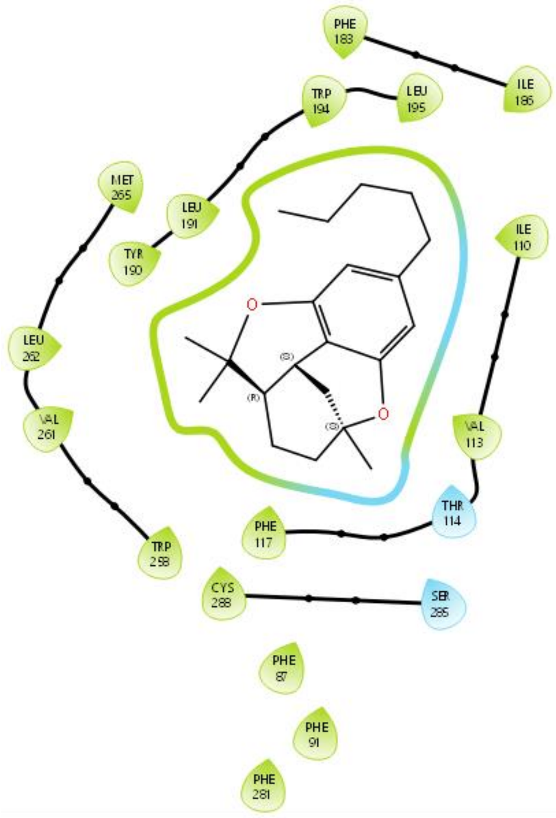

Figure S4.3.4. Two-dimensional rendering of (+)-CBT-C in the orthosteric site of the CB_2R utilizing SP modeling constraints.

Table S4.3.4. (-)-Δ^9-THC in the orthosteric site of the CB_2R: SP model.

CB₂R SP model						
Computer Ranking	Ligand	Docking Score	Glide emodel	Highlighted Residue-Ligand Interactions	Distance of Interaction (Å)	Type of Interaction
3	(-)-Δ^9-THC	-8.312	-52.083	Phe87-AR	3.68	π-π stacking
				Phe183-AR	3.99	π-π stacking

Distances were measured based on the closest atom to atom distance between a given residue and the ligand. Abbreviations: Aromatic ring (AR).

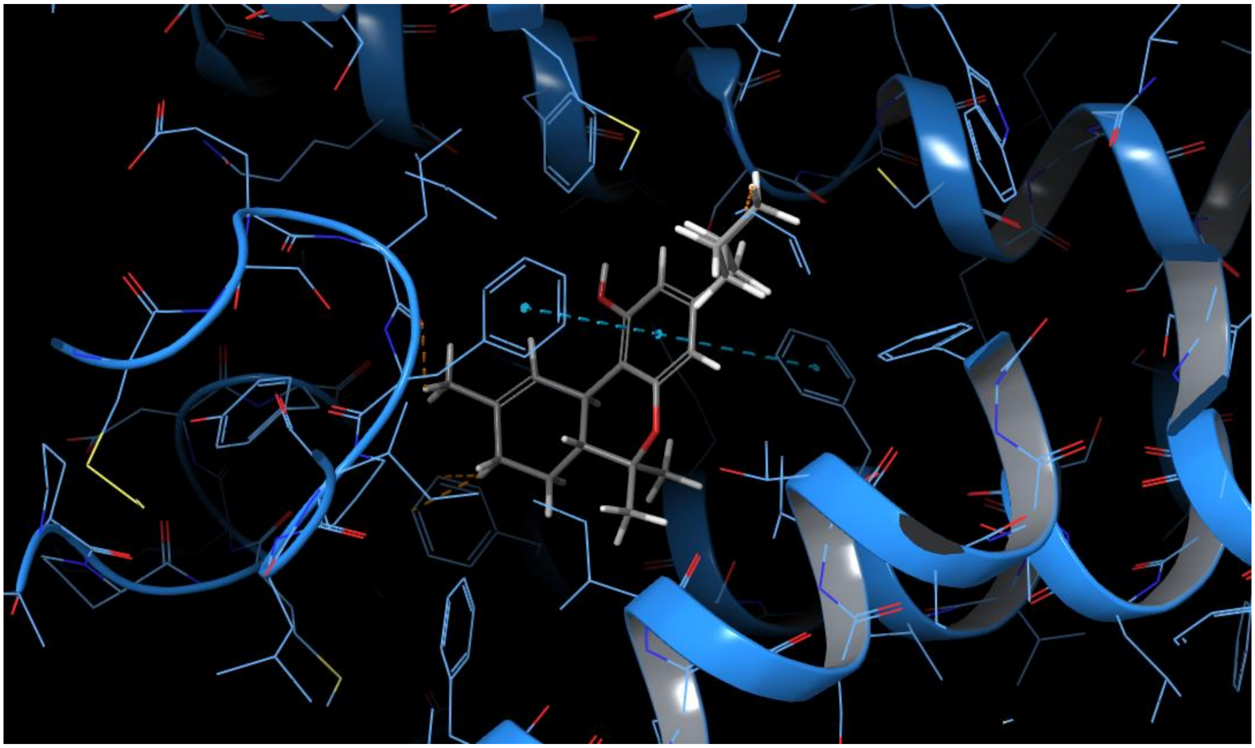

Figure S4.3.5. Three-dimensional rendering of (-)-Δ^9-THC in the orthosteric site of the CB_2R utilizing SP modeling constraints. Blue dotted lines indicate π-π stacking and orange dotted lines indicate negative interactions.

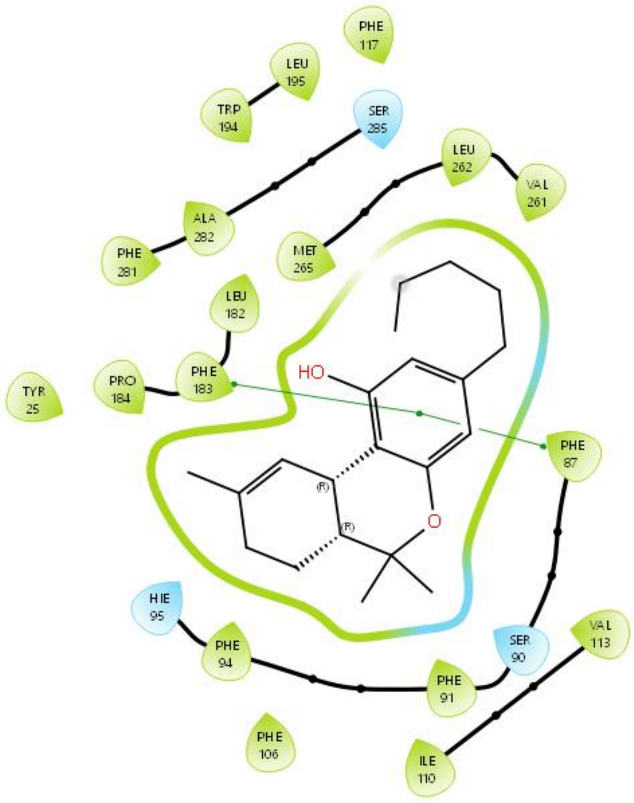

Figure S4.3.6. Two-dimensional rendering of (-)-Δ[9]-THC in the orthosteric site of the CB_2R utilizing SP modeling constraints. Green lines indicate π-π stacking.

Table S4.3.5. (S)-2’-hydroxy-(-)-cannabicitran in the orthosteric site of the CB_2R: SP model.

		**CB_2R SP model**				
Computer Ranking	Ligand	Docking Score	Glide emodel	Highlighted Residue-Ligand Interactions	Distance of Interaction (Å)	Type of Interaction
4	(S)-2'-OH-(-)-cannabicitran	-8.042	-37.567	-	-	-

Distances were measured based on the closest atom to atom distance between a given residue and the ligand.

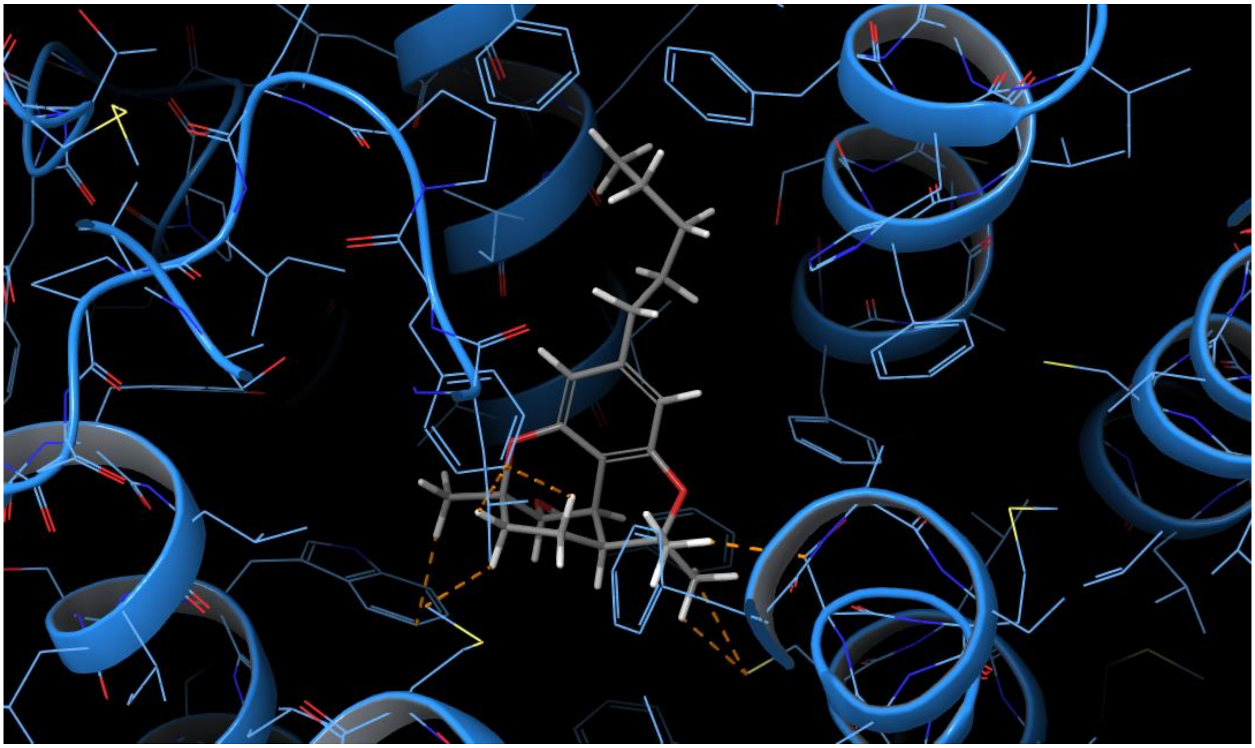

Figure S4.3.7. Three-dimensional rendering of (S)-2’-hydroxy-(-)-cannabicitran in the orthosteric site of the CB_2R utilizing SP modeling constraints. Orange dotted lines indicate negative interactions.

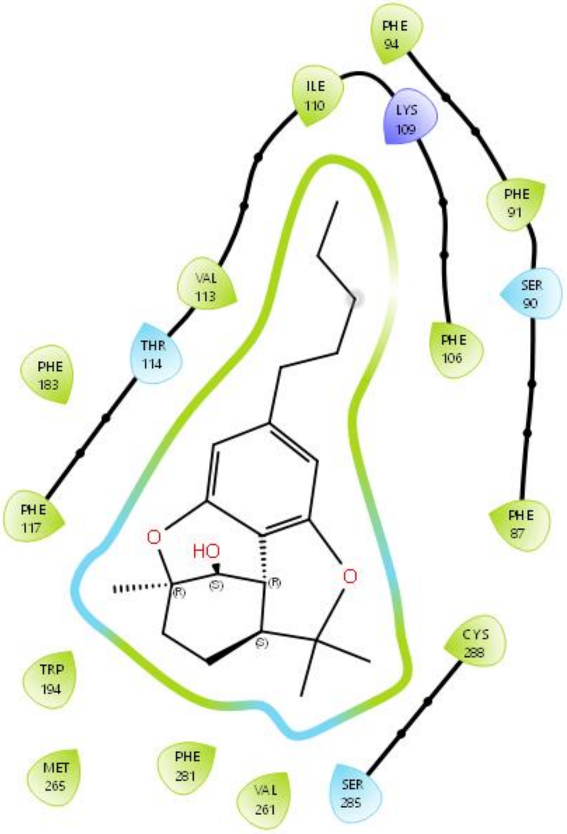

Figure S4.3.8. Two-dimensional rendering of (S)-2'-hydroxy-(-)-cannabicitran in the orthosteric site of the CB_2R utilizing SP modeling constraints.

Table S4.3.6. (-)-CBT-C in the orthosteric site of the CB_2R: SP model.

CB₂R SP model						
Computer Ranking	Ligand	Docking Score	Glide emodel	Highlighted Residue-Ligand Interactions	Distance of Interaction (Å)	Type of Interaction
5	(-)-CBT-C	-7.949	-39.069	Phe183-AR	3.28	π-π stacking

Distances were measured based on the closest atom to atom distance between a given residue and the ligand. Abbreviations: Aromatic ring (AR).

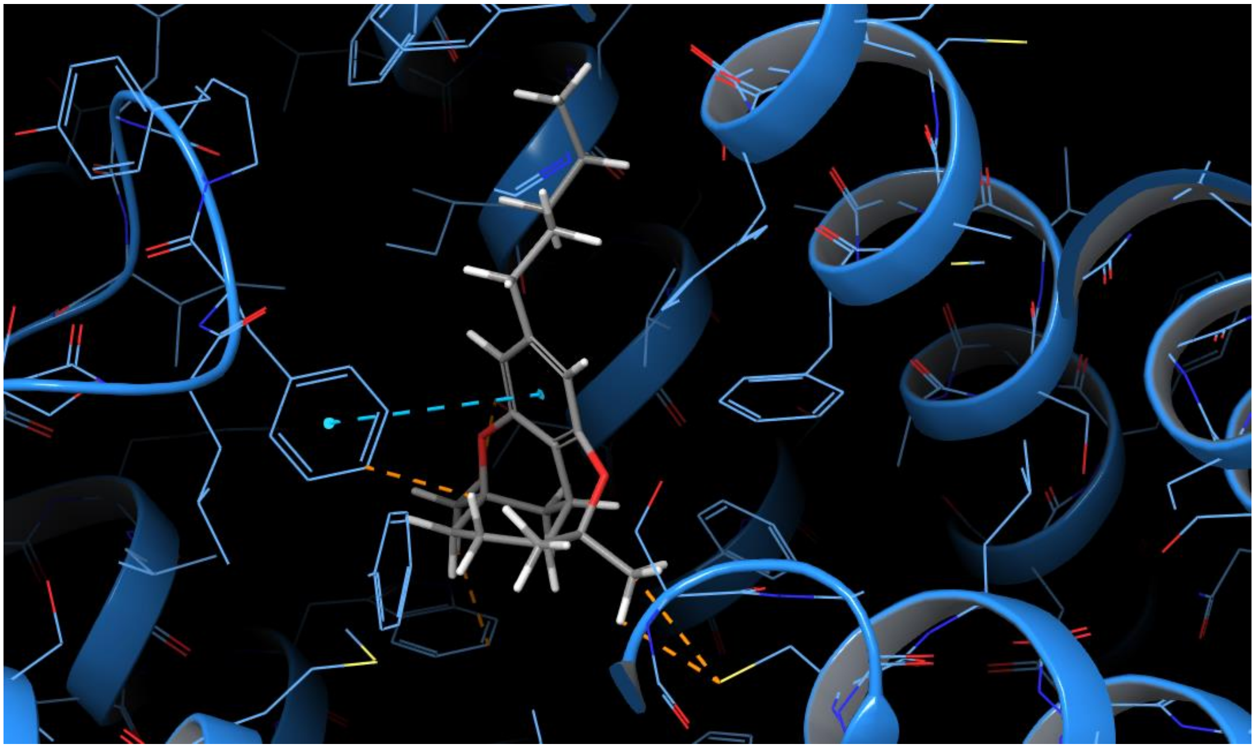

Figure S4.3.9. Three-dimensional rendering of (-)-CBT-C in the orthosteric site of the CB_2R utilizing SP modeling constraints. Blue dotted lines indicate π-π stacking and orange dotted lines indicate negative interactions.

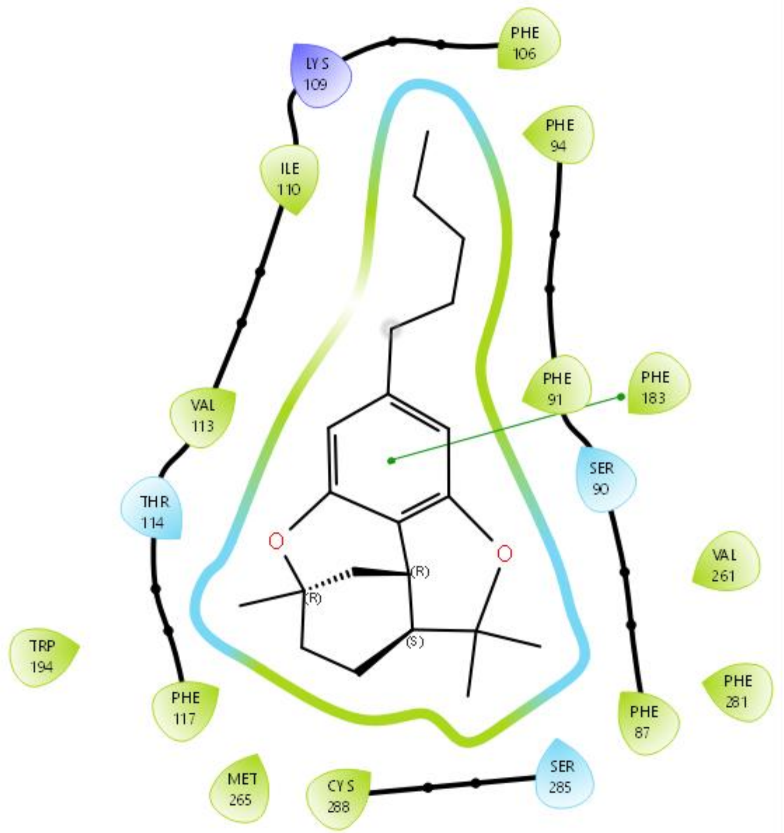

Figure S4.3.10. Two-dimensional rendering of (-)-CBT-C in the orthosteric site of the CB_2R utilizing SP modeling constraints. Green lines indicate π-π stacking.

Table S4.3.7. (-)-CBC in the orthosteric site of the CB_2R: SP model.

CB₂R SP model						
Computer Ranking	Ligand	Docking Score	Glide emodel	Highlighted Residue-Ligand Interactions	Distance of Interaction (Å)	Type of Interaction
6	(-)-CBC	-7.892	-51.054	Phe87-P	3.57	π-π stacking
				Phe87-AR	3.57	π-π stacking
				Phe183-P	4.17	π-π stacking

Distances were measured based on the closest atom to atom distance between a given residue and the ligand. Abbreviations: Aromatic ring (AR) and pyran-type ring (P).

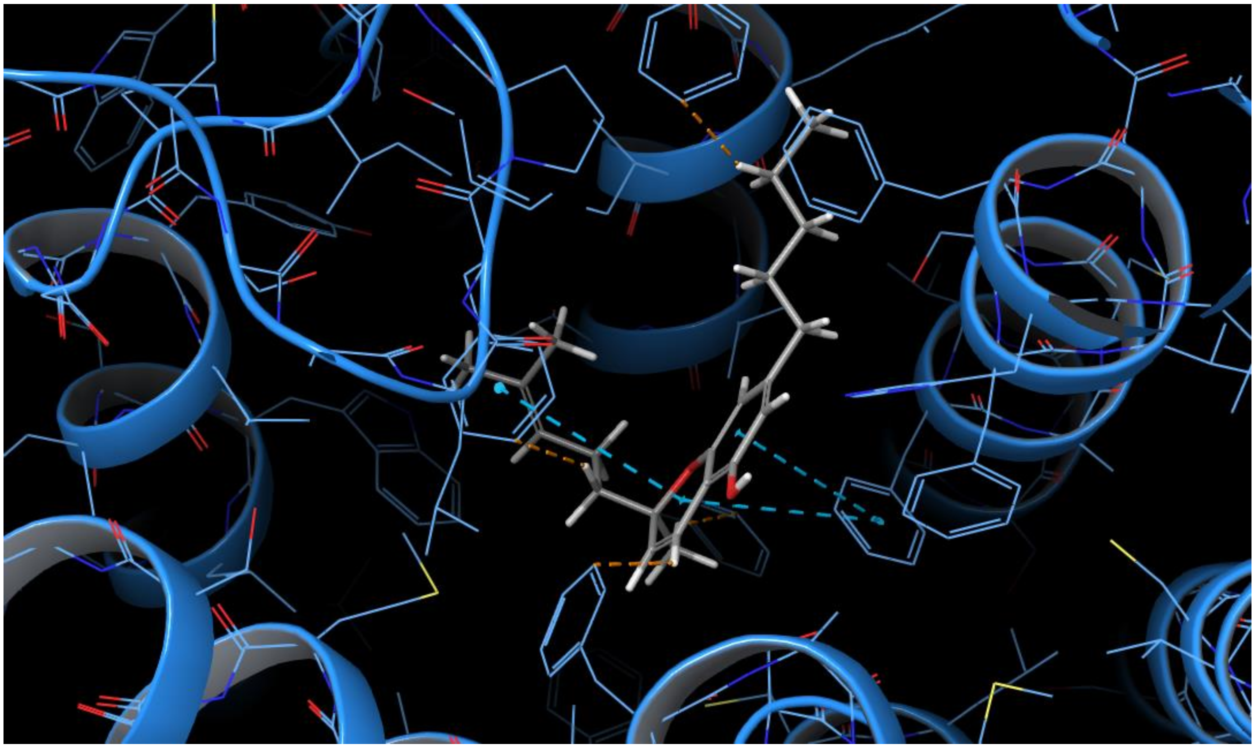

Figure S4.3.11. Three-dimensional rendering of (-)-CBC in the orthosteric site of the CB_2R utilizing SP modeling constraints. Blue dotted lines indicate π-π stacking and orange dotted lines indicate negative interactions.

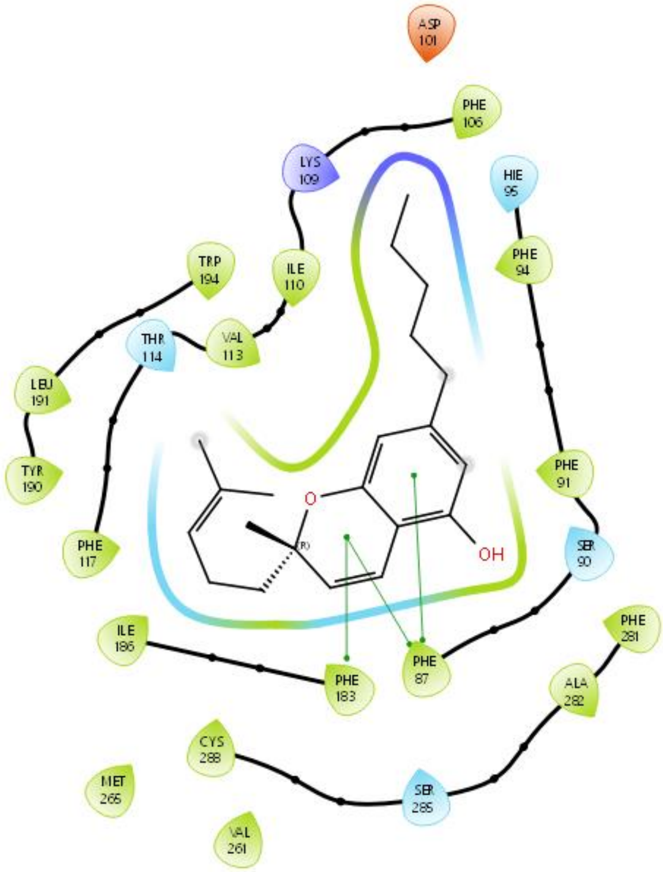

Figure S4.3.12. Two-dimensional rendering of (-)-CBC in the orthosteric site of the CB_2R utilizing SP modeling constraints. Green lines indicate π-π stacking.

Table S4.3.8. (R)-2'-hydroxy-(+)-cannabicitran in the orthosteric site of the CB_2R: SP model.

CB_2R SP model

Computer Ranking	Ligand	Docking Score	Glide emodel	Highlighted Residue-Ligand Interactions	Distance of Interaction (Å)	Type of Interaction
				Ser90-OH	2.67	H-bond
7	(R)-2'-OH-(+)-cannabicitran	-7.81	-44.543	Phe183-AR	3.63	π-π stacking
				Phe87-AR	3.91	π-π stacking

Distances were measured based on the closest atom to atom distance between a given residue and the ligand. Abbreviations: Aromatic ring (AR) and hydrogen bond (H-bond).

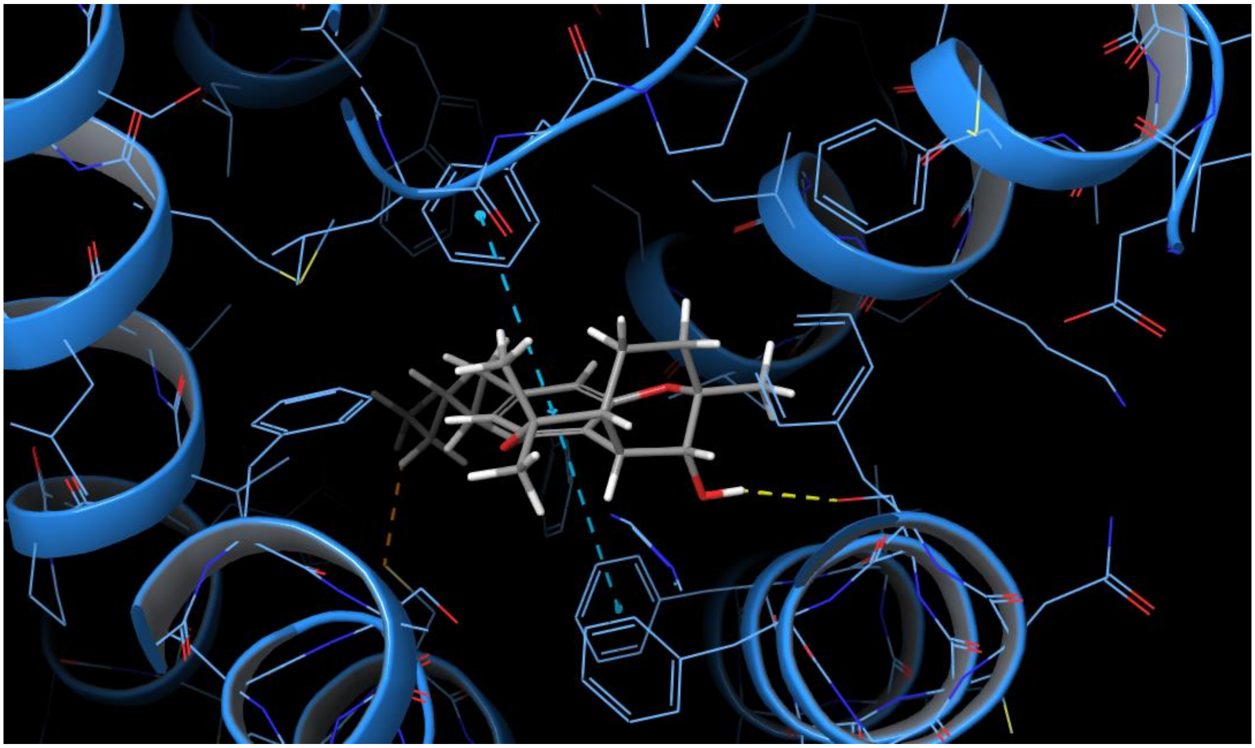

Figure S4.3.13. Three-dimensional rendering of (R)-2'-hydroxy-(+)-cannabicitran in the orthosteric site of the CB_2R utilizing SP modeling constraints. Blue dotted lines indicate π-π stacking, the yellow dotted line indicates a hydrogen bond, and orange dotted lines indicate negative interactions.

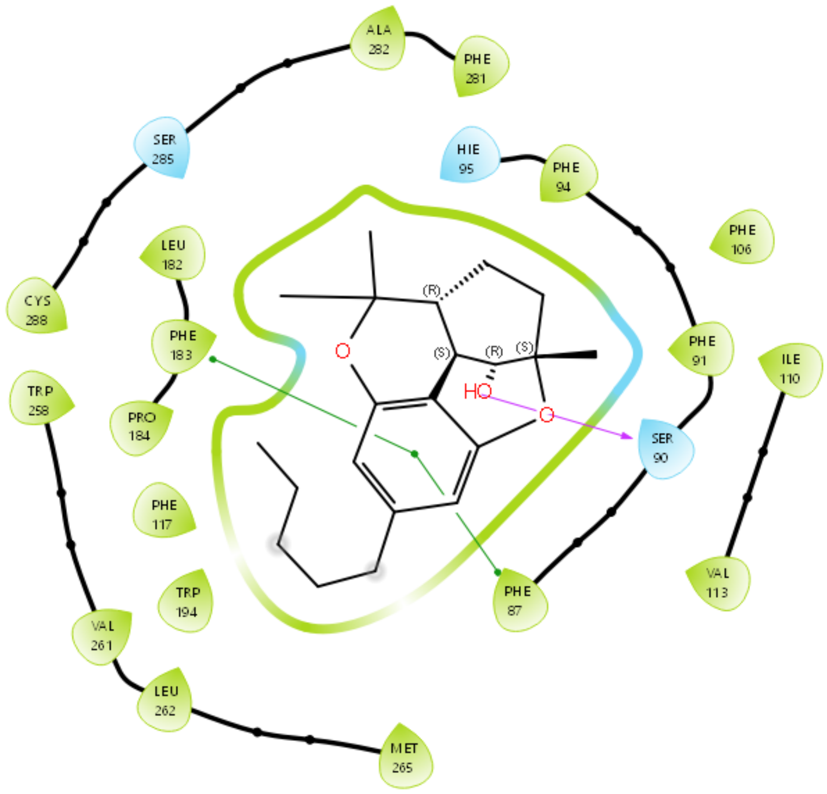

Figure S4.3.14. Two-dimensional rendering of (R)-2'-hydroxy-(+)-cannabicitran in the orthosteric site of the CB_2R utilizing SP modeling constraints. Green lines indicate π-π stacking and the pink arrow indicates a hydrogen bond.

Table S4.3.9. Ligand ranking and interactions in the orthosteric site of the CB_2R: XP model.

CB_2R XP model						
Computer Ranking	Ligand	Docking Score	Glide emodel	Highlighted Residue-Ligand Interactions	Distance of Interaction (Å)	Type of Interaction
1	(S)-2'-OH-(-)-cannabicitran	-10.651	-15.83	-	-	-
2	(-)-Δ^9-THC	-8.726	-44.287	Phe183-AR	3.36	π-π stacking

Distances were measured based on the closest atom to atom distance between a given residue and the ligand. Abbreviations: Aromatic ring (AR).

Table S4.3.10. (S)-2’-hydroxy-(-)-cannabicitran in the orthosteric site of the CB_2R: XP model.

		**CB_2R XP model**				
Computer Ranking	Ligand	Docking Score	Glide emodel	Highlighted Residue-Ligand Interactions	Distance of Interaction (Å)	Type of Interaction
1	(S)-2'-OH-(-)-cannabicitran	-10.651	-15.83	-	-	-

Distances were measured based on the closest atom to atom distance between a given residue and the ligand.

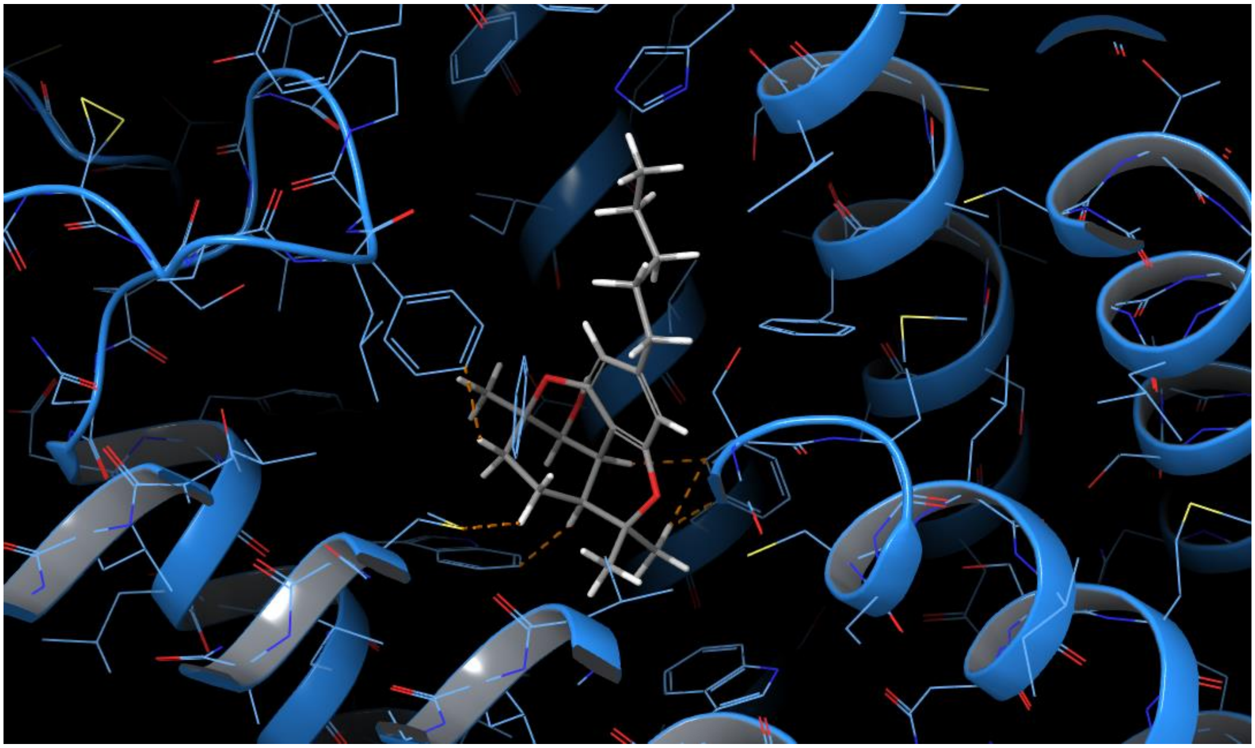

Figure S4.3.15. Three-dimensional rendering of (S)-2’-hydroxy-(-)-cannabicitran in the orthosteric site of the CB_2R utilizing XP modeling constraints. Orange dotted lines indicate negative interactions.

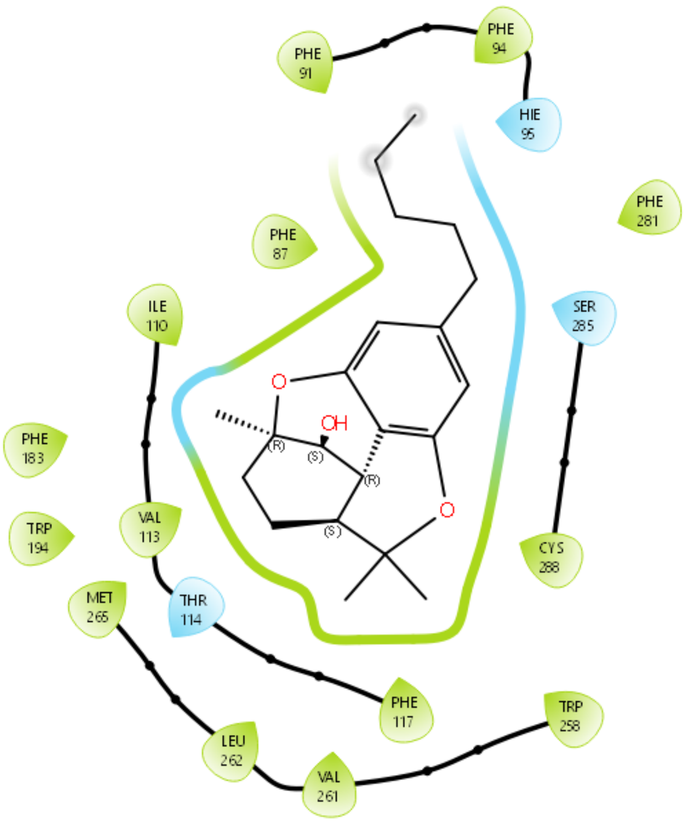

Figure S4.3.16. Two-dimensional rendering of (S)-2’-hydroxy-(-)-cannabicitran in the orthosteric site of the CB_2R utilizing XP modeling constraints.

Table S4.3.11. (-)-Δ[9]-THC in the orthosteric site of the CB_1R: XP model.

CB_2R XP model

Computer Ranking	Ligand	Docking Score	Glide emodel	Highlighted Residue-Ligand Interactions	Distance of Interaction (Å)	Type of Interaction
2	(-)-Δ[9]-THC	-8.726	-44.287	Phe183-AR	3.36	π-π stacking

Distances were measured based on the closest atom to atom distance between a given residue and the ligand. Abbreviations: Aromatic ring (AR).

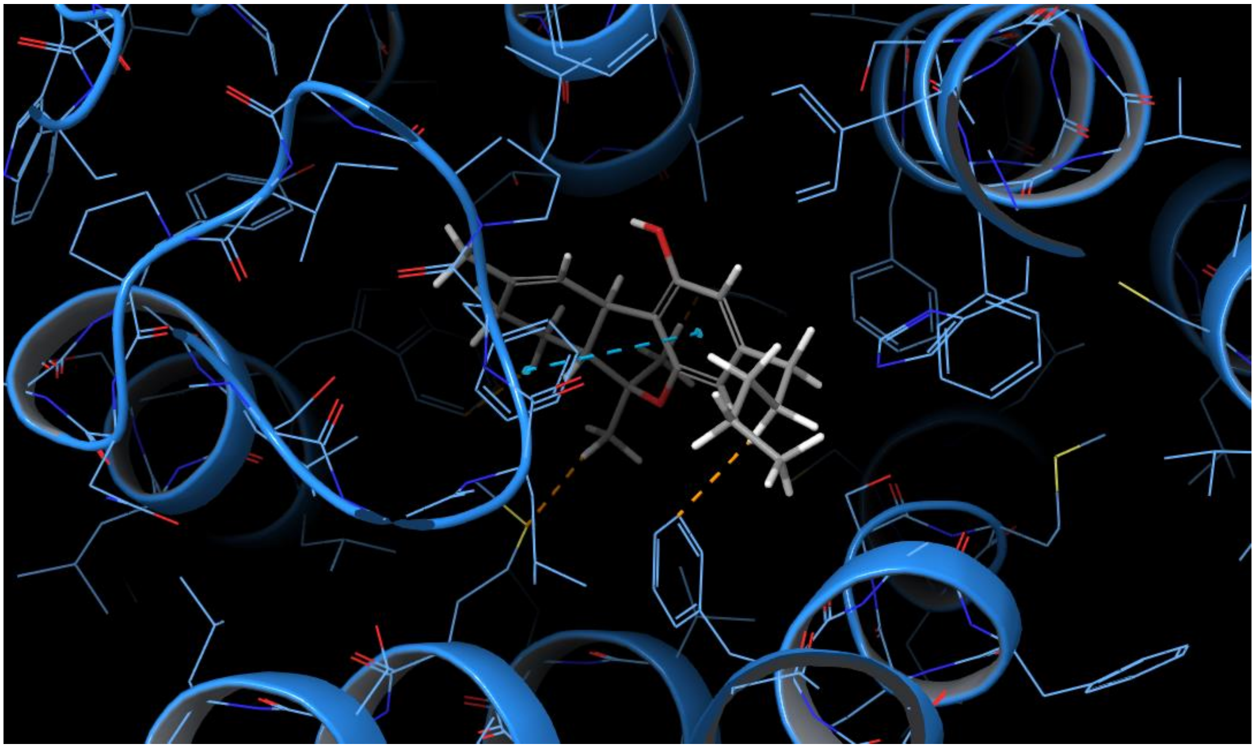

Figure S4.3.17. Three-dimensional rendering of (-)-Δ[9]-THC in the orthosteric site of the CB_2R utilizing XP modeling constraints. Blue dotted lines indicate π-π stacking and orange dotted lines indicate negative interactions.

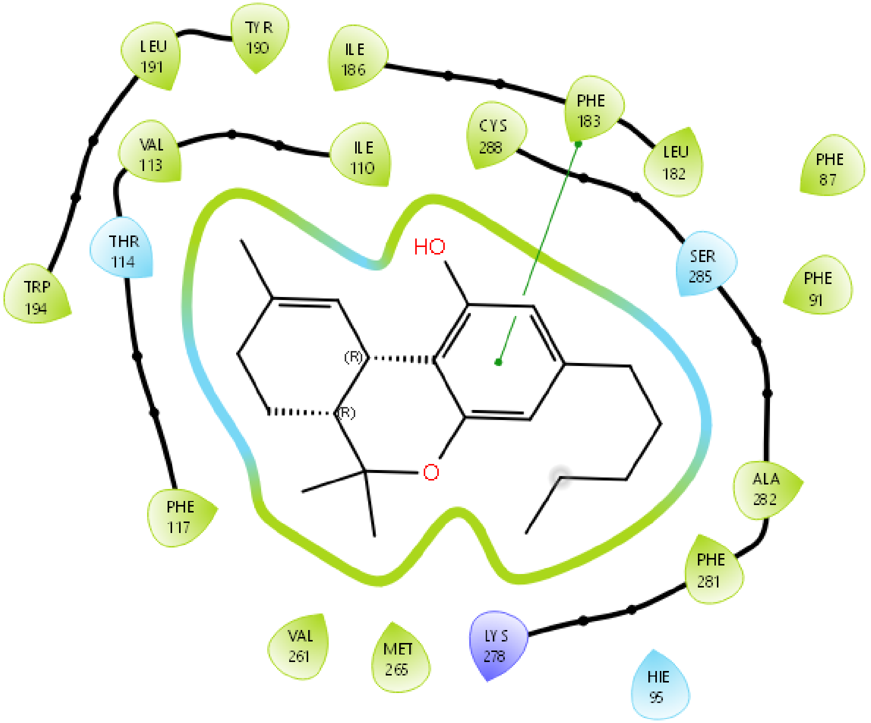

Figure S4.3.18. Two-dimensional rendering of (-)-Δ[9]-THC in the orthosteric site of the CB_2R utilizing XP modeling constraints. The green line indicates π-π stacking.

References

39. Friesner RA, Banks JL, Murphy RB, Halgren TA, Klicic JJ, Mainz DT, et al. Glide: A New Approach for Rapid, Accurate Docking and Scoring. 1. Method and Assessment of Docking Accuracy. J Med Chem. 2004;47(7):1739-49. doi: 10.1021/jm0306430.

40. Halgren TA, Murphy RB, Friesner RA, Beard HS, Frye LL, Pollard WT, et al. Glide: A New Approach for Rapid, Accurate Docking and Scoring. 2. Enrichment Factors in Database Screening. J Med Chem. 2004;47(7):1750-9. doi: 10.1021/jm030644s.

41. Friesner RA, Murphy RB, Repasky MP, Frye LL, Greenwood JR, Halgren TA, et al. Extra Precision Glide: Docking and Scoring Incorporating a Model of Hydrophobic Enclosure for Protein-Ligand Complexes. J Med Chem. 2006;49(21):6177-96. doi: 10.1021/jm051256o.

42. Hua T, Vemuri K, Nikas SP, Laprairie RB, Wu Y, Qu L, et al. Crystal structures of agonist-bound human cannabinoid receptor CB(1). Nature. 2017;547(7664):468-71. doi: 10.1038/nature23272.

43. Li X, Hua T, Vemuri K, Ho JH, Wu Y, Wu L, et al. Crystal Structure of the Human Cannabinoid Receptor CB2. Cell. 2019;176(3):459-67 e13. doi: 10.1016/j.cell.2018.12.011.

44. Jorgensen WL, Maxwell DS, Tirado-Rives J. Development and Testing of the OPLS All-Atom Force Field on Conformational Energetics and Properties of Organic Liquids. J Am Chem Soc. 1996;118(45):11225-36. doi: 10.1021/ja9621760.
